# Functional Alleles of Chicken BG Genes, Members of the Butyrophilin Gene Family, in Peripheral T Cells

**DOI:** 10.3389/fimmu.2018.00930

**Published:** 2018-05-01

**Authors:** Lei Chen, Michaela Fakiola, Karen Staines, Colin Butter, Jim Kaufman

**Affiliations:** ^1^Department of Pathology, University of Cambridge, Cambridge, United Kingdom; ^2^Pirbright Institute, Compton, United Kingdom; ^3^School of Life Sciences, University of Lincoln, Lincoln, United Kingdom; ^4^Department of Veterinary Medicine, University of Cambridge, Cambridge, United Kingdom

**Keywords:** B-G, membrane protein, adaptive immunity, innate immunity, B7 family

## Abstract

γδ T cells recognize a wide variety of ligands in mammals, among them members of the butyrophilin (BTN) family. Nothing is known about γδ T cell ligands in chickens, despite there being many such cells in blood and lymphoid tissues, as well as in mucosal surfaces. The major histocompatibility complex (MHC) of chickens was discovered because of polymorphic BG genes, part of the BTN family. All but two BG genes are located in the BG region, oriented head-to-tail so that unequal crossing-over has led to copy number variation (CNV) as well as hybrid (chimeric) genes, making it difficult to identify true alleles. One approach is to examine BG genes expressed in particular cell types, which likely have the same functions in different BG haplotypes and thus can be considered “functional alleles.” We cloned nearly full-length BG transcripts from peripheral T cells of four haplotypes (B2, B15, B19, and B21), and compared them to the BG genes of the B12 haplotype that previously were studied in detail. A dominant BG gene was found in each haplotype, but with significant levels of subdominant transcripts in three haplotypes (B2, B15, and B19). For three haplotypes (B15, B19, and B21), most sequences are closely-related to BG8, BG9, and BG12 from the B12 haplotype. We found that variation in the extracellular immunoglobulin-variable-like (Ig-V) domain is mostly localized to the membrane distal loops but without evidence for selection. However, variation in the cytoplasmic tail composed of many amino acid heptad repeats does appear to be selected (although not obviously localized), consistent with an intriguing clustering of charged and polar residues in an apparent α-helical coiled-coil. By contrast, the dominantly-expressed BG gene in the B2 haplotype is identical to BG13 from the B12 haplotype, and most of the subdominant sequences are from the BG5-BG7-BG11 clade. Moreover, alternative splicing leading to intron read-through results in dramatically truncated cytoplasmic tails, particularly for the dominantly-expressed BG gene of the B2 haplotype. The approach of examining “functional alleles” has yielded interesting data for closely-related genes, but also thrown up unexpected findings for at least one haplotype.

## Introduction

The chicken major histocompatibility complex (MHC) was first described as the B blood group, based on serological reactions mainly with the so-called BG antigen on erythrocytes. Later experiments showed that recombination events could separate most of the BG antigen reactivity in the BG region from the antigens encoded by classical class I and class II genes in the BF-BL region (1–5). The fact is that BG molecules, like class I and class II molecules, are highly polymorphic cell surface antigens with wide tissue distributions and encoded in the MHC led one eminent researcher to refer to them as the class IV antigens and to the early speculation that they might be the ligands of the newly discovered chicken γδ T cells, but various approaches to demonstrate this possibility failed (6, 7).

Now it is clear that some homologs of the BG molecules, such as the butyrophilin (BTN) and butyrophilin-like (BTNL) molecules, may indeed to be the ligands of mammalian γδ cells (8–11). The discovery of myelin oligodendrocyte glycoprotein (MOG) in the nervous system of rodents and of BTN in lipid droplets of cow milk (12–14) eventually led to the description of the BTN gene family. This BTN family includes BTN, BTNL, skin T cell (SKINT), and BG genes, based mainly on the sequence relationships of the immunoglobulin-variable-like (Ig-V) extracellular domain, and is overall part of the larger B7 gene family (15). Certain BTN family members are known to be involved in immunological reactions, including some expressed on T cells reported to be involved in negative co-stimulation and some expressed as heterodimers on epithelial cells involved in recognition by T cells with certain restricted γδ TCRs (8, 10, 16, 17).

There are similarities but also differences between the mammalian BTN family members and chicken BG molecules. Both the BTN and BG genes are multigene families with wide tissue distribution, some members being expressed on hemopoietic cells, and others being expressed on other cell types, particularly epithelial cells (8–11, 18, 19). Some BTN family genes are known to function as heterodimeric glycoproteins in recognition by mammalian γδ T cells (16, 17); BG molecules have long been known to be disulfide-linked dimers, although without apparent glycosylation, and the presence of homo- versus hetero-dimers has not been resolved (20–22). However, there are various intron–exon and domain organizations within the mammalian BTN family (8–11), none of which are identical to the BG genes (20, 23, 24). In particular, the cytoplasmic tails of mammalian BTN family members have only a few heptad repeats and typically end with a 30.2 (also called PRY-SPRY) domain; by comparison the BG molecules all have long cytoplasmic tails composed of many heptad repeats. Moreover, high serologic polymorphism, copy number variation (CNV) and rapid evolution of BG genes in the BG region have been reported compared with the mammalian BTN family members (24).

At the moment, it is not clear whether the polymorphism of the BG genes is functionally important. Comparison of alleles of BG loci was easy for the two singleton genes: a nearly monomorphic BG0 gene on chromosome 2 and the polymorphic BG1 gene in the BF-BL region on chromosome 16 (25). All other known BG genes are located head-to-tail in the BG region on chromosome 16, which renders them targets for apparent gene conversion (meaning that the polymorphism might be due to drift rather than selection) and also subject to unequal crossing-over (meaning that the CNV makes it hard to unequivocally identify orthologous alleles in different BG haplotypes) (24, 25). To approach these problems, we have assumed that the genes from different haplotypes expressed in particular cell types could be considered alleles in a functional sense. If such “functional alleles” could be reliably identified, then the sequences could be compared for amount and location of variation, and assessed for selection at the protein level.

The BG genes of the B12 haplotype are the most intensely studied, and one of the simplest patterns was from peripheral T cells, in which the BG9 gene was strongly expressed and the BG12 gene was weakly expressed, as assessed by reverse-transcriptase polymerase chain reaction (RT-PCR) with SS-TM primers that amplified the signal sequence to transmembrane region, followed by cloning and sequencing (24). In this study, we developed “HU” primers from near the beginning of the 5′ untranslated region (5′UTR) of hemopoietic (“H”) BG genes to near the end of the 3′ untranslated region (3′UTR) of all known (universal or “U”) BG genes, and sequenced the nearly full-length amplicons from four chicken lines with other B haplotypes: line 6_1_ (B2), line 15I (B15), line P2a (B19), and line N (B21). We expected to find a single or dominantly expressed BG gene in each haplotype that would be closely related to the BG9 gene found in the B12 haplotype, which would allow us to determine whether the sequence variation between haplotypes is localized and/or selected in the extracellular region, the cytoplasmic tail, both, or neither.

## Materials and Methods

### Chicken Lines and Haplotypes

Four lines of White Leghorn chickens were maintained under specific pathogen-free condition at the Pirbright Institute (formerly the Institute for Animal Health) in Compton, UK: line N, line P2a, line 15I, and line 6_1_, with the MHC haplotypes of B21, B19, B15, and B2, respectively. The history of these lines is described (26).

### Isolation of Cells

Peripheral blood was taken from wing veins with heparin and washed twice with cold PBS by centrifugation at 300 *g* at 4°C for 5 min and resuspension in cold PBS. Cells were counted using a hemocytometer, and around 5 × 10^7^ lymphocytic cells in 2 ml were stained at 4°C in the dark for 1 h using T cell specific antibodies [10 µl mouse anti-chicken CD4-FITC and 10 µl of mouse anti-chicken CD8b-FITC for lines N and P2a; 10 µl mouse anti-chicken CD4-RPE and 10 µl of mouse anti-chicken CD8-RPE for lines 15I and 6_1_ (all antibodies from Southern Biotech)]. Then the cells were washed 3–4 times with cold PBS and resuspended into 1 ml cold PBS for sorting, using magnetic-activated cell sorting (MACS, Miltenyi Biotec) for line N and P2a, and a DakoCytomation MoFlo MLS high-speed cell sorter (Beckman Coulter) for fluorescence-activated cell sorting (FACS, performed by Mr. Nigel Miller in the Department of Pathology) for line 15I and 6_1_.

### RNA Isolation, cDNA Synthesis, and PCR Amplification

Roughly 1 × 10^6^ sorted T cells were extracted for total RNA following the manufacturer’s protocol for the NucleoSpin RNA II RNA extraction kit (Machery-Nagel). First strand cDNA was produced from 5 to 10 ng RNA following the manufacturer’s protocol for the Maxima H Minus First Strand cDNA Synthesis Kit (ThermoFisher). Briefly, the RNA was mixed with oligo-(dT)_18_ primer and dNTP mixtures, heated at 65°C for 5 min, chilled on ice for 3 min, RT buffer and Maxima H Minus Enzyme Mix added, and the reaction mixture incubated at 55°C for 45 min, followed by 85°C for 45 min to inactivate the enzyme.

PCR amplification was carried out using Phusion^®^ Hot Start Flex DNA Polymerase (NEB) in a 50 µl reaction mixture with 0.5 µl (5–10 ng) cDNA, 200 µM total dNTPs (1 µl of 10 mM stock), Phusion buffer (10 µl of 5× stock), 1 U Phusion enzyme (0.5 µl of 200 U/ml), 250 nM forward primer and 250 nM reverse primer (both 1.25 µl of 10 µM stocks), and nuclease-free water (35.5 µl), and with reaction conditions of 2 min at 98°C, 35 cycles of 98°C 10 s, 66.5°C 20 s and 72°C 60 s, and finally 10 min at 72°C.

**Figure 1:**
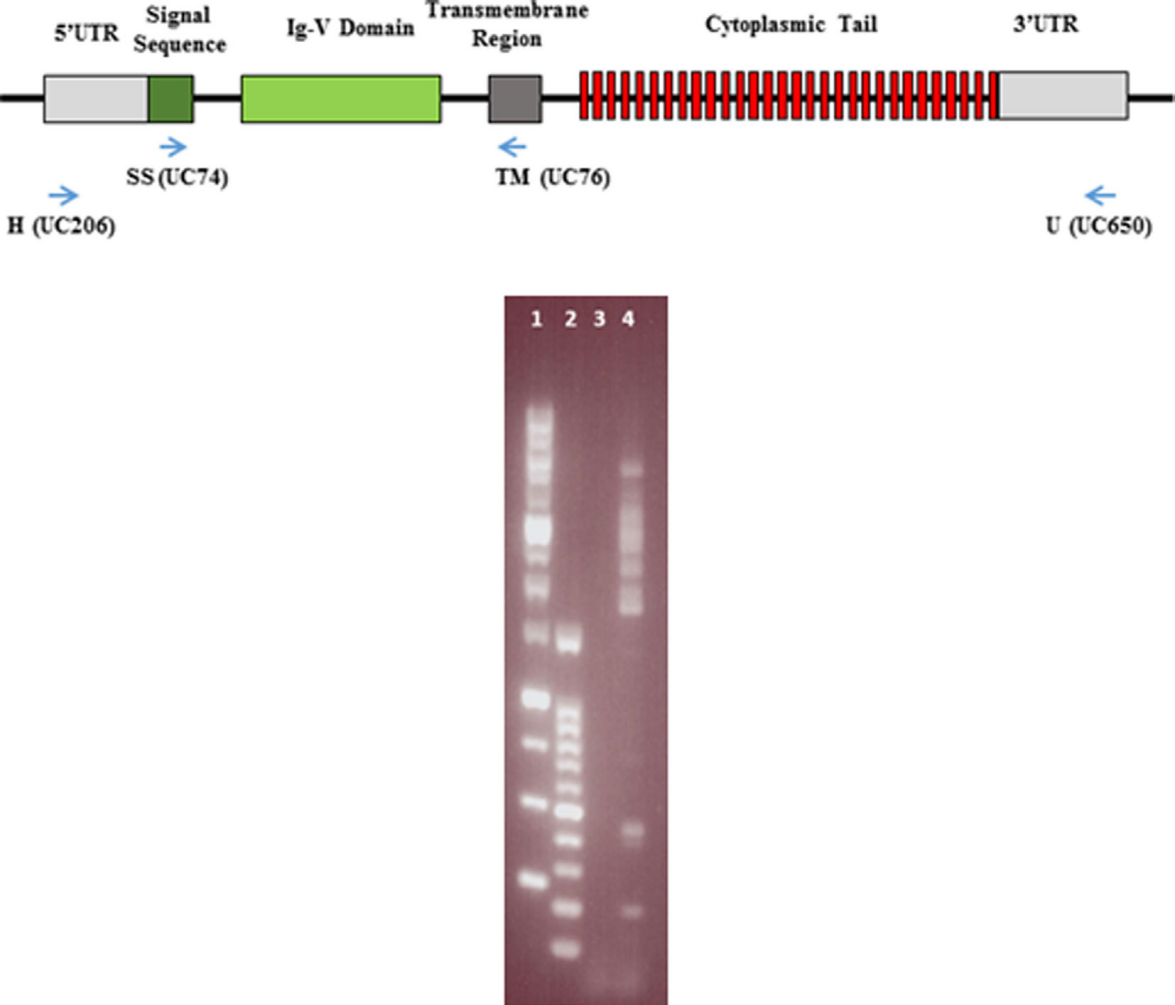
Representation of the intron–exon structure of a typical BG gene, with the encoded mRNA regions and protein domains indicated, as well as the location of the primers for the SS-TM and HU amplifications (top). Picture of agarose gel with the results of a representative amplification using HU primers (bottom). Lane 1, GeneDireX 1 kB markers (10, 8, 6, 5, 4, 3, 2.5, 2, 1.5, 1, 0.75, 0.5, and 0.25 kB); lane 2, GeneDireX 100 bp markers (1500, 1000, 900, 800, 700, 600, 500, 400, 300, 200, and 100 bp); lane 3, no template control PCR; lane 4, PCR with line N T cell cDNA.

The SS forward (UC74) and TM reverse (UC76) primers (5′CTCCTGCCTTATCTCRTGGCTCTGCAC 3′and 5′CACAGCCAGAGCCACYKTCCAG 3′) to amplify the signal sequence to transmembrane region (Figure 1) have been described (24). The H forward (UC206) primer (5′TCCGCTCGAGCTCTCTYCTCCTACAG3′) has been described (25). The U reverse (UC650) primer (5′TAACACCCAAAGCAGTTTTCTNCC3′) was designed by inspection. The location of the HU primer pair to amplify nearly full-length cDNA is indicated (Figure 1).

### Cloning and Sequencing

Several bands were generated after HU–PCR reaction, as illustrated by 1% agarose gel electrophoresis of a representative example (Figure 1). Amplification with SS-TM primers in pilot experiments revealed that all bands from 1,500 to 3,000 bp contained BG cDNA sequences. For the final experiments, a single region was cut out of the gel after shorter times of electrophoresis (20 min at 100 V), so that sequences of all these sizes were treated in parallel. DNA was extracted using the ISOLATE II PCR and Gel Kit (Bioline).

DNA fragments were cloned into the pJET vector (CloneJET PCR cloning kit, ThermoFisher), 92–96 colonies were picked for colony PCR using HU primers, and DNA from the 40–60 positive clones was prepared by Miniprep (PureLink Quick Plasmid Miniprep Kit, Invitrogen) and sent for dideoxy chain termination sequencing (DNA Sequencing Facility, Department of Biochemistry, University of Cambridge). Sequencing primers were T7 (5′ TAATACGACTCACTATAGGG 3′), pJETR (5′ AAGAACATCGATTTTCCATGGCAG 3′), UC699 (5′ TTTTCTATGATCATCC 3′), UC700 (5′ TTTTCTATGATCATCC 3′), UC701 (5′ TGGCTCTGCACYTCCTCS 3′), and UC703 (5′ TGRACCTGGAGGTGTCAG 3′). Sequencing identified 35–45 BG clones, some of which were BG0 and BG1 and therefore were not further analyzed. Names were given to the sequences from the remaining clones, according to the following convention: abbreviated line name, “T” for T cells, “BG,” a letter representing the exon 2 sequence with “a” being the most frequently detected exon 2 sequence (and “b” being the second most frequently detected exon 2 sequence, and so forth), a dash and then a number representing the alternative splicing variant with “1” being the most frequently detected clone (and “2” the second most frequently detected clone, and so forth). Some of these clones were eventually found to be chimeras, and were not further considered in the analyses, leading to 57 final sequences (Figure S1 in Supplementary Material).

### Sequence Analysis

Sequencing trace data were viewed, trimmed, and assembled in CLC DNA Workbench 5 (QIAGEN). Primary sequence alignments were carried out in CLC and finished sequences were exported into MEGA7^1^ for Clustal W alignment, from which the “.meg” file was generated and used for phylogenetic analysis using Neighbor Joining method in MEGA7 with bootstrap (1000) for phylogeny test. Sequence alignments were imported into BioEdit Sequence Alignment Editor,^2^ then exported as a “rich text with current shaded view setting,” opened in Word (Microsoft) and modified manually by adding annotations. Helical wheel analysis for the cytoplasmic tails was done using online program DrawCoil10^3^ and Figure 8 and Figure S5 in Supplementary Material were modified from the diagrams generated from this program. The model for Ig-V domain of BG8 in Figure 7 was built by Swiss-Model^4^ based on the template of the MOG molecule (PDB ID 3csp.1) sharing 40.35% identity in amino acid sequences. The structure was then viewed, edited, and annotated in PyMOL.^5^ All the other figures were designed and manipulated in Word or Powerpoint (Microsoft).

## Results

### One Dominant and Several Other BG Genes Are Expressed in Peripheral T Cells of Each B Haplotype, With Most Part of the BG8-BG9-BG12-BG13 Clade

Peripheral T cells were isolated from the blood of four chickens from lines with different B haplotypes: line 6_1_ (B2) and line 15I (B15) by FACS and line P2a (B19) and line N (B21) by MACS, and both with a cocktail of monoclonal antibodies (mAb) to CD4 and CD8. Total RNA was converted to cDNA using reverse-transcriptase and an oligo-dT primer, and then nearly full-length transcripts were amplified by PCR using HU primers, cloned and sequenced on both strands. Two independent PCR reactions were analysed, and for line 6_1_ (B2) a third PCR reaction was carried out using SS-TM primers, expected to detect all BG transcripts.

**Figure 2:**
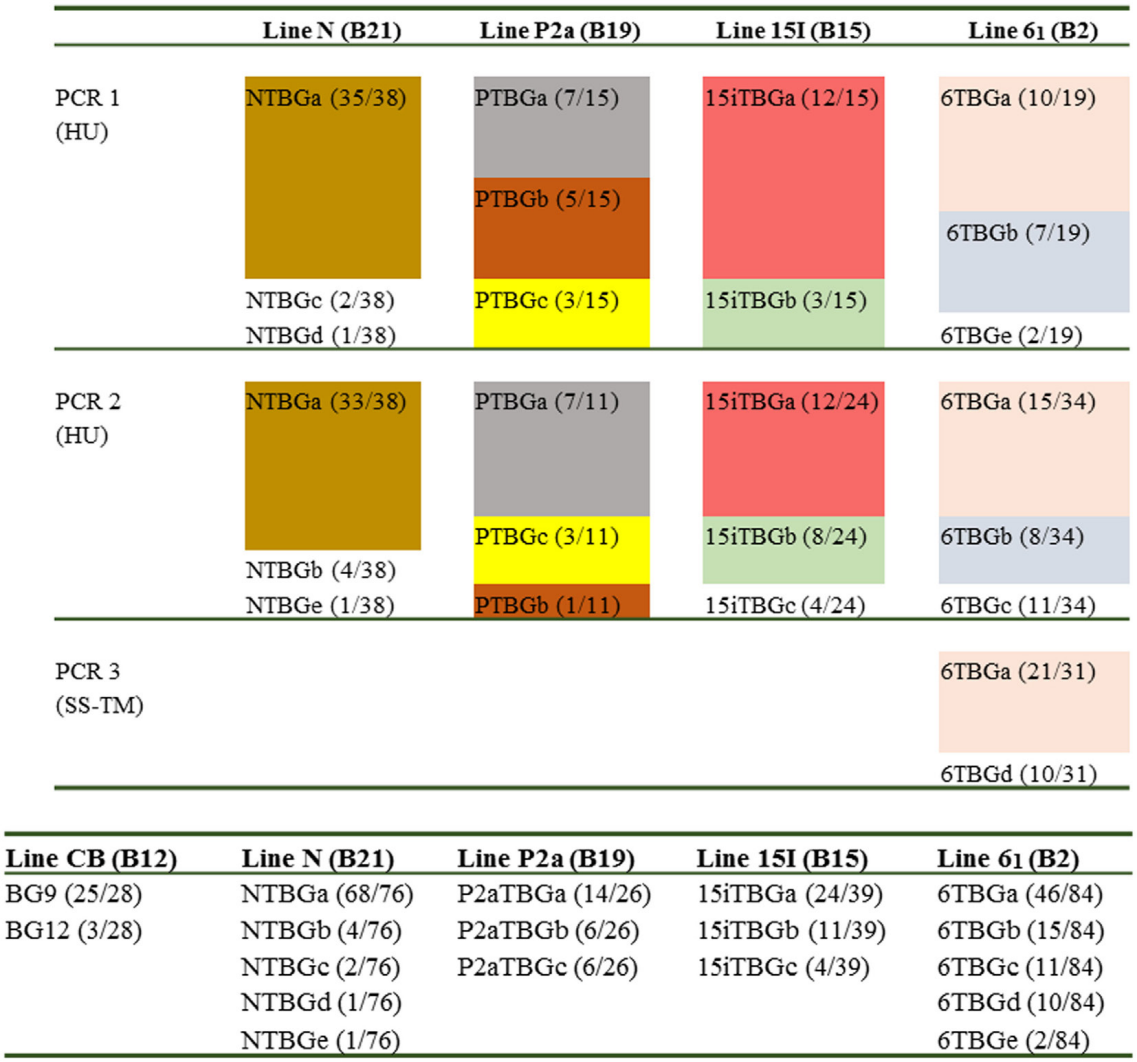
Overall results for the number of genes (based on exon 2 sequences) amplified from cDNA preparations derived from blood T cells isolated from various chicken lines (with different B haplotypes): line CB (B12), line N (B21), line P2a (B19), line 15I (B15), and line 6_1_ (B2). Top panel, independent amplifications from the four chicken lines; HU, hemopoietic forward and “universal” reverse primers to give nearly full-length sequences; SS–TM, signal sequence forward and transmembrane reverse primers to give SS, extracellular Ig-V domain and TM regions. Different colors indicate different exon 2 sequences, except those sequences that are only found in one PCR reaction. Names follow the convention: abbreviated line name, “T” for T cells, “BG” and a letter representing the exon 2 sequence with “a” being the most frequently detected exon 2 sequence (and “b” being the second most frequently detected exon 2 sequence, and so forth); numbers in parentheses indicate the number of clones found for a particular exon 2 sequence out of the total number for the particular PCR reaction. Bottom panel, the total results for four chicken lines from this paper and for the CB line (B12) from Ref. (24).

For each chicken line, 26–84 BG cDNA clones (excluding BG0 and BG1 clones) were isolated and then sequenced with a variety of primers, with the reads assembled and analysed (Figure 2). Fifty-seven unique sequences were found (Figure S1 in Supplementary Material). Assuming that each unique sequence of the extracellular Ig-V domain (encoded by exon 2) corresponds to a gene, the 57 unique sequences originate from 16 genes, with 3–5 genes expressed in each haplotype, none of which were shared between any two of the four haplotypes (Figure 2). Comparison of the nearly full-length sequences within each gene based on exon 2 sequences revealed that almost all differences were due to alternative splicing events in the cytoplasmic tail, which will be further described in a later section of the Results. However, some exon 2 sequences within a line differ in only one nucleotide and were only found in a single PCR (see Figure S1 in Supplementary Material). It seems likely that some of these clones are due to nucleotide mis-incorporation during amplification, but they were considered as separate genes since there are examples of separate genes with single nucleotide differences within the B12 haplotype (24). Based on the number of clones with different exon 2 sequences, there is one gene expressed more than the others in all four samples, but only in the N line (B21) was one gene really overwhelmingly dominant as found previously for the B12 haplotype (Figure 2).

**Figure 3:**
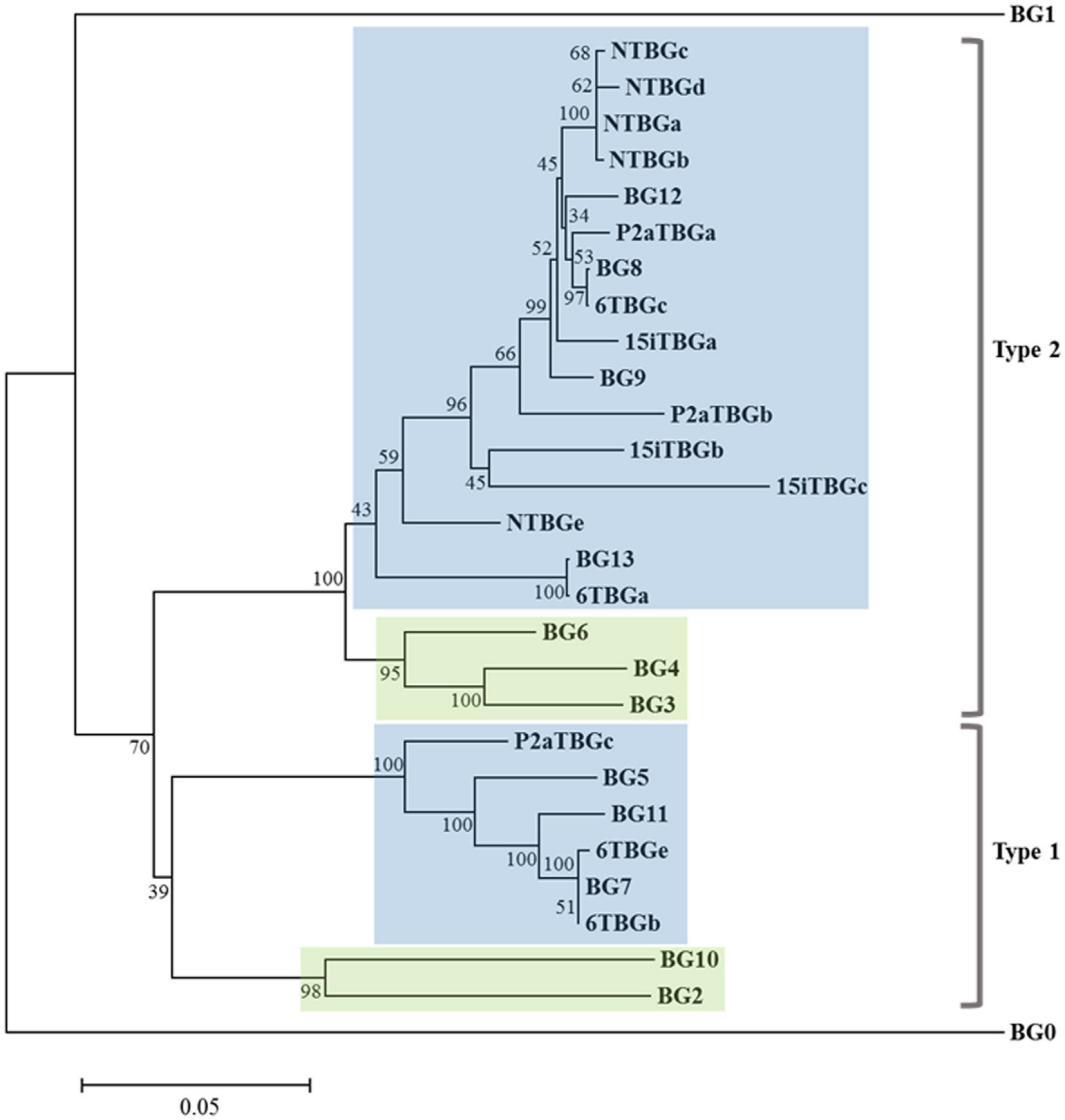
Phylogenetic tree of nucleotide sequences for the “(nearly) full-length conceptual transcripts” (i.e., exons without introns) for the 16 genes from four chicken lines identified in this paper, and for the 14 BG genes of the B12 haplotype from Ref. (24). Names of the transcripts follow the convention: abbreviated line name, “T” for T cells, “BG” and the letter “a” representing the most frequently detected clone from the most frequently detected exon 2 sequence (and “b” representing the most frequently detected clone from the second most frequently detected exon 2 sequence, and so forth). Names of the genes follow the convention “BG” and the number of the gene locus from the B12 haplotype. Indicated by color are those clades with 5′ ends of hemopoietic (blue) and tissue (green), and by brackets for 3′ ends of type 1 and type 2. Branch lengths are scaled by genetic distance, and percentage bootstrap values are indicated at the nodes. 6TBGd is not present in this tree since it was only detected by the SS–TM amplification, and some sequences may be due to nucleotide mis-incorporation during amplification (for instance, NTBGa may have given rise to NTBGb and NTBGc, see Figure S1 in Supplementary Material).

**Figure 4:**
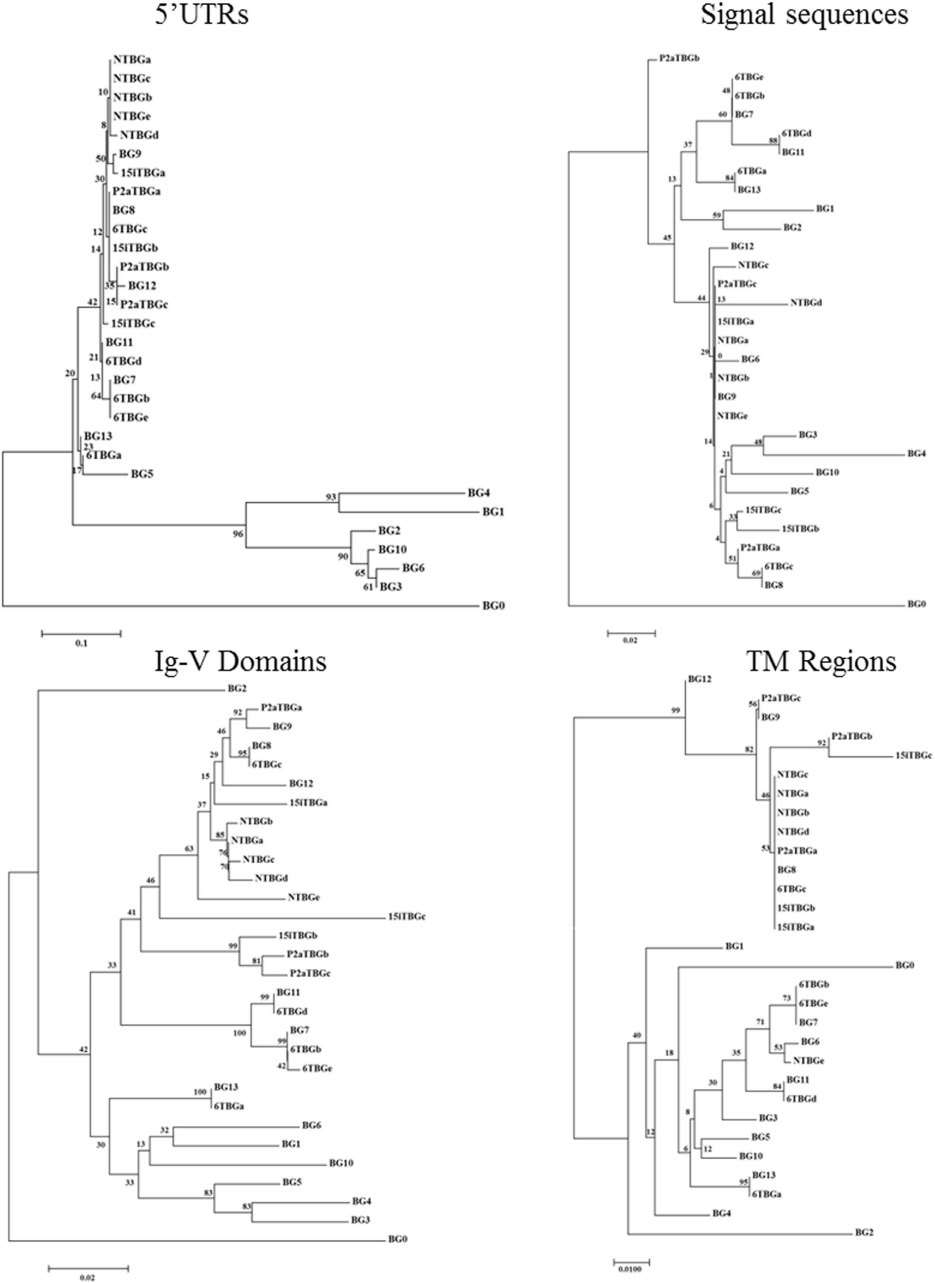

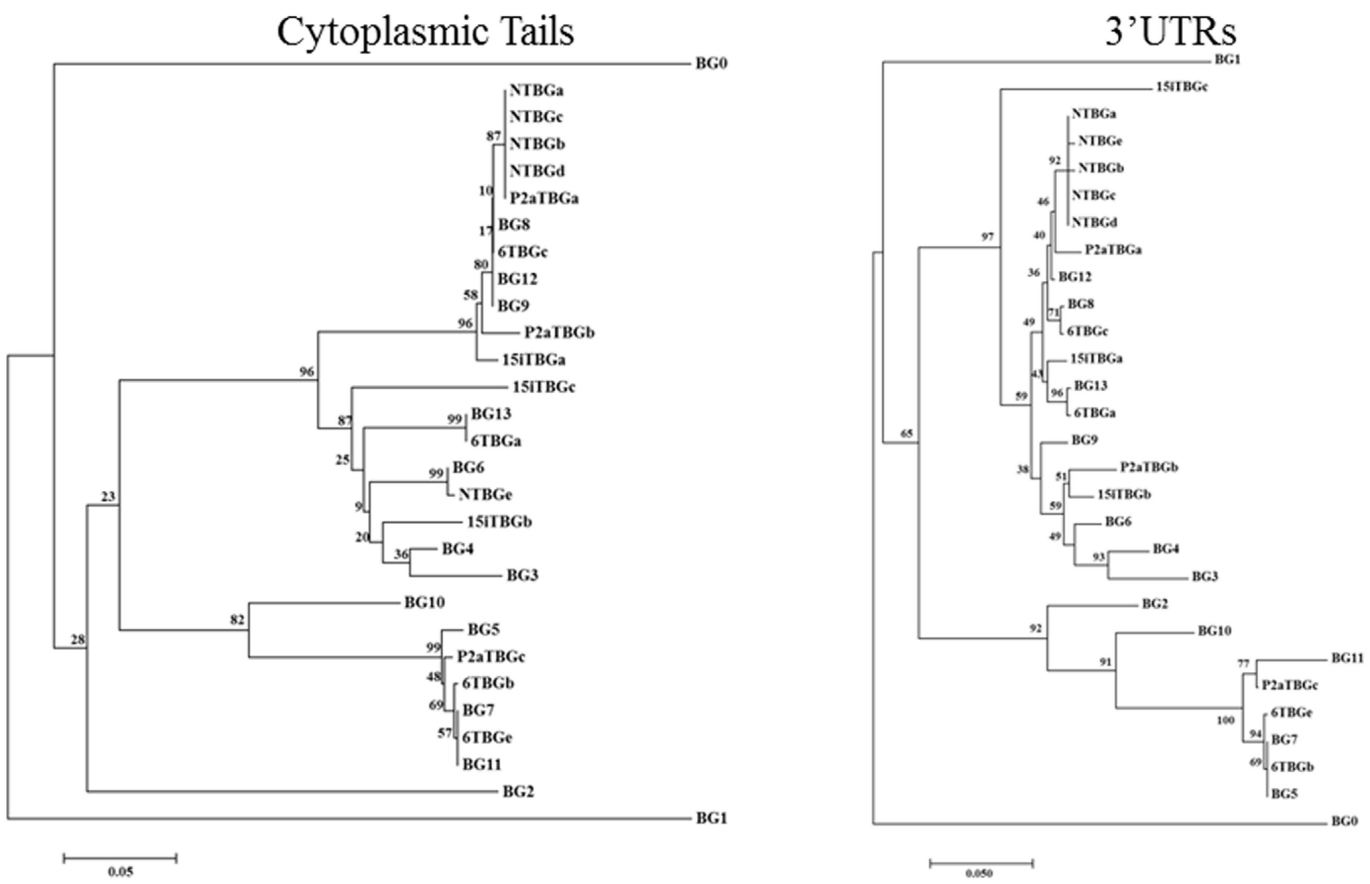
Phylogenetic trees of nucleotide sequences for portions of the “(nearly) full-length conceptual transcripts” (i.e., exons without introns) for the 16 genes (based on exon 2 sequences) from four chicken lines identified in this paper, and for the 14 BG genes of the B12 haplotype from Ref. (24). Trees include 5′ untranslated region (5′UTR) from exon 1, signal sequence from exon 1, exon 2 (two nucleotides from the signal sequence and then the nucleotides encoding the Ig-V domain), exon 3 (transmembrane region), the exons corresponding to the cytoplasmic tail (excluding any nucleotides in the final exon that encode amino acids), and the final exon [which is exactly the 3′ untranslated region (3′UTR) in some sequences, but for which the first nucleotides encode the last amino acids of the cytoplasmic tail in most sequences]. Other details are in the Figure 3.

Based on the intron–exon structures of BG genes (Figure 1) from the well-characterized B12 haplotype, the cDNA sequences from the four haplotypes could be organized conceptually into transcript sequences without introns, which could be used for the first stage of analysis. By comparison with the 14 BG genes of the B12 haplotype (24), the conceptual transcript sequences from these cDNA clones were mainly from the phylogenetic clade of BG8-BG9-BG12-BG13 genes of the B12 haplotype (Figure 3). The sequences of this clade have a 5′UTR characteristic of hemopoietic BG genes (as expected for genes amplified with an H forward primer) with a cytoplasmic tail and 3′UTR characteristic of the so-called type 2 sequence, quite different from the 5′UTR sequences of tissue BG genes and those genes with so-called type 1 cytoplasmic region and 3′UTR sequences (Figure 4).

**Figure 5:**
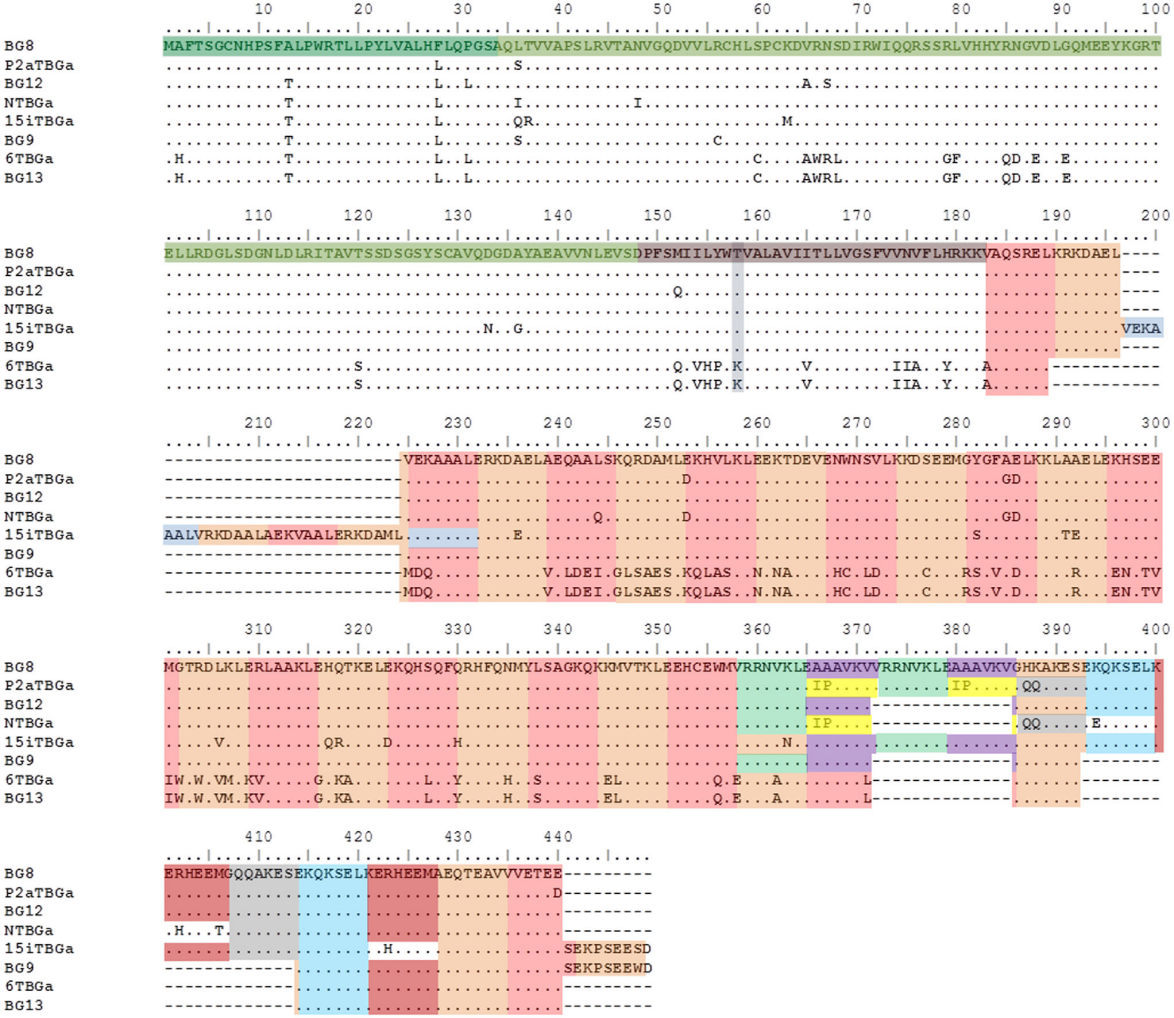
Alignment of amino acid sequences from the “(nearly) full-length conceptual transcripts” (i.e., exons without introns) for the dominantly expressed genes from four chicken lines identified in this paper, and for the appropriate BG genes (BG8, BG9, BG12, and BG13) of the B12 haplotype from Ref. (24). Names of the transcripts follow the convention: abbreviated line name, “T” for T cells, “BG” and the letter “a” representing the most frequently detected clone from the most frequently detected exon 2 sequence. Names of the genes follow the convention “BG” and the number of the gene locus from the B12 haplotype. Regions of the amino acid sequence are indicated with colors (but with amino acids split between two exons indicated by both colors): signal sequence, darker green; Ig-V domain, bright green; transmembrane region, darker brown (with lysine/threonine dimorphism in gray-blue); heptad repeats based on 21 nucleotide exons, alternating orange and light brown (except for some repeated exons in gray, light green, purple yellow, and light blue). Letters indicate amino acids by single letter code, dots indicate identities with BG8 sequence, dashes indicate no sequence present (deletion). Cytoplasmic tail of 6TBGa is conceptual, as alternative splicing leads to intron read-through and an early stop codon.

Some features of the BG genes from line 6_1_ (B2) are different from those of the other three haplotypes. Throughout the length of the sequences, the dominantly expressed conceptual transcripts (as defined in the previous paragraph) from B15, B19, and B21 (BG8, BG9, and BG12 gene sequences from B12) are much more closely related with each other than with the dominantly expressed conceptual transcript from B2 (and the BG13 gene sequence) (Figures 3 and 5). The BG13 gene had already been seen to have an apparent gene conversion in exon 2 (25), but based on these latest data, we now consider the BG8-BG9-BG12 clade as having a type 2a cytoplasmic tail, with the BG13 (and other sequences such as BG3, BG4, and BG6) having a type 2b cytoplasmic tail (but not for the 3′UTR, which are nearly identical in all these sequences). Another surprise was the fact that many of the cDNA sequences isolated from line 6_1_ (B2) are in fact identical (or very nearly so) to BG genes from the B12 haplotype (Figure 3), an unexpected finding for us since the B haplotype was originally defined by serology predominantly of the BG region. In fact, the serological identity of B2 and B12 molecules on erythrocytes was noted long ago (5), and confirmed by two-dimensional gel analysis (27). The mystery deepens with the realization that the dominantly expressed BG gene from B12 T cells is BG9 (despite the presence of a BG13 gene), whereas the dominantly expressed BG transcript from B2 T cells is identical in sequence to BG13, with no BG9 sequence found (Figures 2 and 3).

Finally, the subdominant cDNA sequences varied between haplotypes. Most of these subdominant sequences are also most closely related to the BG8, BG9, and BG12 sequences, but some are more closely related to the BG5-BG7-BG11 clade (Figures 3 and 4), which has 5′UTR sequences characteristic of hemopoietic BG genes but with a cytoplasmic tail and 3′UTR characteristic of the so-called type 1 sequence (24). In particular, of the four subdominant transcripts in line 6_1_ (B2), one is identical and another nearly identical to BG7 while a third (for which only the V domain sequence is complete) is identical to BG11 (Figures 3 and 4).

### The Dominantly Expressed BG Genes for Three Haplotypes Show Evidence for Clustering of Variation but Not Selection in the Extracellular Domain Compared With Selection but Not Clear Clustering in the Cytoplasmic Tail

All BG genes can be divided up into the 5′UTR and signal sequence encoded by exon 1, the Ig-V extracellular domain encoded by exon 2, the transmembrane region encoded in exon 3, a cytoplasmic tail of heptad repeats mostly encoded by many 21 nucleotide exons, and 3′UTR encoded within the final exon (Figure 1) (24). The phylogenetic relationships seen for exon 2 are true for the whole of the conceptual transcripts, except for the few that have a type 1 cytoplasmic tail and 3′UTR.

It is of interest to gain insight into the features of the sequences at the nucleotide and amino acid level, including the location and potential clustering of the sequence variation as well as any evidence for selection. As mentioned above, the dominantly expressed conceptual transcript from line 6_1_ (B2) is identical to the BG13 sequence of the B12 haplotype, so there is no allelic variation to consider (Figures 3 and 5). However, there is variation throughout the conceptual transcripts of the dominantly expressed cDNAs from the three other haplotypes, which can be compared with the BG genes of the B12 haplotype (Figure 5; Figure S3 in Supplementary Material).

The 5′UTR of the dominantly expressed BG sequences expressed in T cells, like all other hemopoietic BG genes, has a large indel compared with those BG genes of the B12 haplotype that are expressed primarily in tissues (Figure S3 in Supplementary Material). Only 15 positions out of 137 nucleotides in the 5′UTR (excluding the primer binding site) differ in one or another of the dominantly expressed sequences from the four haplotypes (including B2) as well as the BG8, BG9, BG12, and BG13 genes of the B12 haplotype, and this variation is of unknown significance.

**Figure 6:**
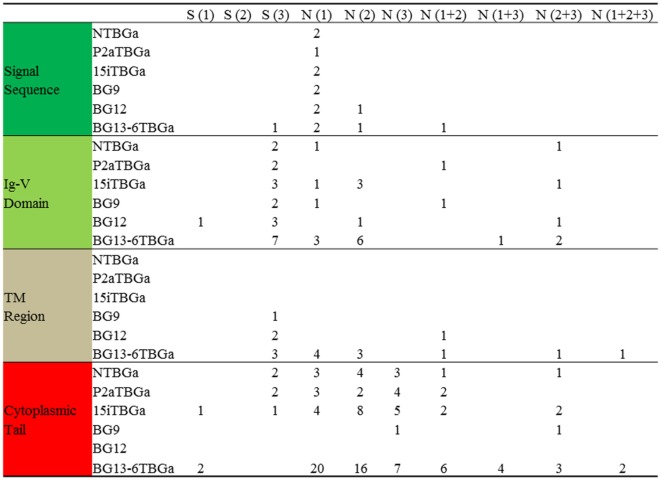
Compared with BG8 of the B12 haplotype, the number of silent and replacement changes by codon position for the “(nearly) full-length conceptual transcripts” (i.e., exons without introns) of the dominantly expressed genes from four chicken lines identified in this paper, and of the other appropriate BG genes (BG9, BG12, and BG13) of the B12 haplotype from Ref. (24). Names of the transcripts follow the convention: abbreviated line name, “T” for T cells, “BG” and the letter “a” representing the most frequently detected clone from the most frequently detected exon 2 sequence. Names of the genes follow the convention “BG” and the number of the gene locus from the B12 haplotype. Values are based on the alignments in Figure S4 in Supplementary Material, and amino acids from split codons at the edges of the exons are assigned to the exon with two of the three nucleotides of the codon (for instance, last amino acid of the signal sequence is assigned to the Ig-V domain, which in fact starts with glutamine in the mature protein).

In the portion of exon 1 encoding most of the signal sequence (Figure 6; Figure S4 in Supplementary Material), only 1–6 differences out of 99 nucleotides leading to 0–4 changes in 33 amino acids are found in the dominantly expressed sequences from the four haplotypes (including B2) as well as the BG8, BG9, BG12, and BG13 genes of the B12 haplotype. There is only one (silent) nucleotide change that fails to lead to an amino acid change, so the variation might appear to be selected. However, this variation does not change the overall hydrophobic sequence nor does it change the signal peptidase site of three small amino acids (the last codon of which is split, with the second and third positions located in exon 2).

**Figure 7:**
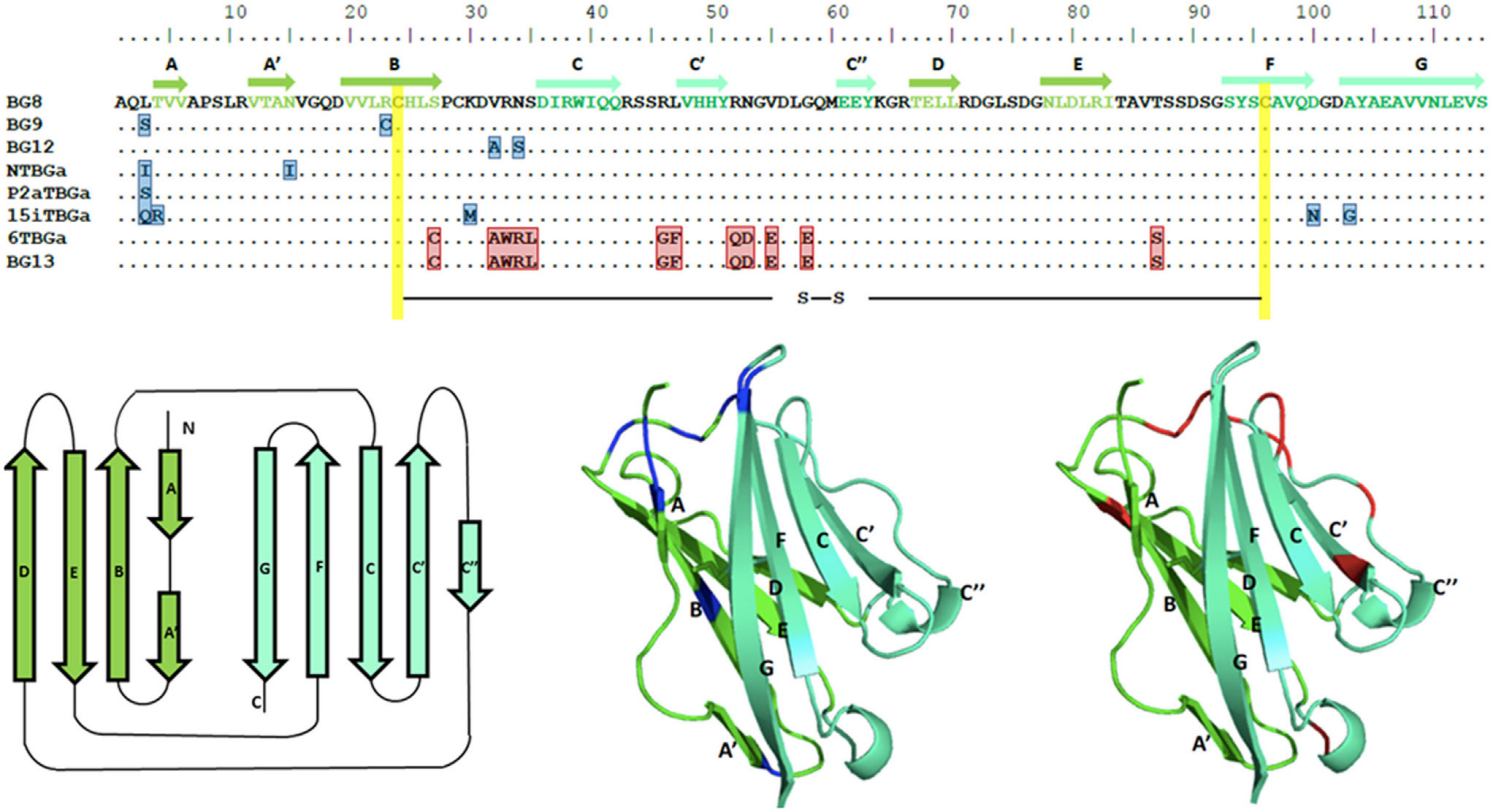
Alignment of amino acid sequences for the Ig-V domains of the dominantly expressed genes from four chicken lines identified in this paper, and for the appropriate genes of the B12 haplotype from Ref. (24), along with structural models of the Ig-V domains with the location of variation compared with the BG8 sequence of the B12 haplotype indicated. Names of the transcripts follow the convention: abbreviated line name, “T” for T cells, “BG” and the letter “a” representing the most frequently detected exon 2 sequence. Names of the genes follow the convention “BG” and the number of the gene locus from the B12 haplotype. In the top panel, letters indicate amino acids by single letter code, dots indicate identities with BG8 sequence, residues that differ from BG8 are boxed in blue for the three lines, and red for line 6_1_ and BG13; yellow indicates the intra-domain cysteines. The β-strands of the V region are indicated by arrows in the top panel, and are colored dark green for one face of the domain and light green for the other face. The same color scheme is used for the three panels below, with the positions of residues for the three lines (and BG9 and BG12) that differ from BG8 colored blue in the middle panel, and positions of residues for line 6_1_ and BG13 that differ from BG8 colored red in the right hand panel.

Compared with the BG8 gene of the B12 haplotype, the variation in the part of exon 2 encoding the extracellular Ig-V domain ranges from 4 to 9 differences out of 342 nucleotides leading to 1–5 changes in 114 amino acids in the three haplotypes (B15, B19, and B21), and 22 nucleotides and 12 amino acids for line 6_1_ (B2) and BG13 (B12) (Figure 7; Figure S4 in Supplementary Material). The location of the variation is not clustered along the sequence, but for the three haplotypes (and BG8, BG9, and BG12 of the B12 haplotype), a structural model (Figure 7) shows that nearly all the amino acid variation is located in the membrane distal loops presumably pointing away from the cell surface, with one position in the β-strands and one in the loops underneath the Ig-V domain. For line 6_1_ (B2) and BG13 (B12), there is more variation away from the distal loops. However, there was no change in the cysteines that form the intra-domain disulfide bond, or the cysteine located in the equivalent of complementarity determining region 1 (CDR1) that form a disulfide bond between the two chains of a BG dimer.

Comparison of the codons in the extracellular Ig-V domain for which there is nucleotide variation (Figure 6; Figure S4 in Supplementary Material) shows that for each sequence of the three haplotypes, the nucleotide changes that lead to no change in the amino acid (silent or synonymous changes) versus those that lead to a change in the amino acid (replacement or non-synonymous changes) range from two silent and one replacement change to three silent and five replacement changes. Comparison of the dominantly expressed BG from line 6_1_ (B2) and BG13 (B12) with the other three haplotypes (and BG8, BG9, and BG12) shows 7 silent and 12 replacement changes. Given that random changes would be expected to lead to only twice as many replacements as silent changes, these data are not consistent with strong selection.

There are two kinds of transmembrane regions described for BG genes, which are also found in the conceptual transcripts of the four haplotypes (Figure S3 in Supplementary Material). The dominantly expressed BG sequence for line 6_1_ (B2) is identical to BG13, with the transmembrane region bearing a lysine in the otherwise hydrophobic region. The dominantly expressed BG sequences from the other three haplotypes (and BG8, BG9, and BG12) all have with a threonine instead of the lysine along with nine other amino acid differences compared to BG13. There is no variation between the transmembrane region sequences of the three haplotypes (and only one amino acid difference in BG12). By contrast, there are only three silent nucleotide changes out of 17 total in line 6_1_ (B2) and BG13, and three codons have multiple nucleotide changes, again consistent with some selection between the BG8-BG9-BG12 sequences and the BG13 sequences (Figure 6; Figure S4 in Supplementary Material).

The cytoplasmic tail is composed of amino acid heptad repeats encoded by 21 nucleotide exons (with a few exons of 18 or 24 nucleotides), the numbers of which vary between BG genes (Figure 5; Figures S2 and S3 in Supplementary Material). Besides different numbers of the exons that code for these heptad repeats, the location of the first stop codon either in the penultimate exon or at the beginning of the last exon affects the numbers of repeats. The dominantly expressed BG sequence for line 6_1_ (B2) is like BG13, with 27 such exons in the conceptual transcript. The conceptual transcripts of the other three haplotypes have dominantly expressed BG sequences that are similar to BG8 (33 exons), BG9 (28 exons but a stop codon after a repeat in final exon, giving 29 apparent heptad repeats), and BG12 (31 exons): 37 exons for line 15I (B15) that has an apparent insertion of 4 exons but in addition a stop codon after a repeat in the final exon (giving 38 apparent repeats), 33 repeats for line P2a (B19), and 29 repeats for line N (B21).

The presence of amino acid heptad repeats encoded by 21 nucleotide exons strongly suggests that the two cytoplasmic tails of a BG dimer form an α-helical coiled-coil, similar to what is sometimes called a leucine zipper (28, 29). In such coiled-coils, the first and fourth amino acids in a true heptad repeat (which from here will be called *a* and *d* positions) act as the interface between the two chains, with some contribution by the neighboring amino acids (*e* and *g* positions) (30). To better understand the sequence features of the cytoplasmic tail, as well as location of any variation, representations of helical wheels were inspected.

**Figure 8:**
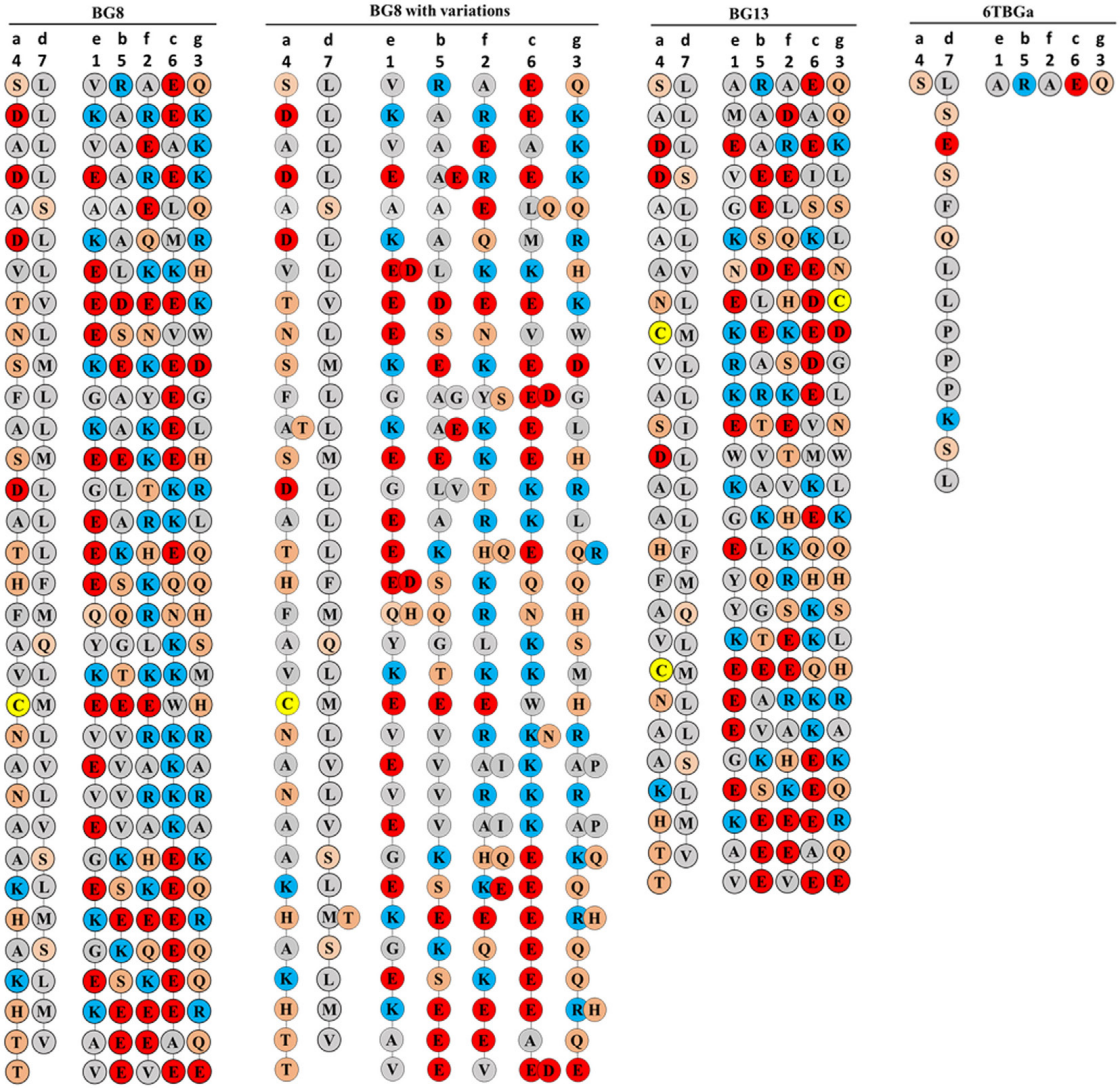
Coiled-coil representations of the cytoplasmic tails of BG8; BG8 with the different amino acids found in BG9 and BG12 of the B12 haplotype and in the “(nearly) full-length conceptual transcripts” (i.e., exons without introns) of the dominant sequences of line 15I (B15), line P2a (B19), line N (B21); B13 which is identical to the “(nearly) full-length conceptual transcript” (i.e., exons without introns) of the dominant sequence of line 6_1_ (B2); and the dominant sequence of line 6_1_ (B2) with the expected amino acid sequence from the most frequent clone using the real transcript (i.e., exons with the intron read-throughs leading to an early stop codon, sequence 6TBGa-1). The transmembrane region would be at the top of the page, so the C-terminus of the BG protein is at the bottom of the page. The positions of the seven codons in the 21 nucleotide repeat are indicated with numbers at the top, and the position of the seven amino acid positions of the “true heptad repeat” are indicated with letters. Colors of circles surrounding the amino acids (single letter code) indicate features of the amino acids (red, acidic; blue, basic; orange, polar; and gray, hydrophobic except for yellow, cysteine), with the understanding that these features do not correspond to full descriptions of the properties of the amino acids.

It seems unlikely that *a* and *d* amino acids forming the interface of the two chains in the coiled-coil would involve the first amino acid of each 21 nucleotide exon, since that amino acid is encoded by one nucleotide from the previous exon followed by two nucleotides from the exon under consideration, and thus the first amino acid encoded by this split codon would vary depending on the previous exon. In fact, the helical wheels of both BG8 and BG13 revealed a clear pattern (Figure 8): the amino acids from the fourth codon and the last codon of the 21 nucleotide repeat are mostly hydrophobic, presumably corresponding to the *a* and *d* amino acids of the true heptad repeat that would form a hydrophobic interface between the two chains. Moreover, there are many fewer hydrophobic amino acids at the other positions, with many of the amino acids from the first and third codon (corresponding to the *e* and *g* positions) charged, potentially allowing salt bridges between oppositely charged amino acids of the two chains (30). It is not immediately clear from the data whether the potential salt bridges might be for homodimers or for heterodimers with some of the subdominantly expressed chains. However, the charges in the five positions other than those forming the hydrophobic stripe between the chains are clustered into acidic, basic, and polar patches along the coiled-coil, with a particularly clear acidic patch at the C-terminus. Also striking is the presence of a cysteine residue in the same position of the cytoplasmic tail of the conceptual transcripts of all the dominantly expressed BG molecules.

There is no sequence variation in the cytoplasmic tail between the dominantly expressed BG conceptual transcripts for line 6_1_ (B2) and BG13 from the B12 haplotype, but the variation in the three other haplotypes, BG8, BG9, and BG12 is scattered along the sequence (except for an apparent insertion in the B15 sequence from line 15I), with only one *a* and one *d* position being variable out of 25 variable positions total (Figure 8; Figures S2 and S3 in Supplementary Material). This variation is all di-allelic, most of which is arguably conservative changes (A/T, M/T, E/D, Q/H, A/G, L/V, Y/S, A/I, and A/P) with only a few arguably radical changes (A/E, K/E, L/Q, K/N, Q/R, K/Q, and R/H). Decorating the coiled-coil representation of the cytoplasmic tail sequence revealed that much of the variation is located in two parts of the coil, 11–18 and 23–26 of 33 heptads (Figure 8), but whether this constitutes clustering is not yet clear. The cytoplasmic tail from the dominantly expressed conceptual transcript of line 6_1_ (B2) and from the BG13 gene (B12) is shorter (27 heptads) than the dominantly expressed genes from B15, B19, and B21 and the BG8, BG9, and BG12 genes from B12 haplotype (Figure 8). Interestingly, the actual cytoplasmic tails of the dominant and some subdominant sequences of line 6_1_ (B2) are much shorter (Figure 8; Figure S5 in Supplementary Material), as discussed below.

Unlike the protein coding regions including the cytoplasmic tail, the final exon (which includes the 3′UTR) of the dominantly expressed BG genes of all four haplotypes as well as the BG8, BG9, BG12, and BG13 genes are co-linear (except for a 20 nucleotide insertion in BG9 that is shared with most BG genes not in the BG8-BG9-BG12-BG13 clade) and nearly identical in sequence (Figures S3 and S4 in Supplementary Material). Including the 27 nucleotides that code for protein in BG9 and the dominantly expressed BG from line 15I (B15) but are untranslated in the other members of this clade, there are only 26 positions out of 411 nucleotides that vary between the eight sequences, with unknown significance.

### Alternative Splicing and Intron Read-Through Lead to Truncated Cytoplasmic Tails, Particularly for the Dominantly Expressed BG Gene From Line 6_1_ (B2)

**Figure 9:**
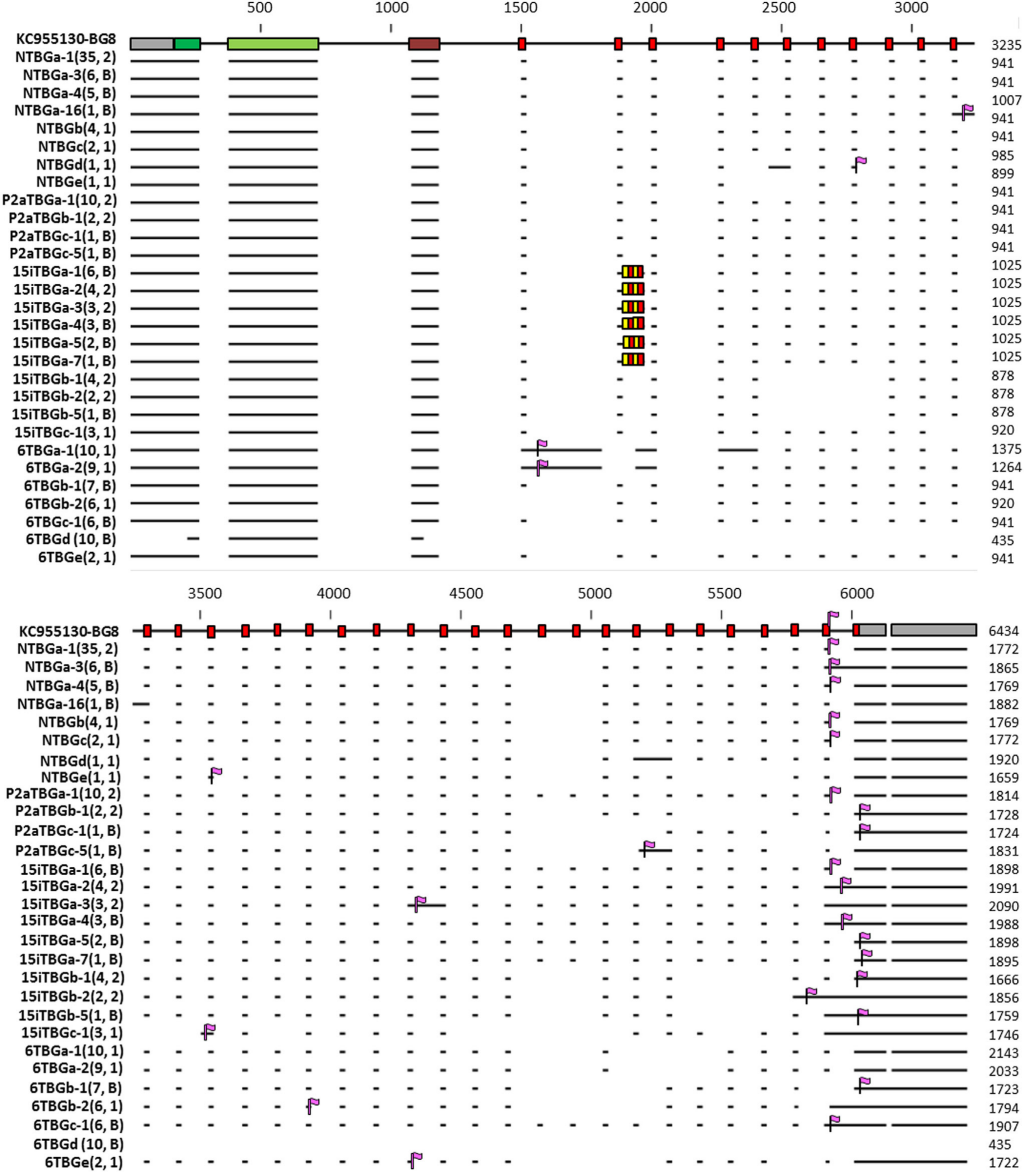
Representation of the intron–exon structure of the BG genes (based on exon 2 sequences) inferred from 29 sequences (representative of each of the 16 genes based on exon 2, as well as those alternatively expressed transcripts identified in more than one independent PCR) from line 6_1_ (B2), line 15I (B15), line P2a (B19), and line N (B21), indicating the actual mRNA transcripts as horizontal lines and stop codons as vertical purple flags. In 15iTBGa, the alternating red and yellow boxes indicate the four additional heptad repeats found for this gene, compared with BG8 (B12). Names of the transcripts follow the convention: abbreviated line name, “T” for T cells, “BG” and the letter “a” representing the most frequently detected clone from the most frequently detected exon 2 sequence (and “b” representing the most frequently detected clone from the second most frequently detected exon 2 sequence, and so forth), a dash and then a number representing the alternative splicing variant with “1” being the most frequently detected clone (and “2” the second most frequently detected clone, and so forth). Numbers in parentheses indicate the number of clones found for a particular full sequence, followed by the number of independent PCRs in which the sequence was identified (1, found in one PCR; 2, found in two PCRs; B, found in one PCR described in this paper and one using B cell cDNA, data not shown). The 29 sequences were deposited in GenBank, with the accession numbers as follows: NTBGa-1, MH156615; NTBGa-3, MH156616; NTBGa-4, MH156617; NTBGa-16, MH156618; NTBGb, MH156619; NTBGc, MH156620; NTBGd, MH156621; NTBGe, MH156622; P2aTBGa-1, MH156623; P2aTBGb-1, MH156624; P2aTBGc-1, MH156625; P2aTBGc-5, MH156626; 15iTBGa-1, MH156627; 15iTBGa-2, MH156628; 15iTBGa-3, MH156629; 15iTBGa-4, MH156630; 15iTBGa-5, MH156631; 15iTBGa-7, MH156632; 15iTBGb-1, MH156633; 15iTBGb-2, MH156634; 15iTBGb-5, MH156635; 15iTBGc-1, MH156636; 6TBGa-1, MH156637; 6TBGa-2, MH156638; 6TBGb-1, MH156639; 6TBGb-2, MH156640; 6TBGc-1, MH156641; 6TBGd, MH156642; 6TBGe, MH156643.

The analysis thus far has assumed that the RNA transcripts correspond to the exons as identified by their sequence features without any introns that were present, a minimal length for the mRNA. However, many of the 57 unique sequences actually isolated include stretches of sequence that are clearly introns, based on comparison with known genes in the B12 haplotype (Figure 9; Figures S1 and S3 in Supplementary Material).

Almost all of the retained introns lead to in-frame stop codons, some of which are long before the stop codon expected from the conceptual transcripts (Figure 9; Figures S1 and S3 in Supplementary Material). The dominantly expressed BG sequence from line 6_1_ (B2) retains the intron directly after the first 21 nucleotide exon, which truncates the cytoplasmic tail after only 13 amino acids (Figure 8; Figure S1 in Supplementary Material). Thus, the dominantly expressed BG sequence from line 6_1_ (B2) has a different sequence from the other three haplotypes, but also lacks the long cytoplasmic tail. Some of the subdominant sequences also have truncated cytoplasmic tails, some with clusters of cysteines (Figures 8 and 9; Figure S5 in Supplementary Material).

## Discussion

**Figure 10:**
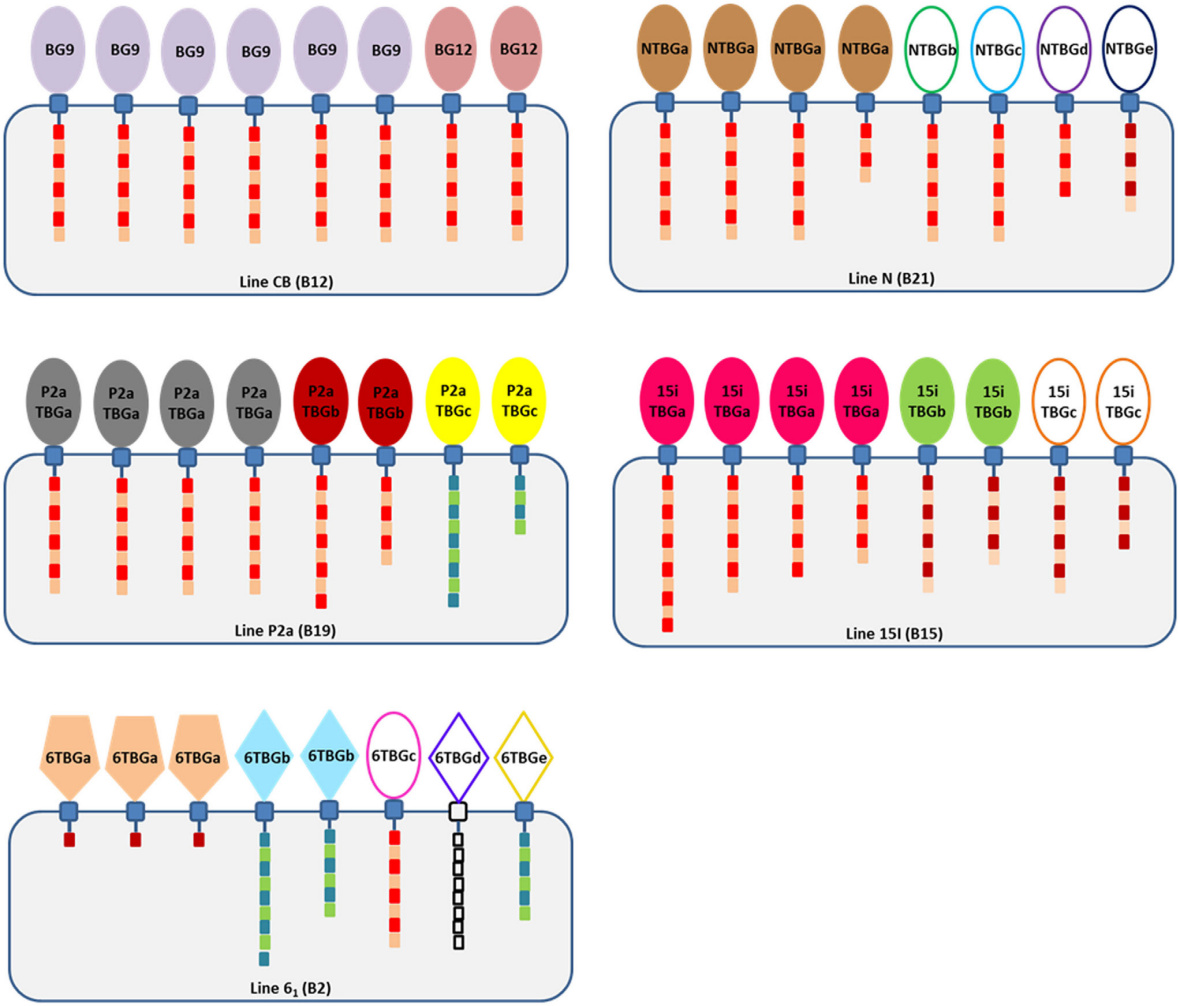
Cartoon summary of the findings, showing that there are no expressed sequences identical between the four chicken lines but that line 6_1_ has sequences identical to genes of the B12 haplotype (which, however, are not expressed in T cells of the B12 haplotype), that most expressed sequences are from the B8-B9-B12 clade although line 6_1_ (B2) has a dominantly expressed BG sequence of the BG13 clade and two subdominant sequences from the BG5-BG7-BG11 clade, and that cytoplasmic tails are mostly type 2a and that the length varies due to alternative splicing (intron read-through). The cartoon shows BG proteins in cells of each haplotype, with the numbers of each protein reflecting the ratio of different sequences in that haplotype. Extracellular Ig-V domains are represented by shapes to indicate relationship to clades of BG genes from the B12 haplotype (ovals, BG8-BG9-BG12 clade; pentagons, BG13 clade; and diamonds, BG5-BG7-BG11 clade) and by color (colors as in Figure 2, with those sequences found in only one PCR represented by ovals and diamonds not filled with color); cytoplasmic tails indicated by boxes representing heptad repeats, with lengths correlated with the length of the tail taking into account alternative splicing and with colors representing the clade (type 1, blue and green; type 2a, bright red and brown; type 2b, dark red and brown; and 6TBGd, white since no data are available).

To overcome the difficulty of identifying truly orthologous alleles in the ever-shifting panoply of BG genes in the BG region, we adopted the approach of looking at the BG transcripts in single cell types to identify “functional alleles.” Based on our limited examination of the transcripts in cells and tissues of the B12 haplotype (24), we began with peripheral T cells from chicken lines bearing four additional haplotypes. The overall results are summarized as a cartoon (Figure 10).

Based on our previous results, we expected a single dominantly expressed BG transcript (perhaps with another subdominant transcript at a low level) for each haplotype. We hoped that the transcripts from the five haplotypes would be similar enough that we could identify limited variation and determine whether such variation was clustered in regions of the protein with functional significance and/or under selective pressure. In particular, we wanted to ascertain whether such variation in the extracellular Ig-V domain and the cytoplasmic tail showed evidence for selected function, since there is no evidence that the serological polymorphism found in the extracellular Ig-V domain has functional significance while the two reported examples of function have been localized to the cytoplasmic tail. In fact, we found a series of surprises.

First, only line N (B21) cells had one really dominantly expressed BG gene, as we had found with the CB (B12) line. The other three lines with different haplotypes had one dominant gene expressed, but the subdominantly expressed genes were present in significant amounts. We were so concerned about this result that we carried out a third amplification of the cDNA from line 6_1_ (B2) using SS–TM primers (the same used for the B12 experiments), which gave the same dominantly expressed BG gene but a completely different subdominant BG gene compared with the amplifications with HU primers. Therefore, we are not completely convinced that our amplifications are without bias. Unbiased approaches such as RNAseq or proteomics might be suitable for answering this question.

The significant levels of subdominant BG transcripts in three of the four haplotypes did lead us to wonder whether the dominantly expressed BG protein in some haplotypes might associate with subdominant BG proteins to make heterodimers. Another possibility is that some or all of these expressed proteins might associate as heterodimers with BG0 or BG1 chains, which were found in all cells at significant amounts. We were unable to see an obvious pattern from our helical wheel analyses. Careful analyses at the protein level of *ex vivo* cells as well as flow cytometry and biochemical analysis of cells transfected with one versus two BG genes might help answer this question.

Second, three of the four haplotypes have dominantly expressed BG genes with sequences close enough with each other (and to the gene strongly expressed in the B12 haplotype) to allow good comparisons for allelic variability, but one haplotype is rather different. The dominantly expressed BG gene from all four haplotypes came from one clade of BG genes in B12 chickens (BG8-BG9-BG12-BG13, hemopoietic 5′ UTR with type 2 cytoplasmic tail and 3′UTR). The T cells from line 15I (B15), line P2a (B19), and line N (B21) expressed BG genes that are very closely related to the BG9 gene (and also to BG8 and BG12). By contrast, the dominantly expressed BG gene from line 6_1_ (B2) has many differences throughout the sequence (except in the 3′UTR). This B2 gene seems identical with the BG13 gene of the B12 haplotype (as presaged by serology of erythrocytes (5)), which is not expressed in B12 T cells (at least as assessed by amplification with the SS-TM primers (24)) despite being present in the B12 haplotype.

Among the subdominant BG genes expressed at significant levels, most are closely related to BG8, BG9, and BG12, with none that clustered with BG13. However, there were several subdominant BG genes whose overall sequences clustered with the BG5-BG7-BG11 clade (hemopoietic 5′ UTR with type 1 cytoplasmic tail and 3′UTR), three from line 6_1_ (B2) and one from line P2a (B19). The potential significance of these different sequences is unclear.

Third, the comparison of the Ig-V domains from closely related BG genes showed clustering of the variation but no evidence for selection at the protein level. Only low levels of variation were found in the Ig-V domain of the dominantly expressed BG genes of the three haplotypes along with the closely related BG8, BG9, and BG12 genes of the B12 haplotype. This variation is mainly localized to the distal loops, which could suggest selection for functional interactions with other molecules, but there was no evidence for selection of variation based on non-synonymous (replacement) versus synonymous (silent) changes; perhaps data from additional haplotypes will help. By contrast, the dominantly expressed BG gene of line 6_1_ (B2) is identical to the BG13 gene of the B12 haplotype, the differences with the other three haplotypes and three other genes of the B12 haplotype were scattered throughout the structure, and again there was no support for selection at the protein level.

Fourth, by contrast to the extracellular Ig-V domain, there was clear support for selection of variation in the cytoplasmic tail, which could be mapped to a conceptual model of an α-helical coiled-coil. The presence of exons with 21 nucleotide repeats encoding heptad amino acid repeats in a molecule known to be a dimer originally prompted the view that this portion of the molecule is a coiled-coil of two α-helices (20, 24, 31). This view was supported by the isolation of a soluble BG cytoplasmic tail as a molecule that displaces tropomyosin (32, 33), an actin-myosin regulator composed of a coiled-coil (34).

Visualization of the repeats in the BG cytoplasmic tails by forms of helical wheels (30) identified the amino acid encoded by the fourth codon and the last codon of each 21 nucleotide exon as predominantly hydrophobic, and thus likely to be the first (“*a*”) and fourth (“*d*”) amino acids of the true heptad. The fact that the true heptad repeat spans two exons was unexpected, but perhaps obvious in retrospect, given that the first codon is split and thus depends on two exons. This finding is most easily interpreted as a hydrophobic stripe on one α-helix interacting with a hydrophobic stripe on the other α-helix, buried between the two chains, whether as a homo- or a hetero-dimer. The other residues would project into the cytoplasm and, in the dominantly expressed BG genes, are present as patches of highly charged residues, along with a highly conserved cysteine. In some of the subdominantly expressed BG genes, there are clusters of cysteine residues. It is very likely that these various patches interact with other molecules, which might be identified by proteomics. Another possibility for the cysteines is modification, for instance palmitoylation (35) which could bring the α-helical coiled-coil to the underside of the membrane.

The variation in the cytoplasmic tail for the conceptual transcripts from the three haplotypes with similar BG sequences (and the BG8, BG9, and BG12 genes from the B12 haplotype) is predominantly located in two stretches at the five positions that are not in the hydrophobic stripe between the two α-helices of the coiled-coil. The functional significance of this variation is not clear, but the evidence from silent versus replacement substitutions supports selection for this variation. The only two known examples of function for BG molecules, regulation of actin-myosin by “zipper protein” in intestinal epithelial cells and effect of the BG1 gene on viral disease (32, 33, 36), are both associated with the cytoplasmic tail rather than the extracellular Ig-V domain, which may fit with the notion that the cytoplasmic tail is under selection for variation.

Fifth, many of the real transcripts had intron read-through that shortened the cytoplasmic tail compared to what was expected from the conceptual gene sequence, most dramatically truncating nearly the whole cytoplasmic tail of the dominantly expressed BG gene of line 6_1_ (B2). Such intron read-through, a form of alternative splicing, was first noticed long ago in the cytoplasmic tails encoded by BG cDNAs (20, 31). Some intron read-through seems to have become fixed, for example BG1 genes in which an active immunotyrosine inhibition motif (ITIM) is located in an exon bounded by two 21 nucleotide repeats (25, 36). A bioinformatic analysis of the 14 BG genes of the B12 haplotype found many read-through introns that led to in-frame stop codons, but no additional signaling motifs were obvious in those introns that read through in-frame (24).

The large proportion of transcripts with intron read-through was unexpected. One possibility that cannot be ruled out from our data is that these RNAs are incompletely spliced nuclear RNAs which would never be translocated to the cytoplasm or be translated. However, the RNA was primed with oligo-dT for the reverse transcription step and the amplicons are nearly full-length, so it seems most likely that these RNAs are polyadenylated. Ultimately, isolation and analysis of cytoplasmic or polysome RNA and/or analysis at the protein level is required to be sure that these transcripts encode real BG molecules.

Assuming that the intron read-through leads to truncated cytoplasmic tails in a real BG dimer, this kind of alternative splicing could be a way to regulate the interaction of the BG dimer with other molecules (i.e., the interactome) between different cell types. One possible interaction might be with orphan 30.2 (PRY-SPRY) domains (37), which would result in BG-30.2 complexes reminiscent of BTN and BTNL proteins. It is also possible that the truncated cytoplasmic tail of the dominantly expressed BG from line 6_1_ (B2) serves to ensure that the type 2b tail is not present in T cells, if it is type 2a tails that are necessary.

In summary, the work described in this report provides a first basis from which additional experiments can clarify the nature of the BG molecules found on the surface of different cell types, with the ultimate aim of determining the function of different domains of the molecules and the selection pressure under which they evolve. The unexpected results lead to many questions, which eventually will be answered in our quest to understand the structure, function and evolution of the BG genes and molecules.

## Data Availability Statement

Twenty-nine sequences generated in this work have been deposited in GenBank, and given accession numbers are from MH156615 to MH156643.

## Ethics Statement

This study was carried out in accordance with the recommendations of Home Office guidelines. The protocol was approved by the Local Ethics committee of the Pirbright Institute.

## Author Contributions

LC carried out most of the experimental procedures; MF, KS, and CB isolated the cells; LC and JK made the figures and wrote the text.

## Conflict of Interest Statement

The authors declare that the research was conducted in the absence of any commercial or financial relationships that could be construed as a potential conflict of interest.

## Footnotes

^1^http://www.megasoftware.net/ (Accessed: January, 2018).

^2^http://en.bio-soft.net/format/BioEdit.html (Accessed: January, 2018).

^3^http://www.grigoryanlab.org/drawcoil/ (Accessed: January, 2018).

^4^https://swissmodel.expasy.org/ (Accessed: January, 2018).

^5^https://pymol.org/2/ (Accessed: January, 2018).
