# Supplementary material for: Functional Alleles of Chicken BG Genes, Members of the Butyrophilin Gene Family, in Peripheral T Cells
Source: Front Immunol. 2018 May 1;9:930. doi: 10.3389/fimmu.2018.00930 (PMC5938342; doi:10.3389/fimmu.2018.00930)
Supplement: Supplementary file 1 [file Data_Sheet_1.PDF]

Supplementary Material

Functional alleles of chicken BG genes, members of the butyrophilin gene family, in peripheral T cells

Lei Chen, Michaela Fakiola, Karen Staines, Colin Butter, Jim Kaufman*

*** Correspondence:** Jim Kaufman, jfk31@cam.ac.uk

1 Supplementary Figures 1 to 5

1.1 Supplementary Figure S1

```
                             10        20        30        40        50        60        70        80        90       100
                    ....|....|....|....|....|....|....|....|....|....|....|....|....|....|....|....|....|....|....|....|
NTBGa-1(35,2)       TCCGCTCGAGCTCTCTCCTCCTACAGTTTCTGCCCTCATATTCTCCCCACACTTCTTCCCCATATTCTTTCCAAATCCTCTTCCCCATCTCCTCCATCGT
NTBGa-2(8,1)        ....................................................................................................
NTBGa-3(6,B)        ....................................................................................................
NTBGa-4(5,B)        ................T...................................................................................
NTBGa-6(2,1)        ....................................................................................................
NTBGa-7(1,1)        ....................................................................................................
NTBGa-8(1,1)        ....................................................................................................
NTBGa-10(1,1)       ....................................................................................................
NTBGa-11(1,1)       ....................................................................................................
NTBGa-12(1,1)       ....................................................................................................
NTBGa-13(1,1)       ................T...................................................................................
NTBGa-15(1,1)       ....................................................................................................
NTBGa-16(1,B)       ................T...................................................................................
NTBGb(4,1)          ................T...................................................................................
NTBGd(1,1)          ....................................................................................................
NTBGc(2,1)          ................T...................................................................................
NTBGe(1,1)          ................T...................................................................................
P2aTBGa-1(10,2)     ................T.........C...................A..G..C...........................................C...
P2aTBGa-2(3,1)      ................T.........C...................A..G..C...........................................C...
P2aTBGa-3(1,1)      ................T.........C...................A..G..C...........................................C...
P2aTBGb-1(2,2)      ................T.........C.....................................................................C...
P2aTBGb-2(2,1)      .T..............T.........C.....................................................................C...
P2aTBGb-3(1,1)      ................T.........C.....................................................................C...
P2aTBGb-4(1,1)      ................T.........C.....................................................................C...
P2aTBGc-1(1,B)      ................T.........C.....................................................................C...
P2aTBGc-2(1,1)      ..........................C.....................................................................C...
P2aTBGc-3(1,1)      ..........................C.....................................................................C...
P2aTBGc-4(1,1)      ................T.........C.....................................................................C...
P2aTBGc-6(1,1)      ................T.........C.....................................................................C...
P2aTBGc-5(1,B)      ................T.........C.....................................................................C...
15iTBGa-1(6,B)      ................T.........C.G.......................................................................
15iTBGa-2(4,2)      ..........................C.G.......................................................................
15iTBGa-3(3,2)      ................T.........C.G.......................................................................
15iTBGa-4(3,B)      ................T.........C.G.......................................................................
15iTBGa-5(2,B)      ................T.........C.G...................................................................C...
15iTBGa-6(2,1)      ..........................C.G.......................................................................
15iTBGa-7(1,B)      ..........................C.G.......................................................................
15iTBGa-8(1,1)      ................T.........C.G.......................................................................
15iTBGa-9(1,1)      ................T.........C............TA........TT.................................................
15iTBGb-1(4,2)      ..........................C.G...................................................................C...
15iTBGb-2(2,2)      ................T.........C.G...................................................................C...
15iTBGb-4(1,1)      ................T.........C.G...................................................................C...
15iTBGb-5(1,B)      ................T.........C.G...................................................................C...
15iTBGb-7(1,1)      ................T.........C.G...................................................................C...
15iTBGc-1(3,1)      ..........................C.......................................................................A.
6TBGa-1(10,1)       ................T............T...........A.............---................................T.....C.A.
6TBGa-2(9,1)        .............................T...........A.............---................................T.....C.A.
6TBGa-3(3,1)        ................T............T...........A.............---................................T.....C.A.
6TBGa-4(2,1)        ................T............T...........A.............---................................T.....C.A.
6TBGa-5(1,1)        ................T............T...........A.............---................................T.....C.A.
6TBGb-1(7,B)        ................T.........CC.....................T..............................................C.A.
6TBGb-2(6,1)        ..........................CC.....................T..............................................C.A.
6TBGb-3(2,1)        ................T.........CC.....................T..............................................C.A.
6TBGc-1(6,B)        ................T.........C.....................................................................C...
6TBGc-2(3,1)        ................T.........C.....................................................................C...
6TBGd(10,B)         ----------------------------------------------------------------------------------------------------
6TBGe(2,1)          ..........................CC.....................T..............................................C.A.

                            110       120       130       140       150       160       170       180       190       200
                    ....|....|....|....|....|....|....|....|....|....|....|....|....|....|....|....|....|....|....|....|
NTBGa-1(35,2)       CTCCTTCTCAGAGTCCTTCCTCTCTCTCCCTAAATTCTTCCCCCCTCCTCTTCTCCAGCACAGATGGCCTTCACATCGGGCTGCAACCACCCCAGTTTCA
NTBGa-2(8,1)        ....................................................................................................
NTBGa-3(6,B)        ....................................................................................................
NTBGa-4(5,B)        ....................................................................................................
NTBGa-6(2,1)        ....................................................................................................
NTBGa-7(1,1)        ....................................................................................................
NTBGa-8(1,1)        ....................................................................................................
NTBGa-10(1,1)       ....................................................................................................
NTBGa-11(1,1)       ....................................................................................................
NTBGa-12(1,1)       ....................................................................................................
NTBGa-13(1,1)       ....................................................................................................
NTBGa-15(1,1)       ....................................................................................................
NTBGa-16(1,B)       ....................................................................................................
NTBGb(4,1)          ....................................................................................................
NTBGd(1,1)          ..........A..........................................................................C..C...........
NTBGc(2,1)          ....................................................................................................
NTBGe(1,1)          ....................................................................................................
P2aTBGa-1(10,2)     ...................................................................................................G
P2aTBGa-2(3,1)      ...................................................................................................G
P2aTBGa-3(1,1)      ...................................................................................................G
P2aTBGb-1(2,2)      ...T..............................................................CG.........A......................
P2aTBGb-2(2,1)      ...T..............................................................CG.........A......................
P2aTBGb-3(1,1)      ...T..............................................................CG.........A......................
P2aTBGb-4(1,1)      ...T..............................................................CG.........A......................
P2aTBGc-1(1,B)      ...T................................................................................................
P2aTBGc-2(1,1)      ...T................................................................................................
P2aTBGc-3(1,1)      ...T................................................................................................
P2aTBGc-4(1,1)      ...T................................................................................................
P2aTBGc-6(1,1)      ...T................................................................................................
P2aTBGc-5(1,B)      ...T................................................................................................
15iTBGa-1(6,B)      .........................................T..........................................................
15iTBGa-2(4,2)      .........................................T..........................................................
15iTBGa-3(3,2)      .........................................T..........................................................
15iTBGa-4(3,B)      .........................................T..........................................................
15iTBGa-5(2,B)      ....................................................................................................
15iTBGa-6(2,1)      .........................................T..........................................................
15iTBGa-7(1,B)      .........................................T..........................................................
15iTBGa-8(1,1)      .........................................T..........................................................
15iTBGa-9(1,1)      ...........TC.........C.............................................................................
15iTBGb-1(4,2)      ...................................................................................................G
15iTBGb-2(2,2)      ...................................................................................................G
15iTBGb-4(1,1)      ...................................................................................................G
15iTBGb-5(1,B)      ...................................................................................................G
15iTBGb-7(1,1)      ...................................................................................................G
15iTBGc-1(3,1)      ....................................................................................................
6TBGa-1(10,1)       ...........TA.....................................................CA................................
6TBGa-2(9,1)        ...........TA.....................................................CA................................
6TBGa-3(3,1)        ...........TA.....................................................CA................................
6TBGa-4(2,1)        ...........TA.....................................................CA................................
6TBGa-5(1,1)        ...........TA.....................................................CA................................
6TBGb-1(7,B)        .........................A........................................CG................................
6TBGb-2(6,1)        .........................A........................................CG................................
6TBGb-3(2,1)        .........................A........................................CG................................
6TBGc-1(6,B)        ...................................................................................................G
6TBGc-2(3,1)        ...................................................................................................G
6TBGd(10,B)         ----------------------------------------------------------------------------------------------------
6TBGe(2,1)          .........................A........................................CG................................
```

```
                           210       220       230       240       250       260       270       280       290       300
                    ....|....|....|....|....|....|....|....|....|....|....|....|....|....|....|....|....|....|....|....|
NTBGa-1(35,2)       CCCTCCCCTGGAGGACCCTCCTGCCTTATCTCGTGGCTCTGCACCTCCTCCAGCCGGGATCAGCCCAGATCACGGTGGTGGCACCGAGCCTCCGTGTCAC
NTBGa-2(8,1)        ....................................................................................................
NTBGa-3(6,B)        ....................................................................................................
NTBGa-4(5,B)        ....................................................................................................
NTBGa-6(2,1)        ....................................................................................................
NTBGa-7(1,1)        ....................................................................................................
NTBGa-8(1,1)        ....................................................................................................
NTBGa-10(1,1)       ....................................................................................................
NTBGa-11(1,1)       ....................................................................................................
NTBGa-12(1,1)       ....................................................................................................
NTBGa-13(1,1)       ....................................................................................................
NTBGa-15(1,1)       ....................................................................................................
NTBGa-16(1,B)       .............................................C......................................................
NTBGb(4,1)          ........................................................................T...........................
NTBGd(1,1)          .................................G.................................A.......G........................
NTBGc(2,1)          .........................................T..........................................................
NTBGe(1,1)          ....................................................................................................
P2aTBGa-1(10,2)     .....................................................................G..............................
P2aTBGa-2(3,1)      .....................................................................G..............................
P2aTBGa-3(1,1)      .....................................................................G..............................
P2aTBGb-1(2,2)      ....................................................................C..............................G
P2aTBGb-2(2,1)      ....................................................................C..............................G
P2aTBGb-3(1,1)      ....................................................................C..............................G
P2aTBGb-4(1,1)      ....................................................................C..............................G
P2aTBGc-1(1,B)      ....................................................................C..............................G
P2aTBGc-2(1,1)      ....................................................................C..............................G
P2aTBGc-3(1,1)      ....................................................................C..............................G
P2aTBGc-4(1,1)      ....................................................................C..............................G
P2aTBGc-6(1,1)      ....................................................................C..............................G
P2aTBGc-5(1,B)      ....................................................................C..............................G
15iTBGa-1(6,B)      ....................................................................CAA.G...........................
15iTBGa-2(4,2)      ....................................................................CAA.G...........................
15iTBGa-3(3,2)      ....................................................................CAA.G...........................
15iTBGa-4(3,B)      ....................................................................CAA.G...........................
15iTBGa-5(2,B)      ....................................................................CAA.G...........................
15iTBGa-6(2,1)      ....................................................................CAA.G...........................
15iTBGa-7(1,B)      ....................................................................CAA.G...........................
15iTBGa-8(1,1)      ....................................................................CAA.G...........................
15iTBGa-9(1,1)      ....................................................................CAA.G...........................
15iTBGb-1(4,2)      ................................C...................C...............C..............................G
15iTBGb-2(2,2)      ................................C...................C...............C..............................G
15iTBGb-4(1,1)      ................................C...................C...............C..............................G
15iTBGb-5(1,B)      ................................C...................C...............C..............................G
15iTBGb-7(1,1)      ................................C...................C...............C..............................G
15iTBGc-1(3,1)      ....................................................C...............C...............................
6TBGa-1(10,1)       ......................................................T...C.........C...............................
6TBGa-2(9,1)        ......................................................T...C.........C...............................
6TBGa-3(3,1)        ......................................................T...C.........C...............................
6TBGa-4(2,1)        ......................................................T...C.........C...............................
6TBGa-5(1,1)        ......................................................T...C.........C...............................
6TBGb-1(7,B)        ..................................................G...T..............G..............................
6TBGb-2(6,1)        ..................................................G...T..............G..............................
6TBGb-3(2,1)        ..................................................G...T..............G..............................
6TBGc-1(6,B)        ............................................T.......................C...............................
6TBGc-2(3,1)        ............................................T.......................C...............................
6TBGd(10,B)         -----------------.................................G...T..............G..............................
6TBGe(2,1)          ..................................................G...T..............G..............................

                           310       320       330       340       350       360       370       380       390       400
                    ....|....|....|....|....|....|....|....|....|....|....|....|....|....|....|....|....|....|....|....|
NTBGa-1(35,2)       TGCCATCGTGGGACAGGATGTTGTGCTGCGCTGCCACTTGTCCCCATGCAAGGATGTTCGGAATTCAGACATCAGATGGATCCAGCAGCGGTCCTCTCGG
NTBGa-2(8,1)        ....................................................................................................
NTBGa-3(6,B)        ....................................................................................................
NTBGa-4(5,B)        ....................................................................................................
NTBGa-6(2,1)        ....................................................................................................
NTBGa-7(1,1)        ....................................................................................................
NTBGa-8(1,1)        ....................................................................................................
NTBGa-10(1,1)       ....................................................................................................
NTBGa-11(1,1)       ....................................................................................................
NTBGa-12(1,1)       ....................................................................................................
NTBGa-13(1,1)       ....................................................................................................
NTBGa-15(1,1)       ....................................................................................................
NTBGa-16(1,B)       ....................................................................................................
NTBGb(4,1)          ....................................................................................................
NTBGd(1,1)          ....................................................................................................
NTBGc(2,1)          ....................................................................................................
NTBGe(1,1)          ....................................................................................................
P2aTBGa-1(10,2)     .....A............C.................................................................................
P2aTBGa-2(3,1)      .....A............C.................................................................................
P2aTBGa-3(1,1)      .....A............C.................................................................................
P2aTBGb-1(2,2)      ......T...........C.................G........T...................................................G..
P2aTBGb-2(2,1)      ......T...........C.................G........T...................................................G..
P2aTBGb-3(1,1)      ......T...........C.................G........T...................................................G..
P2aTBGb-4(1,1)      ......T...........C.................G........T...................................................G..
P2aTBGc-1(1,B)      ......T...........C.................G........T...................................................G..
P2aTBGc-2(1,1)      ......T...........C.................G........T...................................................G..
P2aTBGc-3(1,1)      ......T...........C.................G........T...................................................G..
P2aTBGc-4(1,1)      ......T...........C.................G........T...................................................G..
P2aTBGc-6(1,1)      ......T...........C.................G........T...................................................G..
P2aTBGc-5(1,B)      ......T...........C.................G........T...................................................G..
15iTBGa-1(6,B)      .....A............................................T.................................................
15iTBGa-2(4,2)      .....A............................................T.................................................
15iTBGa-3(3,2)      .....A............................................T.................................................
15iTBGa-4(3,B)      .....A............................................T.................................................
15iTBGa-5(2,B)      .....A............................................T.................................................
15iTBGa-6(2,1)      .....A............................................T.................................................
15iTBGa-7(1,B)      .....A............................................T.................................................
15iTBGa-8(1,1)      .....A............................................T.................................................
15iTBGa-9(1,1)      .....A............................................T.................................................
15iTBGb-1(4,2)      ......T...........C..C.......................T...................................................G..
15iTBGb-2(2,2)      ......T...........C..C.......................T...................................................G..
15iTBGb-4(1,1)      ......T...........C..C.......................T...................................................G..
15iTBGb-5(1,B)      ......T...........C..C.......................T...................................................G..
15iTBGb-7(1,1)      ......T...........C..C.......................T...................................................G..
15iTBGc-1(3,1)      .....AT...........C.......................T..TC.........C........T.................T.............G..
6TBGa-1(10,1)       .....A...................................G...T..........C.T...GA.TG..............................G.T
6TBGa-2(9,1)        .....A...................................G...T..........C.T...GA.TG..............................G.T
6TBGa-3(3,1)        .....A...................................G...T..........C.T...GA.TG..............................G.T
6TBGa-4(2,1)        .....A...................................G...T..........C.T...GA.TG..............................G.T
6TBGa-5(1,1)        .....A......................T............G...T..........C.T...GA.TG..............................G.T
6TBGb-1(7,B)        .....................C.A.....................T........................................T..........A..
6TBGb-2(6,1)        .....................C.A.....................T........................................T..........A..
6TBGb-3(2,1)        .....................C.A.....................T........................................T..........A..
6TBGc-1(6,B)        .....AT...........C.................................................................................
6TBGc-2(3,1)        .....AT...........C.................................................................................
6TBGd(10,B)         .....A.......................................T........................................T..........G..
6TBGe(2,1)          .....................C.A.....................T........................................T..........A..
```

```
                           410       420       430       440       450       460       470       480       490       500
                    ....|....|....|....|....|....|....|....|....|....|....|....|....|....|....|....|....|....|....|....|
NTBGa-1(35,2)       CTTGTGCACCACTACCGAAATGGAGTGGACCTGGGGCAGATGGAGGAATATAAAGGGAGAACAGAACTGCTCAGGGATGGTCTCTCTGATGGAAACCTGG
NTBGa-2(8,1)        ....................................................................................................
NTBGa-3(6,B)        ....................................................................................................
NTBGa-4(5,B)        ....................................................................................................
NTBGa-6(2,1)        ....................................................................................................
NTBGa-7(1,1)        ....................................................................................................
NTBGa-8(1,1)        ....................................................................................................
NTBGa-10(1,1)       ....................................................................................................
NTBGa-11(1,1)       ....................................................................................................
NTBGa-12(1,1)       ....................................................................................................
NTBGa-13(1,1)       ....................................................................................................
NTBGa-15(1,1)       ....................................................................................................
NTBGa-16(1,B)       ....................................................................................................
NTBGb(4,1)          ....................................................................................................
NTBGd(1,1)          ....................................................................................................
NTBGc(2,1)          ....................................................................................................
NTBGe(1,1)          .....................................................................................A..............
P2aTBGa-1(10,2)     ....................................................................................................
P2aTBGa-2(3,1)      ....................................................................................................
P2aTBGa-3(1,1)      ....................................................................................................
P2aTBGb-1(2,2)      A...............A.T........................................G........................................
P2aTBGb-2(2,1)      A...............A.T........................................G........................................
P2aTBGb-3(1,1)      A...............A.T........................................G........................................
P2aTBGb-4(1,1)      A...............A.T........................................G........................................
P2aTBGc-1(1,B)      A...............A.G........................................G........................................
P2aTBGc-2(1,1)      A...............A.G........................................G........................................
P2aTBGc-3(1,1)      A...............A.G........................................G........................................
P2aTBGc-4(1,1)      A.............T.A.G........................................G........................................
P2aTBGc-6(1,1)      A...............A.G........................................G........................................
P2aTBGc-5(1,B)      A...............A.G........................................G........................................
15iTBGa-1(6,B)      ....................................................................................................
15iTBGa-2(4,2)      ....................................................................................................
15iTBGa-3(3,2)      ....................................................................................................
15iTBGa-4(3,B)      ....................................................................................................
15iTBGa-5(2,B)      ....................................................................................................
15iTBGa-6(2,1)      ....................................................................................................
15iTBGa-7(1,B)      ....................................................................................................
15iTBGa-8(1,1)      ....................................................................................................
15iTBGa-9(1,1)      ....................................................................................................
15iTBGb-1(4,2)      A...............A.G........................................G.................C......................
15iTBGb-2(2,2)      A...............A.G........................................G.................C......................
15iTBGb-4(1,1)      A...............A.G........................................G.................C......................
15iTBGb-5(1,B)      A...............A.G........................................G.................C......................
15iTBGb-7(1,1)      A...............A.G........................................G.................C......................
15iTBGc-1(3,1)      A........GC.......................A................G................A...............................
6TBGa-1(10,1)       T...............A.G......A........A............................................................T....
6TBGa-2(9,1)        T...............A.G......A........A............................................................T....
6TBGa-3(3,1)        T...............A.G......A........A............................................................T....
6TBGa-4(2,1)        T...............A.G......A........A............................................................T....
6TBGa-5(1,1)        T...............A.G......A........A............................................................T....
6TBGb-1(7,B)        A...............A.......T.........AT.......................G........................................
6TBGb-2(6,1)        A...............A.......T.........AT.......................G........................................
6TBGb-3(2,1)        A...............A.......T.........AT.......................G........................................
6TBGc-1(6,B)        ....................................................................................................
6TBGc-2(3,1)        ....................................................................................................
6TBGd(10,B)         A...............A.......T.........AT...............G.......G........................................
6TBGe(2,1)          A...............A.......T.........AT.......................G.........T..............................

                           510       520       530       540       550       560       570       580       590       600
                    ....|....|....|....|....|....|....|....|....|....|....|....|....|....|....|....|....|....|....|....|
NTBGa-1(35,2)       ATTTGCGCATCACTGCTGTGACCTCCTCTGATAGTGGCTCCTACAGCTGTGCTGTGCAAGATGGTGATGCCTATGCAGAAGCTGTGGTGAACCTGGAGGT
NTBGa-2(8,1)        ....................................................................................................
NTBGa-3(6,B)        ....................................................................................................
NTBGa-4(5,B)        ....................................................................................................
NTBGa-6(2,1)        ....................................................................................................
NTBGa-7(1,1)        ....................................................................................................
NTBGa-8(1,1)        ....................................................................................................
NTBGa-10(1,1)       ....................................................................................................
NTBGa-11(1,1)       ....................................................................................................
NTBGa-12(1,1)       ....................................................................................................
NTBGa-13(1,1)       ....................................................................................................
NTBGa-15(1,1)       ....................................................................................................
NTBGa-16(1,B)       ....................................................................................................
NTBGb(4,1)          ....................................................................................................
NTBGd(1,1)          ....................................................................................................
NTBGc(2,1)          .........................................C..........................................................
NTBGe(1,1)          .....................G.A....C...........A.................G..........G.........C.........G..........
P2aTBGa-1(10,2)     ................C..............C....................................................................
P2aTBGa-2(3,1)      ................C..............C....................................................................
P2aTBGa-3(1,1)      ................C..............C....................................................................
P2aTBGb-1(2,2)      ................C.......................A..T.T.....G....G..A...C....................................
P2aTBGb-2(2,1)      ................C.......................A..T.T.....G....G..A...C....................................
P2aTBGb-3(1,1)      ................C.......................A..T.T.....G....G..A...C....................................
P2aTBGb-4(1,1)      ................C.......................A..T.T.....G....G..A...C....................................
P2aTBGc-1(1,B)      .....................G.A................A..T.T.....G....G..A...C....................................
P2aTBGc-2(1,1)      .....................G.A................A..T.T.....G....G..A...C....................................
P2aTBGc-3(1,1)      .....................G.A................A..T.T.....G....G..A...C....................................
P2aTBGc-4(1,1)      .....................G.A................A..T.T.....G....G..A...C....................................
P2aTBGc-6(1,1)      .....................G.A................A..T.T.....G....G..A...C....................................
P2aTBGc-5(1,B)      .....................G.A................A..T.T.....G....G..A...C....................................
15iTBGa-1(6,B)      ...........................................................A.........G..............................
15iTBGa-2(4,2)      ...........................................................A.........G..............................
15iTBGa-3(3,2)      ...........................................................A.........G..............................
15iTBGa-4(3,B)      ...........................................................A.........G..............................
15iTBGa-5(2,B)      ...........................................................A.........G..............................
15iTBGa-6(2,1)      ...........................................................A.........G..............................
15iTBGa-7(1,B)      ...........................................................A.........G..............................
15iTBGa-8(1,1)      ...........................................................A.........G..............................
15iTBGa-9(1,1)      ...........................................................A.........G..............................
15iTBGb-1(4,2)      ................C.......................A..T.T.....G....A..A........................................
15iTBGb-2(2,2)      ................C.......................A..T.T.....G....A..A........................................
15iTBGb-4(1,1)      ................C.......................A..T.T.....G....A..A........................................
15iTBGb-5(1,B)      ................C.......................A..T.T.....G....A..A........................................
15iTBGb-7(1,1)      ................C.......................A..T.T.....G....A..A........................................
15iTBGc-1(3,1)      ......TT...........................................T......G....................C.....T...G.G........
6TBGa-1(10,1)       .....................G..................A....................C......................................
6TBGa-2(9,1)        .....................G..................A....................C......................................
6TBGa-3(3,1)        .....................G..................A....................C......................................
6TBGa-4(2,1)        .....................G..................A....................C......................................
6TBGa-5(1,1)        .....................G..................A....................C......................................
6TBGb-1(7,B)        ............T........G.........C.......TG..........T...........AC....G..............................
6TBGb-2(6,1)        ............T........G.........C.......TG..........T...........AC....G..............................
6TBGb-3(2,1)        ............T........G.........C.......TG..........T...........AC....G..............................
6TBGc-1(6,B)        ................C...................................................................................
6TBGc-2(3,1)        ................C...................................................................................
6TBGd(10,B)         ............T........G.........C.......TG..........T...........AC....G..............................
6TBGe(2,1)          ............T........G.........C.......TG..........T...........AC....G..............................
```

```
                            610       620       630       640       650       660       670       680       690       700
                    ....|....|....|....|....|....|....|....|....|....|....|....|....|....|....|....|....|....|....|....|
NTBGa-1(35,2)       GTCAGACCCCTTTTCTATGATCATCCTTTACTGGACAGTGGCTCTGGCTGTGATCATCACACTTCTGGTTGGGTCATTTGTCGTCAATGTTTTT-CTCCA
NTBGa-2(8,1)        ..............................................................................................T.....
NTBGa-3(6,B)        ..............................................................................................-.....
NTBGa-4(5,B)        ..............................................................................................-.....
NTBGa-6(2,1)        ..............................................................................................-.....
NTBGa-7(1,1)        ..............................................................................................-.....
NTBGa-8(1,1)        ..............................................................................................-.....
NTBGa-10(1,1)       ..............................................................................................-.....
NTBGa-11(1,1)       ..............................................................................................-.....
NTBGa-12(1,1)       ..............................................................................................T.....
NTBGa-13(1,1)       ..............................................................................................-.....
NTBGa-15(1,1)       ..............................................................................................-.....
NTBGa-16(1,B)       ..............................................................................................-.....
NTBGb(4,1)          ..............................................................................................-.....
NTBGd(1,1)          ..............................................................................................-.....
NTBGc(2,1)          ..............................................................................................-.....
NTBGe(1,1)          ......T........CCA....G...A.CC.....AG...............G..G.....A....C...............A...........-...TG
P2aTBGa-1(10,2)     ..............................................................................................-.....
P2aTBGa-2(3,1)      ..............................................................................................-.....
P2aTBGa-3(1,1)      ..............................................................................................-.....
P2aTBGb-1(2,2)      ..................................................................................A...T..C....-.....
P2aTBGb-2(2,1)      ..................................................................................A...T..C....-.....
P2aTBGb-3(1,1)      ..................................................................................A...T..C....-.....
P2aTBGb-4(1,1)      ..................................................................................A...T..C....-.....
P2aTBGc-1(1,B)      ......T.......................................................................................-.....
P2aTBGc-2(1,1)      ......T.......................................................................................-.....
P2aTBGc-3(1,1)      ......T.......................................................................................-.....
P2aTBGc-4(1,1)      ......T.......................................................................................-.....
P2aTBGc-6(1,1)      ......T.......................................................................................-.....
P2aTBGc-5(1,B)      ......T.......................................................................................-.....
15iTBGa-1(6,B)      ....------------------------------------------------------------------------------------------------
15iTBGa-2(4,2)      ....------------------------------------------------------------------------------------------------
15iTBGa-3(3,2)      ....------------------------------------------------------------------------------------------------
15iTBGa-4(3,B)      ....------------------------------------------------------------------------------------------------
15iTBGa-5(2,B)      ....------------------------------------------------------------------------------------------------
15iTBGa-6(2,1)      ..............................................................................................-.....
15iTBGa-7(1,B)      ....------------------------------------------------------------------------------------------------
15iTBGa-8(1,1)      ....------------------------------------------------------------------------------------------------
15iTBGa-9(1,1)      ....------------------------------------------------------------------------------------------------
15iTBGb-1(4,2)      ....------------------------------------------------------------------------------------------------
15iTBGb-2(2,2)      ....------------------------------------------------------------------------------------------------
15iTBGb-4(1,1)      ....------------------------------------------------------------------------------------------------
15iTBGb-5(1,B)      ....------------------------------------------------------------------------------------------------
15iTBGb-7(1,1)      ....------------------------------------------------------------------------------------------------
15iTBGc-1(3,1)      ....------------------------------------------------------------------------------------------------
6TBGa-1(10,1)       ......T........CCAA...G...A.CC.....AG.................AG..........................A...T..C....-...T.
6TBGa-2(9,1)        ......T........CCAA...G...A.CC.....AG.................AG..........................A...T..C....-...T.
6TBGa-3(3,1)        ......T........CCAA...G...A.CC.....AG.................AG..........................A...T..C....-...T.
6TBGa-4(2,1)        ......T........CCAA...G...A.CC.....AG.................AG..........................A...T..C....-...T.
6TBGa-5(1,1)        ......T........CCAA...G...A.CC.....AG.................AG..........................A...T..C....-...T.
6TBGb-1(7,B)        ......T........CCA....G...A.CC.....AG...............G..G.....A...............C....A...T.......-...TG
6TBGb-2(6,1)        ......T........CCA....G...A.CC.....AG...............G..G.....A...............C....A...T.......-...TG
6TBGb-3(2,1)        ......T........CCA....G...A.CC.....AG...............G..G.....A...............C....A...T.......-...TG
6TBGc-1(6,B)        ..............................................................................................-.....
6TBGc-2(3,1)        ..............................................................................................-.....
6TBGd(10,B)         ......T.T......CCA....G...A.CC.....AG...............------------------------------------------------
6TBGe(2,1)          ......T........CCA....G...A.CC.....AG...............G..G.....A...............C....A...T.......-...TG

                            710       720       730       740       750       760       770       780       790       800
                    ....|....|....|....|....|....|....|....|....|....|....|....|....|....|....|....|....|....|....|....|
NTBGa-1(35,2)       TAGAAAGAAAGTGGCACAGAGCAGAGAGCTGA--------------------------------------------------------------------
NTBGa-2(8,1)        ................................--------------------------------------------------------------------
NTBGa-3(6,B)        ................................--------------------------------------------------------------------
NTBGa-4(5,B)        ................................--------------------------------------------------------------------
NTBGa-6(2,1)        ................................--------------------------------------------------------------------
NTBGa-7(1,1)        ................................--------------------------------------------------------------------
NTBGa-8(1,1)        ................................--------------------------------------------------------------------
NTBGa-10(1,1)       ................................--------------------------------------------------------------------
NTBGa-11(1,1)       ................................--------------------------------------------------------------------
NTBGa-12(1,1)       ................................--------------------------------------------------------------------
NTBGa-13(1,1)       ................................--------------------------------------------------------------------
NTBGa-15(1,1)       ................................GTGAGTCCTTCCATCCCCATCCACCACCAAAGTCCCTTTAATGGAACTGACAGCAGACTGCAGAGTGC
NTBGa-16(1,B)       ................................--------------------------------------------------------------------
NTBGb(4,1)          ................................--------------------------------------------------------------------
NTBGd(1,1)          ................................--------------------------------------------------------------------
NTBGc(2,1)          ................................--------------------------------------------------------------------
NTBGe(1,1)          ...G.......C....................--------------------------------------------------------------------
P2aTBGa-1(10,2)     ................................--------------------------------------------------------------------
P2aTBGa-2(3,1)      ................................--------------------------------------------------------------------
P2aTBGa-3(1,1)      ................................--------------------------------------------------------------------
P2aTBGb-1(2,2)      ...........CT..T.......C........--------------------------------------------------------------------
P2aTBGb-2(2,1)      ...........CT..T.......C........--------------------------------------------------------------------
P2aTBGb-3(1,1)      ...........CT..T.......C........--------------------------------------------------------------------
P2aTBGb-4(1,1)      ...........CT..T.......C........--------------------------------------------------------------------
P2aTBGc-1(1,B)      ................................--------------------------------------------------------------------
P2aTBGc-2(1,1)      ................................--------------------------------------------------------------------
P2aTBGc-3(1,1)      ................................--------------------------------------------------------------------
P2aTBGc-4(1,1)      ...........-----------------------------------------------------------------------------------------
P2aTBGc-6(1,1)      ................................--------------------------------------------------------------------
P2aTBGc-5(1,B)      ................................--------------------------------------------------------------------
15iTBGa-1(6,B)      ----------------------------------------------------------------------------------------------------
15iTBGa-2(4,2)      ----------------------------------------------------------------------------------------------------
15iTBGa-3(3,2)      ----------------------------------------------------------------------------------------------------
15iTBGa-4(3,B)      ----------------------------------------------------------------------------------------------------
15iTBGa-5(2,B)      ----------------------------------------------------------------------------------------------------
15iTBGa-6(2,1)      ................................GTGAGTCCTTCCATCCCCATCCACCACCAAAGTCCCTTTAATGGAACTGACAGCAGGCTGCAGAGTGC
15iTBGa-7(1,B)      ----------------------------------------------------------------------------------------------------
15iTBGa-8(1,1)      ----------------------------------------------------------------------------------------------------
15iTBGa-9(1,1)      ----------------------------------------------------------------------------------------------------
15iTBGb-1(4,2)      ----------------------------------------------------------------------------------------------------
15iTBGb-2(2,2)      ----------------------------------------------------------------------------------------------------
15iTBGb-4(1,1)      ----------------------------------------------------------------------------------------------------
15iTBGb-5(1,B)      ----------------------------------------------------------------------------------------------------
15iTBGb-7(1,1)      ----------------------------------------------------------------------------------------------------
15iTBGc-1(3,1)      ----------------------------------------------------------------------------------------------------
6TBGa-1(10,1)       ...........C....................GTGAGTCCTTCCAGCTCCTTCCACCACCAAAGTCCCTTTAATGGAACTGATAGAAGACTGCAGAGTGC
6TBGa-2(9,1)        ...........C....................GTGAGTCCTTCCAGCTCCTTCCACCACCAAAGTCCCTTTAATGGAACTGATAGAAGACTGCAGAGTGC
6TBGa-3(3,1)        ...........C....................GTGAGTCCTTCCAGCTCCTTCCACCACCAAAGTCCCTTTAATGGAACTGATAGAAGACTGCAGAGTGC
6TBGa-4(2,1)        ...........C....................GTGAGTCCTTCCAGC-------ACCACCAAAGTCCCTTTAATGGAACTGATAGAAGACTTCAGAGTGC
6TBGa-5(1,1)        ...........C....................GTGAGTCCTTCCAGCTCCTTCCACCACCAAAGTCCCTTTAATGGAACTGATAGAAGACTGCAGAGTGC
6TBGb-1(7,B)        ...G..........T.................--------------------------------------------------------------------
6TBGb-2(6,1)        ...G.......-----------------------------------------------------------------------------------------
6TBGb-3(2,1)        ...G..........T.................GTGAGTCCTTCCAGTCCCTTCCACCACCAAAGCCTCTTTAATGGAAATGATAGAAGACTGCAGAGTGT
6TBGc-1(6,B)        ................................--------------------------------------------------------------------
6TBGc-2(3,1)        ................................GTGAGTCCTTCCATCCCCATCCACCACCAAAGTCCCTTTAATGGAACTGACAGCAGACTGCAGAGTGC
6TBGd(10,B)         ----------------------------------------------------------------------------------------------------
6TBGe(2,1)          ...G..........T...............A.--------------------------------------------------------------------
```

```
                           810       820       830       840       850       860       870       880       890       900
                    ....|....|....|....|....|....|....|....|....|....|....|....|....|....|....|....|....|....|....|....|
NTBGa-1(35,2)       ----------------------------------------------------------------------------------------------------
NTBGa-2(8,1)        ----------------------------------------------------------------------------------------------------
NTBGa-3(6,B)        ----------------------------------------------------------------------------------------------------
NTBGa-4(5,B)        ----------------------------------------------------------------------------------------------------
NTBGa-6(2,1)        ----------------------------------------------------------------------------------------------------
NTBGa-7(1,1)        ----------------------------------------------------------------------------------------------------
NTBGa-8(1,1)        ----------------------------------------------------------------------------------------------------
NTBGa-10(1,1)       ----------------------------------------------------------------------------------------------------
NTBGa-11(1,1)       ----------------------------------------------------------------------------------------------------
NTBGa-12(1,1)       ----------------------------------------------------------------------------------------------------
NTBGa-13(1,1)       ----------------------------------------------------------------------------------------------------
NTBGa-15(1,1)       TGGGTTATGCCATGTGCTGGGGCCATGAGCTATGTTGAGGCTTTGGAATGTGTTGGGGTTGTGGGATGTACTGGGGTCGTGGGATGTGTTATTCCTGGCT
NTBGa-16(1,B)       ----------------------------------------------------------------------------------------------------
NTBGb(4,1)          ----------------------------------------------------------------------------------------------------
NTBGd(1,1)          ----------------------------------------------------------------------------------------------------
NTBGc(2,1)          ----------------------------------------------------------------------------------------------------
NTBGe(1,1)          ----------------------------------------------------------------------------------------------------
P2aTBGa-1(10,2)     ----------------------------------------------------------------------------------------------------
P2aTBGa-2(3,1)      ----------------------------------------------------------------------------------------------------
P2aTBGa-3(1,1)      ----------------------------------------------------------------------------------------------------
P2aTBGb-1(2,2)      ----------------------------------------------------------------------------------------------------
P2aTBGb-2(2,1)      ----------------------------------------------------------------------------------------------------
P2aTBGb-3(1,1)      ----------------------------------------------------------------------------------------------------
P2aTBGb-4(1,1)      ----------------------------------------------------------------------------------------------------
P2aTBGc-1(1,B)      ----------------------------------------------------------------------------------------------------
P2aTBGc-2(1,1)      ----------------------------------------------------------------------------------------------------
P2aTBGc-3(1,1)      ----------------------------------------------------------------------------------------------------
P2aTBGc-4(1,1)      ----------------------------------------------------------------------------------------------------
P2aTBGc-6(1,1)      ----------------------------------------------------------------------------------------------------
P2aTBGc-5(1,B)      ----------------------------------------------------------------------------------------------------
15iTBGa-1(6,B)      ----------------------------------------------------------------------------------------------------
15iTBGa-2(4,2)      ----------------------------------------------------------------------------------------------------
15iTBGa-3(3,2)      ----------------------------------------------------------------------------------------------------
15iTBGa-4(3,B)      ----------------------------------------------------------------------------------------------------
15iTBGa-5(2,B)      ----------------------------------------------------------------------------------------------------
15iTBGa-6(2,1)      TGGGTTATGCCATGTGCTGGGGCCATGAGCTATGTTGAGGCTTTGGAATGTGTTGGGGTTGTGGGATGTACTGGGGTCGTGGGATGTGTCAATCCTGGCT
15iTBGa-7(1,B)      ----------------------------------------------------------------------------------------------------
15iTBGa-8(1,1)      ----------------------------------------------------------------------------------------------------
15iTBGa-9(1,1)      ----------------------------------------------------------------------------------------------------
15iTBGb-1(4,2)      ----------------------------------------------------------------------------------------------------
15iTBGb-2(2,2)      ----------------------------------------------------------------------------------------------------
15iTBGb-4(1,1)      ----------------------------------------------------------------------------------------------------
15iTBGb-5(1,B)      ----------------------------------------------------------------------------------------------------
15iTBGb-7(1,1)      ----------------------------------------------------------------------------------------------------
15iTBGc-1(3,1)      ----------------------------------------------------------------------------------------------------
6TBGa-1(10,1)       TGGGTTTATGCCTTGTGCAGGGGCCATGGGATCTAT-GGGACCTTGGGATGTGTTGGGGCCGTGGGATGTGCTGGGGTCGTGGGATCT------------
6TBGa-2(9,1)        TGGGTTTATGCCTTGTGCAGGGGCCATGGGATCTAT-GGGACCTTGGGATGTGTTGGGGCCGTGGGATGTGCTGGGGTCGTGGGATCT------------
6TBGa-3(3,1)        TGGGTTTATGCCTTGTGCAGGGGCCATGGGATCTAT-GGGACCTTGGGATGTGTTGGGGCCGTGGGATGTGCTGGGGTCGTGGGATCT------------
6TBGa-4(2,1)        TGGGTTTATGCCTTGTGCAGGGGCCATGGGATCTAT-GGGACCTTGGGATGTGTTGGGGCCGTGGGATGTGCTGGGGTNNNNNNNNNN------------
6TBGa-5(1,1)        TGGGTTTATGCCTTGTGCAGGGGCCATGGGATCTAT-GGGACCTTGGGATGTGTTGGGGCCGTGGGATGTGCTGGGGTCGTGGGATCT------------
6TBGb-1(7,B)        ----------------------------------------------------------------------------------------------------
6TBGb-2(6,1)        ----------------------------------------------------------------------------------------------------
6TBGb-3(2,1)        TGGGTTTATGCCATGTGTTAGGTTCATGGGATGTCTTGGGGCCTTGAAATTAAGTGGGTTCATGGGATGTACCAGAGTTGTCGGGATTTGTTATTGTTAT
6TBGc-1(6,B)        ----------------------------------------------------------------------------------------------------
6TBGc-2(3,1)        TGGGTT-ATGCCATGTGCTGGGGCCATGAGCTATGTTGAGGCTTTGGAATGTGTTGGGGTTGTGGGATGTACTGGGGTCGTGGGATGT------------
6TBGd(10,B)         ----------------------------------------------------------------------------------------------------
6TBGe(2,1)          ----------------------------------------------------------------------------------------------------

                           910       920       930       940       950       960       970       980       990      1000
                    ....|....|....|....|....|....|....|....|....|....|....|....|....|....|....|....|....|....|....|....|
NTBGa-1(35,2)       ----------------------------------------------------------------------------------------------------
NTBGa-2(8,1)        ----------------------------------------------------------------------------------------------------
NTBGa-3(6,B)        ----------------------------------------------------------------------------------------------------
NTBGa-4(5,B)        ----------------------------------------------------------------------------------------------------
NTBGa-6(2,1)        ----------------------------------------------------------------------------------------------------
NTBGa-7(1,1)        ----------------------------------------------------------------------------------------------------
NTBGa-8(1,1)        ----------------------------------------------------------------------------------------------------
NTBGa-10(1,1)       ----------------------------------------------------------------------------------------------------
NTBGa-11(1,1)       ----------------------------------------------------------------------------------------------------
NTBGa-12(1,1)       ----------------------------------------------------------------------------------------------------
NTBGa-13(1,1)       ----------------------------------------------------------------------------------------------------
NTBGa-15(1,1)       GATTCACGTGGAAAAACCTTT----------------------CACAATCGGTTCCTTCCAGTTTGTTTAATTCCTTCTTGGGCCCAAAGTGGTCATTGG
NTBGa-16(1,B)       ----------------------------------------------------------------------------------------------------
NTBGb(4,1)          ----------------------------------------------------------------------------------------------------
NTBGd(1,1)          ----------------------------------------------------------------------------------------------------
NTBGc(2,1)          ----------------------------------------------------------------------------------------------------
NTBGe(1,1)          ----------------------------------------------------------------------------------------------------
P2aTBGa-1(10,2)     ----------------------------------------------------------------------------------------------------
P2aTBGa-2(3,1)      ----------------------------------------------------------------------------------------------------
P2aTBGa-3(1,1)      ----------------------------------------------------------------------------------------------------
P2aTBGb-1(2,2)      ----------------------------------------------------------------------------------------------------
P2aTBGb-2(2,1)      ----------------------------------------------------------------------------------------------------
P2aTBGb-3(1,1)      ----------------------------------------------------------------------------------------------------
P2aTBGb-4(1,1)      ----------------------------------------------------------------------------------------------------
P2aTBGc-1(1,B)      ----------------------------------------------------------------------------------------------------
P2aTBGc-2(1,1)      ----------------------------------------------------------------------------------------------------
P2aTBGc-3(1,1)      ----------------------------------------------------------------------------------------------------
P2aTBGc-4(1,1)      ----------------------------------------------------------------------------------------------------
P2aTBGc-6(1,1)      ----------------------------------------------------------------------------------------------------
P2aTBGc-5(1,B)      ----------------------------------------------------------------------------------------------------
15iTBGa-1(6,B)      -----------------------GACCCCTT---------------------TTCTATGATCATCCTTTACTGGACAGTGGCTCTGGCTGTGATCATCAC
15iTBGa-2(4,2)      -----------------------GACCCCTT---------------------TTCTATGATCATCCTTTACTGGACAGTGGCTCTGGCTGTGATCATCAC
15iTBGa-3(3,2)      -----------------------GACCCCTT---------------------TTCTATGATCATCCTTTACTGGACAGTGGCTCTGGCTGTGATCATCAC
15iTBGa-4(3,B)      -----------------------GACCCCTT---------------------TTCTATGATCATCCTTTACTGGACAGTGGCTCTGGCTGTGATCATCAC
15iTBGa-5(2,B)      -----------------------GACCCCTT---------------------TTCTATGATCATCCTTTACTGGACAGTGGCTCTGGCTGTGATCATCAC
15iTBGa-6(2,1)      GATTCACGTGGAAAAACCTTTCACAATCGGT---------------------TCCTTCCAGTTTGTTTAATTCCTTCTTGGGTCCAAA-GTGGTCATTGG
15iTBGa-7(1,B)      -----------------------GACCCCTT---------------------TTCTATGATCATCCTTTACTGGACAGTGGCTCTGGCTGTGATCATCAC
15iTBGa-8(1,1)      -----------------------GACCCCTT---------------------TTCTATGATCATCCTTTACTGGACAGTGGCTCTGGCTGTGATCATCAC
15iTBGa-9(1,1)      -----------------------GACCCCTT---------------------TTCTATGATCATCCTTTACTGGACAGTGGCTCTGGCTGTGATCATCAC
15iTBGb-1(4,2)      -----------------------GACCCCTT---------------------TTCTATGATCATCCTTTACTGGACAGTGGCTCTGGCTGTGATCATCAC
15iTBGb-2(2,2)      -----------------------GACCCCTT---------------------TTCTATGATCATCCTTTACTGGACAGTGGCTCTGGCTGTGATCATCAC
15iTBGb-4(1,1)      -----------------------GACCCCTT---------------------TTCTATGATCATCCTTTACTGGACAGTGGCTCTGGCTGTGATCATCAC
15iTBGb-5(1,B)      -----------------------GACCCCTT---------------------TTCTATGATCATCCTTTACTGGACAGTGGCTCTGGCTGTGATCATCAC
15iTBGb-7(1,1)      -----------------------GACCCCTT---------------------TTCTATGATCATCCTTTACTGGACAGTGGCTCTGGCTGTGATCATCAC
15iTBGc-1(3,1)      -----------------------GACCCCAA---------------------TTCTATGATCATCCTTTACTGGACAGTGGCTCTGGCTGTGATCGTCAC
6TBGa-1(10,1)       ---------GTCAATCCTGATTGATCCTC-TTCAGAACTCTTGCCCAATCGGTTCCTTCCGATTCTTTTAACTCCTTCTTGGGACCAAAGTGGTCATTGG
6TBGa-2(9,1)        ---------GTCAATCCTGATTGATCCTC-TTCAGAACTCTTGCCCAATCGGTTCCTTCCGATTCTTTTAACTCCTTCTTGGGACCAAAGTGGTCATTGG
6TBGa-3(3,1)        ---------GTCAATCCTGATTGATCCTC-TTCAGAACTCTTGCCCAATCGGTTCCTTCCGATTCTTTTAACTCCTTCTTGGGACCAAAGTGGTCATTGG
6TBGa-4(2,1)        ---------NNNNNNNNNNNTT--------TGCAGAGCA--------------------------------------------------------ATTAG
6TBGa-5(1,1)        ---------GTCAATCCTGATTGATCCTC-TTCAGAACTCTTGCCCAATCGGTTCCTTCCGATTCTTTTAACTCCTTCTTGGGACCAAAGTGGTCATTGG
6TBGb-1(7,B)        ----------------------------------------------------------------------------------------------------
6TBGb-2(6,1)        ----------------------------------------------------------------------------------------------------
6TBGb-3(2,1)        TGTGGGATTGTCGATCCAGACTGCTGCATATGAAAAACTTATCCCCAGTCGTTCCCTTCCCATTGTTTTAATTCCTTCTTGGGACCAAATTGGTCATTGG
6TBGc-1(6,B)        ----------------------------------------------------------------------------------------------------
6TBGc-2(3,1)        ---------GTCAATCCTGGCTGATTCACGTGGAAAAACCTTTCACAATCGGTTCCTTCCAGTTTGTTTAATTCCTTCTTGG-CCCAAAGTGGTCATTGG
6TBGd(10,B)         ----------------------------------------------------------------------------------------------------
6TBGe(2,1)          ----------------------------------------------------------------------------------------------------
```

```
                          1010      1020      1030      1040      1050      1060      1070      1080      1090      1100
                    ....|....|....|....|....|....|....|....|....|....|....|....|....|....|....|....|....|....|....|....|
NTBGa-1(35,2)       ----------------------------------------------------------------------------------------------------
NTBGa-2(8,1)        ----------------------------------------------------------------------------------------------------
NTBGa-3(6,B)        ----------------------------------------------------------------------------------------------------
NTBGa-4(5,B)        ----------------------------------------------------------------------------------------------------
NTBGa-6(2,1)        ----------------------------------------------------------------------------------------------------
NTBGa-7(1,1)        ----------------------------------------------------------------------------------------------------
NTBGa-8(1,1)        ----------------------------------------------------------------------------------------------------
NTBGa-10(1,1)       ----------------------------------------------------------------------------------------------------
NTBGa-11(1,1)       ----------------------------------------------------------------------------------------------------
NTBGa-12(1,1)       ----------------------------------------------------------------------------------------------------
NTBGa-13(1,1)       ----------------------------------------------------------------------------------------------------
NTBGa-15(1,1)       ACTCCTCCCAGAAAAAAGGGTTTGGGGTCAGGGTGTGAGAGCTGATGGCACGGAAACGTGTCCCCTCTGACCATGCATTTCATTTGCTTCTATTTTGCAG
NTBGa-16(1,B)       ----------------------------------------------------------------------------------------------------
NTBGb(4,1)          ----------------------------------------------------------------------------------------------------
NTBGd(1,1)          ----------------------------------------------------------------------------------------------------
NTBGc(2,1)          ----------------------------------------------------------------------------------------------------
NTBGe(1,1)          ----------------------------------------------------------------------------------------------------
P2aTBGa-1(10,2)     ----------------------------------------------------------------------------------------------------
P2aTBGa-2(3,1)      ----------------------------------------------------------------------------------------------------
P2aTBGa-3(1,1)      ----------------------------------------------------------------------------------------------------
P2aTBGb-1(2,2)      ----------------------------------------------------------------------------------------------------
P2aTBGb-2(2,1)      ----------------------------------------------------------------------------------------------------
P2aTBGb-3(1,1)      ----------------------------------------------------------------------------------------------------
P2aTBGb-4(1,1)      ----------------------------------------------------------------------------------------------------
P2aTBGc-1(1,B)      ----------------------------------------------------------------------------------------------------
P2aTBGc-2(1,1)      ----------------------------------------------------------------------------------------------------
P2aTBGc-3(1,1)      ----------------------------------------------------------------------------------------------------
P2aTBGc-4(1,1)      ----------------------------------------------------------------------------------------------------
P2aTBGc-6(1,1)      ----------------------------------------------------------------------------------------------------
P2aTBGc-5(1,B)      ----------------------------------------------------------------------------------------------------
15iTBGa-1(6,B)      ACTTCTGGTTGGGTCA----TTTGTCGTCAATGTTT------TT-CTCCATAGAAAGAAAGTGGCACAGAGCAGAGAGCTGA------------------
15iTBGa-2(4,2)      ACTTCTGGTTGGGTCA----TTTGTCGTCAATGTTT------TT-CTCCATAGAAAGAAAGTGGCACAGAGCAGAGAGCTGA------------------
15iTBGa-3(3,2)      ACTTCTGGTTGGGTCA----TTTGTCGTCAATGTTT------TT-CTCCATAGAAAGAAAGTGGCACAGAGCAGAGAGCTGA------------------
15iTBGa-4(3,B)      ACTTCTGGTTGGGTCA----TTTGTCGTCAATGTTT------TTTCTCCATAGAAAGAAAGTGGCACAGAGCAGAGAGCTGA------------------
15iTBGa-5(2,B)      ACTTCTGGTTGGGTCA----TTTGTCGTCAATGTTT------TT-CTCCATAGAAAGAAAGTGGCACAGAGCAGAGAGCTGA------------------
15iTBGa-6(2,1)      ACTCCTCCCAGAAAAAAGGGTTTGGGGTCAGGGTGTGAGAGCTGATGGCATGGAAATGTGTCCCCTCTGACCATGCACTTCATTTGCTTCTATTTTGCAG
15iTBGa-7(1,B)      ACTTCTGGTTGGGTCA----TTTGTCGTCAATGTTT------TT-CTCCATAGAAAGAAAGTGGCACAGAGCAGAGAGCTGA------------------
15iTBGa-8(1,1)      ACTTCTGGTTGGGTCA----TTTGTCGTCAATGTTT------TT-CTCCATAGAAAGAAAGTGGCACAGAGCAGAGAGCTGA------------------
15iTBGa-9(1,1)      ACTTCTGGTTGGGTCA----TTTGTCGTCAATGTTT------TT-CTCCATAGAAAGAAAGTGGCACAGAGCAGAGAGCTGA------------------
15iTBGb-1(4,2)      ACTTCTGGTTGGGTCA----TTTGTCGTCAATGTTT-------TTCTCCATAGAAAGAAAGCGGCACAGAGCAGAGAGCTGA------------------
15iTBGb-2(2,2)      ACTTCTGGTTGGGTCA----TTTGTCGTCAATGTTT-------TTCTCCATAGAAAGAAAGCGGCACAGAGCAGAGAGCTGA------------------
15iTBGb-4(1,1)      ACTTCTGGTTGGGTCA----TTTGTCGTCAATGTTT-------TTCTCCATAGAAAGAAAG---------------------------------------
15iTBGb-5(1,B)      ACTTCTGGTTGGGTCA----TTTGTCGTCAATGTTT-------TTCTCCATAGAAAGAAAGCGGCACAGAGCAGAGAGCTGA------------------
15iTBGb-7(1,1)      ACTTCTGGTTGGGTCA----TTTGTCGTCAATGTTT-------TTCTCCATAGAAAGAAAGCGGCACAGAGCAGAGAGCTGA------------------
15iTBGc-1(3,1)      ACTTCTGGTTGGGTCA----TTTGTCATCATTGCTT-------TTCTCCATAGAAAGAAAGCTGCACATAGCAGAGAGCTGA------------------
6TBGa-1(10,1)       CCTC-----------------------------------------------------------------------------------------TTAATAG
6TBGa-2(9,1)        CCTC-----------------------------------------------------------------------------------------TTAATAG
6TBGa-3(3,1)        CCTC-----------------------------------------------------------------------------------------TTAATAG
6TBGa-4(2,1)        CTTC-----------------------------------------------------------------------------------------AAAACTG
6TBGa-5(1,1)        CCTC-----------------------------------------------------------------------------------------TTAATAG
6TBGb-1(7,B)        ----------------------------------------------------------------------------------------------------
6TBGb-2(6,1)        ----------------------------------------------------------------------------------------------------
6TBGb-3(2,1)        CCACTTCCC-----------------------------------------------------------------------------------------AG
6TBGc-1(6,B)        ----------------------------------------------------------------------------------------------------
6TBGc-2(3,1)        ACTCCTCCCAGAAAAAAGGGTTTGGGGTCAGGGTGTGAGAGCTGATGGCATGGAAACGTGTCCCCTCTGACCATGCATTTCATTTGCTTCTATTTTGCAG
6TBGd(10,B)         ----------------------------------------------------------------------------------------------------
6TBGe(2,1)          ----------------------------------------------------------------------------------------------------

                          1110      1120      1130      1140      1150      1160      1170      1180      1190      1200
                    ....|....|....|....|....|....|....|....|....|....|....|....|....|....|....|....|....|....|....|....|
NTBGa-1(35,2)       AGAGAAAAGATGCAGAGTTGG-------------------------------------------------------------------------------
NTBGa-2(8,1)        .....................-------------------------------------------------------------------------------
NTBGa-3(6,B)        .....................-------------------------------------------------------------------------------
NTBGa-4(5,B)        .....................-------------------------------------------------------------------------------
NTBGa-6(2,1)        .....................-------------------------------------------------------------------------------
NTBGa-7(1,1)        .....................-------------------------------------------------------------------------------
NTBGa-8(1,1)        .....................-------------------------------------------------------------------------------
NTBGa-10(1,1)       ....................T-------------------------------------------------------------------------------
NTBGa-11(1,1)       .....................-------------------------------------------------------------------------------
NTBGa-12(1,1)       .....................-------------------------------------------------------------------------------
NTBGa-13(1,1)       .....................-------------------------------------------------------------------------------
NTBGa-15(1,1)       .....................GTAAGTCTCCTTCCCTAAAGCGAGGGAATTCAGGGTGTCCCCATGGCATCAGCCGTGGAAATTAGTAGCTGTCCTCTCT
NTBGa-16(1,B)       .....................-------------------------------------------------------------------------------
NTBGb(4,1)          .....................-------------------------------------------------------------------------------
NTBGd(1,1)          .....................-------------------------------------------------------------------------------
NTBGc(2,1)          .....................-------------------------------------------------------------------------------
NTBGe(1,1)          ----------------------------------------------------------------------------------------------------
P2aTBGa-1(10,2)     .....................-------------------------------------------------------------------------------
P2aTBGa-2(3,1)      .....................-------------------------------------------------------------------------------
P2aTBGa-3(1,1)      .....................-------------------------------------------------------------------------------
P2aTBGb-1(2,2)      .....................-------------------------------------------------------------------------------
P2aTBGb-2(2,1)      .....................-------------------------------------------------------------------------------
P2aTBGb-3(1,1)      .....................-------------------------------------------------------------------------------
P2aTBGb-4(1,1)      .....................-------------------------------------------------------------------------------
P2aTBGc-1(1,B)      ..G............CAC..--------------------------------------------------------------------------------
P2aTBGc-2(1,1)      ..G............CAC..--------------------------------------------------------------------------------
P2aTBGc-3(1,1)      ..G............CAC..--------------------------------------------------------------------------------
P2aTBGc-4(1,1)      ..G............CAC..--------------------------------------------------------------------------------
P2aTBGc-6(1,1)      ..G............CAC..--------------------------------------------------------------------------------
P2aTBGc-5(1,B)      ..G............CAC..--------------------------------------------------------------------------------
15iTBGa-1(6,B)      ...................---------------------------------------------------------------------------------
15iTBGa-2(4,2)      ...................---------------------------------------------------------------------------------
15iTBGa-3(3,2)      ...................---------------------------------------------------------------------------------
15iTBGa-4(3,B)      ...................---------------------------------------------------------------------------------
15iTBGa-5(2,B)      ...................---------------------------------------------------------------------------------
15iTBGa-6(2,1)      ...................---------------------------------------------------------------------------------
15iTBGa-7(1,B)      ...................---------------------------------------------------------------------------------
15iTBGa-8(1,1)      ...................---------------------------------------------------------------------------------
15iTBGa-9(1,1)      ...................---------------------------------------------------------------------------------
15iTBGb-1(4,2)      ----------------------------------------------------------------------------------------------------
15iTBGb-2(2,2)      ----------------------------------------------------------------------------------------------------
15iTBGb-4(1,1)      ----------------------------------------------------------------------------------------------------
15iTBGb-5(1,B)      ----------------------------------------------------------------------------------------------------
15iTBGb-7(1,1)      ----------------------------------------------------------------------------------------------------
15iTBGc-1(3,1)      ----------------------------------------------------------------------------------------------------
6TBGa-1(10,1)       .A.........TTG....CT.G---GTATGGGAGCA-------------------------------------GCCATGGGATGAGAAGGTGTTCCCTCT
6TBGa-2(9,1)        .A.....-...TTG....CT.G---GTATGGGAGCA-------------------------------------GCCATGGGATGAGAAGGTGTTCCCTCT
6TBGa-3(3,1)        .A.........TTG....CT.G---GTATGGGAGCA-------------------------------------GCCATGGGATGAGAAGGTGTTCCCTCT
6TBGa-4(2,1)        .AC.......CTGT..A-------------------------------------------------------------GAGATGCGTGAGTCTCCCCTC-
6TBGa-5(1,1)        .A.........TTG....CT.G---GTATGGGAGCA-------------------------------------GCCATGGGATGAGAAGGTGTTCCCTCT
6TBGb-1(7,B)        ----------------------------------------------------------------------------------------------------
6TBGb-2(6,1)        ----------------------------------------------------------------------------------------------------
6TBGb-3(2,1)        .ACA.......TTG.G..CTAGGTA---------------------------------------------------------------------------
6TBGc-1(6,B)        .....................-------------------------------------------------------------------------------
6TBGc-2(3,1)        .....................-------------------------------------------------------------------------------
6TBGd(10,B)         ----------------------------------------------------------------------------------------------------
6TBGe(2,1)          ----------------------------------------------------------------------------------------------------
```

```
                          1210      1220      1230      1240      1250      1260      1270      1280      1290      1300
                    ....|....|....|....|....|....|....|....|....|....|....|....|....|....|....|....|....|....|....|....|
NTBGa-1(35,2)       --------------------------------TGGAGAAAGCTGCAGCATTGG-----------------------------------------------
NTBGa-2(8,1)        --------------------------------.....................-----------------------------------------------
NTBGa-3(6,B)        --------------------------------.....................-----------------------------------------------
NTBGa-4(5,B)        --------------------------------.....................-----------------------------------------------
NTBGa-6(2,1)        --------------------------------.....................-----------------------------------------------
NTBGa-7(1,1)        --------------------------------.....................-----------------------------------------------
NTBGa-8(1,1)        --------------------------------.....................-----------------------------------------------
NTBGa-10(1,1)       --------------------------------.....................-----------------------------------------------
NTBGa-11(1,1)       --------------------------------.....................-----------------------------------------------
NTBGa-12(1,1)       --------------------------------.....................-----------------------------------------------
NTBGa-13(1,1)       --------------------------------.....................-----------------------------------------------
NTBGa-15(1,1)       GACAATTCACTGCTCTGCTCTTTCCTTTCCAG.....................GTGAGTTATATTCCCCAAGCCAAAGTACTTTGGGTCTTCCCATTGGA
NTBGa-16(1,B)       --------------------------------.....................-----------------------------------------------
NTBGb(4,1)          --------------------------------.....................-----------------------------------------------
NTBGd(1,1)          --------------------------------.....................-----------------------------------------------
NTBGc(2,1)          --------------------------------.....................-----------------------------------------------
NTBGe(1,1)          ----------------------------------------------------------------------------------------------------
P2aTBGa-1(10,2)     --------------------------------.....................-----------------------------------------------
P2aTBGa-2(3,1)      --------------------------------.....................-----------------------------------------------
P2aTBGa-3(1,1)      --------------------------------.....................-----------------------------------------------
P2aTBGb-1(2,2)      --------------------------------....CC...............-----------------------------------------------
P2aTBGb-2(2,1)      --------------------------------....CC...............-----------------------------------------------
P2aTBGb-3(1,1)      --------------------------------....CC...............-----------------------------------------------
P2aTBGb-4(1,1)      --------------------------------....CC...............-----------------------------------------------
P2aTBGc-1(1,B)      ----------------------------------------------------------------------------------------------------
P2aTBGc-2(1,1)      ----------------------------------------------------------------------------------------------------
P2aTBGc-3(1,1)      ----------------------------------------------------------------------------------------------------
P2aTBGc-4(1,1)      ----------------------------------------------------------------------------------------------------
P2aTBGc-6(1,1)      ----------------------------------------------------------------------------------------------------
P2aTBGc-5(1,B)      ----------------------------------------------------------------------------------------------------
15iTBGa-1(6,B)      ------------------------------GG.....................TGAGAAAAGATGCAGCACTGGCGGAGAAAGTTGCAGCAT--------
15iTBGa-2(4,2)      ------------------------------GG.....................TGAGAAAAGATGCAGCACTGGCGGAGAAAGTTGCAGCAT--------
15iTBGa-3(3,2)      ------------------------------GG.....................TGAGAAAAGATGCAGCACTGGCGGAGAAAGTTGCAGCAT--------
15iTBGa-4(3,B)      ------------------------------GG.....................TGAGAAAAGATGCAGCACTGGCGGAGAAAGTTGCAGCAT--------
15iTBGa-5(2,B)      ------------------------------GG.....................TGAGAAAAGATGCAGCACTGGCGGAGAAAGTTGCAGCAT--------
15iTBGa-6(2,1)      ------------------------------GG.....................TGAGAAAAGATGCAGCACTGGCGGAGAAAGTTGCAGCAT--------
15iTBGa-7(1,B)      ------------------------------GG.....................TGAGAAAAGATGCAGCACTGGCGGAGAAAGTTGCAGCAT--------
15iTBGa-8(1,1)      ------------------------------GG.....................TGAGAAAAGATGCAGCACTGGCGGAGAAAGTTGCAGCAT--------
15iTBGa-9(1,1)      ------------------------------GG.....................TGAGAAAAGATGCAGCACTGGCGGAGAAAGTTGCAGCAT--------
15iTBGb-1(4,2)      ----------------------------------------------------------------------------------------------------
15iTBGb-2(2,2)      ----------------------------------------------------------------------------------------------------
15iTBGb-4(1,1)      ----------------------------------------------------------------------------------------------------
15iTBGb-5(1,B)      ----------------------------------------------------------------------------------------------------
15iTBGb-7(1,1)      ----------------------------------------------------------------------------------------------------
15iTBGc-1(3,1)      ----------------------------------------------------------------------------------------------------
6TBGa-1(10,1)       GACCATGCACTGCTCTGCTCTTTCCTTTCCAG....CC...............AGAGAAAAGAT------------------------------------
6TBGa-2(9,1)        GACCATGCACTGCTCTGCTCTTTCCTTTCCAG....CC...............-----------------------------------------------
6TBGa-3(3,1)        GACCATGCACTGCTCTGCTCTTTCCTTTCCAG....CC...............GTGAGTTATATTCCCCAAGCCAAAGTACTTTGGGTCTTCCCATTGGA
6TBGa-4(2,1)        ------------------------------------CC..AA.AA.A......-----------------------------------------------
6TBGa-5(1,1)        GACCATGCACTGCTCTGCTCTTTCCTTTCCAG....CC...............-----------------------------------------------
6TBGb-1(7,B)        ----------------------------------------------------------------------------------------------------
6TBGb-2(6,1)        ----------------------------------------------------------------------------------------------------
6TBGb-3(2,1)        --------------------------------..C.AGC...CAGG.G..GA.AAG--------------------------------------------
6TBGc-1(6,B)        --------------------------------.....................-----------------------------------------------
6TBGc-2(3,1)        --------------------------------.....................-----------------------------------------------
6TBGd(10,B)         ----------------------------------------------------------------------------------------------------
6TBGe(2,1)          ----------------------------------------------------------------------------------------------------

                          1310      1320      1330      1340      1350      1360      1370      1380      1390      1400
                    ....|....|....|....|....|....|....|....|....|....|....|....|....|....|....|....|....|....|....|....|
NTBGa-1(35,2)       ----------------------------------------------------------------------------------------------------
NTBGa-2(8,1)        ----------------------------------------------------------------------------------------------------
NTBGa-3(6,B)        ----------------------------------------------------------------------------------------------------
NTBGa-4(5,B)        ----------------------------------------------------------------------------------------------------
NTBGa-6(2,1)        ----------------------------------------------------------------------------------------------------
NTBGa-7(1,1)        ----------------------------------------------------------------------------------------------------
NTBGa-8(1,1)        ----------------------------------------------------------------------------------------------------
NTBGa-10(1,1)       ----------------------------------------------------------------------------------------------------
NTBGa-11(1,1)       ----------------------------------------------------------------------------------------------------
NTBGa-12(1,1)       ----------------------------------------------------------------------------------------------------
NTBGa-13(1,1)       ----------------------------------------------------------------------------------------------------
NTBGa-15(1,1)       AGTTATTTCCTCAGACCATCCTTTCTGTTGTGTTTGCTTTGGCATCATGTTAGTAAAATGCCTTTCTTGGGACCAAAGTGGTCATTGGCCACTTCCCAGA
NTBGa-16(1,B)       ----------------------------------------------------------------------------------------------------
NTBGb(4,1)          ----------------------------------------------------------------------------------------------------
NTBGd(1,1)          ----------------------------------------------------------------------------------------------------
NTBGc(2,1)          ----------------------------------------------------------------------------------------------------
NTBGe(1,1)          ----------------------------------------------------------------------------------------------------
P2aTBGa-1(10,2)     ----------------------------------------------------------------------------------------------------
P2aTBGa-2(3,1)      ----------------------------------------------------------------------------------------------------
P2aTBGa-3(1,1)      ----------------------------------------------------------------------------------------------------
P2aTBGb-1(2,2)      ----------------------------------------------------------------------------------------------------
P2aTBGb-2(2,1)      ----------------------------------------------------------------------------------------------------
P2aTBGb-3(1,1)      ----------------------------------------------------------------------------------------------------
P2aTBGb-4(1,1)      ----------------------------------------------------------------------------------------------------
P2aTBGc-1(1,B)      ----------------------------------------------------------------------------------------------------
P2aTBGc-2(1,1)      ----------------------------------------------------------------------------------------------------
P2aTBGc-3(1,1)      ----------------------------------------------------------------------------------------------------
P2aTBGc-4(1,1)      ----------------------------------------------------------------------------------------------------
P2aTBGc-6(1,1)      ----------------------------------------------------------------------------------------------------
P2aTBGc-5(1,B)      ----------------------------------------------------------------------------------------------------
15iTBGa-1(6,B)      ----------------------------------------------------------------------------------------------------
15iTBGa-2(4,2)      ----------------------------------------------------------------------------------------------------
15iTBGa-3(3,2)      ----------------------------------------------------------------------------------------------------
15iTBGa-4(3,B)      ----------------------------------------------------------------------------------------------------
15iTBGa-5(2,B)      ----------------------------------------------------------------------------------------------------
15iTBGa-6(2,1)      ----------------------------------------------------------------------------------------------------
15iTBGa-7(1,B)      ----------------------------------------------------------------------------------------------------
15iTBGa-8(1,1)      ----------------------------------------------------------------------------------------------------
15iTBGa-9(1,1)      ----------------------------------------------------------------------------------------------------
15iTBGb-1(4,2)      ----------------------------------------------------------------------------------------------------
15iTBGb-2(2,2)      ----------------------------------------------------------------------------------------------------
15iTBGb-4(1,1)      ----------------------------------------------------------------------------------------------------
15iTBGb-5(1,B)      ----------------------------------------------------------------------------------------------------
15iTBGb-7(1,1)      ----------------------------------------------------------------------------------------------------
15iTBGc-1(3,1)      ----------------------------------------------------------------------------------------------------
6TBGa-1(10,1)       ------------------------------------------------------------------------GCAGAGTTGGGTAAGTCTCCTTCCCTAA
6TBGa-2(9,1)        ----------------------------------------------------------------------------------------------------
6TBGa-3(3,1)        AGTTATTTCCTCAGATCATCCTTTCTATTGTGTTTGCTTTGGCTTTCATGTT-AGTAAAATGCCTTCTTGGGGCGAAAGTGGTCATTGGCCACTTCCCAG
6TBGa-4(2,1)        ----------------------------------------------------------------------------------------------------
6TBGa-5(1,1)        ----------------------------------------------------------------------------------------------------
6TBGb-1(7,B)        ----------------------------------------------------------------------------------------------------
6TBGb-2(6,1)        ----------------------------------------------------------------------------------------------------
6TBGb-3(2,1)        ----------------------------------------------------------------------------------------------------
6TBGc-1(6,B)        ----------------------------------------------------------------------------------------------------
6TBGc-2(3,1)        ----------------------------------------------------------------------------------------------------
6TBGd(10,B)         ----------------------------------------------------------------------------------------------------
6TBGe(2,1)          ----------------------------------------------------------------------------------------------------
```

```
                         1410      1420      1430      1440      1450      1460      1470      1480      1490      1500
                   ....|....|....|....|....|....|....|....|....|....|....|....|....|....|....|....|....|....|....|....|
NTBGa-1(35,2)      --------------------------------------------------------------------------------------------AGAGAAAA
NTBGa-2(8,1)       --------------------------------------------------------------------------------------------........
NTBGa-3(6,B)       --------------------------------------------------------------------------------------------........
NTBGa-4(5,B)       --------------------------------------------------------------------------------------------........
NTBGa-6(2,1)       --------------------------------------------------------------------------------------------........
NTBGa-7(1,1)       --------------------------------------------------------------------------------------------........
NTBGa-8(1,1)       --------------------------------------------------------------------------------------------........
NTBGa-10(1,1)      --------------------------------------------------------------------------------------------........
NTBGa-11(1,1)      --------------------------------------------------------------------------------------------........
NTBGa-12(1,1)      --------------------------------------------------------------------------------------------........
NTBGa-13(1,1)      --------------------------------------------------------------------------------------------........
NTBGa-15(1,1)      AAAAAAGGTTTGGGGTCAGGGTGTGGGAGCTGATGGCATGGAAACATGTTCCCTCTGACCATGCATTTCCTTTGCTTCT-TTTTCCA----G........
NTBGa-16(1,B)      --------------------------------------------------------------------------------------------........
NTBGb(4,1)         --------------------------------------------------------------------------------------------........
NTBGd(1,1)         --------------------------------------------------------------------------------------------........
NTBGc(2,1)         --------------------------------------------------------------------------------------------........
NTBGe(1,1)         --------------------------------------------------------------------------------------------........
P2aTBGa-1(10,2)    --------------------------------------------------------------------------------------------........
P2aTBGa-2(3,1)     --------------------------------------------------------------------------------------------........
P2aTBGa-3(1,1)     --------------------------------------------------------------------------------------------........
P2aTBGb-1(2,2)     --------------------------------------------------------------------------------------------........
P2aTBGb-2(2,1)     --------------------------------------------------------------------------------------------........
P2aTBGb-3(1,1)     --------------------------------------------------------------------------------------------........
P2aTBGb-4(1,1)     --------------------------------------------------------------------------------------------........
P2aTBGc-1(1,B)     ----------------------------------------------------------------------------------------------------
P2aTBGc-2(1,1)     ----------------------------------------------------------------------------------------------------
P2aTBGc-3(1,1)     ----------------------------------------------------------------------------------------------------
P2aTBGc-4(1,1)     ----------------------------------------------------------------------------------------------------
P2aTBGc-6(1,1)     ----------------------------------------------------------------------------------------------------
P2aTBGc-5(1,B)     ----------------------------------------------------------------------------------------------------
15iTBGa-1(6,B)     -----------------------------------------------------------------------------------------TGG........
15iTBGa-2(4,2)     -----------------------------------------------------------------------------------------TGG........
15iTBGa-3(3,2)     -----------------------------------------------------------------------------------------TGG........
15iTBGa-4(3,B)     -----------------------------------------------------------------------------------------TGG........
15iTBGa-5(2,B)     -----------------------------------------------------------------------------------------TGG........
15iTBGa-6(2,1)     -----------------------------------------------------------------------------------------TGG........
15iTBGa-7(1,B)     -----------------------------------------------------------------------------------------TGG........
15iTBGa-8(1,1)     -----------------------------------------------------------------------------------------TGG........
15iTBGa-9(1,1)     -----------------------------------------------------------------------------------------TGG........
15iTBGb-1(4,2)     --------------------------------------------------------------------------------------------........
15iTBGb-2(2,2)     --------------------------------------------------------------------------------------------........
15iTBGb-4(1,1)     --------------------------------------------------------------------------------------------........
15iTBGb-5(1,B)     --------------------------------------------------------------------------------------------........
15iTBGb-7(1,1)     --------------------------------------------------------------------------------------------........
15iTBGc-1(3,1)     --------------------------------------------------------------------------------------------........
6TBGa-1(10,1)      AGCGAGGGAATTCAGGGTCTCCCCATGGCATCAGCTGTGGGATGAGCAGCTGTCCTCTCTGACCATGCACTGCTCTGCTCTTTCTTTT------------
6TBGa-2(9,1)       --------------------------------------------------------------------------------------------........
6TBGa-3(3,1)       AACAAAAAGGTTTGGGGTCAGGGTGTAAGAGCTG--ATGACATGGAAATGTGTCCCCTCTGACCATGCATTTCCTTTGCTCCTTTTTTGCAG........
6TBGa-4(2,1)       ----------------------------------------------------------------------------------------------------
6TBGa-5(1,1)       --------------------------------------------------------------------------------------------........
6TBGb-1(7,B)       --------------------------------------------------------------------------------------------..G.....
6TBGb-2(6,1)       --------------------------------------------------------------------------------------------..G.....
6TBGb-3(2,1)       -------------------------------------------------TTGTTCCCTCTGACCATGCATTTCCATTGCTTTTATTTTGCAG..G.....
6TBGc-1(6,B)       --------------------------------------------------------------------------------------------........
6TBGc-2(3,1)       --------------------------------------------------------------------------------------------........
6TBGd(10,B)        ----------------------------------------------------------------------------------------------------
6TBGe(2,1)         --------------------------------------------------------------------------------------------..G.....

                         1510      1520      1530      1540      1550      1560      1570      1580      1590      1600
                   ....|....|....|....|....|....|....|....|....|....|....|....|....|....|....|....|....|....|....|....|
NTBGa-1(35,2)      GATGCAGAGTTGGCGGA-ACAAGCAGCGCAATCGA-----------------------------------------------------------------
NTBGa-2(8,1)       .................-.................-----------------------------------------------------------------
NTBGa-3(6,B)       .................-.................-----------------------------------------------------------------
NTBGa-4(5,B)       .................-.................-----------------------------------------------------------------
NTBGa-6(2,1)       .................-.................-----------------------------------------------------------------
NTBGa-7(1,1)       .................-.................-----------------------------------------------------------------
NTBGa-8(1,1)       .............T...-.................-----------------------------------------------------------------
NTBGa-10(1,1)      .................-.................-----------------------------------------------------------------
NTBGa-11(1,1)      .................-.................-----------------------------------------------------------------
NTBGa-12(1,1)      .................-.................-----------------------------------------------------------------
NTBGa-13(1,1)      .................-.................-----------------------------------------------------------------
NTBGa-15(1,1)      .................-.................-----------------------------------------------------------------
NTBGa-16(1,B)      .................-.................-----------------------------------------------------------------
NTBGb(4,1)         .................-.................-----------------------------------------------------------------
NTBGd(1,1)         ..............CTCCGGT...T.T.GG..GAGATG------------------------------TTCCTCTCATCATACACTGACTCTGCTTTTCC
NTBGc(2,1)         .................-.................-----------------------------------------------------------------
NTBGe(1,1)         .......C.........-..T..AT.A.AT....GGT---------------------------------------------------------------
P2aTBGa-1(10,2)    .................----------------------------------------------------------------------ACAAGCAGCGCTA
P2aTBGa-2(3,1)     .................----------------------------------------------------------------------ACAAGCAGCGCTA
P2aTBGa-3(1,1)     .................----------------------------------------------------------------------ACAAGCAGCGCTA
P2aTBGb-1(2,2)     .................----------------------------------------------------------------------ACAAGCAGCGCTA
P2aTBGb-2(2,1)     .................----------------------------------------------------------------------ACAAGCAGCGCTA
P2aTBGb-3(1,1)     .................----------------------------------------------------------------------ACAAGCAGCGCTA
P2aTBGb-4(1,1)     .................----------------------------------------------------------------------ACAAGCAGCGCTA
P2aTBGc-1(1,B)     ------------.....----------------------------------------------------------------------ACTACCTGCGATA
P2aTBGc-2(1,1)     ------------.....----------------------------------------------------------------------ACTACCTGCGATA
P2aTBGc-3(1,1)     ------------.....----------------------------------------------------------------------ACTACCTGCGATA
P2aTBGc-4(1,1)     ------------.....----------------------------------------------------------------------ACTACCTGCGATA
P2aTBGc-6(1,1)     ------------.....----------------------------------------------------------------------ACTACCTGCGATA
P2aTBGc-5(1,B)     ------------.....----------------------------------------------------------------------ACTACCTGCGATA
15iTBGa-1(6,B)     ......AT.....------TGGAG.AA..TGCA.CATTGGAGAGAAAAGATGAAGAGTTGGCGGA----------------------ACAAGCAGCGCTA
15iTBGa-2(4,2)     ......AT.....------TGGAG.AA..TGCA.CATTGGAGAGAAAAGATGAAGAGTTGGCGGA----------------------ACAAGCAGCGCTA
15iTBGa-3(3,2)     ......AT.....------TGGAG.AA..TGCA.CATTGGAGAGAAAAGATGAAGAGTTGGCGGA----------------------ACAAGCAGCGCTA
15iTBGa-4(3,B)     ......AT.....------TGGAG.AA..TGCA.CATTGGAGAGAAAAGATGAAGAGTTGGCGGA----------------------ACAAGCAGCGCTA
15iTBGa-5(2,B)     ......AT.....------TGGAG.AA..TGCA.CATTGGAGAGAAAAGATGAAGAGTTGGCGGA----------------------ACAAGCAGCGCTA
15iTBGa-6(2,1)     ......AT.....------TGGAG.AA..TGCA.CATTGGAGAGAAAAGATGAAGAGTTGGCGGA----------------------ACAAGCAGCGCTA
15iTBGa-7(1,B)     ......AT.....------TGGAG.AA..TGCA.CATTGGAGAGAAAAGATGAAGAGTTGGCGGA----------------------ACAAGCAGCGCTA
15iTBGa-8(1,1)     ......AT.....------TGGAG.AA..TGCA.CATTGGAGAGAAAAGATGAAGAGTTGGCGGA----------------------ACAAGCAGCGCTA
15iTBGa-9(1,1)     ......AT.....------TGGAG.AA..TGCA.CATTGGAGAGAAAAGATGAAGAGTTGGCGGA----------------------ACAAGCAGCGCTA
15iTBGb-1(4,2)     ......AT.....GT-------------------------------------------------------------------------------------
15iTBGb-2(2,2)     ......AT.....GT-------------------------------------------------------------------------------------
15iTBGb-4(1,1)     ......AT.....GT-------------------------------------------------------------------------------------
15iTBGb-5(1,B)     ......AT.....GT-------------------------------------------------------------------------------------
15iTBGb-7(1,1)     ......AT.....GT-------------------------------------------------------------------------------------
15iTBGc-1(3,1)     .......C.....GTTCCT---------------------------------------------------------------------------------
6TBGa-1(10,1)      ---------CCA.------------------------------------------------TGGA----------------------ACTAGATGAGATA
6TBGa-2(9,1)       .............------------------------------------------------TGGA----------------------ACTAGATGAGATA
6TBGa-3(3,1)       .............------------------------------------------------TGGA----------------------ACTAGATGAGATA
6TBGa-4(2,1)       -----G.TCC.CA------------------------------------------------TG--------------------------TGGATGAGATG
6TBGa-5(1,1)       .............------------------------------------------------TGGA----------------------ACTAGATGAGATA
6TBGb-1(7,B)       .......CAC...------------------------------------------------CGGA----------------------ACTACCTGCGATA
6TBGb-2(6,1)       .......CAC...------------------------------------------------CGGA----------------------ACTACCTGCGATA
6TBGb-3(2,1)       .......CAC...------------------------------------------------CGGA----------------------ACTACCTGCGATA
6TBGc-1(6,B)       .............------------------------------------------------CGGA----------------------ACAAGCAGCGCTA
6TBGc-2(3,1)       .............------------------------------------------------CGGA----------------------ACAAGCAGCGCTA
6TBGd(10,B)        ----------------------------------------------------------------------------------------------------
6TBGe(2,1)         .......CAC...------------------------------------------------CGGA----------------------ACTACCTGCGATA
```

```
                          1610      1620      1630      1640      1650      1660      1670      1680      1690      1700
                    ....|....|....|....|....|....|....|....|....|....|....|....|....|....|....|....|....|....|....|....|
NTBGa-1(35,2)       -------AGCAAAGAGATGCAATGTTGG------------------------------------------------------------------------
NTBGa-2(8,1)        -------.....................------------------------------------------------------------------------
NTBGa-3(6,B)        -------.....................------------------------------------------------------------------------
NTBGa-4(5,B)        -------.....................------------------------------------------------------------------------
NTBGa-6(2,1)        -------.....................------------------------------------------------------------------------
NTBGa-7(1,1)        -------.....................------------------------------------------------------------------------
NTBGa-8(1,1)        -------.....................------------------------------------------------------------------------
NTBGa-10(1,1)       -------.....................------------------------------------------------------------------------
NTBGa-11(1,1)       -------.....................------------------------------------------------------------------------
NTBGa-12(1,1)       -------.....................------------------------------------------------------------------------
NTBGa-13(1,1)       -------.....................------------------------------------------------------------------------
NTBGa-15(1,1)       -------.....................------------------------------------------------------------------------
NTBGa-16(1,B)       -------.....................------------------------------------------------------------------------
NTBGb(4,1)          -------.....................------------------------------------------------------------------------
NTBGd(1,1)          TTTGCAG.....................------------------------------------------------------------------------
NTBGc(2,1)          -------.....................------------------------------------------------------------------------
NTBGe(1,1)          ---------TT...T.C..A..ATC..A------------------------------------------------------------------------
P2aTBGa-1(10,2)     TCGA---.....................AC----------------------------------------------------------------------
P2aTBGa-2(3,1)      TCGA---......T..............AC----------------------------------------------------------------------
P2aTBGa-3(1,1)      TCGA---.....................AC----------------------------------------------------------------------
P2aTBGb-1(2,2)      TCGA---..............G......AG----------------------------------------------------------------------
P2aTBGb-2(2,1)      TCGA---..............G......AG----------------------------------------------------------------------
P2aTBGb-3(1,1)      TCGA---..............G......AG----------------------------------------------------------------------
P2aTBGb-4(1,1)      TCGA---..............G......AG----------------------------------------------------------------------
P2aTBGc-1(1,B)      TTGG---GTGT.T.T.C.....ATC..A------------------------------------------------------------------------
P2aTBGc-2(1,1)      TTGG---GTGT.T.T.C.....ATC..A------------------------------------------------------------------------
P2aTBGc-3(1,1)      TTGG---GTGT.T.T.C.....ATC..A------------------------------------------------------------------------
P2aTBGc-4(1,1)      TTGG---GTGT.T.T.C.....ATC..A------------------------------------------------------------------------
P2aTBGc-6(1,1)      TTGG---GTGT.T.T.C.....ATC..A------------------------------------------------------------------------
P2aTBGc-5(1,B)      TTGG---GTGT.T.T.C.....ATC..A------------------------------------------------------------------------
15iTBGa-1(6,B)      TCGA---.....................------------------------------------------------------------------------
15iTBGa-2(4,2)      TCGA---.....................------------------------------------------------------------------------
15iTBGa-3(3,2)      TCGA---.....................------------------------------------------------------------------------
15iTBGa-4(3,B)      TCGA---.....................------------------------------------------------------------------------
15iTBGa-5(2,B)      TCGA---.....................------------------------------------------------------------------------
15iTBGa-6(2,1)      TCGA---.....................------------------------------------------------------------------------
15iTBGa-7(1,B)      TCGA---.....................------------------------------------------------------------------------
15iTBGa-8(1,1)      TCGA---.....................------------------------------------------------------------------------
15iTBGa-9(1,1)      TCGA---.....................------------------------------------------------------------------------
15iTBGb-1(4,2)      ---------TT...T.C.....ATC..A------------------------------------------------------------------------
15iTBGb-2(2,2)      ---------TT...T.C.....ATC..A------------------------------------------------------------------------
15iTBGb-4(1,1)      ---------TT...T.C.....ATC..A------------------------------------------------------------------------
15iTBGb-5(1,B)      ---------TT...T.C.....ATC..A------------------------------------------------------------------------
15iTBGb-7(1,1)      ---------TT...T.C.....ATC..A------------------------------------------------------------------------
15iTBGc-1(3,1)      -------------AT.C.....ATC..A------------------------------------------------------------------------
6TBGa-1(10,1)       TCGGGT---TT...T.C..A..GTC..A------------------------------------------------------------------------
6TBGa-2(9,1)        TCGGGT---TT...T.C..A..GTC..A------------------------------------------------------------------------
6TBGa-3(3,1)        TCGGGT---TT...T.C..A..GTC..AGTAAGTGCCCTTCCTGACACTGAAGGAATTTGGGGGTCTTCCCATGGGATCAGCCGGGGAATGAAAACATGT
6TBGa-4(2,1)        TTCCTC---TC.----------------------------------------------------------------------------------------
6TBGa-5(1,1)        TCGGGT---TT...T.C..A..GTC..A------------------------------------------------------------------------
6TBGb-1(7,B)        TTGGGTG---T.T.TAC.....ATC..A------------------------------------------------------------------------
6TBGb-2(6,1)        TTGGGTG---T.T.TAC.....ATC..A------------------------------------------------------------------------
6TBGb-3(2,1)        TTGGGTG---T.T.TAC.....ATC..A------------------------------------------------------------------------
6TBGc-1(6,B)        TCGA---.....................------------------------------------------------------------------------
6TBGc-2(3,1)        TCGA---.....................------------------------------------------------------------------------
6TBGd(10,B)         ----------------------------------------------------------------------------------------------------
6TBGe(2,1)          TTGGGTG---T.T.TAC.....ATC..A------------------------------------------------------------------------

                          1710      1720      1730      1740      1750      1760      1770      1780      1790      1800
                    ....|....|....|....|....|....|....|....|....|....|....|....|....|....|....|....|....|....|....|....|
NTBGa-1(35,2)       ----------------------------------------ACAAACACGTTCTAAAACTGGAGGAAA-------AGACAGACGAAG--------------
NTBGa-2(8,1)        ----------------------------------------...........................-------............--------------
NTBGa-3(6,B)        ----------------------------------------...........................-------............--------------
NTBGa-4(5,B)        ----------------------------------------...........................-------............--------------
NTBGa-6(2,1)        ----------------------------------------...........................-------............--------------
NTBGa-7(1,1)        ----------------------------------------...........................-------............--------------
NTBGa-8(1,1)        ----------------------------------------...........................-------............--------------
NTBGa-10(1,1)       ----------------------------------------................C..........-------............--------------
NTBGa-11(1,1)       ----------------------------------------...........................-------............--------------
NTBGa-12(1,1)       ----------------------------------------...........................-------............--------------
NTBGa-13(1,1)       ----------------------------------------...........................-------............--------------
NTBGa-15(1,1)       ----------------------------------------...........................-------............--------------
NTBGa-16(1,B)       ----------------------------------------...........................-------............--------------
NTBGb(4,1)          ----------------------------------------...........................-------............--------------
NTBGd(1,1)          ----------------------------------------......................A.G..A------............--------------
NTBGc(2,1)          ----------------------------------------...........................-------............--------------
NTBGe(1,1)          ----------------------------------------.GC..TTA.C.TC.......A.C....-------.TG.T.......--------------
P2aTBGa-1(10,2)     ----------------------------------------.A.C..--...................-------............--------------
P2aTBGa-2(3,1)      ----------------------------------------.A.C..--...................-------............--------------
P2aTBGa-3(1,1)      ----------------------------------------.A.C..--...................-------............--------------
P2aTBGb-1(2,2)      ----------------------------------------.A.C..--...................-------............--------------
P2aTBGb-2(2,1)      ----------------------------------------.A.C..--...................-------............--------------
P2aTBGb-3(1,1)      ----------------------------------------.A.C..--...................-------............--------------
P2aTBGb-4(1,1)      ----------------------------------------.A.C..--...................-------............--------------
P2aTBGc-1(1,B)      ----------------------------------------.G.TC.TA.C.TC.......AT.A..C-------.A.TG..AA..T--------------
P2aTBGc-2(1,1)      ----------------------------------------.G.TC.TA.C.TC.......AT.A..C-------.A.TG..AA..T--------------
P2aTBGc-3(1,1)      ----------------------------------------.G.TC.TA.C.TC.......AT.A..C-------.A.TG..AA..T--------------
P2aTBGc-4(1,1)      ----------------------------------------.G.TC.TA.C.TC.......AT.A..C-------.A.TG..AA..T--------------
P2aTBGc-6(1,1)      ----------------------------------------.G.TC.TA.C.TC.......AGT..GTGCTCCCTCCCA.ACTA..AGTAAAGGGGCTCTG
P2aTBGc-5(1,B)      ----------------------------------------.G.TC.TA.C.TC.......AT.A..C-------.A.TG..AA..T--------------
15iTBGa-1(6,B)      ----------------------------------------.G...................-A.G..A------............--------------
15iTBGa-2(4,2)      ----------------------------------------.G...................-A.G..A------............--------------
15iTBGa-3(3,2)      ----------------------------------------.G...................-A.G..A------............--------------
15iTBGa-4(3,B)      ----------------------------------------.G...................-A.G..A------............--------------
15iTBGa-5(2,B)      ----------------------------------------.G...................-A.G..A------............--------------
15iTBGa-6(2,1)      ----------------------------------------.G...................-A.G..A------............--------------
15iTBGa-7(1,B)      ----------------------------------------.G...................-A.G..A------............--------------
15iTBGa-8(1,1)      ----------------------------------------.G...................-A.G..A------......G.....--------------
15iTBGa-9(1,1)      ----------------------------------------.G...................-A.G..A------............--------------
15iTBGb-1(4,2)      ----------------------------------------.G...TTA.CCTC.......A-AC...A------.TG.T.......--------------
15iTBGb-2(2,2)      ----------------------------------------.G...TTA.CCTC.......A-AC...A------.TG.T.......--------------
15iTBGb-4(1,1)      ----------------------------------------.G...TTA.CCTC.......A-AC...A------.TG.T.......--------------
15iTBGb-5(1,B)      ----------------------------------------.G...TTA.CCTC.......A-AC...A------.TG.T.......--------------
15iTBGb-7(1,1)      ----------------------------------------.G...TTA.CCTC.......A-AC...A------.TG.T.......--------------
15iTBGc-1(3,1)      ----------------------------------------.G.CC.TA.CCTC.......A-AC.G.A------TTG.T.......--------------
6TBGa-1(10,1)       ----------------------------------------.GC..TTA.C.TC.......A-ACG..A------.TG.T.......--------------
6TBGa-2(9,1)        ----------------------------------------.GC..TTA.C.TC.......A-ACG..A------.TG.T.......--------------
6TBGa-3(3,1)        CCCCCTCTCTTCATGCATTTCCTATTTCTTACCTTTGCAG.GC..TTA.C.TC.......A-ACG..A------.TG.T.......--------------
6TBGa-4(2,1)        ------------------------------------------------------------------------------TC...C.C--------------
6TBGa-5(1,1)        ----------------------------------------.GC..TTA.C.TC.......A-ACG..A------.TG.T.......--------------
6TBGb-1(7,B)        ----------------------------------------.G.TC.TA.C.TC.......AT-....C------.A.TG..AA..T--------------
6TBGb-2(6,1)        ----------------------------------------.G.TC.TA.C.TC.......AT-....C------.A.TG..AA..T--------------
6TBGb-3(2,1)        ----------------------------------------.G.TC.TA.C.TC.......AT-....C------.A.TG..AA..T--------------
6TBGc-1(6,B)        ----------------------------------------.G...................-A.G..A------............--------------
6TBGc-2(3,1)        ----------------------------------------.G...................-A.G..A------............--------------
6TBGd(10,B)         ----------------------------------------------------------------------------------------------------
6TBGe(2,1)          ----------------------------------------.G.TC.TA.C.TC.......AT-....C------.A.TG..AA..T--------------
```

```
                             1810      1820      1830      1840      1850      1860      1870      1880      1890      1900
                    ....|....|....|....|....|....|....|....|....|....|....|....|....|....|....|....|....|....|....|....|
NTBGa-1(35,2)       ----------------------------------------------------------------------------------TGGAGAATTGGAATTCAG
NTBGa-2(8,1)        ----------------------------------------------------------------------------------..................
NTBGa-3(6,B)        ----------------------------------------------------------------------------------..................
NTBGa-4(5,B)        ----------------------------------------------------------------------------------..................
NTBGa-6(2,1)        ----------------------------------------------------------------------------------..............G...
NTBGa-7(1,1)        ----------------------------------------------------------------------------------..................
NTBGa-8(1,1)        ----------------------------------------------------------------------------------..................
NTBGa-10(1,1)       ----------------------------------------------------------------------------------..................
NTBGa-11(1,1)       ----------------------------------------------------------------------------------..................
NTBGa-12(1,1)       ----------------------------------------------------------------------------------..................
NTBGa-13(1,1)       ----------------------------------------------------------------------------------..................
NTBGa-15(1,1)       ----------------------------------------------------------------------------------..................
NTBGa-16(1,B)       ----------------------------------------------------------------------------------..................
NTBGb(4,1)          ----------------------------------------------------------------------------------..................
NTBGd(1,1)          ----------------------------------------------------------------------------------..................
NTBGc(2,1)          ----------------------------------------------------------------------------------..................
NTBGe(1,1)          ----------------------------------------------------------------------------------.....G....C.......
P2aTBGa-1(10,2)     ----------------------------------------------------------------------------------..................
P2aTBGa-2(3,1)      ----------------------------------------------------------------------------------..................
P2aTBGa-3(1,1)      ----------------------------------------------------------------------------------..................
P2aTBGb-1(2,2)      ----------------------------------------------------------------------------------..................
P2aTBGb-2(2,1)      ----------------------------------------------------------------------------------..................
P2aTBGb-3(1,1)      ----------------------------------------------------------------------------------..................
P2aTBGb-4(1,1)      ----------------------------------------------------------------------------------..................
P2aTBGc-1(1,B)      ----------------------------------------------------------------------------------......T.CA.......C
P2aTBGc-2(1,1)      ----------------------------------------------------------------------------------......T.CA.......C
P2aTBGc-3(1,1)      ----------------------------------------------------------------------------------......T.CA.......C
P2aTBGc-4(1,1)      ----------------------------------------------------------------------------------......T.CA.......C
P2aTBGc-6(1,1)      CCTGTGTGAGCTGTGGGATGCGATGTTCCACTCATCATGCATTGCTTTTCACTTCCTTTTCCAGTGAAACAAATGGAAAAAT......T.CA.......C
P2aTBGc-5(1,B)      ----------------------------------------------------------------------------------......T.CA.......C
15iTBGa-1(6,B)      ----------------------------------------------------------------------------------..................
15iTBGa-2(4,2)      ----------------------------------------------------------------------------------..................
15iTBGa-3(3,2)      ----------------------------------------------------------------------------------..................
15iTBGa-4(3,B)      ----------------------------------------------------------------------------------..................
15iTBGa-5(2,B)      ----------------------------------------------------------------------------------..................
15iTBGa-6(2,1)      ----------------------------------------------------------------------------------..................
15iTBGa-7(1,B)      ----------------------------------------------------------------------------------..................
15iTBGa-8(1,1)      ----------------------------------------------------------------------------------..................
15iTBGa-9(1,1)      ----------------------------------------------------------------------------------..................
15iTBGb-1(4,2)      ----------------------------------------------------------------------------------.....G....C.......
15iTBGb-2(2,2)      ----------------------------------------------------------------------------------.....G....C.......
15iTBGb-4(1,1)      ----------------------------------------------------------------------------------.....G....C.......
15iTBGb-5(1,B)      ----------------------------------------------------------------------------------.....G....C.......
15iTBGb-7(1,1)      ----------------------------------------------------------------------------------.....G....C.......
15iTBGc-1(3,1)      ----------------------------------------------------------------------------------.....G....C....T..
6TBGa-1(10,1)       ----------------------------------------------------------------------------------.....C....C....T..
6TBGa-2(9,1)        ----------------------------------------------------------------------------------.....C....C....T..
6TBGa-3(3,1)        ----------------------------------------------------------------------------------.....C....C....T..
6TBGa-4(2,1)        ----------------------------------------------------------------------------------..T----..CT.C..TTC
6TBGa-5(1,1)        ----------------------------------------------------------------------------------.....C....C....T..
6TBGb-1(7,B)        ----------------------------------------------------------------------------------......T.CA........
6TBGb-2(6,1)        ----------------------------------------------------------------------------------......T.CA........
6TBGb-3(2,1)        ----------------------------------------------------------------------------------......T.CA........
6TBGc-1(6,B)        ----------------------------------------------------------------------------------..................
6TBGc-2(3,1)        ----------------------------------------------------------------------------------..................
6TBGd(10,B)         ----------------------------------------------------------------------------------------------------
6TBGe(2,1)          ----------------------------------------------------------------------------------......T.CA........

                             1910      1920      1930      1940      1950      1960      1970      1980      1990      2000
                    ....|....|....|....|....|....|....|....|....|....|....|....|....|....|....|....|....|....|....|....|
NTBGa-1(35,2)       TGCTGA----------------------------------------------------------------------------------------------
NTBGa-2(8,1)        ......----------------------------------------------------------------------------------------------
NTBGa-3(6,B)        ......----------------------------------------------------------------------------------------------
NTBGa-4(5,B)        ......----------------------------------------------------------------------------------------------
NTBGa-6(2,1)        ......----------------------------------------------------------------------------------------------
NTBGa-7(1,1)        ......----------------------------------------------------------------------------------------------
NTBGa-8(1,1)        ......----------------------------------------------------------------------------------------------
NTBGa-10(1,1)       ......----------------------------------------------------------------------------------------------
NTBGa-11(1,1)       ......----------------------------------------------------------------------------------------------
NTBGa-12(1,1)       ......----------------------------------------------------------------------------------------------
NTBGa-13(1,1)       ......----------------------------------------------------------------------------------------------
NTBGa-15(1,1)       ......----------------------------------------------------------------------------------------------
NTBGa-16(1,B)       ......----------------------------------------------------------------------------------------------
NTBGb(4,1)          ......----------------------------------------------------------------------------------------------
NTBGd(1,1)          ......----------------------------------------------------------------------------------------------
NTBGc(2,1)          ......----------------------------------------------------------------------------------------------
NTBGe(1,1)          A.....----------------------------------------------------------------------------------------------
P2aTBGa-1(10,2)     ......----------------------------------------------------------------------------------------------
P2aTBGa-2(3,1)      ......----------------------------------------------------------------------------------------------
P2aTBGa-3(1,1)      ......----------------------------------------------------------------------------------------------
P2aTBGb-1(2,2)      ......----------------------------------------------------------------------------------------------
P2aTBGb-2(2,1)      ......GTAAGTTGCAGTCACTGAACTGAGGGAATGTGGGGTCTTCCTAAGGGACTGCGTAGGGGAGAAGTTCCCATGCACTGCTTTTCTCTTTCTTTTC
P2aTBGb-3(1,1)      ......----------------------------------------------------------------------------------------------
P2aTBGb-4(1,1)      ......----------------------------------------------------------------------------------------------
P2aTBGc-1(1,B)      .CT...----------------------------------------------------------------------------------------------
P2aTBGc-2(1,1)      .CT...----------------------------------------------------------------------------------------------
P2aTBGc-3(1,1)      .CT...----------------------------------------------------------------------------------------------
P2aTBGc-4(1,1)      .CT...----------------------------------------------------------------------------------------------
P2aTBGc-6(1,1)      .CT...----------------------------------------------------------------------------------------------
P2aTBGc-5(1,B)      .CT...----------------------------------------------------------------------------------------------
15iTBGa-1(6,B)      ......----------------------------------------------------------------------------------------------
15iTBGa-2(4,2)      ......----------------------------------------------------------------------------------------------
15iTBGa-3(3,2)      ......----------------------------------------------------------------------------------------------
15iTBGa-4(3,B)      ......----------------------------------------------------------------------------------------------
15iTBGa-5(2,B)      ......----------------------------------------------------------------------------------------------
15iTBGa-6(2,1)      ......----------------------------------------------------------------------------------------------
15iTBGa-7(1,B)      ......----------------------------------------------------------------------------------------------
15iTBGa-8(1,1)      ......----------------------------------------------------------------------------------------------
15iTBGa-9(1,1)      ......----------------------------------------------------------------------------------------------
15iTBGb-1(4,2)      A.....----------------------------------------------------------------------------------------------
15iTBGb-2(2,2)      A.....----------------------------------------------------------------------------------------------
15iTBGb-4(1,1)      A.....----------------------------------------------------------------------------------------------
15iTBGb-5(1,B)      A.....----------------------------------------------------------------------------------------------
15iTBGb-7(1,1)      A.....----------------------------------------------------------------------------------------------
15iTBGc-1(3,1)      GT....----------------------------------------------------------------------------------------------
6TBGa-1(10,1)       AT....----------------------------------------------------------------------------------------------
6TBGa-2(9,1)        AT....----------------------------------------------------------------------------------------------
6TBGa-3(3,1)        AT....----------------------------------------------------------------------------------------------
6TBGa-4(2,1)        CTT..C----------------------------------------------------------------------------------------------
6TBGa-5(1,1)        AT....----------------------------------------------------------------------------------------------
6TBGb-1(7,B)        .CT..G----------------------------------------------------------------------------------------------
6TBGb-2(6,1)        .CT..G----------------------------------------------------------------------------------------------
6TBGb-3(2,1)        .CT..G----------------------------------------------------------------------------------------------
6TBGc-1(6,B)        ......----------------------------------------------------------------------------------------------
6TBGc-2(3,1)        ......----------------------------------------------------------------------------------------------
6TBGd(10,B)         ----------------------------------------------------------------------------------------------------
6TBGe(2,1)          .CT..G----------------------------------------------------------------------------------------------
```

```
                             2010      2020      2030      2040      2050      2060      2070      2080      2090      2100
                    ....|....|....|....|....|....|....|....|....|....|....|....|....|....|....|....|....|....|....|....|
NTBGa-1(35,2)       ---AAAAAGACAGTGAAGAGA-------------------------------------------------------------------------------
NTBGa-2(8,1)        ---..................-------------------------------------------------------------------------------
NTBGa-3(6,B)        ---..................-------------------------------------------------------------------------------
NTBGa-4(5,B)        ---..................-------------------------------------------------------------------------------
NTBGa-6(2,1)        ---..................-------------------------------------------------------------------------------
NTBGa-7(1,1)        ---..................-------------------------------------------------------------------------------
NTBGa-8(1,1)        ---..................-------------------------------------------------------------------------------
NTBGa-10(1,1)       ---..................-------------------------------------------------------------------------------
NTBGa-11(1,1)       ---..................-------------------------------------------------------------------------------
NTBGa-12(1,1)       ---..................-------------------------------------------------------------------------------
NTBGa-13(1,1)       ---..................-------------------------------------------------------------------------------
NTBGa-15(1,1)       ---..................-------------------------------------------------------------------------------
NTBGa-16(1,B)       ---..................-------------------------------------------------------------------------------
NTBGb(4,1)          ---..................-------------------------------------------------------------------------------
NTBGd(1,1)          ---..................-------------------------------------------------------------------------------
NTBGc(2,1)          --A..................-------------------------------------------------------------------------------
NTBGe(1,1)          ---.G......T.........-------------------------------------------------------------------------------
P2aTBGa-1(10,2)     ---..................-------------------------------------------------------------------------------
P2aTBGa-2(3,1)      ---..................-------------------------------------------------------------------------------
P2aTBGa-3(1,1)      ---..................-------------------------------------------------------------------------------
P2aTBGb-1(2,2)      ---.G................-------------------------------------------------------------------------------
P2aTBGb-2(2,1)      CAG.G................-------------------------------------------------------------------------------
P2aTBGb-3(1,1)      ---.G................-------------------------------------------------------------------------------
P2aTBGb-4(1,1)      ---.G................-------------------------------------------------------------------------------
P2aTBGc-1(1,B)      ---.G...CGGTA...GATT.-------------------------------------------------------------------------------
P2aTBGc-2(1,1)      ---.G...CGGTA...GATT.-------------------------------------------------------------------------------
P2aTBGc-3(1,1)      ---.G...CGGTA...GATT.-------------------------------------------------------------------------------
P2aTBGc-4(1,1)      ---.G...CGGTA...GATT.-------------------------------------------------------------------------------
P2aTBGc-6(1,1)      ---.G...CGGTA...GATT.-------------------------------------------------------------------------------
P2aTBGc-5(1,B)      ---.G...CGGTA...GATT.-------------------------------------------------------------------------------
15iTBGa-1(6,B)      ---.G................-------------------------------------------------------------------------------
15iTBGa-2(4,2)      ---.G................-------------------------------------------------------------------------------
15iTBGa-3(3,2)      ---.G................-------------------------------------------------------------------------------
15iTBGa-4(3,B)      ---.G................-------------------------------------------------------------------------------
15iTBGa-5(2,B)      ---.G................-------------------------------------------------------------------------------
15iTBGa-6(2,1)      ---.G................-------------------------------------------------------------------------------
15iTBGa-7(1,B)      ---.G................-------------------------------------------------------------------------------
15iTBGa-8(1,1)      ---.G................-------------------------------------------------------------------------------
15iTBGa-9(1,1)      ---.G................-------------------------------------------------------------------------------
15iTBGb-1(4,2)      ---.G..G...T.........-------------------------------------------------------------------------------
15iTBGb-2(2,2)      ---.G..G...T.........-------------------------------------------------------------------------------
15iTBGb-4(1,1)      ---.G..G...T.........-------------------------------------------------------------------------------
15iTBGb-5(1,B)      ---.G..G...T.........-------------------------------------------------------------------------------
15iTBGb-7(1,1)      ---.G..G...T.........-------------------------------------------------------------------------------
15iTBGc-1(3,1)      ---.G.G....T.....A...-------------------------------------------------------------------------------
6TBGa-1(10,1)       ---.G......T.........TGC----------------------------------------------------------------------------
6TBGa-2(9,1)        ---.G......T.........TGC----------------------------------------------------------------------------
6TBGa-3(3,1)        ---.G......T.......AGATGCGTGAGTCTCCCCCTCCCCAAAATAAAAACATTGGGGTCCTCATGTGGATGAGATGTTCCTCTCATCACGCACTGT
6TBGa-4(2,1)        ---.G-----------------------------------------------------------------------------------------------
6TBGa-5(1,1)        ---.G......T.........TGC----------------------------------------------------------------------------
6TBGb-1(7,B)        ---.G...CGGTA---------------------------------------------------------------------------------------
6TBGb-2(6,1)        ---.G...CGGTA---------------------------------------------------------------------------------------
6TBGb-3(2,1)        ---GT..TTTGCAGTC.CCA.ACTGAGGGAATTTGTGGTCCCCCCAGAGGACAATCTAT-----------------------------------------
6TBGc-1(6,B)        ---.G................TG-----------------------------------------------------------------------------
6TBGc-2(3,1)        ---.G................TG-----------------------------------------------------------------------------
6TBGd(10,B)         ----------------------------------------------------------------------------------------------------
6TBGe(2,1)          ---.G...CGGTA---------------------------------------------------------------------------------------

                             2110      2120      2130      2140      2150      2160      2170      2180      2190      2200
                    ....|....|....|....|....|....|....|....|....|....|....|....|....|....|....|....|....|....|....|....|
NTBGa-1(35,2)       ----------------TGGGTTATGGCTTTGGAGATCTGA------------------------------------------------------------
NTBGa-2(8,1)        ----------------........................------------------------------------------------------------
NTBGa-3(6,B)        ----------------........................------------------------------------------------------------
NTBGa-4(5,B)        ----------------........................------------------------------------------------------------
NTBGa-6(2,1)        ----------------........................------------------------------------------------------------
NTBGa-7(1,1)        ----------------........................------------------------------------------------------------
NTBGa-8(1,1)        ----------------........................------------------------------------------------------------
NTBGa-10(1,1)       ----------------........................GTAAGTCTCCCTCCCAACATGGAAGGAATTTATGGTCTTAGCATGGGATCAGCCATGGGA
NTBGa-11(1,1)       ----------------........................GTAAGTCTCCCTCCCAACATGGAAGGAATTTATGGTCTTAGCATGGGATCAGCCATGGGA
NTBGa-12(1,1)       ----------------........................------------------------------------------------------------
NTBGa-13(1,1)       ----------------........................------------------------------------------------------------
NTBGa-15(1,1)       ----------------........................------------------------------------------------------------
NTBGa-16(1,B)       ----------------........................GTAAGTCTCCCTCCCAACATGGAAGGAATTTATGGTCTTAGCATGGGATCAGCCATGGGA
NTBGb(4,1)          ----------------........................------------------------------------------------------------
NTBGd(1,1)          ----------------........................------------------------------------------------------------
NTBGc(2,1)          ----------------........................------------------------------------------------------------
NTBGe(1,1)          ----------------......C....G...C........------------------------------------------------------------
P2aTBGa-1(10,2)     ----------------........................------------------------------------------------------------
P2aTBGa-2(3,1)      ----------------........................------------------------------------------------------------
P2aTBGa-3(1,1)      ----------------........................------------------------------------------------------------
P2aTBGb-1(2,2)      ----------------......C....G...C........------------------------------------------------------------
P2aTBGb-2(2,1)      ----------------......C....G...C........------------------------------------------------------------
P2aTBGb-3(1,1)      ----------------......C....G...C........------------------------------------------------------------
P2aTBGb-4(1,1)      ----------------......C....G...C........------------------------------------------------------------
P2aTBGc-1(1,B)      -------------------------------------C.G------------------------------------------------------------
P2aTBGc-2(1,1)      -------------------------------------C.G------------------------------------------------------------
P2aTBGc-3(1,1)      -------------------------------------C.G------------------------------------------------------------
P2aTBGc-4(1,1)      -------------------------------------C.G------------------------------------------------------------
P2aTBGc-6(1,1)      -------------------------------------C.G------------------------------------------------------------
P2aTBGc-5(1,B)      -------------------------------------C.G------------------------------------------------------------
15iTBGa-1(6,B)      ----------------......C........C...A....------------------------------------------------------------
15iTBGa-2(4,2)      ----------------......C........C...A....------------------------------------------------------------
15iTBGa-3(3,2)      ----------------......C........C...A....------------------------------------------------------------
15iTBGa-4(3,B)      ----------------......C........C...A....------------------------------------------------------------
15iTBGa-5(2,B)      ----------------......C........C...A....------------------------------------------------------------
15iTBGa-6(2,1)      ----------------......C........C...A....------------------------------------------------------------
15iTBGa-7(1,B)      ----------------......C........C...A....------------------------------------------------------------
15iTBGa-8(1,1)      ----------------......C........C...A....------------------------------------------------------------
15iTBGa-9(1,1)      ----------------......C........C...A....------------------------------------------------------------
15iTBGb-1(4,2)      ----------------......C..T.G...C...C....------------------------------------------------------------
15iTBGb-2(2,2)      ----------------......C..T.G...C...C....------------------------------------------------------------
15iTBGb-4(1,1)      ----------------......C..T.G...C...C....------------------------------------------------------------
15iTBGb-5(1,B)      ----------------......C..T.G...C...C....------------------------------------------------------------
15iTBGb-7(1,1)      ----------------......C..T.G...C...C....------------------------------------------------------------
15iTBGc-1(3,1)      ----------------..C...C....G...C........------------------------------------------------------------
6TBGa-1(10,1)       -------------------...C....G...C........------------------------------------------------------------
6TBGa-2(9,1)        -------------------...C....G...C........------------------------------------------------------------
6TBGa-3(3,1)        TTCTACTTTTCCTTTGCA....C....G...C........------------------------------------------------------------
6TBGa-4(2,1)        -------------------...C....G...C........------------------------------------------------------------
6TBGa-5(1,1)        -------------------...C....G...C........------------------------------------------------------------
6TBGb-1(7,B)        -----------------------------..AGA..AC.G------------------------------------------------------------
6TBGb-2(6,1)        -----------------------------..AGA..AC.G------------------------------------------------------------
6TBGb-3(2,1)        -------------------.GG.CAAAAAATCCCC.....------------------------------------------------------------
6TBGc-1(6,B)        ------------------.............C...A....------------------------------------------------------------
6TBGc-2(3,1)        ------------------.............C...A....------------------------------------------------------------
6TBGd(10,B)         ----------------------------------------------------------------------------------------------------
6TBGe(2,1)          -----------------------------..AGA..AC.G------------------------------------------------------------
```

```
                      2210      2220      2230      2240      2250      2260      2270      2280      2290      2300
                  ....|....|....|....|....|....|....|....|....|....|....|....|....|....|....|....|....|....|....|....|
NTBGa-1(35,2)     --------------------------------------------------AGAAACTGGCTGCA--GAACTGGAGAAACA------CTCTGAAGAGATGG
NTBGa-2(8,1)      --------------------------------------------------..............--..............------..............
NTBGa-3(6,B)      --------------------------------------------------..............--..............------..............
NTBGa-4(5,B)      --------------------------------------------------..............--..............------..............
NTBGa-6(2,1)      --------------------------------------------------..............--..............------..............
NTBGa-7(1,1)      --------------------------------------------------..............--..............------..............
NTBGa-8(1,1)      --------------------------------------------------..............--..............------..............
NTBGa-10(1,1)     TGATCATCTGACCCCTCTCATCATGCAATTCATATTTGTTCCTTATGCAG..............--..............------..............
NTBGa-11(1,1)     TGATCATCTGACCCCTCTCATCATGCAATTCATATTTGTTCCTTTTGCAG..............--..............------..............
NTBGa-12(1,1)     --------------------------------------------------..............--..............------..............
NTBGa-13(1,1)     --------------------------------------------------..............--..............------..............
NTBGa-15(1,1)     --------------------------------------------------..............--..............------..............
NTBGa-16(1,B)     TGATCATCTGACCCCTCTCATCATGCAATTCATATTTGTTCCTTTTGCAG..............--..............------..............
NTBGb(4,1)        --------------------------------------------------..............--..............------..............
NTBGd(1,1)        --------------------------------------------------..............--..............------..............
NTBGc(2,1)        --------------------------------------------------..............--..............------..............
NTBGe(1,1)        --------------------------------------------------..G...........--A..T.....G..T.------TAT..C..T..ATC
P2aTBGa-1(10,2)   --------------------------------------------------..............--..............------..............
P2aTBGa-2(3,1)    --------------------------------------------------..............--..............------..............
P2aTBGa-3(1,1)    --------------------------------------------------..............--..............------..............
P2aTBGb-1(2,2)    --------------------------------------------------..............--..............------..............
P2aTBGb-2(2,1)    --------------------------------------------------..............--..............------..............
P2aTBGb-3(1,1)    --------------------------------------------------..............--..............------..............
P2aTBGb-4(1,1)    --------------------------------------------------..............--..............------..............
P2aTBGc-1(1,B)    --------------------------------------------------..G...........--..T......G....------TCT..CT....A..
P2aTBGc-2(1,1)    --------------------------------------------------..G...........--..T......G....------TCT..CT....A..
P2aTBGc-3(1,1)    --------------------------------------------------..G...........--..T......G....------TCT..CT....A..
P2aTBGc-4(1,1)    --------------------------------------------------..G...........--..T......G....------TCT..CT....A..
P2aTBGc-6(1,1)    --------------------------------------------------..G...........--..T......G....------TCT..CT....A..
P2aTBGc-5(1,B)    --------------------------------------------------..G...........--..T......G....------TCT..CT....A..
15iTBGa-1(6,B)    --------------------------------------------------........A...A.--..............------..............
15iTBGa-2(4,2)    --------------------------------------------------........A...A.--..............------..............
15iTBGa-3(3,2)    --------------------------------------------------........A...A.--..............------..............
15iTBGa-4(3,B)    --------------------------------------------------........A...A.--..............------..............
15iTBGa-5(2,B)    --------------------------------------------------........A...A.--..............------..............
15iTBGa-6(2,1)    --------------------------------------------------........A...A.--..............------..............
15iTBGa-7(1,B)    --------------------------------------------------........A...A.--..............------..............
15iTBGa-8(1,1)    --------------------------------------------------........A...A.--..............------..............
15iTBGa-9(1,1)    --------------------------------------------------........A...A.--..............------..............
15iTBGb-1(4,2)    --------------------------------------------------..............--...T...C.G....------..A...........
15iTBGb-2(2,2)    --------------------------------------------------..............--...T...C.G....------..A...........
15iTBGb-4(1,1)    --------------------------------------------------..............--...T...C.G....------..A...........
15iTBGb-5(1,B)    --------------------------------------------------..............--...T...C.G....------..A...........
15iTBGb-7(1,1)    --------------------------------------------------..............--...T...C.G....------..A...........
15iTBGc-1(3,1)    --------------------------------------------------..G..G........--A.............------..............
6TBGa-1(10,1)     --------------------------------------------------...........AG.--......----------------------------
6TBGa-2(9,1)      --------------------------------------------------...........AG.--......----------------------------
6TBGa-3(3,1)      --------------------------------------------------...........AG.--......----------------------------
6TBGa-4(2,1)      --------------------------------------------------...........AG.--......----------------------------
6TBGa-5(1,1)      --------------------------------------------------...........AG.--......----------------------------
6TBGb-1(7,B)      --------------------------------------------------..G...........--..T...----------------------------
6TBGb-2(6,1)      --------------------------------------------------..G...........--..T...----------------------------
6TBGb-3(2,1)      -------------CCATACACTGCTTTTCTCTTTCTTTTCCAGAGAAACG..G...........--..T....GTG.GTGCTGCCT.C.AA.CT.AGAAA
6TBGc-1(6,B)      --------------------------------------------------..............--......----------------------------
6TBGc-2(3,1)      --------------------------------------------------..............--......----------------------------
6TBGd(10,B)       ----------------------------------------------------------------------------------------------------
6TBGe(2,1)        --------------------------------------------------..G...........--..T...----------------------------

                      2310      2320      2330      2340      2350      2360      2370      2380      2390      2400
                  ....|....|....|....|....|....|....|....|....|....|....|....|....|....|....|....|....|....|....|....|
NTBGa-1(35,2)     ----------------------------------------------------------------------------------------------------
NTBGa-2(8,1)      ----------------------------------------------------------------------------------------------------
NTBGa-3(6,B)      ----------------------------------------------------------------------------------------------------
NTBGa-4(5,B)      ----------------------------------------------------------------------------------------------------
NTBGa-6(2,1)      ----------------------------------------------------------------------------------------------------
NTBGa-7(1,1)      ----------------------------------------------------------------------------------------------------
NTBGa-8(1,1)      ----------------------------------------------------------------------------------------------------
NTBGa-10(1,1)     ----------------------------------------------------------------------------------------------------
NTBGa-11(1,1)     ----------------------------------------------------------------------------------------------------
NTBGa-12(1,1)     ----------------------------------------------------------------------------------------------------
NTBGa-13(1,1)     ----------------------------------------------------------------------------------------------------
NTBGa-15(1,1)     ----------------------------------------------------------------------------------------------------
NTBGa-16(1,B)     ----------------------------------------------------------------------------------------------------
NTBGb(4,1)        ----------------------------------------------------------------------------------------------------
NTBGd(1,1)        ----------------------------------------------------------------------------------------------------
NTBGc(2,1)        ----------------------------------------------------------------------------------------------------
NTBGe(1,1)        ----------------------------------------------------------------------------------------------------
P2aTBGa-1(10,2)   ----------------------------------------------------------------------------------------------------
P2aTBGa-2(3,1)    ----------------------------------------------------------------------------------------------------
P2aTBGa-3(1,1)    ----------------------------------------------------------------------------------------------------
P2aTBGb-1(2,2)    ----------------------------------------------------------------------------------------------------
P2aTBGb-2(2,1)    ----------------------------------------------------------------------------------------------------
P2aTBGb-3(1,1)    ----------------------------------------------------------------------------------------------------
P2aTBGb-4(1,1)    GTGAGTCTCCCCACCCCAATTATAAATGCTGGGGACTTCTTGTGGGAGCTGTGGGATGAGCTCGTGCTCTCATCATGTGCTGTTTCTGCTTTTCCTTTGC
P2aTBGc-1(1,B)    ----------------------------------------------------------------------------------------------------
P2aTBGc-2(1,1)    ----------------------------------------------------------------------------------------------------
P2aTBGc-3(1,1)    ----------------------------------------------------------------------------------------------------
P2aTBGc-4(1,1)    ----------------------------------------------------------------------------------------------------
P2aTBGc-6(1,1)    ----------------------------------------------------------------------------------------------------
P2aTBGc-5(1,B)    ----------------------------------------------------------------------------------------------------
15iTBGa-1(6,B)    ----------------------------------------------------------------------------------------------------
15iTBGa-2(4,2)    ----------------------------------------------------------------------------------------------------
15iTBGa-3(3,2)    ----------------------------------------------------------------------------------------------------
15iTBGa-4(3,B)    ----------------------------------------------------------------------------------------------------
15iTBGa-5(2,B)    ----------------------------------------------------------------------------------------------------
15iTBGa-6(2,1)    ----------------------------------------------------------------------------------------------------
15iTBGa-7(1,B)    ----------------------------------------------------------------------------------------------------
15iTBGa-8(1,1)    ----------------------------------------------------------------------------------------------------
15iTBGa-9(1,1)    ----------------------------------------------------------------------------------------------------
15iTBGb-1(4,2)    ----------------------------------------------------------------------------------------------------
15iTBGb-2(2,2)    ----------------------------------------------------------------------------------------------------
15iTBGb-4(1,1)    ----------------------------------------------------------------------------------------------------
15iTBGb-5(1,B)    ----------------------------------------------------------------------------------------------------
15iTBGb-7(1,1)    ----------------------------------------------------------------------------------------------------
15iTBGc-1(3,1)    ----------------------------------------------------------------------------------------------------
6TBGa-1(10,1)     ----------------------------------------------------------------------------------------------------
6TBGa-2(9,1)      ----------------------------------------------------------------------------------------------------
6TBGa-3(3,1)      ----------------------------------------------------------------------------------------------------
6TBGa-4(2,1)      ----------------------------------------------------------------------------------------------------
6TBGa-5(1,1)      ----------------------------------------------------------------------------------------------------
6TBGb-1(7,B)      ----------------------------------------------------------------------------------------------------
6TBGb-2(6,1)      ----------------------------------------------------------------------------------------------------
6TBGb-3(2,1)      ----------------------------------------------------------------------------------------------------
6TBGc-1(6,B)      ----------------------------------------------------------------------------------------------------
6TBGc-2(3,1)      ----------------------------------------------------------------------------------------------------
6TBGd(10,B)       ----------------------------------------------------------------------------------------------------
6TBGe(2,1)        ----------------------------------------------------------------------------------------------------
```

```
                           2410      2420      2430      2440      2450      2460      2470      2480      2490      2500
                    ....|....|....|....|....|....|....|....|....|....|....|....|....|....|....|....|....|....|....|....|
NTBGa-1(35,2)       --GGACAAGGGATTTAAAGTTGG-----------------------------------------------------------------------------
NTBGa-2(8,1)        --.....................-----------------------------------------------------------------------------
NTBGa-3(6,B)        --.....................-----------------------------------------------------------------------------
NTBGa-4(5,B)        --.....................-----------------------------------------------------------------------------
NTBGa-6(2,1)        --.....................-----------------------------------------------------------------------------
NTBGa-7(1,1)        --.....................-----------------------------------------------------------------------------
NTBGa-8(1,1)        --.....................-----------------------------------------------------------------------------
NTBGa-10(1,1)       --.....................-----------------------------------------------------------------------------
NTBGa-11(1,1)       --.....................-----------------------------------------------------------------------------
NTBGa-12(1,1)       --.....................-----------------------------------------------------------------------------
NTBGa-13(1,1)       --.....................-----------------------------------------------------------------------------
NTBGa-15(1,1)       --.....................-----------------------------------------------------------------------------
NTBGa-16(1,B)       --.....................-----------------------------------------------------------------------------
NTBGb(4,1)          --.....................-----------------------------------------------------------------------------
NTBGd(1,1)          --.....................-----------------------------------------------------------------------------
NTBGc(2,1)          --.....................-----------------------------------------------------------------------------
NTBGe(1,1)          --...G....A..G....----------------------------------------------------------------------------------
P2aTBGa-1(10,2)     --.....................-----------------------------------------------------------------------------
P2aTBGa-2(3,1)      --.....................-----------------------------------------------------------------------------
P2aTBGa-3(1,1)      --.....................-----------------------------------------------------------------------------
P2aTBGb-1(2,2)      --...........G.........-----------------------------------------------------------------------------
P2aTBGb-2(2,1)      --...........G.........-----------------------------------------------------------------------------
P2aTBGb-3(1,1)      --...........G.........-----------------------------------------------------------------------------
P2aTBGb-4(1,1)      AG...........G.........-----------------------------------------------------------------------------
P2aTBGc-1(1,B)      --ATTT...CAC.GC.G.TC..A-----------------------------------------------------------------------------
P2aTBGc-2(1,1)      --ATTT...CAC.GC.G.TC..A-----------------------------------------------------------------------------
P2aTBGc-3(1,1)      --ATTT...CAC.GC.G.TC..A-----------------------------------------------------------------------------
P2aTBGc-4(1,1)      --ATTT...CAC.GC.G.TC..A-----------------------------------------------------------------------------
P2aTBGc-6(1,1)      --ATTT...CAC.GC.G.TC..A-----------------------------------------------------------------------------
P2aTBGc-5(1,B)      --ATTT...CAC.GC.G.TC..A-----------------------------------------------------------------------------
15iTBGa-1(6,B)      --...........G.........-----------------------------------------------------------------------------
15iTBGa-2(4,2)      --...........G.........-----------------------------------------------------------------------------
15iTBGa-3(3,2)      --...........G.........-----------------------------------------------------------------------------
15iTBGa-4(3,B)      --...........G.........-----------------------------------------------------------------------------
15iTBGa-5(2,B)      --...........G.........-----------------------------------------------------------------------------
15iTBGa-6(2,1)      --...........G.........-----------------------------------------------------------------------------
15iTBGa-7(1,B)      --...........G.........-----------------------------------------------------------------------------
15iTBGa-8(1,1)      --...........G.........-----------------------------------------------------------------------------
15iTBGa-9(1,1)      --...........G.........-----------------------------------------------------------------------------
15iTBGb-1(4,2)      --..CT.TT.AG.G...C....A-----------------------------------------------------------------------------
15iTBGb-2(2,2)      --..CT.TT.AG.G...C....A-----------------------------------------------------------------------------
15iTBGb-4(1,1)      --..CT.TT.AG.G...C....A-----------------------------------------------------------------------------
15iTBGb-5(1,B)      --..CT.TT.AG.G...C....A-----------------------------------------------------------------------------
15iTBGb-7(1,1)      --..CT.TT.AG.G...C....AGTGAGTCTCTCTTCCCAAACCAAACAGATTTGGGGGCTTCCCATGGGATCAGCCATCGGATGATAATCGGACCCTTC
15iTBGc-1(3,1)      --.......A.C----------------------------------------------------------------------------------------
6TBGa-1(10,1)       ----------------------------------------------------------------------------------------------------
6TBGa-2(9,1)        ----------------------------------------------------------------------------------------------------
6TBGa-3(3,1)        ----------------------------------------------------------------------------------------------------
6TBGa-4(2,1)        ----------------------------------------------------------------------------------------------------
6TBGa-5(1,1)        ----------------------------------------------------------------------------------------------------
6TBGb-1(7,B)        ----------------------------------------------------------------------------------------------------
6TBGb-2(6,1)        ----------------------------------------------------------------------------------------------------
6TBGb-3(2,1)        --CAGGGGCCTGAC.-------------------------------------------------------------------------------------
6TBGc-1(6,B)        ----------------------------------------------------------------------------------------------------
6TBGc-2(3,1)        ----------------------------------------------------------------------------------------------------
6TBGd(10,B)         ----------------------------------------------------------------------------------------------------
6TBGe(2,1)          ----------------------------------------------------------------------------------------------------

                           2510      2520      2530      2540      2550      2560      2570      2580      2590      2600
                    ....|....|....|....|....|....|....|....|....|....|....|....|....|....|....|....|....|....|....|....|
NTBGa-1(35,2)       ---------------------------------AGCGACTAGCTGCCAAACTGGAACATCAAACTAAAGAATTG----GAGAAACAGCATTCACAGTTCC
NTBGa-2(8,1)        ---------------------------------.........................................----......................
NTBGa-3(6,B)        ---------------------------------.........................................----......................
NTBGa-4(5,B)        ---------------------------------.........................................----......................
NTBGa-6(2,1)        ---------------------------------.........................................----......................
NTBGa-7(1,1)        ---------------------------------.........................................----......................
NTBGa-8(1,1)        ---------------------------------.........................................----......................
NTBGa-10(1,1)       ---------------------------------.........................................----......................
NTBGa-11(1,1)       ---------------------------------.........................................----......................
NTBGa-12(1,1)       ---------------------------------.........................................----......................
NTBGa-13(1,1)       ---------------------------------.........................................----......................
NTBGa-15(1,1)       ---------------------------------.........................................----......................
NTBGa-16(1,B)       ---------------------------------.........................................----......................
NTBGb(4,1)          ---------------------------------.........................................----......................
NTBGd(1,1)          ---------------------------------.........................................----......................
NTBGc(2,1)          ---------------------------------.........................................----......................
NTBGe(1,1)          ---------------------------------.TAATA...............C...A...............----......................
P2aTBGa-1(10,2)     ---------------------------------.........................................----......................
P2aTBGa-2(3,1)      ---------------------------------.........................................----......................
P2aTBGa-3(1,1)      ---------------------------------.........................................----......................
P2aTBGb-1(2,2)      ---------------------------------............A............................----......................
P2aTBGb-2(2,1)      ---------------------------------............A............................----......................
P2aTBGb-3(1,1)      ---------------------------------............A............................----......................
P2aTBGb-4(1,1)      ---------------------------------............A............................----......................
P2aTBGc-1(1,B)      ---------------------------------...T.........A.......TGG.A....GAG...C.G..----...G...G.G........C.GA
P2aTBGc-2(1,1)      ---------------------------------...T.........A.......TGG.A....GAG...C.G..GGCA...G...G.G........C.GA
P2aTBGc-3(1,1)      ---------------------------------...T.........A.......TGG.A....GAG...C.G..GGCA...G...G.G........C.GA
P2aTBGc-4(1,1)      ---------------------------------...T.........A.......TGG.A....GAG...C.G..G----..G...G.G........C.GA
P2aTBGc-6(1,1)      ---------------------------------...T.........A.......TGG.A..G.GAG...C.G..----...G...G.G........C.GA
P2aTBGc-5(1,B)      ---------------------------------...T.........A.......TGG.A....GAG...C.G..G----..G...G.G........C.GA
15iTBGa-1(6,B)      ---------------------------------.........................A.G.............----..C...................
15iTBGa-2(4,2)      ---------------------------------.........................A.G.............----..C...................
15iTBGa-3(3,2)      ---------------------------------.........................A.G.............----..C...................
15iTBGa-4(3,B)      ---------------------------------.........................A.G.............----..C...................
15iTBGa-5(2,B)      ---------------------------------.........................A.G.............----..C...................
15iTBGa-6(2,1)      ---------------------------------.........................A.G.............----..C...................
15iTBGa-7(1,B)      ---------------------------------.........................A.G.............----..C...................
15iTBGa-8(1,1)      ---------------------------------.........................A.G.............----..C...................
15iTBGa-9(1,1)      ---------------------------------.........................A.G.............----..C...................
15iTBGb-1(4,2)      ---------------------------------............A............A.G.............----..C.....A.............
15iTBGb-2(2,2)      ---------------------------------............A............A.G.............----..C.....A.............
15iTBGb-4(1,1)      ---------------------------------............A............A.G.............----..C.....A.............
15iTBGb-5(1,B)      ---------------------------------............A............A.G.............----..C.....A.............
15iTBGb-7(1,1)      TCATCATGCGTATCTTATTGGTTCCTTTTGCAG............A............A.G.............----..C.....A.............
15iTBGc-1(3,1)      ---------------TACTAGCTGCTGAACTGAG..C.TGGAA...A..GTC.A.TA.A.T.G..GCCA..C..----...CT.................
6TBGa-1(10,1)       ---------------------------------------------------------------------------------------------------G
6TBGa-2(9,1)        ---------------------------------------------------------------------------------------------------G
6TBGa-3(3,1)        ---------------------------------------------------------------------------------------------------G
6TBGa-4(2,1)        ---------------------------------------------------------------------------------------------------G
6TBGa-5(1,1)        ---------------------------------------------------------------------------------------------------G
6TBGb-1(7,B)        ---------------------------------------------------------------------------------------------------G
6TBGb-2(6,1)        ---------------------------------------------------------------------------------------------------G
6TBGb-3(2,1)        -----------------------------------.TGTG...A..GGG.TG...TGT..CTCTC.TCATG.GT----TGCTTTTCT.T.C..TTACCAG
6TBGc-1(6,B)        ---------------------------------------------------------------------------------------------------G
6TBGc-2(3,1)        ---------------------------------------------------------------------------------------------------G
6TBGd(10,B)         ----------------------------------------------------------------------------------------------------
6TBGe(2,1)          ---------------------------------------------------------------------------------------------------G
```

```
                          2610      2620      2630      2640      2650      2660      2670      2680      2690      2700
                 ....|....|....|....|....|....|....|....|....|....|....|....|....|....|....|....|....|....|....|....|
NTBGa-1(35,2)    AGAGACACTTTCAGAATATGTATTTAAGTGCTGGAAAACAGAAGAAAATGG--TTACAAAACTG------------------------------------
NTBGa-2(8,1)     ...................................................--...........------------------------------------
NTBGa-3(6,B)     ...................................................--...........------------------------------------
NTBGa-4(5,B)     ...................................................--...........------------------------------------
NTBGa-6(2,1)     ............................G......................--...........------------------------------------
NTBGa-7(1,1)     .....................................T.............--...........------------------------------------
NTBGa-8(1,1)     ...................................................--...........------------------------------------
NTBGa-10(1,1)    ...................................................--...........------------------------------------
NTBGa-11(1,1)    ...................................................--...........------------------------------------
NTBGa-12(1,1)    ...................................................--...........------------------------------------
NTBGa-13(1,1)    ...................................................--...........------------------------------------
NTBGa-15(1,1)    ...................................................--...........------------------------------------
NTBGa-16(1,B)    ...................................................--...........------------------------------------
NTBGb(4,1)       ...................................................--...........------------------------------------
NTBGd(1,1)       ...................................................--...........------------------------------------
NTBGc(2,1)       ...................................................--...........------------------------------------
NTBGe(1,1)       .C............CG....G............T...C.........C...--...........------------------------------------
P2aTBGa-1(10,2)  ...................................................--...........------------------------------------
P2aTBGa-2(3,1)   ...................................................--...........------------------------------------
P2aTBGa-3(1,1)   ...................................................--...........------------------------------------
P2aTBGb-1(2,2)   ..............................T..T.................--...........------------------------------------
P2aTBGb-2(2,1)   ..............................T..T........GT..GTCTCCC.CC.TGC..A.------------------------------------
P2aTBGb-3(1,1)   ..............................T..T.................--...........------------------------------------
P2aTBGb-4(1,1)   ..............................T..T.................--...........------------------------------------
P2aTBGc-1(1,B)   G..A...G.A.G.A..GT..GG..CGC......C...T.T.....C.CAAC--...A...GT..------------------------------------
P2aTBGc-2(1,1)   G..A...G.A.G.A..GT..GG..CGC......C...T.T.....C.CAAC--...A...GT..------------------------------------
P2aTBGc-3(1,1)   G..A...G.A.G.A..GT..GG..CGC......C...T.T.....C.CAAC--...A...GT..------------------------------------
P2aTBGc-4(1,1)   G..A...G.A.G.A..GT..GG..CGC......C...T.T.....C.CAAC--...A...GT..------------------------------------
P2aTBGc-6(1,1)   G..A...G.A.G.A..GT..GG..CGC......C...T.T.....C.CAAC--...A...GT..------------------------------------
P2aTBGc-5(1,B)   G..A...G.A.G.A..GT..GG..CGC......C...T.T.....C.CAAC--...A...GT..------------------------------------
15iTBGa-1(6,B)   .C.................................................--...........------------------------------------
15iTBGa-2(4,2)   .C.................................................--...........------------------------------------
15iTBGa-3(3,2)   .C.................................................--...........GGTGAGTCCAACCTCCCAAACTAAATTAAAAGCGGT
15iTBGa-4(3,B)   .C.................................................--...........------------------------------------
15iTBGa-5(2,B)   .C.................................................--...........------------------------------------
15iTBGa-6(2,1)   .C.................................................--...........------------------------------------
15iTBGa-7(1,B)   .C.................................................--...........------------------------------------
15iTBGa-8(1,1)   .C----.............................................--...........------------------------------------
15iTBGa-9(1,1)   .C.................................................--...........------------------------------------
15iTBGb-1(4,2)   ....................G....C..................G..C...--...........------------------------------------
15iTBGb-2(2,2)   ....................G....C..................G..C...--...........------------------------------------
15iTBGb-4(1,1)   ....................G....C..................G..C...--...........------------------------------------
15iTBGb-5(1,B)   ....................G....C..................G..C...--...........------------------------------------
15iTBGb-7(1,1)   ....................G....C..................G..C...--...........------------------------------------
15iTBGc-1(3,1)   ....................G.......................G..C...--.........T.------------------------------------
6TBGa-1(10,1)    ..GA.A.T.C.ACAGTG..T.GGAC.T.G.A..T..TGTT....GT.C.A.--C.G.C......GGACACAAAGCTAAAGAATTGGAGAAACAGCATTTA
6TBGa-2(9,1)     ..GA.A.T.C.ACAGTG..T.GGAC.T.G.A..T..TGTT....GT.C.A.--C.G.C......GGACACAAAGCTAAAGAATTGGAGAAACAGCATTTA
6TBGa-3(3,1)     ..GA.A.T.C.ACAGTG..T.GGAC.T.G.A..T..TGTT....GT.C.A.--C.G.C......GGACACAAAGCTAAAGAATTGGAGAAACAGCATTTA
6TBGa-4(2,1)     ..GA.A.T.C.ACAGTG..T.GGAC.T.G.A..T..TGTT....GT.C.A.--C.G.C......GGACACAAAGCTAAAGAATTGGAGAAACAGCATTTA
6TBGa-5(1,1)     ..GA.A.T.C.ACAGTG..T.GGAC.T.G.A..T..TGTT....GT.C.A.--C.G.C......GGACACAAAGCTAAAGAATTGGAGAAACAGCATTTA
6TBGb-1(7,B)     ..GA...TC..GCTG.G.A.G.......CA...C.G.T.T....CT.C.A.--C.G........GTGGAACAAAGAGAAGCAGTGGAGGAATGGGATTCA
6TBGb-2(6,1)     ..GA...TC..GCTG.G.A.G.......CA...C.G.T.T....CT.C.A.--C.G........GTGGAACAAAGAGAAGCAGTGG--GAATGGGATTCA
6TBGb-3(2,1)     ..GA...TC..GCTG.G.A.G.......CA...C.G.T.T....CT.C.A.--C.G........GTGGAACAAAGAGAAGCAGTGGAGGAATGGGATTCA
6TBGc-1(6,B)     ...A.....C.G.AG.G...GGGAC...G.A.TT...GTT.G..CG.C.A.--C.G.C......GAACATCAAACTAAAGAATTGGAGAAACAGCATTCA
6TBGc-2(3,1)     ...A.....C.G.AG.G...GGGAC...G.A.TT...GTT.G..CG.C.A.--C.G.C......GAACATCAAACTAAAGAATTGGAGAAACAGCATTCA
6TBGd(10,B)      ----------------------------------------------------------------------------------------------------
6TBGe(2,1)       ..GA...TC..GCTG.G.A.G.......CA...C.G.T.T....CT.C.A.--C.G........GTGGAACAAAGAGAAGCAGTGGAGGAATGGGATTCA

                          2710      2720      2730      2740      2750      2760      2770      2780      2790      2800
                 ....|....|....|....|....|....|....|....|....|....|....|....|....|....|....|....|....|....|....|....|
NTBGa-1(35,2)    ---------------------------------------------------------------------GAGGAACACTGTGAATGGATGG-TGAGAAGG
NTBGa-2(8,1)     ---------------------------------------------------------------------......................-........
NTBGa-3(6,B)     ---------------------------------------------------------------------......................-........
NTBGa-4(5,B)     ---------------------------------------------------------------------......................-........
NTBGa-6(2,1)     ---------------------------------------------------------------------......................-........
NTBGa-7(1,1)     ---------------------------------------------------------------------......................-........
NTBGa-8(1,1)     ---------------------------------------------------------------------......................-........
NTBGa-10(1,1)    ---------------------------------------------------------------------......................-........
NTBGa-11(1,1)    ---------------------------------------------------------------------......................-........
NTBGa-12(1,1)    ---------------------------------------------------------------------......................-........
NTBGa-13(1,1)    ---------------------------------------------------------------------......................-........
NTBGa-15(1,1)    ---------------------------------------------------------------------......................-........
NTBGa-16(1,B)    ---------------------------------------------------------------------......................-........
NTBGb(4,1)       ---------------------------------------------------------------------......................-........
NTBGd(1,1)       ---------------------------------------------------------------------......................-........
NTBGc(2,1)       ---------------------------------------------------------------------......................-........
NTBGe(1,1)       ---------------------------------------------------------------------..........T...........-A.......
P2aTBGa-1(10,2)  ---------------------------------------------------------------------......................-........
P2aTBGa-2(3,1)   ---------------------------------------------------------------------......................-........
P2aTBGa-3(1,1)   ---------------------------------------------------------------------......................-........
P2aTBGb-1(2,2)   ---------------------------------------------------------------------....C...---------------..T...CA
P2aTBGb-2(2,1)   ---------------------------------------------------------------------A......CTACA.TT.TCCCATGG..TC..C
P2aTBGb-3(1,1)   ---------------------------------------------------------------------....C...---------------..T...CA
P2aTBGb-4(1,1)   ---------------------------------------------------------------------....C...---------------..T...CA
P2aTBGc-1(1,B)   ---------------------------------------------------------------------..----------------------..ACGAA
P2aTBGc-2(1,1)   ---------------------------------------------------------------------..----------------------..ACGAA
P2aTBGc-3(1,1)   ---------------------------------------------------------------------..----------------------..ACGAA
P2aTBGc-4(1,1)   ---------------------------------------------------------------------..----------------------..ACGAA
P2aTBGc-6(1,1)   ---------------------------------------------------------------------..----------------------..ACGAA
P2aTBGc-5(1,B)   ---------------------------------------------------------------------..----------------------..ACGAA
15iTBGa-1(6,B)   ---------------------------------------------------------------------......................-........
15iTBGa-2(4,2)   ---------------------------------------------------------------------......................-........
15iTBGa-3(3,2)   CAGAC---TTTGTGAGCTGTGGGATGAGATGTTCCTCTCATCATGTGCTGCTTTCCTTTTACTTCTCCA......................-........
15iTBGa-4(3,B)   ---------------------------------------------------------------------......................-........
15iTBGa-5(2,B)   ---------------------------------------------------------------------......................-........
15iTBGa-6(2,1)   ---------------------------------------------------------------------......................-........
15iTBGa-7(1,B)   ---------------------------------------------------------------------......................-........
15iTBGa-8(1,1)   ---------------------------------------------------------------------......................-........
15iTBGa-9(1,1)   ---------------------------------------------------------------------......................-........
15iTBGb-1(4,2)   ---------------------------------------------------------------------...............CA.....-A.......
15iTBGb-2(2,2)   ---------------------------------------------------------------------...............CA.....-A.......
15iTBGb-4(1,1)   ---------------------------------------------------------------------...............CA.....-A.......
15iTBGb-5(1,B)   ---------------------------------------------------------------------...............CA.....-A.......
15iTBGb-7(1,1)   ---------------------------------------------------------------------...............CA.....-A.......
15iTBGc-1(3,1)   ---------------------------------------------------------------------.....C.........C......-........
6TBGa-1(10,1)    CAGTTCTACAGACACTTTCAGCATATGTATTCAAGTGCTGGAAAACAGAAGGAACTGGTTACAAAATTG...............CA.....-A.......
6TBGa-2(9,1)     CAGTTCTACAGACACTTTCAGCATATGTATTCAAGTGCTGGAAAACAGAAGGAACTGGTTACAAAATTG...............CA.....-A.......
6TBGa-3(3,1)     CAGTTCTACAGACACTTTCAGCATATGTATTCAAGTGCTGGAAAACAGAAGGAACTGGTTACAAAATTG...............CA.....-A.......
6TBGa-4(2,1)     CAGTTCTACA----CTTTCAGCATATGTATTCAAGTGCTGGAAAACAGAAGGAACTGGTTACAAAATTG...............CA.....-A.......
6TBGa-5(1,1)     CAGTTCTACAGACACTTTCAGCATATGTATTCAAGTGCTGGAAAACAGAAGGAACTGGTTACAAAATTG...............CA.....-A.......
6TBGb-1(7,B)     CAGCTGAGGAAACAGTACGAAAAGTTGGGTTCGCGTGCTGCAAATCTGAAGACACAACTTAAAAAGTTG...A.CG.AAT....GAAG...-A-.A.CAC
6TBGb-2(6,1)     CAGCTGAGGAAACAGTACGAAAAGTTGGGTTCGCGTGCTGCAAATCTGAAGACACAACTTAAAAAGTTG...A.CG.AAT....GAAG...-A..A.CAC
6TBGb-3(2,1)     CAGCTGAGGAAACAGTACGAAAAGTTGGGTTCGCGTGCTGCAAATCTGAAGACACAACTTAAAAAGTTG...A.CG.AAT....GAAG...-A..A.CAC
6TBGc-1(6,B)     CAGTTCCAGAGACACTTTCAGAATATGTATTTAAGTGCTGGAAAACAGAAGAAAATGGTTACAAAACTG......................-........
6TBGc-2(3,1)     CAGTTCCAGAGACACTTTCAGAATATGTATTTAAGTGCTGGAAAACAGAAGAAAATGGTTACAAAACTG......................-........
6TBGd(10,B)      ----------------------------------------------------------------------------------------------------
6TBGe(2,1)       CAGCTGAGGAAACAGTACGAAAAGTTGGGTTCGCGTGCTGCAAATCTGAAGACACAACTTAAAAAGTTG...A.CG.AAT....GAAG...-A-.A.CAC
```

```
                        2810      2820      2830      2840      2850      2860      2870      2880      2890      2900
                  ....|....|....|....|....|....|....|....|....|....|....|....|....|....|....|....|....|....|....|....|
NTBGa-1(35,2)     AATGTAAAGTTG----------------------------------------------------------------------------------------
NTBGa-2(8,1)      ............----------------------------------------------------------------------------------------
NTBGa-3(6,B)      ............----------------------------------------------------------------------------------------
NTBGa-4(5,B)      ............----------------------------------------------------------------------------------------
NTBGa-6(2,1)      ............----------------------------------------------------------------------------------------
NTBGa-7(1,1)      ............----------------------------------------------------------------------------------------
NTBGa-8(1,1)      ............----------------------------------------------------------------------------------------
NTBGa-10(1,1)     ............----------------------------------------------------------------------------------------
NTBGa-11(1,1)     ............----------------------------------------------------------------------------------------
NTBGa-12(1,1)     ............GGTGAGTCTTCTT------------------------CCCCAACCAAAGA---------------------------------GATTC
NTBGa-13(1,1)     ............----------------------------------------------------------------------------------------
NTBGa-15(1,1)     ............----------------------------------------------------------------------------------------
NTBGa-16(1,B)     ............----------------------------------------------------------------------------------------
NTBGb(4,1)        ............----------------------------------------------------------------------------------------
NTBGd(1,1)        ............----------------------------------------------------------------------------------------
NTBGc(2,1)        ............----------------------------------------------------------------------------------------
NTBGe(1,1)        ............----------------------------------------------------------------------------------------
P2aTBGa-1(10,2)   ............GAGATACCAGCTGTAAAAGTGGTGAGA----------AGGAATGTAAAGTTGGAGA--------------------------------
P2aTBGa-2(3,1)    ............GAGATACCAGCTGTAAAAGTGGTGAGA----------AGGAATGTAAAGTTGGAGA--------------------------------
P2aTBGa-3(1,1)    ............GAGATACCAGCTGTAAAAGTGGTGAGA----------AGGAATGTAAAGTTGGAGA--------------------------------
P2aTBGb-1(2,2)    G...------------------------------------------------------------------------------------------------
P2aTBGb-2(2,1)    C...GG.TCA.CATCCGACTCTTCTCATCATGAATTTCGTCTTTCTTTCTTTTGCAGAGAAAATGGT---------------------------------
P2aTBGb-3(1,1)    G...------------------------------------------------------------------------------------------------
P2aTBGb-4(1,1)    G...GGTGAG.CTCCCCTCCCAAATTAAAAATGTTGGGGTCTTCCTGTGAGAGCTGTGGGATGAGCTG--------------------------------
P2aTBGc-1(1,B)    .T..A.G.AG..GAGAAA-CACCTT----------AAAAAGACTGGTAGACGTGCTCCTAATCTGAAG--------------------------------
P2aTBGc-2(1,1)    .T..A.G.AG..GAGAAA-CACCTT----------AAAAAGACTGGTAGACGTGCTCCTAATCTGAGTAAGTCTTCCTCCCCAAACCAAAGAGATTGGGG
P2aTBGc-3(1,1)    .T..A.G.AG..GAGAAA-CACCTT----------AAAAAGACTGGTAGACGTGCTCCTAATCTGAAG--------------------------------
P2aTBGc-4(1,1)    .T..A.G.AG..GAGAAA-CACCTT----------AAAAAGACTGGTAGACGTGCTCCTAATCTGAAG--------------------------------
P2aTBGc-6(1,1)    .T..A.G.AG..GAGAAA-CACCTT----------AAAAAGACTGGTAGACGTGCTCCTAATCTGAAG--------------------------------
P2aTBGc-5(1,B)    .T..A.G.AG..GAGAAA-CACCTT----------AAAAAGACTGGTAGACGTGCTCCTAATCTGAAG--------------------------------
15iTBGa-1(6,B)    ........T...GAGGCAGCAGC-----------------------------------------------------------------------------
15iTBGa-2(4,2)    ........T...GAGGCAGCAGC-----------------------------------------------------------------------------
15iTBGa-3(3,2)    ........T...GAGGCAGCAGC-----------------------------------------------------------------------------
15iTBGa-4(3,B)    ........T...GAGGCAGCAGC-----------------------------------------------------------------------------
15iTBGa-5(2,B)    ........T...GAGGCAGCAGC-----------------------------------------------------------------------------
15iTBGa-6(2,1)    ........T...GAGGCAGCAGC-----------------------------------------------------------------------------
15iTBGa-7(1,B)    ........T...GAGGCAGCAGC-----------------------------------------------------------------------------
15iTBGa-8(1,1)    ........T...GAGGCAGCAGC-----------------------------------------------------------------------------
15iTBGa-9(1,1)    ........T...GAGGCAGCAGC-----------------------------------------------------------------------------
15iTBGb-1(4,2)    ........--------------------------------------------------------------------------------------------
15iTBGb-2(2,2)    ........--------------------------------------------------------------------------------------------
15iTBGb-4(1,1)    ........--------------------------------------------------------------------------------------------
15iTBGb-5(1,B)    ........--------------------------------------------------------------------------------------------
15iTBGb-7(1,1)    ........--------------------------------------------------------------------------------------------
15iTBGc-1(3,1)    ........--------------------------------------------------------------------------------------------
6TBGa-1(10,1)     ....C.......GAGGCAGCAGC-----------------------------------------------------------------------------
6TBGa-2(9,1)      ....C.......GAGGCAGCAGC-----------------------------------------------------------------------------
6TBGa-3(3,1)      ....C.......GAGGCAGCAGC-----------------------------------------------------------------------------
6TBGa-4(2,1)      ....C.......GAGGCAGCAGC-----------------------------------------------------------------------------
6TBGa-5(1,1)      ....C.......GAGGCAGCAGC-----------------------------------------------------------------------------
6TBGb-1(7,B)      CT.AA....A.TGGTATACGTGC-----------------------------------------------------------------------------
6TBGb-2(6,1)      CT.AA....A.TGGTATACGTGC-----------------------------------------------------------------------------
6TBGb-3(2,1)      CT.AA....A.TGGTATACGTGC-----------------------------------------------------------------------------
6TBGc-1(6,B)      ............GAGGCAGCAGC-----------------------------------------------------------------------------
6TBGc-2(3,1)      ............GAGGCAGCAGC-----------------------------------------------------------------------------
6TBGd(10,B)       ----------------------------------------------------------------------------------------------------
6TBGe(2,1)        CT.AA....A.TGGTATACGTGC-----------------------------------------------------------------------------

                        2910      2920      2930      2940      2950      2960      2970      2980      2990      3000
                  ....|....|....|....|....|....|....|....|....|....|....|....|....|....|....|....|....|....|....|....|
NTBGa-1(35,2)     --------------------------------------------------------------------------GAGATACCAGCTGTAAAAGTGGGGCA
NTBGa-2(8,1)      --------------------------------------------------------------------------..........................
NTBGa-3(6,B)      --------------------------------------------------------------------------..........................
NTBGa-4(5,B)      --------------------------------------------------------------------------..........................
NTBGa-6(2,1)      --------------------------------------------------------------------------..........................
NTBGa-7(1,1)      --------------------------------------------------------------------------........................T.
NTBGa-8(1,1)      --------------------------------------------------------------------------..........................
NTBGa-10(1,1)     --------------------------------------------------------------------------..........................
NTBGa-11(1,1)     --------------------------------------------------------------------------..........................
NTBGa-12(1,1)     GGAGTCTTCCATGGGATCAGCCATGGGATGATAACCTGAACCTTATCATGTGTTTCTTATTTGTTCCTTTTGCA..........................
NTBGa-13(1,1)     --------------------------------------------------------------------------..........................
NTBGa-15(1,1)     --------------------------------------------------------------------------..........................
NTBGa-16(1,B)     --------------------------------------------------------------------------..........................
NTBGb(4,1)        --------------------------------------------------------------------------..........................
NTBGd(1,1)        --------------------------------------------------------------------------..........................
NTBGc(2,1)        --------------------------------------------------------------------------..........................
NTBGe(1,1)        --------------------------------------------------------------------------................T.C.......
P2aTBGa-1(10,2)   ------------------------------------------------------------------------------......................
P2aTBGa-2(3,1)    ------------------------------------------------------------------------------......................
P2aTBGa-3(1,1)    ------------------------------------------------------------------------------......................
P2aTBGb-1(2,2)    ----------------------------------------------------------------------------------------------------
P2aTBGb-2(2,1)    -------------------------------------------------------------------------------------.AC....C...A.GC
P2aTBGb-3(1,1)    ----------------------------------------------------------------------------------------------------
P2aTBGb-4(1,1)    ------------------------------------------------------------------------------.T..TCTCA.CGTGCACT.TTT
P2aTBGc-1(1,B)    -----------------------------------------------------------------------------CA..ACATG.C.G..C...T.G.
P2aTBGc-2(1,1)    TCTTCCCTTGGGATCGGCCATGGGATGTTCATCTGACCCTTCTCATCATGCGTTTCTTATTGGTGCCTTTTGCA...CA..ACATG.C.G..C...T.G.
P2aTBGc-3(1,1)    -----------------------------------------------------------------------------CA..ACATG.C.G..C...T.G.
P2aTBGc-4(1,1)    -----------------------------------------------------------------------------CA..ACATG.C.G..C...T.G.
P2aTBGc-6(1,1)    -----------------------------------------------------------------------------CA..ACATG.C.G..C...T.G.
P2aTBGc-5(1,B)    -----------------------------------------------------------------------------CA..ACATG.C.G..C...T.G.
15iTBGa-1(6,B)    -------------------------------------------TGTAAAAGTGGTGAGAAGGAATGTAAAGTTG...GC.G................A..
15iTBGa-2(4,2)    -------------------------------------------TGTAAAAGTGGTGAGAAGGAATGTAAAGTTG...GC.G................A..
15iTBGa-3(3,2)    -------------------------------------------TGTAAAAGTGGTGAGAAGGAATGTAAAGTTG...GC.G................A..
15iTBGa-4(3,B)    -------------------------------------------TGTAAAAGTGGTGAGAAGGAATGTAAAGTTG...GC.G................A..
15iTBGa-5(2,B)    -------------------------------------------TGTAAAAGTGGTGAGAAGGAATGTAAAGTTG...GC.G................A..
15iTBGa-6(2,1)    -------------------------------------------TGTAAAAGTGGTGAGAAGGAATGTAAAGTTG...GC.G................A..
15iTBGa-7(1,B)    -------------------------------------------TGTAAAAGTGGTGAGAAGGAATGTAAAGTTG...GC.G................A..
15iTBGa-8(1,1)    -------------------------------------------TGTAAAAGTGGTGAGAAGGAATGTAAAGTTG...GC.G................A..
15iTBGa-9(1,1)    -------------------------------------------TGTAAAAGTGGTGAGAAGGAATGTAAAGTTG...GC.G................A..
15iTBGb-1(4,2)    ----------------------------------------------------------------------GTTG...GC.....................
15iTBGb-2(2,2)    ----------------------------------------------------------------------GTTG...GC.....................
15iTBGb-4(1,1)    ----------------------------------------------------------------------GTTG...GC.....................
15iTBGb-5(1,B)    ----------------------------------------------------------------------GTTG...GC.....................
15iTBGb-7(1,1)    ----------------------------------------------------------------------GTTG...GC.....................
15iTBGc-1(3,1)    ----------------------------------------------------------------------GTTG..........................
6TBGa-1(10,1)     -------------------------------------------TGTAAAACTGGGACA------------------------------------------
6TBGa-2(9,1)      -------------------------------------------TGTAAAACTGGGACA------------------------------------------
6TBGa-3(3,1)      -------------------------------------------TGTAAAACTGGGACA------------------------------------------
6TBGa-4(2,1)      -------------------------------------------TGTAAAACTGGGACA------------------------------------------
6TBGa-5(1,1)      -------------------------------------------TGTAAAACTGGGACA------------------------------------------
6TBGb-1(7,B)      -------------------------------------------TCCTAATCTGAGGCTACACATGGCAGAACTG.T.GA---------------------
6TBGb-2(6,1)      -------------------------------------------TCCTAATCTGAGGCTACACATGGCAGAACTG.T.GA---------------------
6TBGb-3(2,1)      -------------------------------------------TCCTAATCTGAGGCTACACATGGCAGAACTG.T.GA---------------------
6TBGc-1(6,B)      -------------------------------------------TGTAAAAGTGGTGAGAAGGAATGTAAAGTTG...GC.G................A..
6TBGc-2(3,1)      -------------------------------------------TGTAAAAGTGGTGAGAAGGAATGTAAAGTTG...GC.G................A..
6TBGd(10,B)       ----------------------------------------------------------------------------------------------------
6TBGe(2,1)        -------------------------------------------TCCTAATCTGAGGCTACACATGGCAGAACTG.T.GA---------------------
```

```
                      3010      3020      3030      3040      3050      3060      3070      3080      3090      3100
                  ....|....|....|....|....|....|....|....|....|....|....|....|....|....|....|....|....|....|....|....|
NTBGa-1(35,2)     ACAAGCTAAAGAATCAGAGGAACAGAAATCGGAGCTGA--------------------------------------------------------------
NTBGa-2(8,1)      .................................A....--------------------------------------------------------------
NTBGa-3(6,B)      ......................................--------------------------------------------------------------
NTBGa-4(5,B)      ......................................--------------------------------------------------------------
NTBGa-6(2,1)      ......................................--------------------------------------------------------------
NTBGa-7(1,1)      C..................................C..--------------------------------------------------------------
NTBGa-8(1,1)      ......................................--------------------------------------------------------------
NTBGa-10(1,1)     ......................................--------------------------------------------------------------
NTBGa-11(1,1)     ......................................GTAAGTTGCAGTCACTGAAATGAGGGAATGTGGGGTCTTCCCAAAGTCCTGCGTATGGGATG
NTBGa-12(1,1)     ......................................GTAAGTTGCAGTCACTGAAATGAGGGAATGTGGGGTCTTCCCAAAGTCCTGCGTATGGGATG
NTBGa-13(1,1)     ......................................--------------------------------------------------------------
NTBGa-15(1,1)     ......................................--------------------------------------------------------------
NTBGa-16(1,B)     ......................................--------------------------------------------------------------
NTBGb(4,1)        ......................................--------------------------------------------------------------
NTBGd(1,1)        ......................................GTAAGTTGCAGTCACTGAAATGAGGGAATGTGGGGTCTTCCCAAAGTCCTGCGTATGGGATG
NTBGc(2,1)        ......................................--------------------------------------------------------------
NTBGe(1,1)        ...................A..................--------------------------------------------------------------
P2aTBGa-1(10,2)   ...................A..................------------------------------------------AGGAGCGCCATGAGGAGATG
P2aTBGa-2(3,1)    ...................A..................------------------------------------------AGGAGCGCCATGAGGAGATG
P2aTBGa-3(1,1)    ...................A..................------------------------------------------AGGAGCGCCATGAGGAGATG
P2aTBGb-1(2,2)    ----------------.T.AG.AG...TG.AAG.T..G------------------------------------------AGGCAGCAGCTGTAAAAGTG
P2aTBGb-2(2,1)    ...CTG.G..C.GATG.T.AG.AG...TG.AAG.T..G------------------------------------------AGGCAGCAGCTGTAAAAGTG
P2aTBGb-3(1,1)    ----------------.T.AG.AG...TG.AAG.T..G------------------------------------------AGGCAGCAGCTGTAAAAGTG
P2aTBGb-4(1,1)    CTGCTT.TCCTTTG...T.AG.AG...TG.AAG.T..G------------------------------------------AGGCAGCAGCTGTAAAAGTG
P2aTBGc-1(1,B)    T......G...C.GTG...A.T.G......A.......--------------------------------------------------------------
P2aTBGc-2(1,1)    T......G...C.GTG...A.T.G......A.......--------------------------------------------------------------
P2aTBGc-3(1,1)    T......G...C.GTG...A.T.G......A.......--------------------------------------------------------------
P2aTBGc-4(1,1)    T......G...C.GTG...A.T.G......A.......GTAAGTCGCAGTCACTGAACTGAGCGAATTTGGGGTCTTCCCAAGGGACAGCATACGGGATG
P2aTBGc-6(1,1)    T......G...C.GTG...A.T.G......A.......--------------------------------------------------------------
P2aTBGc-5(1,B)    T......G...C.GTG...A.T.G......A.......GTAAGTCGCAGTCACTGAACTGAGCGAATTTGGGGTCTTCCCAAGGGACAGCATACGGGATG
15iTBGa-1(6,B)    CA.................A..................--------------------------------------------------------------
15iTBGa-2(4,2)    CA.................A..................--------------------------------------------------------------
15iTBGa-3(3,2)    CA.................A..................--------------------------------------------------------------
15iTBGa-4(3,B)    CA.................A..................--------------------------------------------------------------
15iTBGa-5(2,B)    CA.................A..................--------------------------------------------------------------
15iTBGa-6(2,1)    CA.................A..................--------------------------------------------------------------
15iTBGa-7(1,B)    CA.................A..................--------------------------------------------------------------
15iTBGa-8(1,1)    CA.................A..................--------------------------------------------------------------
15iTBGa-9(1,1)    CA.................A..................--------------------------------------------------------------
15iTBGb-1(4,2)    ...................A...............C..--------------------------------------------------------------
15iTBGb-2(2,2)    ...................A...............C..--------------------------------------------------------------
15iTBGb-4(1,1)    ...................A...............C..--------------------------------------------------------------
15iTBGb-5(1,B)    ...................A...............C..--------------------------------------------------------------
15iTBGb-7(1,1)    ...................A...............C..--------------------------------------------------------------
15iTBGc-1(3,1)    ..............---------------------C.G--------------------------------------------------------------
6TBGa-1(10,1)     CA.................A..................--------------------------------------------------------------
6TBGa-2(9,1)      CA.................A..................--------------------------------------------------------------
6TBGa-3(3,1)      CA.................A..................--------------------------------------------------------------
6TBGa-4(2,1)      CA.................A..................--------------------------------------------------------------
6TBGa-5(1,1)      CA.................A..................--------------------------------------------------------------
6TBGb-1(7,B)      T....T.G...C.GTG...A.T.G......A...TG..--------------------------------------------------------------
6TBGb-2(6,1)      T....T.G...C.GTG...A.T.G......A...TG..--------------------------------------------------------------
6TBGb-3(2,1)      T....T.G...C.GTG...A.T.G......A...TG..--------------------------------------------------------------
6TBGc-1(6,B)      CA.................A..................--------------------------------------------------------------
6TBGc-2(3,1)      CA.................A..................--------------------------------------------------------------
6TBGd(10,B)       ----------------------------------------------------------------------------------------------------
6TBGe(2,1)        T....T.G...C.GTG...A.T.G......A...TG..--------------------------------------------------------------

                      3110      3120      3130      3140      3150      3160      3170      3180      3190      3200
                  ....|....|....|....|....|....|....|....|....|....|....|....|....|....|....|....|....|....|....|....|
NTBGa-1(35,2)     ---------------------------------------------AGGAGCACCATGAGGAGACG-----------------------------------
NTBGa-2(8,1)      ---------------------------------------------....................-----------------------------------
NTBGa-3(6,B)      ---------------------------------------------....................-----------------------------------
NTBGa-4(5,B)      ---------------------------------------------....................-----------------------------------
NTBGa-6(2,1)      ---------------------------------------------....................-----------------------------------
NTBGa-7(1,1)      ---------------------------------------------....................-----------------------------------
NTBGa-8(1,1)      ---------------------------------------------....................-----------------------------------
NTBGa-10(1,1)     ---------------------------------------------....................-----------------------------------
NTBGa-11(1,1)     AAAAATCCCCTCTGACCATGCACTGCTTTTCTCCTCCTTTGCCAG....................-----------------------------------
NTBGa-12(1,1)     AAAAATCCCCTCTGACCATGCACTGCTTTTCTCCTCCTTTGCCAG....................-----------------------------------
NTBGa-13(1,1)     ---------------------------------------------....................GGTGAGTCTCCCCTCCCATATTAAAATCGTTGGGG
NTBGa-15(1,1)     ---------------------------------------------....................-----------------------------------
NTBGa-16(1,B)     ---------------------------------------------....................-----------------------------------
NTBGb(4,1)        ---------------------------------------------....................-----------------------------------
NTBGd(1,1)        AAAAATCCCCTCTGACCATGCACTGCTTTTCTCCTCCTTTGCCAG....................-----------------------------------
NTBGc(2,1)        ---------------------------------------------....................-----------------------------------
NTBGe(1,1)        ----------------------------------------------------------------------------------------------------
P2aTBGa-1(10,2)   ----------------------------------------------------------------------------------------------------
P2aTBGa-2(3,1)    ----------------------------------------------------------------------------------------------------
P2aTBGa-3(1,1)    ----------------------------------------------------------------------------------------------------
P2aTBGb-1(2,2)    ----------------------------------------------------------------------------------------------------
P2aTBGb-2(2,1)    ----------------------------------------------------------------------------------------------------
P2aTBGb-3(1,1)    ----------------------------------------------------------------------------------------------------
P2aTBGb-4(1,1)    ----------------------------------------------------------------------------------------------------
P2aTBGc-1(1,B)    ----------------------------------------------------------------------------------------------------
P2aTBGc-2(1,1)    ----------------------------------------------------------------------------------------------------
P2aTBGc-3(1,1)    ----------------------------------------------------------------------------------------------------
P2aTBGc-4(1,1)    AAAAATCCCCTCTGACCATGCGCTGATTTTGTCTTTCTATTCC---------------------------------------------------------
P2aTBGc-6(1,1)    ----------------------------------------------------------------------------------------------------
P2aTBGc-5(1,B)    AAAAATCCCCTCTGACCATGCGCTGATTTTGTCTTTCTATTCC---------------------------------------------------------
15iTBGa-1(6,B)    ---------------------------------------------......G...........T.-----------------------------------
15iTBGa-2(4,2)    ---------------------------------------------......G...........T.-----------------------------------
15iTBGa-3(3,2)    ---------------------------------------------......G...........T.-----------------------------------
15iTBGa-4(3,B)    ---------------------------------------------......G...........T.-----------------------------------
15iTBGa-5(2,B)    ---------------------------------------------......G...........T.-----------------------------------
15iTBGa-6(2,1)    ---------------------------------------------......G...........T.-----------------------------------
15iTBGa-7(1,B)    ---------------------------------------------......G...........T.-----------------------------------
15iTBGa-8(1,1)    ---------------------------------------------......G...........T.-----------------------------------
15iTBGa-9(1,1)    ---------------------------------------------......G...........T.-----------------------------------
15iTBGb-1(4,2)    ---------------------------------------------......G...........T.-----------------------------------
15iTBGb-2(2,2)    ---------------------------------------------......G...........T.-----------------------------------
15iTBGb-4(1,1)    ---------------------------------------------......G...........T.-----------------------------------
15iTBGb-5(1,B)    ---------------------------------------------......G...........T.-----------------------------------
15iTBGb-7(1,1)    ---------------------------------------------......G...........T.-----------------------------------
15iTBGc-1(3,1)    ---------------------------------------------...G..G...........T.-----------------------------------
6TBGa-1(10,1)     ----------------------------------------------------------------------------------------------------
6TBGa-2(9,1)      ----------------------------------------------------------------------------------------------------
6TBGa-3(3,1)      ----------------------------------------------------------------------------------------------------
6TBGa-4(2,1)      ----------------------------------------------------------------------------------------------------
6TBGa-5(1,1)      ----------------------------------------------------------------------------------------------------
6TBGb-1(7,B)      ----------------------------------------------------------------------------------------------------
6TBGb-2(6,1)      ----------------------------------------------------------------------------------------------------
6TBGb-3(2,1)      ----------------------------------------------------------------------------------------------------
6TBGc-1(6,B)      ----------------------------------------------------------------------------------------------------
6TBGc-2(3,1)      ----------------------------------------------------------------------------------------------------
6TBGd(10,B)       ----------------------------------------------------------------------------------------------------
6TBGe(2,1)        ----------------------------------------------------------------------------------------------------
```

```
                           3210      3220      3230      3240      3250      3260      3270      3280      3290      3300
                      ....|....|....|....|....|....|....|....|....|....|....|....|....|....|....|....|....|....|....|....|
NTBGa-1(35,2)         -------------------------------------------------------------GGGCAACAAGCTAAAGAAT-CAGAGAAACAGAAATCGGA
NTBGa-2(8,1)          -------------------------------------------------------------...................-...................
NTBGa-3(6,B)          -------------------------------------------------------------...................-...................
NTBGa-4(5,B)          -------------------------------------------------------------...................-...................
NTBGa-6(2,1)          -------------------------------------------------------------...................-...................
NTBGa-7(1,1)          -------------------------------------------------------------...................-...................
NTBGa-8(1,1)          -------------------------------------------------------------...................-...................
NTBGa-10(1,1)         -------------------------------------------------------------...................-...................
NTBGa-11(1,1)         -------------------------------------------------------------...................-...................
NTBGa-12(1,1)         -------------------------------------------------------------...................-...................
NTBGa-13(1,1)         TCTTCCTGTGTGAGCTGTGGGATGAGATGTTCCTCTCATCACGCATTGTTTTTCTTTTCCA...................-...................
NTBGa-15(1,1)         -------------------------------------------------------------...................-...................
NTBGa-16(1,B)         -------------------------------------------------------------...................-...................
NTBGb(4,1)            -------------------------------------------------------------...................-...................
NTBGd(1,1)            -------------------------------------------------------------...................-...................
NTBGc(2,1)            -------------------------------------------------------------...................-...................
NTBGe(1,1)            ----------------------------------------------------------------------------------------------------
P2aTBGa-1(10,2)       -------------------------------------------------------------...................-...................
P2aTBGa-2(3,1)        -------------------------------------------------------------...................-...................
P2aTBGa-3(1,1)        -------------------------------------------------------------...................-...................
P2aTBGb-1(2,2)        -------------------------------------------------------------..A..CA............-...................
P2aTBGb-2(2,1)        -------------------------------------------------------------..A..CA............-...................
P2aTBGb-3(1,1)        -------------------------------------------------------------..A..CA............-...................
P2aTBGb-4(1,1)        -------------------------------------------------------------..A..CA............-...................
P2aTBGc-1(1,B)        ---------------------------------------------------------------AG.GAT.TT.G.CA...AT..GTTT..GTGCTG.A..
P2aTBGc-2(1,1)        ---------------------------------------------------------------AG.GAT.TT.G.CA...AT..GTTT..GTGCTG.A..
P2aTBGc-3(1,1)        ---------------------------------------------------------------AG.GAT.TT.G.CA...AT..GTTT..GTGCTG.A..
P2aTBGc-4(1,1)        -------------------------------------------------------------A.AG.GAT.TT.G.CA...AT..GTTT..GTGCTG.A..
P2aTBGc-6(1,1)        ---------------------------------------------------------------AG.GAT.TT.G.CA...AT..GTTT..GTGCTG.A..
P2aTBGc-5(1,B)        -------------------------------------------------------------A.AG.GAT.TT.G.CA...AT..GTTT..GTGCTG.A..
15iTBGa-1(6,B)        -------------------------------------------------------------...................-...................
15iTBGa-2(4,2)        -------------------------------------------------------------...................-...................
15iTBGa-3(3,2)        -------------------------------------------------------------...................-...................
15iTBGa-4(3,B)        -------------------------------------------------------------...................-...................
15iTBGa-5(2,B)        -------------------------------------------------------------...................-...................
15iTBGa-6(2,1)        -------------------------------------------------------------...................-...................
15iTBGa-7(1,B)        -------------------------------------------------------------...................-...................
15iTBGa-8(1,1)        -------------------------------------------------------------...................-...................
15iTBGa-9(1,1)        -------------------------------------------------------------...................-...................
15iTBGb-1(4,2)        -------------------------------------------------------------.CAG.....T..G...C.G-TG.----------------
15iTBGb-2(2,2)        -------------------------------------------------------------.CAG.....T..G...C.G-TG.GTG.GTCTTTG..CCC
15iTBGb-4(1,1)        -------------------------------------------------------------.CAG.....T..G...C.G-TG.GTG.GTCTTTG..CCC
15iTBGb-5(1,B)        -------------------------------------------------------------.CAG.....T..G...C.G-TG.----------------
15iTBGb-7(1,1)        -------------------------------------------------------------.CAG.....T..G...C.G-TG.----------------
15iTBGc-1(3,1)        -------------------------------------------------------------.CAG.....A..G...C.G-TG-----------------
6TBGa-1(10,1)         ----------------------------------------------------------------------------------------------------
6TBGa-2(9,1)          ----------------------------------------------------------------------------------------------------
6TBGa-3(3,1)          ----------------------------------------------------------------------------------------------------
6TBGa-4(2,1)          ----------------------------------------------------------------------------------------------------
6TBGa-5(1,1)          -----------------------------------------GTAAGTTGCAGTCACTGAACT.AGGGA.T.GAGGGTCT.-.CC.A.GT.CTGCG.AT.G
6TBGb-1(7,B)          ----------------------------------------------------------------------------------------------------
6TBGb-2(6,1)          ----------------------------------------------------------------------------------------------------
6TBGb-3(2,1)          ----------------------------------------------------------------------------------------------------
6TBGc-1(6,B)          -----------------------------------------AGGAGCGCCATGAGGAGATG...................-...................
6TBGc-2(3,1)          -----------------------------------------AGGAGCGCCATGAGGAGATG...................-...................
6TBGd(10,B)           ----------------------------------------------------------------------------------------------------
6TBGe(2,1)            ----------------------------------------------------------------------------------------------------

                           3310      3320      3330      3340      3350      3360      3370      3380      3390      3400
                      ....|....|....|....|....|....|....|....|....|....|....|....|....|....|....|....|....|....|....|....|
NTBGa-1(35,2)         GCTG------------------------------------------------------------------------------------------------
NTBGa-2(8,1)          ....------------------------------------------------------------------------------------------------
NTBGa-3(6,B)          ....------------------------------------------------------------------------------------------------
NTBGa-4(5,B)          ....------------------------------------------------------------------------------------------------
NTBGa-6(2,1)          ....------------------------------------------------------------------------------------------------
NTBGa-7(1,1)          ....------------------------------------------------------------------------------------------------
NTBGa-8(1,1)          ....------------------------------------------------------------------------------------------------
NTBGa-10(1,1)         ....------------------------------------------------------------------------------------------------
NTBGa-11(1,1)         ....------------------------------------------------------------------------------------------------
NTBGa-12(1,1)         ....------------------------------------------------------------------------------------------------
NTBGa-13(1,1)         ....------------------------------------------------------------------------------------------------
NTBGa-15(1,1)         ....------------------------------------------------------------------------------------------------
NTBGa-16(1,B)         ....------------------------------------------------------------------------------------------------
NTBGb(4,1)            ....------------------------------------------------------------------------------------------------
NTBGd(1,1)            ....------------------------------------------------------------------------------------------------
NTBGc(2,1)            ....------------------------------------------------------------------------------------------------
NTBGe(1,1)            ----------------------------------------------------------------------------------------------------
P2aTBGa-1(10,2)       ....------------------------------------------------------------------------------------------------
P2aTBGa-2(3,1)        ....------------------------------------------------------------------------------------------------
P2aTBGa-3(1,1)        ....------------------------------------------------------------------------------------------------
P2aTBGb-1(2,2)        ....------------------------------------------------------------------------------------------------
P2aTBGb-2(2,1)        ....------------------------------------------------------------------------------------------------
P2aTBGb-3(1,1)        ....------------------------------------------------------------------------------------------------
P2aTBGb-4(1,1)        ....------------------------------------------------------------------------------------------------
P2aTBGc-1(1,B)        ....------------------------------------------------------------------------------------------------
P2aTBGc-2(1,1)        ....------------------------------------------------------------------------------------------------
P2aTBGc-3(1,1)        ....------------------------------------------------------------------------------------------------
P2aTBGc-4(1,1)        ....------------------------------------------------------------------------------------------------
P2aTBGc-6(1,1)        ....------------------------------------------------------------------------------------------------
P2aTBGc-5(1,B)        ....------------------------------------------------------------------------------------------------
15iTBGa-1(6,B)        ....------------------------------------------------------------------------------------------------
15iTBGa-2(4,2)        ....------------------------------------------------------------------------------------------------
15iTBGa-3(3,2)        ....------------------------------------------------------------------------------------------------
15iTBGa-4(3,B)        ....------------------------------------------------------------------------------------------------
15iTBGa-5(2,B)        ....------------------------------------------------------------------------------------------------
15iTBGa-6(2,1)        ....------------------------------------------------------------------------------------------------
15iTBGa-7(1,B)        ....------------------------------------------------------------------------------------------------
15iTBGa-8(1,1)        ....------------------------------------------------------------------------------------------------
15iTBGa-9(1,1)        ....------------------------------------------------------------------------------------------------
15iTBGb-1(4,2)        ----------------------------------------------------------------------------------------------------
15iTBGb-2(2,2)        AAACCAAAGCAATATGGGGCAATCCATGGGATG-------------------------------------------------------------------
15iTBGb-4(1,1)        AAACCAAAGCAATATGGGGCAATCCATGGGATG-------------------------------------------------------------------
15iTBGb-5(1,B)        ----------------------------------------------------------------------------------------------------
15iTBGb-7(1,1)        ----------------------------------------------------------------------------------------------------
15iTBGc-1(3,1)        ----------------------------------------------------------------------------------------------------
6TBGa-1(10,1)         ----------------------------------------------------------------------------------------------------
6TBGa-2(9,1)          ----------------------------------------------------------------------------------------------------
6TBGa-3(3,1)          ----------------------------------------------------------------------------------------------------
6TBGa-4(2,1)          ----------------------------------------------------------------------------------------------------
6TBGa-5(1,1)          .A..A---------------------------------------------------------------AAAATCCCCTCTGACCATGCACTGCTTTTCTC
6TBGb-1(7,B)          ----------------------------------------------------AGAGATATTTGACAAATATAGGTTTACGTGC-------TGCAGAGCT-
6TBGb-2(6,1)          ----------------------------------------------------AGAGATATTTGACAAATATAGGTTTACGTGC-------TGCAGAGCT-
6TBGb-3(2,1)          ----------------------------------------------------AGAGATATTTGACAAATATAGGTTTACGTGC-------TGCAGAGCT-
6TBGc-1(6,B)          ....A-----------------------------------------------------------------------------------------------
6TBGc-2(3,1)          ....AGTAAGTTGCAGTCACTGAACTGAGGGTATTTGGGGTCCTTTCAAGGGACTGTGTATGGGATGAAAAATCCCCTCTGACCATGCACTGCTTTTCTC
6TBGd(10,B)           ----------------------------------------------------------------------------------------------------
6TBGe(2,1)            ----------------------------------------------------AGAGATATTTGACAAATATAGGTTTACGTGC-------TGCAGAGCT-
```

```
                          3410      3420      3430      3440      3450      3460      3470      3480      3490      3500
                    ....|....|....|....|....|....|....|....|....|....|....|....|....|....|....|....|....|....|....|....|
NTBGa-1(35,2)       -----------AAGGAGCGCCA------TGAGGAGATGGCAGAACAAACTGAAGCAGTGG------------------------TGGTAGAAACTGAAGA
NTBGa-2(8,1)        -----------...........------................................------------------------................
NTBGa-3(6,B)        -----------...........------................................------------------------................
NTBGa-4(5,B)        -----------...........------...........---..................------------------------................
NTBGa-6(2,1)        -----------...........------...........---..................------------------------................
NTBGa-7(1,1)        -----------...........------................................------------------------................
NTBGa-8(1,1)        -----------...........------................................------------------------................
NTBGa-10(1,1)       -----------...........------................................------------------------................
NTBGa-11(1,1)       -----------...........------................................------------------------................
NTBGa-12(1,1)       -----------...........------................................------------------------................
NTBGa-13(1,1)       -----------...........------...........---..................------------------------................
NTBGa-15(1,1)       -----------...........------...........---..................------------------------................
NTBGa-16(1,B)       -----------...........------................................------------------------................
NTBGb(4,1)          -----------...........------................................------------------------................
NTBGd(1,1)          -----------...........------...........---..................------------------------................
NTBGc(2,1)          -----------...........------................................------------------------................
NTBGe(1,1)          ------------..........------...........---..................------------------------................
P2aTBGa-1(10,2)     -----------...........------................................------------------------................
P2aTBGa-2(3,1)      -----------...........------................................----------------------------------------
P2aTBGa-3(1,1)      -----------...........------...........---..................------------------------................
P2aTBGb-1(2,2)      -----------...........------................................------------------------................
P2aTBGb-2(2,1)      -----------...........------................................------------------------................
P2aTBGb-3(1,1)      -----------...........------................................-----------------------TG...............
P2aTBGb-4(1,1)      -----------...........------................................------------------------................
P2aTBGc-1(1,B)      -----------..AA.ATA.GT------..CA..AC..AAGA...G..........T...------------------------------.....A....
P2aTBGc-2(1,1)      -----------..AA.ATA.GT------..CA..AC..AAGA...G..........T...----------------------------------------
P2aTBGc-3(1,1)      -----------..AA.ATA.GT------..CA..AC..AAGA...G..........T...------------------------------.....A....
P2aTBGc-4(1,1)      -----------..AA.ATA.GT------..CA..AC..AAGA...G..........T...------------------------------.....A....
P2aTBGc-6(1,1)      -----------..AA.ATA.GT------..CA..AC..AAGA...G..........T...------------------------------.....A....
P2aTBGc-5(1,B)      -----------..AA.ATA.GT------..CA..AC..AAGA...G..........T...------------------------------.....A....
15iTBGa-1(6,B)      -----------.......A...------................................------------------------................
15iTBGa-2(4,2)      -----------.......A...------................................------------------------................
15iTBGa-3(3,2)      -----------.......A...------...........---..................------------------------................
15iTBGa-4(3,B)      -----------.......A...------...........---..................------------------------................
15iTBGa-5(2,B)      -----------.......A...------................................------------------------................
15iTBGa-6(2,1)      -----------.......A...------...........---..................------------------------................
15iTBGa-7(1,B)      -----------.......A...------...........---..................------------------------................
15iTBGa-8(1,1)      -----------.......A...------................................------------------------................
15iTBGa-9(1,1)      -----------.......A...------................................------------------------................
15iTBGb-1(4,2)      ------------------------------------------------------------------------------------.......T.A......
15iTBGb-2(2,2)      -----ACAAGCGTCCCATCT..------.TGT.C.T..CTCTTCT.TT.CTTCTTTCCA.------------------------.......T.A......
15iTBGb-4(1,1)      -----ACAAGCGTCCCATCT..------.TGT.C.T..CTCTTCT.TT.CTTCTTTCCA.------------------------.......T.A......
15iTBGb-5(1,B)      ------------------------------------------------------------------------------------.......T.A......
15iTBGb-7(1,1)      ------------------------------------------------------------------------------------.......T.A......
15iTBGc-1(3,1)      -----------------------------------------------------------------------------------G...G....G.......
6TBGa-1(10,1)       ------------..........------................................------------------------................
6TBGa-2(9,1)        ------------..........------................................------------------------................
6TBGa-3(3,1)        ------------..........------................................------------------------................
6TBGa-4(2,1)        ------------..........------...........---..................------------------------................
6TBGa-5(1,1)        TTCCTTTGCCAG..........------................................------------------------................
6TBGb-1(7,B)        -------GAAA..AA.-..TTGCAGAAC...A..A.C.AA--------------TT.GA.------------------------CAT.G......A....
6TBGb-2(6,1)        -------AAAA..AA.-..TTGCAGAAC...A..A.C.AA--------------TT.GA.------------------------CAT.G......A....
6TBGb-3(2,1)        -------GAAA..AA.A..TTGCAGAAC...A..A.C.AA--------------TT.GA.------------------------CAT.G......A....
6TBGc-1(6,B)        ------------..........------................................------------------------................
6TBGc-2(3,1)        CTTCTTTGCCAG..........------................................------------------------................
6TBGd(10,B)         ----------------------------------------------------------------------------------------------------
6TBGe(2,1)          -------GAAA..AA.-..TTGCAGAAC...A..A.C.AA--------------TT.GA.------------------------CAT.G......A....

                          3510      3520      3530      3540      3550      3560      3570      3580      3590      3600
                    ....|....|....|....|....|....|....|....|....|....|....|....|....|....|....|....|....|....|....|....|
NTBGa-1(35,2)       ATAGG-----------------------------------------------------------------------------------------------
NTBGa-2(8,1)        .....-----------------------------------------------------------------------------------------------
NTBGa-3(6,B)        .....GTGAGTCTTTCCCAAACCAAAGCAATACGGGGTTTCCCATGGCATGACAAGCTGTCCCACCTCAGCATCCGTTCCTTTTTCTTTCTTTTCCAG--
NTBGa-4(5,B)        .....-----------------------------------------------------------------------------------------------
NTBGa-6(2,1)        .....GTGAGTCTTTCCCAAACCAAAGCAATACGGGGTTTCCCATGGCATGACAAGCTGTCCCACCTCAGCATCCGTTCCTTTTTCTTTCTTTTCCAG--
NTBGa-7(1,1)        .....-----------------------------------------------------------------------------------------------
NTBGa-8(1,1)        .....-----------------------------------------------------------------------------------------------
NTBGa-10(1,1)       .....-----------------------------------------------------------------------------------------------
NTBGa-11(1,1)       .....-----------------------------------------------------------------------------------------------
NTBGa-12(1,1)       .....-----------------------------------------------------------------------------------------------
NTBGa-13(1,1)       .....-----------------------------------------------------------------------------------------------
NTBGa-15(1,1)       .....GTGAGTCTTTCCCAAACCAAAGCAATACGGGGTTTCCCATGGCATGACAAGCTGTCCCACCTCAGCATCCGTTCCTTTTTCTTTCTTTTCCAG--
NTBGa-16(1,B)       .....-----------------------------------------------------------------------------------------------
NTBGb(4,1)          .....-----------------------------------------------------------------------------------------------
NTBGd(1,1)          .....-----------------------------------------------------------------------------------------------
NTBGc(2,1)          .....-----------------------------------------------------------------------------------------------
NTBGe(1,1)          .....-----------------------------------------------------------------------------------------------
P2aTBGa-1(10,2)     T....-----------------------------------------------------------------------------------------------
P2aTBGa-2(3,1)      ----------------------------------------------------------------------------------------------------
P2aTBGa-3(1,1)      T....-----------------------------------------------------------------------------------------------
P2aTBGb-1(2,2)      .GC..-----------------------------------------------------------------------------------------------
P2aTBGb-2(2,1)      .GC..-----------------------------------------------------------------------------------------------
P2aTBGb-3(1,1)      .GC..-----------------------------------------------------------------------------------------------
P2aTBGb-4(1,1)      .GC..-----------------------------------------------------------------------------------------------
P2aTBGc-1(1,B)      ..C..-----------------------------------------------------------------------------------------------
P2aTBGc-2(1,1)      ----------------------------------------------------------------------------------------------------
P2aTBGc-3(1,1)      ..C..-----------------------------------------------------------------------------------------------
P2aTBGc-4(1,1)      ..C..-----------------------------------------------------------------------------------------------
P2aTBGc-6(1,1)      ..C..-----------------------------------------------------------------------------------------------
P2aTBGc-5(1,B)      ..C..-----------------------------------------------------------------------------------------------
15iTBGa-1(6,B)      ..C..-----------------------------------------------------------------------------------------------
15iTBGa-2(4,2)      ..C..GTGAGTCTTTCCCAAACCAAAGCAATACGGGGTTT-CCCATGGCATGACAAGCTGTCCCACCTCAGCATCCGTTGCTTTTTATTTCTTTTCCAG-
15iTBGa-3(3,2)      ..C..GTGAGTCTTTCCCAAACCAAAGCAATACGGGGTTT-CCCATGGCATGACAAGCTGTCCCACCTCAGCATCCGTTGCTTTTTATTTCTTTTCCAC-
15iTBGa-4(3,B)      ..C..GTGAGTCTTTCCCAAACCAAAGCAATACGGGGTTT-CCCATGGCATGACAAGCTGTCCCACCTCAGCATCCGTTGCTTTTTATTTCTTTTCCAG-
15iTBGa-5(2,B)      ..C..-----------------------------------------------------------------------------------------------
15iTBGa-6(2,1)      ..C..-----------------------------------------------------------------------------------------------
15iTBGa-7(1,B)      ..C..-----------------------------------------------------------------------------------------------
15iTBGa-8(1,1)      ..C..-----------------------------------------------------------------------------------------------
15iTBGa-9(1,1)      ..C..-----------------------------------------------------------------------------------------------
15iTBGb-1(4,2)      ..C..-----------------------------------------------------------------------------------------------
15iTBGb-2(2,2)      ..C..GTGAGTCTTCCCCAAACCAAAGCAATACGGGGTTT-CCCATGGCATGACAAGCTGTCCCACCTCAGCACCCGTTGCTTTTC-TTTCTTTTCCAG-
15iTBGb-4(1,1)      ..C..GTGAGTCTTCCCCAAACCAAAGCAATACGGGGTTT-CCCATGGCATGACAAGCTGTCCCACCTCAGCACCCGTTGCTTTTC-TTTCTTTTCCAG-
15iTBGb-5(1,B)      ..C..GTGAGTCTTCCCCAAACCAAAGCAATACGGGGTTTTCCCATGGCATGACAAGCTGTCCCACCTCAGCACCCGTTGCTTTTC-TTTCTTTTCCAG-
15iTBGb-7(1,1)      ..C..GTGAGTCTTCCCCAAACCAAAGCAATACGGGGTTT-CCCATGGCATGACAAGCTGTCCCACCTCAGCACCCGTTGCTTTTC-TTTCTTTTCCAG-
15iTBGc-1(3,1)      ..T..GTGAGTCTTTCCCAAACCAAAGCAATACAGGGTTT-CCCAAGGCATGACAAGCTGTCCCACCTCAGCGTCCGTTGCTTTTC-TTTCTTTTCCAG-
6TBGa-1(10,1)       .....-----------------------------------------------------------------------------------------------
6TBGa-2(9,1)        .....-----------------------------------------------------------------------------------------------
6TBGa-3(3,1)        .....GTGAGTCTTTCCCAAACCAAAGCAATACGGGGTTT-CCCATGGCATGACAAGCTGTCCCACCTCAGCATCCGTTCCTTTT-TATTTCTTTTCCAG
6TBGa-4(2,1)        .....-----------------------------------------------------------------------------------------------
6TBGa-5(1,1)        .....-----------------------------------------------------------------------------------------------
6TBGb-1(7,B)        ..T..-----------------------------------------------------------------------------------------------
6TBGb-2(6,1)        ..T..GTGAGTCTTCCCCAAACCAAAGCAATACGGGGTTT-CCCATGGCATGACAAGCTGTCCCACCTCAGCATCCGTTGCTTTTCTCTTTCTTTTCCAG
6TBGb-3(2,1)        ..T..-----------------------------------------------------------------------------------------------
6TBGc-1(6,B)        .....GTGAGTCTTTCCCAAACCAAAGCAATACAGGGTTT-CCCATGGGATGACAAGCTGTCCCACCTCAGCATCCGTTCCTTTT-TATTTCTTTTCCAG
6TBGc-2(3,1)        .....GTGAGTCTTTCCCAAACCAAAGCAATACAGGGTTT-CCCATGGGATGACAAGCTGTCCCACCTCAGCATCCGTTCCTTTT-TATTTCTTTTCCAG
6TBGd(10,B)         ----------------------------------------------------------------------------------------------------
6TBGe(2,1)          ..T..-----------------------------------------------------------------------------------------------
```

```
                           3610      3620      3630      3640      3650      3660      3670      3680      3690      3700
                  ....|....|....|....|....|....|....|....|....|....|....|....|....|....|....|....|....|....|....|....|
NTBGa-1(35,2)     AAAAACCATCTGAAGAATTGGATTGAGAGATGAACTGCGCCTCGCAGTAACCACAGGAGTTAAGCTTCATAGATCAATAACTGCACAGCATACAAAA-CC
NTBGa-2(8,1)      .................................................................................................-..
NTBGa-3(6,B)      .................................................................................................-..
NTBGa-4(5,B)      .................................................................................................-..
NTBGa-6(2,1)      .................................................................................................-..
NTBGa-7(1,1)      .................................................................................................-..
NTBGa-8(1,1)      .................................................................................................-..
NTBGa-10(1,1)     .................................................................................................-..
NTBGa-11(1,1)     .................................................................................................-..
NTBGa-12(1,1)     .................................................................................................-..
NTBGa-13(1,1)     .................................................................................................-..
NTBGa-15(1,1)     .................................................................................................-..
NTBGa-16(1,B)     .................................................................................................-..
NTBGb(4,1)        .................................................................................................-..
NTBGd(1,1)        .................................................................................................-..
NTBGc(2,1)        .................................................................................................-..
NTBGe(1,1)        .................................................................................................-..
P2aTBGa-1(10,2)   .........G........CA..........................A...G..............................................-..
P2aTBGa-2(3,1)    .........G........CA..........................A...G..............................................-..
P2aTBGa-3(1,1)    .........G........CA..........................A...G..............................................-..
P2aTBGb-1(2,2)    ..................CA.......................A...........................C..........A.........A....-..
P2aTBGb-2(2,1)    ..................CA.......................A...........................C..........A.........A....-..
P2aTBGb-3(1,1)    ..................CA.......................A...........................C..........A.........A....-..
P2aTBGb-4(1,1)    ..................CA.......................A...........................C..........A.........A....-..
P2aTBGc-1(1,B)    ..C........A.....CA........................A.............................CTGC.G.T.......G...GC..CAT.
P2aTBGc-2(1,1)    ..C........A.....CA........................A.............................CTGC.G.T.......G...GC..CAT.
P2aTBGc-3(1,1)    ..C........A.....CA........................A.............................CTGC.G.T.......G...GC..CAT.
P2aTBGc-4(1,1)    ..C........A.....CA........................A.............................CTGC.G.T.......G...GC..CAT.
P2aTBGc-6(1,1)    ..C........A.....CA........................A.............................CTGC.G.T.......G...GC..CAT.
P2aTBGc-5(1,B)    ..C........A.....CA........................A.............................CTGC.G.T.......G...GC..CAT.
15iTBGa-1(6,B)    ..................CA.......................A..A...G..............................................-..
15iTBGa-2(4,2)    ..................CA.......................A..A...G..............................................-..
15iTBGa-3(3,2)    ..................CA.......................A..A...G..............................................-..
15iTBGa-4(3,B)    ..................CA.......................A..A...G..............................................-..
15iTBGa-5(2,B)    ..................CA.......................A..A...G..............................................-..
15iTBGa-6(2,1)    ..................CA.......................A..A...G..............................................-..
15iTBGa-7(1,B)    ..................CA.......................A..A...G..............................................-..
15iTBGa-8(1,1)    ..................CA.......................A..A...G..............................................-..
15iTBGa-9(1,1)    ..................CA.......................A..A...G..............................................-..
15iTBGb-1(4,2)    .GG........T......G........................A...........................C..........A.........A....-..
15iTBGb-2(2,2)    .GG........T......G........................A...........................C..........A.........A....-..
15iTBGb-4(1,1)    .GG........T......G........................A...........................C..........A.........A....-..
15iTBGb-5(1,B)    .GG........T......G........................A...........................C..........A.........A....-..
15iTBGb-7(1,1)    .GG........T......G........................A...........................C..........A.........A....-..
15iTBGc-1(3,1)    ........G.........G........................A...............A.............CTGC....A......G...GT..CA..
6TBGa-1(10,1)     ..................CA.......................A..A...G.................T............................-..
6TBGa-2(9,1)      ..................CA.......................A..A...G.................T............................-..
6TBGa-3(3,1)      ..................CA.......................A..A...G.................T............................-..
6TBGa-4(2,1)      ..................CA.......................A..A...G.................T............................-..
6TBGa-5(1,1)      ..................CA.......................A..A...G.................T............................-..
6TBGb-1(7,B)      ...........A.....CA........................A...............A.............CTGC.G.T.......G...GC..CAT.
6TBGb-2(6,1)      ...........A.....CA........................A...............A.............CTGC.G.T.......G...GC..CAT.
6TBGb-3(2,1)      ...........A.....CA........................A...............A.............CTGC.G.T.......G...GC..CAT.
6TBGc-1(6,B)      ..................CA..........................A...G..............................................-..
6TBGc-2(3,1)      ..................CA..........................A...G..............................................-..
6TBGd(10,B)       ----------------------------------------------------------------------------------------------------
6TBGe(2,1)        ...........A.....CA........................A...............A.............CTG-CTGA.TGCACAGGATAGC..CAT

                           3710      3720      3730      3740      3750      3760      3770      3780      3790      3800
                  ....|....|....|....|....|....|....|....|....|....|....|....|....|....|....|....|....|....|....|....|
NTBGa-1(35,2)     ACAATAACTCAAACAGGGTAAGGA--------------------GGAGCCAGTGTTTGTGTTGAGTGAGAACACTGCAGTTCTGTCAGCCAAAGCTGCCT
NTBGa-2(8,1)      ........................--------------------........................................................
NTBGa-3(6,B)      ........................--------------------........................................................
NTBGa-4(5,B)      ........................--------------------........................................................
NTBGa-6(2,1)      ........................--------------------........................................................
NTBGa-7(1,1)      ........................--------------------........................................................
NTBGa-8(1,1)      ........................--------------------........................................................
NTBGa-10(1,1)     ........................--------------------........................................................
NTBGa-11(1,1)     ........................--------------------........................................................
NTBGa-12(1,1)     ........................--------------------........................................................
NTBGa-13(1,1)     ........................--------------------........................................................
NTBGa-15(1,1)     ...........-----------------------------------------------------------------------------------------
NTBGa-16(1,B)     ........................--------------------........................................................
NTBGb(4,1)        ........................--------------------........................................................
NTBGd(1,1)        ........................--------------------........................................................
NTBGc(2,1)        ........................--------------------........................................................
NTBGe(1,1)        ........................--------------------........................................................
P2aTBGa-1(10,2)   ........................--------------------......................................C..G..............
P2aTBGa-2(3,1)    ........................--------------------......................................C..G..............
P2aTBGa-3(1,1)    ........................--------------------......................................C..G..............
P2aTBGb-1(2,2)    ..G..TC.A.......A.C.....AATCCAGAGCGAGAAAAAGA........................................................
P2aTBGb-2(2,1)    ..G..TC.A.......A.C.....AATCCAGAGCGAGAAAAAGA........................................................
P2aTBGb-3(1,1)    ..G..TC.A.......A.C.....AATCCAGAGCGAGAAAAAGA........................................................
P2aTBGb-4(1,1)    ..G..TC.A.......A.C.....AATCCAGAGCGAGAAAAAGA........................................................
P2aTBGc-1(1,B)    G.C.....G....GCAA.C.....AATCCACACGGGGAACAAGA..........CC...A........................CA.....C........
P2aTBGc-2(1,1)    G.C.....G....GCAA.C.....AATCCACACGGGGAACAAGA..........CC...A........................CA.....C........
P2aTBGc-3(1,1)    G.C.....G....GCAA.C.....AATCCACACGGGGAACAAGA..........CC...A........................CA.....C........
P2aTBGc-4(1,1)    G.C.....G....GCAA.C.....AATCCACACGGGGAACAAGA..........CC...A........................CA.....C........
P2aTBGc-6(1,1)    G.C.....G....GCAA.C.....AATCCACACGGGGAACAAGA..........CC...A........................CA.....C........
P2aTBGc-5(1,B)    G.C.....G....GCAA.C.....AATCCACACGGGGAACAAGA..........CC...A........................CA.....C........
15iTBGa-1(6,B)    ................A.C.....--------------------...............A........................................
15iTBGa-2(4,2)    ................A.C.....--------------------...............A........................................
15iTBGa-3(3,2)    ................A.C.....--------------------...............A........................................
15iTBGa-4(3,B)    ................A.C.....--------------------...............A........................................
15iTBGa-5(2,B)    ................A.C.....--------------------...............A........................................
15iTBGa-6(2,1)    ................A.C.....--------------------...............A........................................
15iTBGa-7(1,B)    ................A.C.....--------------------...............A........................................
15iTBGa-8(1,1)    ................A.C.....--------------------...............A........................................
15iTBGa-9(1,1)    ................A.C.....--------------------...............A........................................
15iTBGb-1(4,2)    ..G..TC.A.......A.C.....AATCCACAGTGAGAACAAGA.............................G..........................
15iTBGb-2(2,2)    ..G..TC.A.......A.C.....AATCCACAGTGAGAACAAGA.............................G..........................
15iTBGb-4(1,1)    ..G..TC.A.......A.C.....AATCCACAGTGAGAACAAGA.............................G..........................
15iTBGb-5(1,B)    ..G..TC.A.......A.C.....AATCCACAGTGAGAACAAGA.............................G..........................
15iTBGb-7(1,1)    ..G..TC.A.......A.C.....AATCCACAGTGAGAACAAGA.............................G..........................
15iTBGc-1(3,1)    G.TG....G....AT.A.C.....AACCCAAAGCGAGAACAAGA.........................................G..............
6TBGa-1(10,1)     ................A.......--------------------........................................................
6TBGa-2(9,1)      ................A.......--------------------........................................................
6TBGa-3(3,1)      ................A.......--------------------........................................................
6TBGa-4(2,1)      ................A.......--------------------.......................G................................
6TBGa-5(1,1)      ................A.......--------------------........................................................
6TBGb-1(7,B)      G.C.....G....GCAA.A.....AATCCACACGGGGAACAAGA...............A........................CA.....C.A......
6TBGb-2(6,1)      G.C.....G....GCAA.A.....AATCCACACGGGGAACAAGA...............A........................CA.....C.A......
6TBGb-3(2,1)      G.C.....G....GCAA.A.....AATCCACACGGGGAACAAGA...............A........................CA.....C.A......
6TBGc-1(6,B)      ................A.......--------------------........................................................
6TBGc-2(3,1)      ................A.......--------------------........................................................
6TBGd(10,B)       ----------------------------------------------------------------------------------------------------
6TBGe(2,1)        CGCCAT.ACGC..AGCAAG..A.GAAATCCACACGGGGAACAAGA.GAG.CAGTG..TGTA.TGAGTGAG.ACACTGCAG.TCTG..AG.C.CAACTG.C
```

```
                        3810      3820      3830      3840      3850      3860      3870      3880      3890      3900
                 ....|....|....|....|....|....|....|....|....|....|....|....|....|....|....|....|....|....|....|....|
NTBGa-1(35,2)    GAGGGACCGCCCAATTGAGGGTGTGCGACCTCCAACTCAAAGCCAATTGG-AAGAAAGAAACCATAGAAAGGAA-GAAAAGGGGAGGAAGACAGAGATCC
NTBGa-2(8,1)     ..................................................-.......................-.........................
NTBGa-3(6,B)     ..................................................-.......................-.........................
NTBGa-4(5,B)     ..................................................-.......................-.........................
NTBGa-6(2,1)     ..................................................-.......................-.........................
NTBGa-7(1,1)     ..................................................-.......................-.........................
NTBGa-8(1,1)     ..................................................-.......................-.........................
NTBGa-10(1,1)    ..................................................-.......................-.........................
NTBGa-11(1,1)    ..................................................-.......................-.........................
NTBGa-12(1,1)    ..................................................-.......................-.........................
NTBGa-13(1,1)    ..................................................-.......................-.........................
NTBGa-15(1,1)    ----------------------------------------------------------------------------------------------------
NTBGa-16(1,B)    ..................................................-.......................-.........................
NTBGb(4,1)       ..................................................-.......................-.........................
NTBGd(1,1)       ..................................................-.......................-.........................
NTBGc(2,1)       ..................................................-.......................-.........................
NTBGe(1,1)       ..................................................-.......................-.........................
P2aTBGa-1(10,2)  ..................................................-.......................-.GC......................
P2aTBGa-2(3,1)   ..................................................-.......................-.GC......................
P2aTBGa-3(1,1)   ..................................................-.......................-.GC......................
P2aTBGb-1(2,2)   ........AGAG.....................................C-.............C.A.......-.G..........G............
P2aTBGb-2(2,1)   ........AGAG......................................-.............C.A.......-.G..........G............
P2aTBGb-3(1,1)   ........AGAG......................................-.............C.A.......-.G..........G............
P2aTBGb-4(1,1)   ........AGAG......................................-.............C.A.......-.G..........G............
P2aTBGc-1(1,B)   ........A..A..C..........T........T......T...G....-...................A...-..CT.CAA.................
P2aTBGc-2(1,1)   ........A..A..C..........T........T......T...G....-...................A...-..CT.CAA.................
P2aTBGc-3(1,1)   ........A..A..C..........T........T......T...G....-...................A...-..CT.CAA.................
P2aTBGc-4(1,1)   ........A..A..C..........T........T......T...G....-...................A...-..CT.CAA.................
P2aTBGc-6(1,1)   ........A..A..C..........T........T......T...G....-...................A...-..CT.CAA.................
P2aTBGc-5(1,B)   ........A..A..C..........T........T......T...G....-...................A...-..CT.CAA.................
15iTBGa-1(6,B)   .........................................T...G....-.......................-.G.......................
15iTBGa-2(4,2)   .........................................T...G....-.......................-.G.......................
15iTBGa-3(3,2)   .........................................T...G....-.......................-.G.......................
15iTBGa-4(3,B)   .........................................T...G....-.......................-.G.......................
15iTBGa-5(2,B)   .........................................T...G....-.......................-.G.......................
15iTBGa-6(2,1)   .........................................T...G....-.......................-.G.......................
15iTBGa-7(1,B)   .........................................T...G....-.......................-.G.......................
15iTBGa-8(1,1)   .........................................T...G....-.......................-.G.......................
15iTBGa-9(1,1)   .........................................T...G....-.......................-.G.......................
15iTBGb-1(4,2)   .........................................T...G....-.......................A.G.......................
15iTBGb-2(2,2)   .........................................T...G....-.......................-.G.......................
15iTBGb-4(1,1)   .........................................T...G....G.......................-.G.......................
15iTBGb-5(1,B)   .........................................T...G....-.......................-.G.......................
15iTBGb-7(1,1)   ..................A......................T...G....-.......................-.G.......................
15iTBGc-1(3,1)   ..CA..............................................-.....................G.A..CT.CAA.................
6TBGa-1(10,1)    .........................T...............T...G....A.GA..GA..-.............-.G.......................
6TBGa-2(9,1)     .........................T...............T...G....A.GA..GA................-.G.......................
6TBGa-3(3,1)     .........................T...............T...G....A.GA..GA..-.............-.........................
6TBGa-4(2,1)     .........................T...............T...G....A.GA..GA..C---------------------------------------
6TBGa-5(1,1)     .........................T...............T...G....A.GA..GA..-.............-.G.......................
6TBGb-1(7,B)     ........A..A..C..........T........T......T...G....A.GA..GA..-.........A...-..CT.CAA.................
6TBGb-2(6,1)     ........A..A..C..........T........T......T...G....A.GA..GA..-.........A...-..CT.CAA.................
6TBGb-3(2,1)     ........A..A..C..........T........T......T...G....A.GA..GA..-.........A...-..CT.CAA.................
6TBGc-1(6,B)     ...........G......................................A.GA..GA..-.............-.G..........G............
6TBGc-2(3,1)     ...........G......................................A.GA..GA..-.............-.G..........G............
6TBGd(10,B)      ----------------------------------------------------------------------------------------------------
6TBGe(2,1)       TGA..GA.CA....ACTGA..GTGTGTGA.CT.C.TCTC..AT.C.G.T.G..........-.CATAG..AAG.A-G.CTACAAGA.G.AGACAGAGAT.

                        3910      3920      3930      3940      3950      3960      3970      3980      3990      4000
                 ....|....|....|....|....|....|....|....|....|....|....|....|....|....|....|....|....|....|....|
NTBGa-1(35,2)    TGGAAGAGATATGGGCATTTGGGGAAATAGTGTGACCATGTATCAGGCTTTGTGGACATCTAACGAATATGTCATGTTTTTGTAAATACAAGCATGCACG
NTBGa-2(8,1)     ....................................................................................................
NTBGa-3(6,B)     ....................................................................................................
NTBGa-4(5,B)     ....................................................................................................
NTBGa-6(2,1)     ....................................................................................................
NTBGa-7(1,1)     ....................................................................................................
NTBGa-8(1,1)     ....................................................................................................
NTBGa-10(1,1)    ....................................................................................................
NTBGa-11(1,1)    ....................................................................................................
NTBGa-12(1,1)    ....................................................................................................
NTBGa-13(1,1)    ....................................................................................................
NTBGa-15(1,1)    ----------------------------------------------------------------------------------------------------
NTBGa-16(1,B)    ....................................................................................................
NTBGb(4,1)       ..............................C.....................................................................
NTBGd(1,1)       ....................................................................................................
NTBGc(2,1)       ....................................................................................................
NTBGe(1,1)       ...................................................................................G................
P2aTBGa-1(10,2)  .....................................G..............................................................
P2aTBGa-2(3,1)   .....................................G..............................................................
P2aTBGa-3(1,1)   .....................................G..............................................................
P2aTBGb-1(2,2)   ..............................................................................................-.....
P2aTBGb-2(2,1)   ..............................................................................................-.....
P2aTBGb-3(1,1)   ..............................................................................................-.....
P2aTBGb-4(1,1)   .......................A......................................................................-.....
P2aTBGc-1(1,B)   ..A..A..GG.CA.A.....T.....T..ACA..G............G...TA...AT.....T........A.G.C..C..G.....T..A.......A
P2aTBGc-2(1,1)   ..A..A..GG.CA.A.....T.....T..ACA..G............G...TA...AT.....T........A.G.C..C..G.....T..A.......A
P2aTBGc-3(1,1)   ..A..A..GG.CA.A.....T.....T..ACA..G............G...TA...AT.....T........A.G.C..C..G.....T..A.......A
P2aTBGc-4(1,1)   ..A..A..GG.CA.A.....T.....T..ACA..G............G...TA...AT.....T........A.G.C..C..G.....T..A.......A
P2aTBGc-6(1,1)   ..A..A..GG.CA.A.....T.....T..ACA..G............G...TA...AT.....T........A.G.C..C..G.....T..A.......A
P2aTBGc-5(1,B)   ..A..A..GG.CA.A.....T.....T..ACA..G............G...TA...AT.....T........A.G.C..C..G.....T..A.......A
15iTBGa-1(6,B)   .....................................G...............................................C..............
15iTBGa-2(4,2)   .....................................G...............................................C..............
15iTBGa-3(3,2)   .....................................G...............................................C..............
15iTBGa-4(3,B)   .....................................G...............................................C..............
15iTBGa-5(2,B)   .....................................G...............................................C..............
15iTBGa-6(2,1)   .....................................G...............................................C..............
15iTBGa-7(1,B)   .....................................G...............................................C..............
15iTBGa-8(1,1)   .....................................G...............................................C..............
15iTBGa-9(1,1)   .....................................G...............................................C..............
15iTBGb-1(4,2)   ..................................................................................................T.
15iTBGb-2(2,2)   ..................................................................................................T.
15iTBGb-4(1,1)   ..................................................................................................T.
15iTBGb-5(1,B)   ..................................................................................................T.
15iTBGb-7(1,1)   ..................................................................................................T.
15iTBGc-1(3,1)   .....................................G..........................A....................C............T.
6TBGa-1(10,1)    ...............................................................T............C........C..............
6TBGa-2(9,1)     ...............................................................T............C........C..............
6TBGa-3(3,1)     ...............................................................T............C........C..............
6TBGa-4(2,1)     ---............................................................T............C........C..............
6TBGa-5(1,1)     ...............................................................T............C........C..............
6TBGb-1(7,B)     ...G.A..GG.CA.A.....T.....T..ACA..G............GG.-.A...AT.....T........A.G.C..C..G.....T..A.......A
6TBGb-2(6,1)     ...G.A..GG.CA.A.....T.....T..ACA..G............GG.-.A...AT.....T........A.G.C..C..G.....T..A.......A
6TBGb-3(2,1)     ...G.A..GG.CA.A.....T.....T..ACA..G............GG.-.A...AT.....T........A.G.C..C..G.....T..A.......A
6TBGc-1(6,B)     .....................................G..............................................................
6TBGc-2(3,1)     .....................................G..............................................................
6TBGd(10,B)      ----------------------------------------------------------------------------------------------------
6TBGe(2,1)       CT.GGA.AGGGACA.ACA..TT..G...TAACATGG.CATGTATCA.GGG..A...AT.....T........A.G.C..C..G.....T..A.......A
```

```
                         4010      4020      4030
                    ....|....|....|....|....|....|....
NTBGa-1(35,2)       CAGAAACAAAGGGAGAAAACTGCTTTGGGTGTTA
NTBGa-2(8,1)        ..................................
NTBGa-3(6,B)        ..................................
NTBGa-4(5,B)        ..................................
NTBGa-6(2,1)        ..................................
NTBGa-7(1,1)        ..................................
NTBGa-8(1,1)        ................................AT
NTBGa-10(1,1)       ..................................
NTBGa-11(1,1)       ..................................
NTBGa-12(1,1)       ..................................
NTBGa-13(1,1)       ..................................
NTBGa-15(1,1)       ----------------------------------
NTBGa-16(1,B)       ..................................
NTBGb(4,1)          ..................................
NTBGd(1,1)          ..................................
NTBGc(2,1)          ..................................
NTBGe(1,1)          ..................................
P2aTBGa-1(10,2)     ...........C......................
P2aTBGa-2(3,1)      ...........T......................
P2aTBGa-3(1,1)      ...........T......................
P2aTBGb-1(2,2)      ...........T......................
P2aTBGb-2(2,1)      ...........T......................
P2aTBGb-3(1,1)      ...........C......................
P2aTBGb-4(1,1)      ...........C......................
P2aTBGc-1(1,B)      .....GT....C......................
P2aTBGc-2(1,1)      .....GT....C......................
P2aTBGc-3(1,1)      .....GT....C......................
P2aTBGc-4(1,1)      .....GT....C......................
P2aTBGc-6(1,1)      .....GT....C......................
P2aTBGc-5(1,B)      .....GT....T......................
15iTBGa-1(6,B)      ...........C......................
15iTBGa-2(4,2)      ...........C......................
15iTBGa-3(3,2)      ...........C......................
15iTBGa-4(3,B)      ...........C......................
15iTBGa-5(2,B)      ...........C......................
15iTBGa-6(2,1)      ...........C......................
15iTBGa-7(1,B)      ...........C......................
15iTBGa-8(1,1)      ...........C......................
15iTBGa-9(1,1)      ...........T......................
15iTBGb-1(4,2)      ...........C......................
15iTBGb-2(2,2)      ...........C......................
15iTBGb-4(1,1)      ...........C......................
15iTBGb-5(1,B)      ...........T......................
15iTBGb-7(1,1)      ...........C......................
15iTBGc-1(3,1)      ..................................
6TBGa-1(10,1)       ...........C......................
6TBGa-2(9,1)        ...........T......................
6TBGa-3(3,1)        ...........T......................
6TBGa-4(2,1)        ...........C......................
6TBGa-5(1,1)        ...........T......................
6TBGb-1(7,B)        .....GT....T......................
6TBGb-2(6,1)        .....GT....T......................
6TBGb-3(2,1)        .....GT....C......................
6TBGc-1(6,B)        ...........C......................
6TBGc-2(3,1)        ...........T......................
6TBGd(10,B)         ----------------------------------
6TBGe(2,1)          .....GT....C......................
```

Supplementary Fig. S1. Alignment of nucleotide sequences from all 57 different clones considered to be confirmed (that is, with obvious chimeras not included), including both exons and apparent introns found in the clones. Names of the transcripts follow the convention: abbreviated line name, “T” for T cells, “BG”, a letter representing the exon 2 sequence with “a” being the most frequently detected exon 2 sequence (and “b” being the second most frequently detected exon 2 sequence, and so forth), a dash and then a number representing the alternative splicing variant with “1” being the most frequently detected clone (and “2” the second most frequently detected clone, and so forth). Numbers in parentheses indicate the number of clones found for a particular full sequence, followed by the number of independent PCRs in which the sequence was identified (1, found in one PCR; 2 found in 2 PCRs; B, found in one PCR described in this paper and one using B cell cDNA, data not shown). Letters indicate nucleotides, dots indicate identities with NTBGa-1 sequence, dashes indicate no sequence present compared to one or more of the other sequences. Those sequences representative of each of the 16 genes based on exon 2, as well as those alternatively-expressed transcripts identified in more than one independent PCR, were deposited in GenBank with the accession numbers as follows: NTBGa-1, MH156615; NTBGa-3, MH156616; NTBGa-4, MH156617; NTBGa-16, MH156618; NTBGb, MH156619; NTBGc, MH156620; NTBGd, MH156621; NTBGe, MH156622; P2aTBGa-1, MH156623; P2aTBGb-1, MH156624; P2aTBGc-1, MH156625; P2aTBGc-5, MH156626; 15iTBGa-1, MH156627; 15iTBGa-2, MH156628; 15iTBGa-3, MH156629; 15iTBGa-4, MH156630; 15iTBGa-5, MH156631; 15iTBGa-7, MH156632; 15iTBGb-1, MH156633; 15iTBGb-2, MH156634; 15iTBGb-5, MH156635; 15iTBGc-1, MH156636; 6TBGa-1, MH156637; 6TBGa-2, MH156638; 6TBGb-1, MH156639; 6TBGb-2, MH156640; 6TBGc-1, MH156641; 6TBGd, MH156642; 6TBGe, MH156643. Note: PCR-based mutations in NTBGa-1 are likely to have generated NTBGa-2 (positions 695 and 1232), NTBGa-8 (position 795) and NTBGc (positions 242, 542 and insertion at position 906), and in NTBGa-4 to generate NTBGb (positions 173 and 1666).

1.2 Supplementary Figure S2

```
 'H' primer  5' TCCGCTCGAGCTCTCTYCTCCTACAG
   (UC206)
                          10        20        30        40        50        60        70        80        90       100
                  ....|....|....|....|....|....|....|....|....|....|....|....|....|....|....|....|....|....|....|....|
KC955130_BG8      ATCCGCTCGAGCTCTCTCCTCCTACAGCTTCTGCCCTCATATTCTCCCCACACTTCTTCCCCATATTCTTTCCAAATCCTCTTCCCCATCTCCTCCACCG
NTBGa-1(35/68,2)  -..........................T.....................................................................T..
NTBGb(4/4,1)      -................T.........T.....................................................................T..
NTBGc(2/2,1)      -................T.........T.....................................................................T..
NTBGd(1/1,1)      -..........................T.....................................................................T..
NTBGe(1/1,1)      -................T.........T.....................................................................T..
P2aTBGa-1(10/14,2) -................T.............................A..G..C..............................................
P2aTBGb-1(2/6,2)  -..G.............T..................................................................................
P2aTBGc-1(1/6,B)  -................T..................................................................................
15iTBGa-1(6/24,2) -............................G...................................................................T..
15iTBGb-1(4/11,2) -................T...........G......................................................................
15iTBGc-1(3/4,1)  -................................................................................................T.A
6TBGa-1(10/46,1)  -................T.........T..T...........A.............---................................T.......A
6TBGc-1(6/11,B)   -................T..................................................................................
6TBGb-1(7/15,B)   -................T..........C.....................T................................................A
6TBGd(10/10,1)    ----------------------------------------------------------------------------------------------------
6TBGe(2/2,1)      -...........................C.....................T................................................A

                         110       120       130       140       150       160       170       180       190       200
                  ....|....|....|....|....|....|....|....|....|....|....|....|....|....|....|....|....|....|....|....|
KC955130_BG8      TCTCCTTCTCAGAGTCCTTCCTCTCTCTCCCTAAATTCTTCCCCCCTCCTCTTCTCCAGCACAGATGGCCTTCACATCGGGCTGCAACCACCCCAGTTTC
NTBGa-1(35/68,2)  ....................................................................................................
NTBGb(4/4,1)      ....................................................................................................
NTBGc(2/2,1)      ....................................................................................................
NTBGd(1/1,1)      ...........A..........................................................................C..C..........
NTBGe(1/1,1)      ....................................................................................................
P2aTBGa-1(10/14,2) ....................................................................................................
P2aTBGb-1(2/6,2)  ....T............................................... ..............CG..........A.....................
P2aTBGc-1(1/6,B)  ....T...............................................................................................
15iTBGa-1(6/24,2) ..........................................T.........................................................
15iTBGb-1(4/11,2) ....................................................................................................
15iTBGc-1(3/4,1)  ....................................................................................................
6TBGa-1(10/46,1)  ............TA....................................................CA................................
6TBGc-1(6/11,B)   ....................................................................................................
6TBGb-1(7/15,B)   ..........................A.......................................CG................................
6TBGd(10/10,1)    ----------------------------------------------------------------------------------------------------
6TBGe(2/2,1)      ..........................A.......................................CG................................

                     'SS'     5' CTCCTGCCTTATCTCRTGGCTCTGCAC 3'
                     primer
                         210       220       230       240       250       260       270       280       290       300
                  ....|....|....|....|....|....|....|....|....|....|....|....|....|....|....|....|....|....|....|....|
KC955130_BG8      GCCCTCCCCTGGAGGACCCTCCTGCCTTATCTCGTGGCTCTGCACTTCCTCCAGCCGGGATCAGGTAGGGGTCCTGTGGGGCTGCTGTGCCTGGCACAGG
NTBGa-1(35/68,2)  A............................................C..................------------------------------------
NTBGb(4/4,1)      A............................................C..................------------------------------------
NTBGc(2/2,1)      A.........................................T..C..................------------------------------------
NTBGd(1/1,1)      A.................................G..........C..................------------------------------------
NTBGe(1/1,1)      A............................................C..................------------------------------------
P2aTBGa-1(10/14,2) .............................................C..................------------------------------------
P2aTBGb-1(2/6,2)  A............................................C..................------------------------------------
P2aTBGc-1(1/6,B)  A............................................C..................------------------------------------
15iTBGa-1(6/24,2) A............................................C..................------------------------------------
15iTBGb-1(4/11,2) .................................C...........C.......C..........------------------------------------
15iTBGc-1(3/4,1)  A............................................C.......C..........------------------------------------
6TBGa-1(10/46,1)  A............................................C.........T...C....------------------------------------
6TBGc-1(6/11,B)   ................................................................------------------------------------
6TBGb-1(7/15,B)   A............................................C.....G..T........------------------------------------
6TBGd(10/10,1)    ------------------...........................C.....G..T........------------------------------------
6TBGe(2/2,1)      A............................................C.....G..T........------------------------------------

                         310       320       330       340       350       360       370       380       390       400
                  ....|....|....|....|....|....|....|....|....|....|....|....|....|....|....|....|....|....|....|....|
KC955130_BG8      TGTTGCTGTGCCGCTCCATGCCCCACATTAACACAGACACCATCTCACCATCTCTCCGTGCCCTTCTCATTGCCCAGCCCAGCTCACGGTGGTGGCACCG
NTBGa-1(35/68,2)  -----------------------------------------------------------------------------.....A.................
NTBGb(4/4,1)      -----------------------------------------------------------------------------.....A...T.............
NTBGc(2/2,1)      -----------------------------------------------------------------------------.....A.................
NTBGd(1/1,1)      -----------------------------------------------------------------------------....AA......G..........
NTBGe(1/1,1)      -----------------------------------------------------------------------------.....A.................
P2aTBGa-1(10/14,2) -----------------------------------------------------------------------------.....AG................
P2aTBGb-1(2/6,2)  -----------------------------------------------------------------------------.......................
P2aTBGc-1(1/6,B)  -----------------------------------------------------------------------------.......................
15iTBGa-1(6/24,2) -----------------------------------------------------------------------------......AA.G.............
15iTBGb-1(4/11,2) -----------------------------------------------------------------------------.......................
15iTBGc-1(3/4,1)  -----------------------------------------------------------------------------.......................
6TBGa-1(10/46,1)  -----------------------------------------------------------------------------.......................
6TBGc-1(6/11,B)   -----------------------------------------------------------------------------.......................
6TBGb-1(7/15,B)   -----------------------------------------------------------------------------.....AG................
6TBGd(10/10,1)    -----------------------------------------------------------------------------.....AG................
6TBGe(2/2,1)      -----------------------------------------------------------------------------.....AG................
```

```
                             410       420       430       440       450       460       470       480       490       500
                    ....|....|....|....|....|....|....|....|....|....|....|....|....|....|....|....|....|....|....|....|
KC955130_BG8        AGCCTCCGTGTCACTGCCAATGTGGGACAGGACGTTGTGCTGCGCTGCCACTTGTCCCCATGCAAGGATGTTCGGAATTCAGACATCAGATGGATCCAGC
NTBGa-1(35/68,2)    ...................TC...........T...................................................................
NTBGb(4/4,1)        ...................TC...........T...................................................................
NTBGc(2/2,1)        ...................TC...........T...................................................................
NTBGd(1/1,1)        ...................TC...........T...................................................................
NTBGe(1/1,1)        ...................TC...........T...................................................................
P2aTBGa-1(10/14,2)  ....................C...............................................................................
P2aTBGb-1(2/6,2)    .............G.....T..............................G........T........................................
P2aTBGc-1(1/6,B)    .............G.....T..............................G........T........................................
15iTBGa-1(6/24,2)   ....................C...........T...............................T...................................
15iTBGb-1(4/11,2)   .............G.....T...............C.......................T........................................
15iTBGc-1(3/4,1)    ........................................................T..TC.........C........T.................T..
6TBGa-1(10/46,1)    ....................C...........T......................G...T..........C.T...GA.TG...................
6TBGc-1(6/11,B)     ....................................................................................................
6TBGb-1(7/15,B)     ...................TC...........T..C.A.....................T........................................
6TBGd(10/10,1)      ....................C...........T..........................T........................................
6TBGe(2/2,1)        ...................TC...........T..C.A.....................T........................................


                             510       520       530       540       550       560       570       580       590       600
                    ....|....|....|....|....|....|....|....|....|....|....|....|....|....|....|....|....|....|....|....|
KC955130_BG8        AGCGGTCCTCTCGGCTTGTGCACCACTACCGAAATGGAGTGGACCTGGGGCAGATGGAGGAATATAAAGGGAGAACAGAACTGCTCAGGGATGGTCTCTC
NTBGa-1(35/68,2)    ....................................................................................................
NTBGb(4/4,1)        ....................................................................................................
NTBGc(2/2,1)        ....................................................................................................
NTBGd(1/1,1)        ....................................................................................................
NTBGe(1/1,1)        ...................................................................................................A
P2aTBGa-1(10/14,2)  ....................................................................................................
P2aTBGb-1(2/6,2)    ...........G..A...............A.T........................................G..........................
P2aTBGc-1(1/6,B)    ...........G..A...............A.G........................................G..........................
15iTBGa-1(6/24,2)   ....................................................................................................
15iTBGb-1(4/11,2)   ...........G..A...............A.G........................................G.................C........
15iTBGc-1(3/4,1)    ...........G..A........GC.......................A................G................A.................
6TBGa-1(10/46,1)    ...........G.TT...............A.G......A........A...................................................
6TBGc-1(6/11,B)     ....................................................................................................
6TBGb-1(7/15,B)     T..........A..A...............A.......T.........AT.......................G..........................
6TBGd(10/10,1)      T..........G..A...............A.......T.........AT...............G.......G..........................
6TBGe(2/2,1)        T..........A..A...............A.......T.........AT.......................G.........T................


                             610       620       630       640       650       660       670       680       690       700
                    ....|....|....|....|....|....|....|....|....|....|....|....|....|....|....|....|....|....|....|....|
KC955130_BG8        TGATGGAAACCTGGATTTGCGCATCACTGCCGTGACCTCCTCTGATAGTGGCTCCTACAGCTGTGCTGTGCAAGATGGTGATGCCTATGCAGAAGCTGTG
NTBGa-1(35/68,2)    ..............................T.....................................................................
NTBGb(4/4,1)        ..............................T.....................................................................
NTBGc(2/2,1)        ..............................T........................C............................................
NTBGd(1/1,1)        ..............................T.....................................................................
NTBGe(1/1,1)        ..............................T....G.A....C...........A.................G..........G.........C......
P2aTBGa-1(10/14,2)  .............................................C......................................................
P2aTBGb-1(2/6,2)    ......................................................A..T.T.....G....G..A...C......................
P2aTBGc-1(1/6,B)    ..............................T....G.A................A..T.T.....G....G..A...C......................
15iTBGa-1(6/24,2)   ..............................T..........................................A.........G................
15iTBGb-1(4/11,2)   ......................................................A..T.T.....G....A..A..........................
15iTBGc-1(3/4,1)    ....................TT........T..................................T......G....................C.....T
6TBGa-1(10/46,1)    .........T....................T....G..................A....................C........................
6TBGc-1(6/11,B)     ....................................................................................................
6TBGb-1(7/15,B)     ..........................T...T....G.........C.......TG..........T...........AC....G................
6TBGd(10/10,1)      ..........................T...T....G.........C.......TG..........T...........AC....G................
6TBGe(2/2,1)        ..........................T...T....G.........C.......TG..........T...........AC....G................


                             710       720       730       740       750       760       770       780       790       800
                    ....|....|....|....|....|....|....|....|....|....|....|....|....|....|....|....|....|....|....|....|
KC955130_BG8        GTGAACCTGGAGGTGTCAGGTCAGTGGCTGGGGTGTTCAAGGATGGAGAGCTGACGGATCGCAGCCTTTGGAAGTGGTCAGGGCTGAACAGCTCCATGAG
NTBGa-1(35/68,2)    ...................---------------------------------------------------------------------------------
NTBGb(4/4,1)        ...................---------------------------------------------------------------------------------
NTBGc(2/2,1)        ...................---------------------------------------------------------------------------------
NTBGd(1/1,1)        ...................---------------------------------------------------------------------------------
NTBGe(1/1,1)        ...G...............---------------------------------------------------------------------------------
P2aTBGa-1(10/14,2)  ...................---------------------------------------------------------------------------------
P2aTBGb-1(2/6,2)    ...................---------------------------------------------------------------------------------
P2aTBGc-1(1/6,B)    ...................---------------------------------------------------------------------------------
15iTBGa-1(6/24,2)   ...................------ ---------------------------------------------------------------------------
15iTBGb-1(4/11,2)   ...................---------------------------------------------------------------------------------
15iTBGc-1(3/4,1)    ...G.G.............---------------------------------------------------------------------------------
6TBGa-1(10/46,1)    ...................---------------------------------------------------------------------------------
6TBGc-1(6/11,B)     ...................---------------------------------------------------------------------------------
6TBGb-1(7/15,B)     ...................---------------------------------------------------------------------------------
6TBGd(10/10,1)      ...................---------------------------------------------------------------------------------
6TBGe(2/2,1)        ...................---------------------------------------------------------------------------------
```

```
                            810       820       830       840       850       860       870       880       890       900
                     ....|....|....|....|....|....|....|....|....|....|....|....|....|....|....|....|....|....|....|....|
KC955130_BG8         ATGCTGGAATTGCAGTGGGCGCACGCTGTGATTTGGAGATGGGTCTGCATGGATGAGGTGGTTGGGTTGGGTTTCTGGGATGGGTTTCTCCATGGCTCAG
NTBGa-1(35/68,2)     ----------------------------------------------------------------------------------------------------
NTBGb(4/4,1)         ----------------------------------------------------------------------------------------------------
NTBGc(2/2,1)         ----------------------------------------------------------------------------------------------------
NTBGd(1/1,1)         ----------------------------------------------------------------------------------------------------
NTBGe(1/1,1)         ----------------------------------------------------------------------------------------------------
P2aTBGa-1(10/14,2)   ----------------------------------------------------------------------------------------------------
P2aTBGb-1(2/6,2)     ----------------------------------------------------------------------------------------------------
P2aTBGc-1(1/6,B)     ----------------------------------------------------------------------------------------------------
15iTBGa-1(6/24,2)    ----------------------------------------------------------------------------------------------------
15iTBGb-1(4/11,2)    ----------------------------------------------------------------------------------------------------
15iTBGc-1(3/4,1)     ----------------------------------------------------------------------------------------------------
6TBGa-1(10/46,1)     ----------------------------------------------------------------------------------------------------
6TBGc-1(6/11,B)      ----------------------------------------------------------------------------------------------------
6TBGb-1(7/15,B)      ----------------------------------------------------------------------------------------------------
6TBGd(10/10,1)       ----------------------------------------------------------------------------------------------------
6TBGe(2/2,1)         ----------------------------------------------------------------------------------------------------

                            910       920       930       940       950       960       970       980       990      1000
                     ....|....|....|....|....|....|....|....|....|....|....|....|....|....|....|....|....|....|....|....|
KC955130_BG8         TGGCAGTCGGCACACAATGCTGAGCAGCTCCTCTGCCTGTGCCAATGTGGGGATGCTGCTATTGTGTGTCACTGCTCGCTGGTTGCTGCCCCTTCGGGTT
NTBGa-1(35/68,2)     ----------------------------------------------------------------------------------------------------
NTBGb(4/4,1)         ----------------------------------------------------------------------------------------------------
NTBGc(2/2,1)         ----------------------------------------------------------------------------------------------------
NTBGd(1/1,1)         ----------------------------------------------------------------------------------------------------
NTBGe(1/1,1)         ----------------------------------------------------------------------------------------------------
P2aTBGa-1(10/14,2)   ----------------------------------------------------------------------------------------------------
P2aTBGb-1(2/6,2)     ----------------------------------------------------------------------------------------------------
P2aTBGc-1(1/6,B)     ----------------------------------------------------------------------------------------------------
15iTBGa-1(6/24,2)    ----------------------------------------------------------------------------------------------------
15iTBGb-1(4/11,2)    ----------------------------------------------------------------------------------------------------
15iTBGc-1(3/4,1)     ----------------------------------------------------------------------------------------------------
6TBGa-1(10/46,1)     ----------------------------------------------------------------------------------------------------
6TBGc-1(6/11,B)      ----------------------------------------------------------------------------------------------------
6TBGb-1(7/15,B)      ----------------------------------------------------------------------------------------------------
6TBGd(10/10,1)       ----------------------------------------------------------------------------------------------------
6TBGe(2/2,1)         ----------------------------------------------------------------------------------------------------

                           1010      1020      1030      1040      1050      1060      1070      1080      1090      1100
                     ....|....|....|....|....|....|....|....|....|....|....|....|....|....|....|....|....|....|....|....|
KC955130_BG8         CTGTGATCTCCCAAGGCTGAGTCTTGCTTTTCCACATATGGGAATTTAAAAGGACCCTCTTCTTGACATTTCTTCCAGACCCCTTTTCTATGATCATCCT
NTBGa-1(35/68,2)     ------------------------------------------------------------------------------......................
NTBGb(4/4,1)         ------------------------------------------------------------------------------......................
NTBGc(2/2,1)         ------------------------------------------------------------------------------......................
NTBGd(1/1,1)         ------------------------------------------------------------------------------......................
NTBGe(1/1,1)         ------------------------------------------------------------------------------.T........CCA....G...A
P2aTBGa-1(10/14,2)   ------------------------------------------------------------------------------......................
P2aTBGb-1(2/6,2)     ------------------------------------------------------------------------------......................
P2aTBGc-1(1/6,B)     ------------------------------------------------------------------------------.T....................
15iTBGa-1(6/24,2)    ------------------------------------------------------------------------------......................
15iTBGb-1(4/11,2)    ------------------------------------------------------------------------------......................
15iTBGc-1(3/4,1)     ------------------------------------------------------------------------------.....AA...............
6TBGa-1(10/46,1)     ------------------------------------------------------------------------------.T........CCAA...G...A
6TBGc-1(6/11,B)      ------------------------------------------------------------------------------......................
6TBGb-1(7/15,B)      ------------------------------------------------------------------------------.T........CCA....G...A
6TBGd(10/10,1)       ------------------------------------------------------------------------------.T.T......CCA....G...A
6TBGe(2/2,1)         ------------------------------------------------------------------------------.T........CCA....G...A

              'TM'   5' GACCTKYCACCGAGACCGACAC 3'
              primer    <-----------------------
                        CTGGAKYGTGGCTCTGGCTGTG
                           1110      1120      1130      1140      1150      1160      1170      1180      1190      1200
                     ....|....|....|....|....|....|....|....|....|....|....|....|....|....|....|....|....|....|....|....|
KC955130_BG8         TTACTGGACAGTGGCTCTGGCTGTGATCATCACACTTCTGGTTGGGTCATTTGTCGTCAATGTTTTTCTCCATAGAAAGAAAGGTGAGCTGAGAGCGGAG
NTBGa-1(35/68,2)     ...................................................................................-----------------
NTBGb(4/4,1)         ...................................................................................-----------------
NTBGc(2/2,1)         ...................................................................................-----------------
NTBGd(1/1,1)         ...................................................................................-----------------
NTBGe(1/1,1)         .CC.....AG...............G..G.....A....C...............A..............TG...G.......-----------------
P2aTBGa-1(10/14,2)   ...................................................................................-----------------
P2aTBGb-1(2/6,2)     .......................................................A...T..C....................-----------------
P2aTBGc-1(1/6,B)     ...................................................................................-----------------
15iTBGa-1(6/24,2)    ...................................................................................-----------------
15iTBGb-1(4/11,2)    ...................................................................................-----------------
15iTBGc-1(3/4,1)     ............................G..........................A...T..C....................-----------------
6TBGa-1(10/46,1)     .CC.....AG.................AG..........................A...T..C.......T............-----------------
6TBGc-1(6/11,B)      ...................................................................................-----------------
6TBGb-1(7/15,B)      .CC.....AG...............G..G.....A................C...A...T..........TG...G.......-----------------
6TBGd(10/10,1)       .CC.....AG...............---------------------------------------------------------------------------
6TBGe(2/2,1)         .CC.....AG...............G..G.....A................C...A...T..........TG...G.......-----------------
```

```
                          1210      1220      1230      1240      1250      1260      1270      1280      1290      1300
                    ....|....|....|....|....|....|....|....|....|....|....|....|....|....|....|....|....|....|....|....|
KC955130_BG8        GGGATGGAGCACAGGGAGGTGTTGTGCATGGACAGGGATGGTCGGGGTGGTGCTGAGCTGTGGTCCACGGAGGTACACAGGTGGAGGAACCGTGACTTTT
NTBGa-1(35/68,2)    ----------------------------------------------------------------------------------------------------
NTBGb(4/4,1)        ----------------------------------------------------------------------------------------------------
NTBGc(2/2,1)        ----------------------------------------------------------------------------------------------------
NTBGd(1/1,1)        ----------------------------------------------------------------------------------------------------
NTBGe(1/1,1)        ----------------------------------------------------------------------------------------------------
P2aTBGa-1(10/14,2)  ----------------------------------------------------------------------------------------------------
P2aTBGb-1(2/6,2)    ----------------------------------------------------------------------------------------------------
P2aTBGc-1(1/6,B)    ----------------------------------------------------------------------------------------------------
15iTBGa-1(6/24,2)   ----------------------------------------------------------------------------------------------------
15iTBGb-1(4/11,2)   ----------------------------------------------------------------------------------------------------
15iTBGc-1(3/4,1)    ----------------------------------------------------------------------------------------------------
6TBGa-1(10/46,1)    ----------------------------------------------------------------------------------------------------
6TBGc-1(6/11,B)     ----------------------------------------------------------------------------------------------------
6TBGb-1(7/15,B)     ----------------------------------------------------------------------------------------------------
6TBGd(10/10,1)      ----------------------------------------------------------------------------------------------------
6TBGe(2/2,1)        ----------------------------------------------------------------------------------------------------

                          1310      1320      1330      1340      1350      1360      1370      1380      1390      1400
                    ....|....|....|....|....|....|....|....|....|....|....|....|....|....|....|....|....|....|....|....|
KC955130_BG8        CATGGGATTCCCAGTGCTCATTAAATAACATTTGCCTTTCTTTTGGGGAATAAAAGAAGGGGAAAAACGATAGTGGTAAGGGTGGGCAGATAGGAATGTG
NTBGa-1(35/68,2)    ----------------------------------------------------------------------------------------------------
NTBGb(4/4,1)        ----------------------------------------------------------------------------------------------------
NTBGc(2/2,1)        ----------------------------------------------------------------------------------------------------
NTBGd(1/1,1)        ----------------------------------------------------------------------------------------------------
NTBGe(1/1,1)        ----------------------------------------------------------------------------------------------------
P2aTBGa-1(10/14,2)  ----------------------------------------------------------------------------------------------------
P2aTBGb-1(2/6,2)    ----------------------------------------------------------------------------------------------------
P2aTBGc-1(1/6,B)    ----------------------------------------------------------------------------------------------------
15iTBGa-1(6/24,2)   ----------------------------------------------------------------------------------------------------
15iTBGb-1(4/11,2)   ----------------------------------------------------------------------------------------------------
15iTBGc-1(3/4,1)    ----------------------------------------------------------------------------------------------------
6TBGa-1(10/46,1)    ----------------------------------------------------------------------------------------------------
6TBGc-1(6/11,B)     ----------------------------------------------------------------------------------------------------
6TBGb-1(7/15,B)     ----------------------------------------------------------------------------------------------------
6TBGd(10/10,1)      ----------------------------------------------------------------------------------------------------
6TBGe(2/2,1)        ----------------------------------------------------------------------------------------------------

                          1410      1420      1430      1440      1450      1460      1470      1480      1490      1500
                    ....|....|....|....|....|....|....|....|....|....|....|....|....|....|....|....|....|....|....|....|
KC955130_BG8        GCTGGACTGTGGGGCAGGTGGAAAGTCCAAACCCTCTGGAGAAGTCCCCACAAACCAAGCTGCCCTGCTGACCAGCTATTTCTCTGCTTTGTTTTCCAGT
NTBGa-1(35/68,2)    ---------------------------------------------------------------------------------------------------.
NTBGb(4/4,1)        ---------------------------------------------------------------------------------------------------.
NTBGc(2/2,1)        ---------------------------------------------------------------------------------------------------.
NTBGd(1/1,1)        ---------------------------------------------------------------------------------------------------.
NTBGe(1/1,1)        ---------------------------------------------------------------------------------------------------C
P2aTBGa-1(10/14,2)  ---------------------------------------------------------------------------------------------------.
P2aTBGb-1(2/6,2)    ---------------------------------------------------------------------------------------------------C
P2aTBGc-1(1/6,B)    ---------------------------------------------------------------------------------------------------.
15iTBGa-1(6/24,2)   ---------------------------------------------------------------------------------------------------.
15iTBGb-1(4/11,2)   ---------------------------------------------------------------------------------------------------C
15iTBGc-1(3/4,1)    ---------------------------------------------------------------------------------------------------C
6TBGa-1(10/46,1)    ---------------------------------------------------------------------------------------------------C
6TBGc-1(6/11,B)     ---------------------------------------------------------------------------------------------------.
6TBGb-1(7/15,B)     ---------------------------------------------------------------------------------------------------.
6TBGd(10/10,1)      ---------------------------------------------------------------------------------------------------.
6TBGe(2/2,1)        ---------------------------------------------------------------------------------------------------.

                          1510      1520      1530      1540      1550      1560      1570      1580      1590      1600
                    ....|....|....|....|....|....|....|....|....|....|....|....|....|....|....|....|....|....|....|....|
KC955130_BG8        GGCACAGAGCAGAGAGCTGAGTGAGTCCTTCCATCCCCATCCACCACCAAAGTCCCTTTAATGGAACTGACAGCAGACTGCAGAGTGCTGGGT-TATGCC
NTBGa-1(35/68,2)    ....................--------------------------------------------------------------------------------
NTBGb(4/4,1)        ....................--------------------------------------------------------------------------------
NTBGc(2/2,1)        ....................--------------------------------------------------------------------------------
NTBGd(1/1,1)        ....................--------------------------------------------------------------------------------
NTBGe(1/1,1)        ....................--------------------------------------------------------------------------------
P2aTBGa-1(10/14,2)  ....................--------------------------------------------------------------------------------
P2aTBGb-1(2/6,2)    T..T.......C........--------------------------------------------------------------------------------
P2aTBGc-1(1/6,B)    ....................--------------------------------------------------------------------------------
15iTBGa-1(6/24,2)   ....................--------------------------------------------------------------------------------
15iTBGb-1(4/11,2)   ....................--------------------------------------------------------------------------------
15iTBGc-1(3/4,1)    T.....T.............--------------------------------------------------------------------------------
6TBGa-1(10/46,1)    .................................G.T..T...............................T..A...................T......
6TBGc-1(6/11,B)     ....................--------------------------------------------------------------------------------
6TBGb-1(7/15,B)     ..T.................--------------------------------------------------------------------------------
6TBGd(10/10,1)      ----------------------------------------------------------------------------------------------------
6TBGe(2/2,1)        ..T...............A.--------------------------------------------------------------------------------
```

```
                            1610      1620      1630      1640      1650      1660      1670      1680      1690      1700
                   ....|....|....|....|....|....|....|....|....|....|....|....|....|....|....|....|....|....|....|....|
KC955130_BG8       ATGTGCTGGGGCCATGAGCTATGTTGAGGCTTTGGAATGTGTTGGGGTTGTGGGATGTACTGGGGTCGTGGGATGTGTCAATCCTGGCTGATTCACGTGG
NTBGa-1(35/68,2)   ----------------------------------------------------------------------------------------------------
NTBGb(4/4,1)       ----------------------------------------------------------------------------------------------------
NTBGc(2/2,1)       ----------------------------------------------------------------------------------------------------
NTBGd(1/1,1)       ----------------------------------------------------------------------------------------------------
NTBGe(1/1,1)       ----------------------------------------------------------------------------------------------------
P2aTBGa-1(10/14,2) ----------------------------------------------------------------------------------------------------
P2aTBGb-1(2/6,2)   ----------------------------------------------------------------------------------------------------
P2aTBGc-1(1/6,B)   ----------------------------------------------------------------------------------------------------
15iTBGa-1(6/24,2)  ----------------------------------------------------------------------------------------------------
15iTBGb-1(4/11,2)  ----------------------------------------------------------------------------------------------------
15iTBGc-1(3/4,1)   ----------------------------------------------------------------------------------------------------
6TBGa-1(10/46,1)   T.....A.........G.A.C.A-..G.A.C....G...........CC.........G...............C...........AT....C.T.-.TC
6TBGc-1(6/11,B)    ----------------------------------------------------------------------------------------------------
6TBGb-1(7/15,B)    ----------------------------------------------------------------------------------------------------
6TBGd(10/10,1)     ----------------------------------------------------------------------------------------------------
6TBGe(2/2,1)       ----------------------------------------------------------------------------------------------------

                            1710      1720      1730      1740      1750      1760      1770      1780      1790      1800
                   ....|....|....|....|....|....|....|....|....|....|....|....|....|....|....|....|....|....|....|....|
KC955130_BG8       AAAAACCTTTCACAATCGGTTCCTTCCAGTTTGTTTAATTCCTTCTTGGGCCCAAAGTGGTCATTGGACTCCTCCCAGAAA-AAAGGGTTTGGGGTCAGG
NTBGa-1(35/68,2)   ----------------------------------------------------------------------------------------------------
NTBGb(4/4,1)       ----------------------------------------------------------------------------------------------------
NTBGc(2/2,1)       ----------------------------------------------------------------------------------------------------
NTBGd(1/1,1)       ----------------------------------------------------------------------------------------------------
NTBGe(1/1,1)       ----------------------------------------------------------------------------------------------------
P2aTBGa-1(10/14,2) ----------------------------------------------------------------------------------------------------
P2aTBGb-1(2/6,2)   ----------------------------------------------------------------------------------------------------
P2aTBGc-1(1/6,B)   ----------------------------------------------------------------------------------------------------
15iTBGa-1(6/24,2)  ----------------------------------------------------------------------------------------------------
15iTBGb-1(4/11,2)  ----------------------------------------------------------------------------------------------------
15iTBGc-1(3/4,1)   ----------------------------------------------------------------------------------------------------
6TBGa-1(10/46,1)   .G..CT...G.C...............GA..CT.....C...........A................C...T.AAT.....G...A.A.....A...T..
6TBGc-1(6/11,B)    ----------------------------------------------------------------------------------------------------
6TBGb-1(7/15,B)    ----------------------------------------------------------------------------------------------------
6TBGd(10/10,1)     ----------------------------------------------------------------------------------------------------
6TBGe(2/2,1)       ----------------------------------------------------------------------------------------------------

                            1810      1820      1830      1840      1850      1860      1870      1880      1890      1900
                   ....|....|....|....|....|....|....|....|....|....|....|....|....|....|....|....|....|....|....|....|
KC955130_BG8       GTGTGAGAGCTGATGGCATGGAAACGTGTCCCCTCTGACCATGCATTTCATTTGCTTCTATTTTGCAGAGAGAAAAGATGCAGAGTTGGGTAAGTCTCCT
NTBGa-1(35/68,2)   --------------------------------------------------------------------.....................-----------
NTBGb(4/4,1)       --------------------------------------------------------------------.....................-----------
NTBGc(2/2,1)       --------------------------------------------------------------------.....................-----------
NTBGd(1/1,1)       --------------------------------------------------------------------.....................-----------
NTBGe(1/1,1)       --------------------------------------------------------------------...............C.....-----------
P2aTBGa-1(10/14,2) --------------------------------------------------------------------.....................-----------
P2aTBGb-1(2/6,2)   --------------------------------------------------------------------.....................-----------
P2aTBGc-1(1/6,B)   --------------------------------------------------------------------..G............CAC...-----------
15iTBGa-1(6/24,2)  --------------------------------------------------------------------.....................TGG..AAAG..
15iTBGb-1(4/11,2)  --------------------------------------------------------------------..............AT.....-----------
15iTBGc-1(3/4,1)   --------------------------------------------------------------------...............C.....-----------
6TBGa-1(10/46,1)   ..A..G...-------------------------------------------------------------------------------------------
6TBGc-1(6/11,B)    --------------------------------------------------------------------.....................-----------
6TBGb-1(7/15,B)    --------------------------------------------------------------------..G............CAC...-----------
6TBGd(10/10,1)     ----------------------------------------------------------------------------------------------------
6TBGe(2/2,1)       --------------------------------------------------------------------..G............CAC...-----------

                            1910      1920      1930      1940      1950      1960      1970      1980      1990      2000
                   ....|....|....|....|....|....|....|....|....|....|....|....|....|....|....|....|....|....|....|....|
KC955130_BG8       TCCCTAAAGCGAGGGAATTCAGGGTGTCCCCATGGCATCAGCCGTGGAATTAGTAGCTGTCCTCTCTGACAATTCACTGCTCTGCTCTTTCCTTTCCAGT
NTBGa-1(35/68,2)   ---------------------------------------------------------------------------------------------------.
NTBGb(4/4,1)       ---------------------------------------------------------------------------------------------------.
NTBGc(2/2,1)       ---------------------------------------------------------------------------------------------------.
NTBGd(1/1,1)       ---------------------------------------------------------------------------------------------------.
NTBGe(1/1,1)       ---------------------------------------------------------------------------------------------------C
P2aTBGa-1(10/14,2) ---------------------------------------------------------------------------------------------------.
P2aTBGb-1(2/6,2)   ---------------------------------------------------------------------------------------------------.
P2aTBGc-1(1/6,B)   ---------------------------------------------------------------------------------------------------C
15iTBGa-1(6/24,2)  G.AGC.TT.GTGA.A..AGAT.CAGCA.TGGCG.AG.AAGTTGCA.C.T.GGAG..AAAAGA.GCAATGTTG--------------------------..
15iTBGb-1(4/11,2)  ---------------------------------------------------------------------------------------------------G
15iTBGc-1(3/4,1)   ---------------------------------------------------------------------------------------------------G
6TBGa-1(10/46,1)   --------------------------------------.....A...G..G..A..G...T.C.......C..G..........................
6TBGc-1(6/11,B)    ---------------------------------------------------------------------------------------------------.
6TBGb-1(7/15,B)    ---------------------------------------------------------------------------------------------------C
6TBGd(10/10,1)     ----------------------------------------------------------------------------------------------------
6TBGe(2/2,1)       ---------------------------------------------------------------------------------------------------C
```

```
                          2010      2020      2030      2040      2050      2060      2070      2080      2090      2100
                    ....|....|....|....|....|....|....|....|....|....|....|....|....|....|....|....|....|....|....|....|
KC955130_BG8        GGAGAAAGCTGCAGCATTGGGTGAGTTATATTCCCCA---AGCCAAAGTACTTTGGGTCTTCCCATTGGAAGTTATTTCCTCAGACCATCCTTTCTGTTG
NTBGa-1(35/68,2)    ....................--------------------------------------------------------------------------------
NTBGb(4/4,1)        ....................--------------------------------------------------------------------------------
NTBGc(2/2,1)        ....................--------------------------------------------------------------------------------
NTBGd(1/1,1)        ....................--------------------------------------------------------------------------------
NTBGe(1/1,1)        ...ACT..A..AGAT..C..--------------------------------------------------------------------------------
P2aTBGa-1(10/14,2)  ....................--------------------------------------------------------------------------------
P2aTBGb-1(2/6,2)    ...CC...............--------------------------------------------------------------------------------
P2aTBGc-1(1/6,B)    ...ACT.C....GAT.....--------------------------------------------------------------------------------
15iTBGa-1(6/24,2)   ....................--------------------------------------------------------------------------------
15iTBGb-1(4/11,2)   TTTA.GT......AATC..A--------------------------------------------------------------------------------
15iTBGc-1(3/4,1)    TTCCT.T......AATC..A--------------------------------------------------------------------------------
6TBGa-1(10/46,1)    ...CC...............--------------------------------------------------------------------------------
6TBGc-1(6/11,B)     ....................--------------------------------------------------------------------------------
6TBGb-1(7/15,B)     ...ACT.C....GAT.....--------------------------------------------------------------------------------
6TBGd(10/10,1)      ----------------------------------------------------------------------------------------------------
6TBGe(2/2,1)        ...ACT.C....GAT.....--------------------------------------------------------------------------------
```

```
                          2110      2120      2130      2140      2150      2160      2170      2180      2190      2200
                    ....|....|....|....|....|....|....|....|....|....|....|....|....|....|....|....|....|....|....|....|
KC955130_BG8        TGTTTGCTTTGGCATCATGTTAGTAAAATGCCTTCTTGGGACCAAAGTGGTCATTGGCCACTTCCCAGAAAAAAAGATTTGGGGGCAGGGTGTGGGAGCT
NTBGa-1(35/68,2)    ----------------------------------------------------------------------------------------------------
NTBGb(4/4,1)        ----------------------------------------------------------------------------------------------------
NTBGc(2/2,1)        ----------------------------------------------------------------------------------------------------
NTBGd(1/1,1)        ----------------------------------------------------------------------------------------------------
NTBGe(1/1,1)        ----------------------------------------------------------------------------------------------------
P2aTBGa-1(10/14,2)  ----------------------------------------------------------------------------------------------------
P2aTBGb-1(2/6,2)    ----------------------------------------------------------------------------------------------------
P2aTBGc-1(1/6,B)    ----------------------------------------------------------------------------------------------------
15iTBGa-1(6/24,2)   ----------------------------------------------------------------------------------------------------
15iTBGb-1(4/11,2)   ----------------------------------------------------------------------------------------------------
15iTBGc-1(3/4,1)    ----------------------------------------------------------------------------------------------------
6TBGa-1(10/46,1)    ----------------------------------------------------------------------------------------------------
6TBGc-1(6/11,B)     ----------------------------------------------------------------------------------------------------
6TBGb-1(7/15,B)     ----------------------------------------------------------------------------------------------------
6TBGd(10/10,1)      ----------------------------------------------------------------------------------------------------
6TBGe(2/2,1)        ----------------------------------------------------------------------------------------------------
```

```
                          2210      2220      2230      2240      2250      2260      2270      2280      2290      2300
                    ....|....|....|....|....|....|....|....|....|....|....|....|....|....|....|....|....|....|....|....|
KC955130_BG8        GATGGCATGGAAATTTGTCCCCTCTGACCATGCTTTTCCTTTGCTTCTTTTTGCAGAGAGAAAAGATGCAGAGTTGGGTAAGTCTCCTTCCCCACAGTGA
NTBGa-1(35/68,2)    --------------------------------------------------------.....................-----------------------
NTBGb(4/4,1)        --------------------------------------------------------.....................-----------------------
NTBGc(2/2,1)        --------------------------------------------------------.....................-----------------------
NTBGd(1/1,1)        --------------------------------------------------------.....................-----------------------
NTBGe(1/1,1)        --------------------------------------------------------GTTT..GT.C..A.A.TC..A-----------------------
P2aTBGa-1(10/14,2)  --------------------------------------------------------.....................-----------------------
P2aTBGb-1(2/6,2)    --------------------------------------------------------.....................-----------------------
P2aTBGc-1(1/6,B)    --------------------------------------------------------GTGT.TGT.C....A.TC..A-----------------------
15iTBGa-1(6/24,2)   --------------------------------------------------------............A........-----------------------
15iTBGb-1(4/11,2)   --------------------------------------------------------...A.TT..CCT..A.AC..A-----------------------
15iTBGc-1(3/4,1)    --------------------------------------------------------...CCCT..CCT..A.AC..A-----------------------
6TBGa-1(10/46,1)    --------------------------------------------------------...................................T.A..C..
6TBGc-1(6/11,B)     --------------------------------------------------------.....................-----------------------
6TBGb-1(7/15,B)     --------------------------------------------------------GTGT.TGTAC....A.TC..A-----------------------
6TBGd(10/10,1)      ----------------------------------------------------------------------------------------------------
6TBGe(2/2,1)        --------------------------------------------------------GTGT.TGTAC....A.TC..A-----------------------
```

```
                          2310      2320      2330      2340      2350      2360      2370      2380      2390      2400
                    ....|....|....|....|....|....|....|....|....|....|....|....|....|....|....|....|....|....|....|....|
KC955130_BG8        GGGAATTCAGGGTTTCCCCATGGCGTTAGCCACGGGATGGGCAGCTGTCCTCTCTGACCATGCACTGCTCTGCTCTTTCTTTTCCAGCGGAACAAGCAGC
NTBGa-1(35/68,2)    ---------------------------------------------------------------------------------------.............
NTBGb(4/4,1)        ---------------------------------------------------------------------------------------.............
NTBGc(2/2,1)        ---------------------------------------------------------------------------------------.............
NTBGd(1/1,1)        ----------------------------------------------------------------------------------------------------
NTBGe(1/1,1)        ----------------------------------------------------------------------------------------------------
P2aTBGa-1(10/14,2)  ---------------------------------------------------------------------------------------.............
P2aTBGb-1(2/6,2)    ---------------------------------------------------------------------------------------.............
P2aTBGc-1(1/6,B)    ---------------------------------------------------------------------------------------A.ATC.T...TT.
15iTBGa-1(6/24,2)   ---------------------------------------------------------------------------------------.............
15iTBGb-1(4/11,2)   ---------------------------------------------------------------------------------------ACA..A.T..T.A
15iTBGc-1(3/4,1)    ---------------------------------------------------------------------------------------ACAG.ATT..T.A
6TBGa-1(10/46,1)    .............C..........A.C...TGT......A...............................................T.....T..AT.A
6TBGc-1(6/11,B)     ---------------------------------------------------------------------------------------.............
6TBGb-1(7/15,B)     ---------------------------------------------------------------------------------------A.ATC.T...TT.
6TBGd(10/10,1)      ----------------------------------------------------------------------------------------------------
6TBGe(2/2,1)        ---------------------------------------------------------------------------------------A.ATC.T...TT.
```

```
                         2410      2420      2430      2440      2450      2460      2470      2480      2490      2500
                     ....|....|....|....|....|....|....|....|....|....|....|....|....|....|....|....|....|....|....|....|
KC955130_BG8         GCTATCGAGTGAGTCTCCCCCTCCATTTTTATTATTTTTAAATGTTCAGCCTCCGGTAGCTGTGGGATGAGATGTTCCTCTCATCATACACTGACTCTGC
NTBGa-1(35/68,2)     ..A.....--------------------------------------------------------------------------------------------
NTBGb(4/4,1)         ..A.....--------------------------------------------------------------------------------------------
NTBGc(2/2,1)         ..A.....--------------------------------------------------------------------------------------------
NTBGd(1/1,1)         -------------------------------------------------...................................................
NTBGe(1/1,1)         ----------------------------------------------------------------------------------------------------
P2aTBGa-1(10/14,2)   ........--------------------------------------------------------------------------------------------
P2aTBGb-1(2/6,2)     ........--------------------------------------------------------------------------------------------
P2aTBGc-1(1/6,B)     AAA.CT..--------------------------------------------------------------------------------------------
15iTBGa-1(6/24,2)    ........--------------------------------------------------------------------------------------------
15iTBGb-1(4/11,2)    CGA.GT.G--------------------------------------------------------------------------------------------
15iTBGc-1(3/4,1)     CGA.GT.G--------------------------------------------------------------------------------------------
6TBGa-1(10/46,1)     .A.....G--------------------------------------------------------------------------------------------
6TBGc-1(6/11,B)      ........--------------------------------------------------------------------------------------------
6TBGb-1(7/15,B)      AAA.CT..--------------------------------------------------------------------------------------------
6TBGd(10/10,1)       ----------------------------------------------------------------------------------------------------
6TBGe(2/2,1)         AAA.CT..--------------------------------------------------------------------------------------------

                         2510      2520      2530      2540      2550      2560      2570      2580      2590      2600
                     ....|....|....|....|....|....|....|....|....|....|....|....|....|....|....|....|....|....|....|....|
KC955130_BG8         TTTTCCTTTGCAGAGCAAAGAGATGCAATGTTGGGTGAGTCTCCCACCTGAAACCAAAGAGATTTGGGGTCTTCCCATGGGATCAGCCATGGGATGATAA
NTBGa-1(35/68,2)     -------------.....................------------------------------------------------------------------
NTBGb(4/4,1)         -------------.....................------------------------------------------------------------------
NTBGc(2/2,1)         -------------.....................------------------------------------------------------------------
NTBGd(1/1,1)         ..................................------------------------------------------------------------------
NTBGe(1/1,1)         ----------------------------------------------------------------------------------------------------
P2aTBGa-1(10/14,2)   -------------.....................------------------------------------------------------------------
P2aTBGb-1(2/6,2)     -------------..............G......------------------------------------------------------------------
P2aTBGc-1(1/6,B)     -------------T.A..CA.ATG.A..AA....------------------------------------------------------------------
15iTBGa-1(6/24,2)    -------------.....................------------------------------------------------------------------
15iTBGb-1(4/11,2)    -------------..G.TT.CA..T..GA.C..A------------------------------------------------------------------
15iTBGc-1(3/4,1)     -------------..G.TT.CA..TT.GGTC..A------------------------------------------------------------------
6TBGa-1(10/46,1)     -------------GTTT...T.C..A..GTC..A------------------------------------------------------------------
6TBGc-1(6/11,B)      -------------.....................------------------------------------------------------------------
6TBGb-1(7/15,B)      -------------T.A..CA.ATG.A..AA....------------------------------------------------------------------
6TBGd(10/10,1)       ----------------------------------------------------------------------------------------------------
6TBGe(2/2,1)         -------------T.A..CA.ATG.A..AA....------------------------------------------------------------------

                         2610      2620      2630      2640      2650      2660      2670      2680      2690      2700
                     ....|....|....|....|....|....|....|....|....|....|....|....|....|....|....|....|....|....|....|....|
KC955130_BG8         CCTGAACCTTCTCATCGTGCGTTTCTTATTTGTTCCTTTTGCAGAGAAACACGTTCTAAAACTGGGTGAGTCCTCACTCCCAAATTATAAAGCAAAGGGT
NTBGa-1(35/68,2)     --------------------------------------------.C...................-----------------------------------
NTBGb(4/4,1)         --------------------------------------------.C...................-----------------------------------
NTBGc(2/2,1)         --------------------------------------------.C...................-----------------------------------
NTBGd(1/1,1)         --------------------------------------------.C...................-----------------------------------
NTBGe(1/1,1)         --------------------------------------------..C..TTA.C.TC.......A-----------------------------------
P2aTBGa-1(10/14,2)   --------------------------------------------.C...................-----------------------------------
P2aTBGb-1(2/6,2)     --------------------------------------------.....................-----------------------------------
P2aTBGc-1(1/6,B)     --------------------------------------------...TT..GAA.TC.CTCT..A-----------------------------------
15iTBGa-1(6/24,2)    --------------------------------------------.....................-----------------------------------
15iTBGb-1(4/11,2)    --------------------------------------------....GG..TG.GA.G.GA...-----------------------------------
15iTBGc-1(3/4,1)     --------------------------------------------...G.G..TG.GA...GA..C-----------------------------------
6TBGa-1(10/46,1)     --------------------------------------------..C..TTA.C.TC.......A-----------------------------------
6TBGc-1(6/11,B)      --------------------------------------------.....................-----------------------------------
6TBGb-1(7/15,B)      --------------------------------------------...TT..GAA.TC.GTCT...-----------------------------------
6TBGd(10/10,1)       ----------------------------------------------------------------------------------------------------
6TBGe(2/2,1)         --------------------------------------------...TT..GAA.TC.GTCT...-----------------------------------

                         2710      2720      2730      2740      2750      2760      2770      2780      2790      2800
                     ....|....|....|....|....|....|....|....|....|....|....|....|....|....|....|....|....|....|....|....|
KC955130_BG8         TCTGCCTGTGTGAGCTGTGGGATCAGACGTTCCACTCATCATGCATTGCTTTTCTCTTTCTTTTTCAGAGGAAAAGACAGACGAAGTGGGTGAGTCTACA
NTBGa-1(35/68,2)     --------------------------------------------------------------------.....................-----------
NTBGb(4/4,1)         --------------------------------------------------------------------.....................-----------
NTBGc(2/2,1)         --------------------------------------------------------------------.....................-----------
NTBGd(1/1,1)         -------------------------------------------------------------------A.....................-----------
NTBGe(1/1,1)         --------------------------------------------------------------------.C.....TG.T..........-----------
P2aTBGa-1(10/14,2)   --------------------------------------------------------------------.....................-----------
P2aTBGb-1(2/6,2)     --------------------------------------------------------------------.....................-----------
P2aTBGc-1(1/6,B)     --------------------------------------------------------------------..A..CG.TAT..GATTAC..-----------
15iTBGa-1(6/24,2)    --------------------------------------------------------------------.....................-----------
15iTBGb-1(4/11,2)    --------------------------------------------------------------------GTTCTGTCGTT.CA..CC..A-----------
15iTBGc-1(3/4,1)     --------------------------------------------------------------------GTTCTGGCGTT.CA..TC..A-----------
6TBGa-1(10/46,1)     --------------------------------------------------------------------.C.....TG.T..........-----------
6TBGc-1(6/11,B)      --------------------------------------------------------------------.....................-----------
6TBGb-1(7/15,B)      --------------------------------------------------------------------..A..CG.TAT..GA.TAC..-----------
6TBGd(10/10,1)       ----------------------------------------------------------------------------------------------------
6TBGe(2/2,1)         --------------------------------------------------------------------..A..CG.TAT..GA.TAC..-----------
```

```
                              2810      2820      2830      2840      2850      2860      2870      2880      2890      2900
                     ....|....|....|....|....|....|....|....|....|....|....|....|....|....|....|....|....|....|....|....|
KC955130_BG8         TTCACTAAAGCAAAGAAATATGGGGTCTCCCATGGGATGACAAGCTGTCCCAAAAAATCATGTGGTGCTTTTTCTTGTCTTTTTATTTATTTATTTATTT
NTBGa-1(35/68,2)     ----------------------------------------------------------------------------------------------------
NTBGb(4/4,1)         ----------------------------------------------------------------------------------------------------
NTBGc(2/2,1)         ----------------------------------------------------------------------------------------------------
NTBGd(1/1,1)         ----------------------------------------------------------------------------------------------------
NTBGe(1/1,1)         ----------------------------------------------------------------------------------------------------
P2aTBGa-1(10/14,2)   ----------------------------------------------------------------------------------------------------
P2aTBGb-1(2/6,2)     ----------------------------------------------------------------------------------------------------
P2aTBGc-1(1/6,B)     ----------------------------------------------------------------------------------------------------
15iTBGa-1(6/24,2)    ----------------------------------------------------------------------------------------------------
15iTBGb-1(4/11,2)    ----------------------------------------------------------------------------------------------------
15iTBGc-1(3/4,1)     ----------------------------------------------------------------------------------------------------
6TBGa-1(10/46,1)     ----------------------------------------------------------------------------------------------------
6TBGc-1(6/11,B)      ----------------------------------------------------------------------------------------------------
6TBGb-1(7/15,B)      ----------------------------------------------------------------------------------------------------
6TBGd(10/10,1)       ----------------------------------------------------------------------------------------------------
6TBGe(2/2,1)         ----------------------------------------------------------------------------------------------------

                              2910      2920      2930      2940      2950      2960      2970      2980      2990      3000
                     ....|....|....|....|....|....|....|....|....|....|....|....|....|....|....|....|....|....|....|....|
KC955130_BG8         ATTTATTTGCAGAGAATTGGAATTCAGTGCTGAGTAAGTTGCAGTCACTGAACTGAGGGAATGTGGGGTCTTCCTAAGGGACTGCGTAGGGGAGAAGTTC
NTBGa-1(35/68,2)     ------------.....................-------------------------------------------------------------------
NTBGb(4/4,1)         ------------.....................-------------------------------------------------------------------
NTBGc(2/2,1)         ------------.....................-------------------------------------------------------------------
NTBGd(1/1,1)         ------------.....................-------------------------------------------------------------------
NTBGe(1/1,1)         ------------..G....C.......A.....-------------------------------------------------------------------
P2aTBGa-1(10/14,2)   ------------.....................-------------------------------------------------------------------
P2aTBGb-1(2/6,2)     ------------.....................-------------------------------------------------------------------
P2aTBGc-1(1/6,B)     ------------..G.ACT.GC.G...AT...G-------------------------------------------------------------------
15iTBGa-1(6/24,2)    ------------.....................-------------------------------------------------------------------
15iTBGb-1(4/11,2)    ------------....ACT.GC.G...AAT..G-------------------------------------------------------------------
15iTBGc-1(3/4,1)     ------------..G.AGT.GC.G..AAA...G-------------------------------------------------------------------
6TBGa-1(10/46,1)     ------------..C....C....T..AT....-------------------------------------------------------------------
6TBGc-1(6/11,B)      ------------.....................-------------------------------------------------------------------
6TBGb-1(7/15,B)      ------------..G.ACT.GC.G...AT...G-------------------------------------------------------------------
6TBGd(10/10,1)       ----------------------------------------------------------------------------------------------------
6TBGe(2/2,1)         ------------..G.ACT.GC.G...AT...G-------------------------------------------------------------------

                              3010      3020      3030      3040      3050      3060      3070      3080      3090      3100
                     ....|....|....|....|....|....|....|....|....|....|....|....|....|....|....|....|....|....|....|....|
KC955130_BG8         CCATGCACTGCTTTTCTCTTTCTTTTCCAGAGAAAGACAGTGAAGAGATGGGTGAGTCTCTCCTCCCAAATTAAAAACGTTGGGGTTCCCATGTGGGAGC
NTBGa-1(35/68,2)     ------------------------------.A...................-------------------------------------------------
NTBGb(4/4,1)         ------------------------------.A...................-------------------------------------------------
NTBGc(2/2,1)         -----------------------------A.A...................-------------------------------------------------
NTBGd(1/1,1)         ------------------------------.A...................-------------------------------------------------
NTBGe(1/1,1)         ------------------------------........T............-------------------------------------------------
P2aTBGa-1(10/14,2)   ------------------------------.A...................-------------------------------------------------
P2aTBGb-1(2/6,2)     ------------------------------.....................-------------------------------------------------
P2aTBGc-1(1/6,B)     ------------------------------..G..C.TCT..CT....A..-------------------------------------------------
15iTBGa-1(6/24,2)    ------------------------------.....................-------------------------------------------------
15iTBGb-1(4/11,2)    ------------------------------C.G..C..TA...........-------------------------------------------------
15iTBGc-1(3/4,1)     ------------------------------.....C..TC...........-------------------------------------------------
6TBGa-1(10/46,1)     ------------------------------........T...........C-------------------------------------------------
6TBGc-1(6/11,B)      ------------------------------.....................-------------------------------------------------
6TBGb-1(7/15,B)      ------------------------------..G..C.TCT..CT....A..-------------------------------------------------
6TBGd(10/10,1)       ----------------------------------------------------------------------------------------------------
6TBGe(2/2,1)         ------------------------------..G..C.TCT..CT....A..-------------------------------------------------

                              3110      3120      3130      3140      3150      3160      3170      3180      3190      3200
                     ....|....|....|....|....|....|....|....|....|....|....|....|....|....|....|....|....|....|....|....|
KC955130_BG8         TGTGGGATGAGATGTTCCTCTCATCAACCATCTTTTTTACTTTTCCTTTGCAGGTTATGGCTTTGCAGAACTGAGTAAGTCTCCCTCCCAACACGGAAGG
NTBGa-1(35/68,2)     -----------------------------------------------------............G...T....--------------------------
NTBGb(4/4,1)         -----------------------------------------------------............G...T....--------------------------
NTBGc(2/2,1)         -----------------------------------------------------............G...T....--------------------------
NTBGd(1/1,1)         -----------------------------------------------------............G...T....--------------------------
NTBGe(1/1,1)         -----------------------------------------------------...C....G.......T....--------------------------
P2aTBGa-1(10/14,2)   -----------------------------------------------------............G...T....--------------------------
P2aTBGb-1(2/6,2)     -----------------------------------------------------...C....G.......T....--------------------------
P2aTBGc-1(1/6,B)     -----------------------------------------------------A..TAA..AC......T....--------------------------
15iTBGa-1(6/24,2)    -----------------------------------------------------...C.................--------------------------
15iTBGb-1(4/11,2)    -----------------------------------------------------.GCTATTGAG..T.ACGT...--------------------------
15iTBGc-1(3/4,1)     ----------------------------------------------------------------------------------------------------
6TBGa-1(10/46,1)     -----------------------------------------------------...C....G.......T....--------------------------
6TBGc-1(6/11,B)      -----------------------------------------------------.....................--------------------------
6TBGb-1(7/15,B)      -----------------------------------------------------A..TAA..AC......T....--------------------------
6TBGd(10/10,1)       ----------------------------------------------------------------------------------------------------
6TBGe(2/2,1)         -----------------------------------------------------A..TAA..AC......T....--------------------------
```

```
                           3210      3220      3230      3240      3250      3260      3270      3280      3290      3300
                    ....|....|....|....|....|....|....|....|....|....|....|....|....|....|....|....|....|....|....|....|
KC955130_BG8        GATTTGTGGTCTTCCCATGGGATCAGCCATGGGATGATCATCTGACCCCTCTCATCATGCATTTCGTATTTGTTTCTTTTGCAGAGAAACTGGCTGCAGA
NTBGa-1(35/68,2)    ------------------------------------------------------------------------------------................
NTBGb(4/4,1)        ------------------------------------------------------------------------------------................
NTBGc(2/2,1)        ------------------------------------------------------------------------------------................
NTBGd(1/1,1)        ------------------------------------------------------------------------------------................
NTBGe(1/1,1)        ------------------------------------------------------------------------------------..G...........A.
P2aTBGa-1(10/14,2)  ------------------------------------------------------------------------------------................
P2aTBGb-1(2/6,2)    ------------------------------------------------------------------------------------................
P2aTBGc-1(1/6,B)    ------------------------------------------------------------------------------------..CT...A......A.
15iTBGa-1(6/24,2)   ------------------------------------------------------------------------------------........A...A...
15iTBGb-1(4/11,2)   ------------------------------------------------------------------------------------..CG...A....ACA.
15iTBGc-1(3/4,1)    ----------------------------------------------------------------------------------------------------
6TBGa-1(10/46,1)    ------------------------------------------------------------------------------------...........AG...
6TBGc-1(6/11,B)     ------------------------------------------------------------------------------------................
6TBGb-1(7/15,B)     ------------------------------------------------------------------------------------..CT...A......A.
6TBGd(10/10,1)      ----------------------------------------------------------------------------------------------------
6TBGe(2/2,1)        ------------------------------------------------------------------------------------..CT...A......A.

                           3310      3320      3330      3340      3350      3360      3370      3380      3390      3400
                    ....|....|....|....|....|....|....|....|....|....|....|....|....|....|....|....|....|....|....|....|
KC955130_BG8        ACTGGGTGAGTGCTGCCTCCCAAATTAAATAAAAAATGGGTCTGCCTGGGAGAGTGGTGGGATGGCATGTTCCTCTCACTGCGTGTTGCTTTTCCTTTCT
NTBGa-1(35/68,2)    .....-----------------------------------------------------------------------------------------------
NTBGb(4/4,1)        .....-----------------------------------------------------------------------------------------------
NTBGc(2/2,1)        .....-----------------------------------------------------------------------------------------------
NTBGd(1/1,1)        .....-----------------------------------------------------------------------------------------------
NTBGe(1/1,1)        .T...-----------------------------------------------------------------------------------------------
P2aTBGa-1(10/14,2)  .....-----------------------------------------------------------------------------------------------
P2aTBGb-1(2/6,2)    .....-----------------------------------------------------------------------------------------------
P2aTBGc-1(1/6,B)    .....-----------------------------------------------------------------------------------------------
15iTBGa-1(6/24,2)   .....-----------------------------------------------------------------------------------------------
15iTBGb-1(4/11,2)   .....-----------------------------------------------------------------------------------------------
15iTBGc-1(3/4,1)    ----------------------------------------------------------------------------------------------------
6TBGa-1(10/46,1)    .....-----------------------------------------------------------------------------------------------
6TBGc-1(6/11,B)     .....-----------------------------------------------------------------------------------------------
6TBGb-1(7/15,B)     .....------------------------------------------------------- ---------------------------------------
6TBGd(10/10,1)      ----------------------------------------------------------------------------------------------------
6TBGe(2/2,1)        .....-----------------------------------------------------------------------------------------------

                           3410      3420      3430      3440      3450      3460      3470      3480      3490      3500
                    ....|....|....|....|....|....|....|....|....|....|....|....|....|....|....|....|....|....|....|....|
KC955130_BG8        TTTCCAGAGAAACACTCTGAAGAGATGGGTGAGTCTCCCCTCCCCAATTATAAATGCTGGGGACTTCTTGTGGGAGCTGTGGGATGAGCTCTTCCTCTCA
NTBGa-1(35/68,2)    -------.....................------------------------------------------------------------------------
NTBGb(4/4,1)        -------.....................------------------------------------------------------------------------
NTBGc(2/2,1)        -------.....................------------------------------------------------------------------------
NTBGd(1/1,1)        -------.....................------------------------------------------------------------------------
NTBGe(1/1,1)        -------..G..T.TAT..C..T..ATC------------------------------------------------------------------------
P2aTBGa-1(10/14,2)  -------.....................------------------------------------------------------------------------
P2aTBGb-1(2/6,2)    -------.....................------------------------------------------------------------------------
P2aTBGc-1(1/6,B)    -------T.G....AAGA....CAG...------------------------------------------------------------------------
15iTBGa-1(6/24,2)   -------.....................------------------------------------------------------------------------
15iTBGb-1(4/11,2)   -------.AC...GAA..A....AT...------------------------------------------------------------------------
15iTBGc-1(3/4,1)    ----------------------------------------------------------------------------------------------------
6TBGa-1(10/46,1)    -------..G..A.T...AC..T...TT------------------------------------------------------------------------
6TBGc-1(6/11,B)     -------.....................------------------------------------------------------------------------
6TBGb-1(7/15,B)     -------T.G....AAGA....CAG...------------------------------------------------------------------------
6TBGd(10/10,1)      ----------------------------------------------------------------------------------------------------
6TBGe(2/2,1)        -------T.G....AAGA....CAG...------------------------------------------------------------------------

                           3510      3520      3530      3540      3550      3560      3570      3580      3590      3600
                    ....|....|....|....|....|....|....|....|....|....|....|....|....|....|....|....|....|....|....|....|
KC955130_BG8        TCATGCGCTGTTTCTGCTTTTCCTTTGCAGGGACAAGGGATTTAAAGTTGGGTGAGTCTCTCTTCCCAAACCATACAGATTTGGGGTCTTCCCACGGCAT
NTBGa-1(35/68,2)    ------------------------------.....................-------------------------------------------------
NTBGb(4/4,1)        ------------------------------.....................-------------------------------------------------
NTBGc(2/2,1)        ------------------------------.....................-------------------------------------------------
NTBGd(1/1,1)        ------------------------------.....................-------------------------------------------------
NTBGe(1/1,1)        ------------------------------...G....A..G....A.AAT-------------------------------------------------
P2aTBGa-1(10/14,2)  ------------------------------.....................-------------------------------------------------
P2aTBGb-1(2/6,2)    ------------------------------...........G.........-------------------------------------------------
P2aTBGc-1(1/6,B)    ------------------------------A.GA.C......C.C..C..A-------------------------------------------------
15iTBGa-1(6/24,2)   ------------------------------...........G.........-------------------------------------------------
15iTBGb-1(4/11,2)   ------------------------------AC.A.CAAC...C.C....CC-------------------------------------------------
15iTBGc-1(3/4,1)    --GGACAAGAGCTACTAGCTGCTGAACTGAA..C..T..A..GC.....C.A------------------------------------------------
6TBGa-1(10/46,1)    ------------------------------.....T.....G...T....A-------------------------------------------------
6TBGc-1(6/11,B)     ------------------------------.....................-------------------------------------------------
6TBGb-1(7/15,B)     ------------------------------A.GA.T..A...C.C..C..A-------------------------------------------------
6TBGd(10/10,1)      ----------------------------------------------------------------------------------------------------
6TBGe(2/2,1)        ------------------------------A.GA.T......C.C..C..A-------------------------------------------------
```

```
                         3610      3620      3630      3640      3650      3660      3670      3680      3690      3700
                   ....|....|....|....|....|....|....|....|....|....|....|....|....|....|....|....|....|....|....|....|
KC955130_BG8       CAGCCATGGGATGATAATCGGACCCTTCTCATCATGCATTTCTTATTGGTTCCTTTTGCAGAGCGACTAGCTGCCAAACTGGGTGAGTCCCCCCTCCCAA
NTBGa-1(35/68,2)   -------------------------------------------------------------.....................------------------
NTBGb(4/4,1)       -------------------------------------------------------------.....................------------------
NTBGc(2/2,1)       -------------------------------------------------------------.....................------------------
NTBGd(1/1,1)       -------------------------------------------------------------.....................------------------
NTBGe(1/1,1)       ------------------------------------------------------------------A...............------------------
P2aTBGa-1(10/14,2) -------------------------------------------------------------.....................------------------
P2aTBGb-1(2/6,2)   -------------------------------------------------------------............A........------------------
P2aTBGc-1(1/6,B)   -------------------------------------------------------------G.AA..AGTA..AA..GT...------------------
15iTBGa-1(6/24,2)  -------------------------------------------------------------.....................------------------
15iTBGb-1(4/11,2)  -------------------------------------------------------------..A...ACTT.CAG..TA...------------------
15iTBGc-1(3/4,1)   -------------------------------------------------------------.TAA.................------------------
6TBGa-1(10/46,1)   -------------------------------------------------------------..GT.................------------------
6TBGc-1(6/11,B)    -------------------------------------------------------------.....................------------------
6TBGb-1(7/15,B)    -------------------------------------------------------------G.AA..AGTAC.AA..GT...------------------
6TBGd(10/10,1)     ----------------------------------------------------------------------------------------------------
6TBGe(2/2,1)       -------------------------------------------------------------G.AA..AGTAC.AA..GT...------------------

                         3710      3720      3730      3740      3750      3760      3770      3780      3790      3800
                   ....|....|....|....|....|....|....|....|....|....|....|....|....|....|....|....|....|....|....|....|
KC955130_BG8       ATTAAATAAAAAATGGGGTCTGCCTGTGTGAGCTGTGAGATGAGATGTTCCTCTCATCATGCGCTGCTTTTCTCTTCCTTTTCCAGAACATCAAACTAAA
NTBGa-1(35/68,2)   --------------------------------------------------------------------------------------..............
NTBGb(4/4,1)       --------------------------------------------------------------------------------------..............
NTBGc(2/2,1)       --------------------------------------------------------------------------------------..............
NTBGd(1/1,1)       --------------------------------------------------------------------------------------..............
NTBGe(1/1,1)       --------------------------------------------------------------------------------------C...A.........
P2aTBGa-1(10/14,2) --------------------------------------------------------------------------------------..............
P2aTBGb-1(2/6,2)   --------------------------------------------------------------------------------------..............
P2aTBGc-1(1/6,B)   --------------------------------------------------------------------------------------GTTCG.GTG..GC.
15iTBGa-1(6/24,2)  --------------------------------------------------------------------------------------....A.G.......
15iTBGb-1(4/11,2)  --------------------------------------------------------------------------------------.TTTCAGTG..GG.
15iTBGc-1(3/4,1)   ----------------------------------------------------------------------------------------------------
6TBGa-1(10/46,1)   --------------------------------------------------------------------------------------G...CA..G.....
6TBGc-1(6/11,B)    --------------------------------------------------------------------------------------..............
6TBGb-1(7/15,B)    --------------------------------------------------------------------------------------GTTCG.GTG..GC.
6TBGd(10/10,1)     ----------------------------------------------------------------------------------------------------
6TBGe(2/2,1)       --------------------------------------------------------------------------------------GTTCG.GTG..GC.

                         3810      3820      3830      3840      3850      3860      3870      3880      3890      3900
                   ....|....|....|....|....|....|....|....|....|....|....|....|....|....|....|....|....|....|....|....|
KC955130_BG8       GAATTGGGTGAGTCTTCTTTCCCCAACCCCAAGAAATATGCGTTTCCCATGGGATGACAAGCTGTGCCACCTCATCATGCCCTGTTTTTCTGTCCTTTT
NTBGa-1(35/68,2)   .......---------------------------------------------------------------------------------------------
NTBGb(4/4,1)       .......---------------------------------------------------------------------------------------------
NTBGc(2/2,1)       .......---------------------------------------------------------------------------------------------
NTBGd(1/1,1)       .......---------------------------------------------------------------------------------------------
NTBGe(1/1,1)       .......---------------------------------------------------------------------------------------------
P2aTBGa-1(10/14,2) .......---------------------------------------------------------------------------------------------
P2aTBGb-1(2/6,2)   .......---------------------------------------------------------------------------------------------
P2aTBGc-1(1/6,B)   A.TC..A---------------------------------------------------------------------------------------------
15iTBGa-1(6/24,2)  .......---------------------------------------------------------------------------------------------
15iTBGb-1(4/11,2)  A..CA.A---------------------------------------------------------------------------------------------
15iTBGc-1(3/4,1)   ----------------------------------------------------------------------------------------------------
6TBGa-1(10/46,1)   .......---------------------------------------------------------------------------------------------
6TBGc-1(6/11,B)    .......---------------------------------------------------------------------------------------------
6TBGb-1(7/15,B)    A.TC..A---------------------------------------------------------------------------------------------
6TBGd(10/10,1)     ----------------------------------------------------------------------------------------------------
6TBGe(2/2,1)       A.TC..A---------------------------------------------------------------------------------------------

                         3910      3920      3930      3940      3950      3960      3970      3980      3990      4000
                   ....|....|....|....|....|....|....|....|....|....|....|....|....|....|....|....|....|....|....|....|
KC955130_BG8       TGCAGAGAAACAGCATTCACAGTTCCGTAAGTTGCAGTCACTGAACTGAAGGAATGTGGGGTCTTCCCAAAGTCCTGCATGTGGGATGAAAAATCCCCTC
NTBGa-1(35/68,2)   -----.....................--------------------------------------------------------------------------
NTBGb(4/4,1)       -----.....................--------------------------------------------------------------------------
NTBGc(2/2,1)       -----.....................--------------------------------------------------------------------------
NTBGd(1/1,1)       -----.....................--------------------------------------------------------------------------
NTBGe(1/1,1)       -----.....................--------------------------------------------------------------------------
P2aTBGa-1(10/14,2) -----.....................--------------------------------------------------------------------------
P2aTBGb-1(2/6,2)   -----.....................--------------------------------------------------------------------------
P2aTBGc-1(1/6,B)   -----...C...A.T.AA.A....GG--------------------------------------------------------------------------
15iTBGa-1(6/24,2)  -----.C...................--------------------------------------------------------------------------
15iTBGb-1(4/11,2)  -----..G...T.GT.A..A.AC.GG--------------------------------------------------------------------------
15iTBGc-1(3/4,1)   -----..CT.................--------------------------------------------------------------------------
6TBGa-1(10/46,1)   -----............T.......T--------------------------------------------------------------------------
6TBGc-1(6/11,B)    -----.....................--------------------------------------------------------------------------
6TBGb-1(7/15,B)    -----...C...A.T.AA.A....GG--------------------------------------------------------------------------
6TBGd(10/10,1)     ----------------------------------------------------------------------------------------------------
6TBGe(2/2,1)       -----...C...A.T.AA.A....GG--------------------------------------------------------------------------
```

```
                          4010      4020      4030      4040      4050      4060      4070      4080      4090      4100
                    ....|....|....|....|....|....|....|....|....|....|....|....|....|....|....|....|....|....|....|....|
KC955130_BG8        TGACCATGCACTGCTTTTCTCCTTCTATTCCA--GAGAGACACTTTCAGAATATGTGTGAGTCTCCCCACCCCTGATAAATAAAAACGTTGGGGTCTTGC
NTBGa-1(35/68,2)    -----------------------------------.....................--------------------------------------------
NTBGb(4/4,1)        -----------------------------------.....................--------------------------------------------
NTBGc(2/2,1)        -----------------------------------.....................--------------------------------------------
NTBGd(1/1,1)        -----------------------------------.....................--------------------------------------------
NTBGe(1/1,1)        -----------------------------------.C............CG....G--------------------------------------------
P2aTBGa-1(10/14,2)  -----------------------------------.....................--------------------------------------------
P2aTBGb-1(2/6,2)    -----------------------------------.....................--------------------------------------------
P2aTBGc-1(1/6,B)    -----------------------------------...ACG.AA..G.AG.AG..G--------------------------------------------
15iTBGa-1(6/24,2)   -----------------------------------.C...................--------------------------------------------
15iTBGb-1(4/11,2)   -----------------------------------..GA.....G.G.AC.G...G--------------------------------------------
15iTBGc-1(3/4,1)    -----------------------------------....................G--------------------------------------------
6TBGa-1(10/46,1)    -----------------------------------.C............C......--------------------------------------------
6TBGc-1(6/11,B)     -----------------------------------.....................--------------------------------------------
6TBGb-1(7/15,B)     -----------------------------------...ACG.AA..G.AG.AG..G--------------------------------------------
6TBGd(10/10,1)      ----------------------------------------------------------------------------------------------------
6TBGe(2/2,1)        -----------------------------------...ACG.AA..G.AG.AG..G--------------------------------------------

                          4110      4120      4130      4140      4150      4160      4170      4180      4190      4200
                    ....|....|....|....|....|....|....|....|....|....|....|....|....|....|....|....|....|....|....|....|
KC955130_BG8        TGTGTGAGCTGTGGGATGAGATGTTCCTCTCATCACACATTGTTTTTCTGATTTTCTTTTGCAGATTTAAGTGCTGGAAAACAGAGTAAGTCTCCCTCCC
NTBGa-1(35/68,2)    ----------------------------------------------------------------.....................---------------
NTBGb(4/4,1)        ----------------------------------------------------------------.....................---------------
NTBGc(2/2,1)        ----------------------------------------------------------------.....................---------------
NTBGd(1/1,1)        ----------------------------------------------------------------.....................---------------
NTBGe(1/1,1)        ----------------------------------------------------------------............T...C....---------------
P2aTBGa-1(10/14,2)  ----------------------------------------------------------------.....................---------------
P2aTBGb-1(2/6,2)    ----------------------------------------------------------------.........T..T........---------------
P2aTBGc-1(1/6,B)    ----------------------------------------------------------------.GAA.CACCT.AA...GACTG---------------
15iTBGa-1(6/24,2)   ----------------------------------------------------------------.....................---------------
15iTBGb-1(4/11,2)   ----------------------------------------------------------------.GAG...GAA..T...GTT.G---------------
15iTBGc-1(3/4,1)    ----------------------------------------------------------------.....................---------------
6TBGa-1(10/46,1)    ----------------------------------------------------------------...C.................---------------
6TBGc-1(6/11,B)     ----------------------------------------------------------------.....................---------------
6TBGb-1(7/15,B)     ----------------------------------------------------------------.GAA.CACCT.AA...GATTG---------------
6TBGd(10/10,1)      ----------------------------------------------------------------------------------------------------
6TBGe(2/2,1)        ----------------------------------------------------------------.-AA.CACCT.AA...GATTG---------------

                          4210      4220      4230      4240      4250      4260      4270      4280      4290      4300
                    ....|....|....|....|....|....|....|....|....|....|....|....|....|....|....|....|....|....|....|....|
KC955130_BG8        TGCACAGAAGGAACTTACGGTTTTCCCATGGGATCAGCCATGGGATCATCATCCGACTCTTCTCATCATGAATTTCGTCTTTCTTTCTTTTGCAGAGAAA
NTBGa-1(35/68,2)    -----------------------------------------------------------------------------------------------.....
NTBGb(4/4,1)        -----------------------------------------------------------------------------------------------.....
NTBGc(2/2,1)        -----------------------------------------------------------------------------------------------.....
NTBGd(1/1,1)        -----------------------------------------------------------------------------------------------.....
NTBGe(1/1,1)        -----------------------------------------------------------------------------------------------.....
P2aTBGa-1(10/14,2)  -----------------------------------------------------------------------------------------------.....
P2aTBGb-1(2/6,2)    -----------------------------------------------------------------------------------------------.....
P2aTBGc-1(1/6,B)    -----------------------------------------------------------------------------------------------GT.G.
15iTBGa-1(6/24,2)   -----------------------------------------------------------------------------------------------.....
15iTBGb-1(4/11,2)   -----------------------------------------------------------------------------------------------..GC.
15iTBGc-1(3/4,1)    -----------------------------------------------------------------------------------------------..G..
6TBGa-1(10/46,1)    -----------------------------------------------------------------------------------------------..G..
6TBGc-1(6/11,B)     -----------------------------------------------------------------------------------------------.....
6TBGb-1(7/15,B)     -----------------------------------------------------------------------------------------------GT.T.
6TBGd(10/10,1)      ----------------------------------------------------------------------------------------------------
6TBGe(2/2,1)        -----------------------------------------------------------------------------------------------GT.T.

                          4310      4320      4330      4340      4350      4360      4370      4380      4390      4400
                    ....|....|....|....|....|....|....|....|....|....|....|....|....|....|....|....|....|....|....|....|
KC955130_BG8        ATGGTTACAAAACTGGGTGAGTCCAACCTCCCAAACTAAATTAAAAACAGTCAGACTTTGTGAGCTGTGGGATGAGACGTTCCTCTCATCATGTGCTGCT
NTBGa-1(35/68,2)    ................------------------------------------------------------------------------------------
NTBGb(4/4,1)        ................------------------------------------------------------------------------------------
NTBGc(2/2,1)        ................------------------------------------------------------------------------------------
NTBGd(1/1,1)        ................------------------------------------------------------------------------------------
NTBGe(1/1,1)        C...............------------------------------------------------------------------------------------
P2aTBGa-1(10/14,2)  ................------------------------------------------------------------------------------------
P2aTBGb-1(2/6,2)    ................------------------------------------------------------------------------------------
P2aTBGc-1(1/6,B)    CGT.C.C.T..T...A------------------------------------------------------------------------------------
15iTBGa-1(6/24,2)   ................------------------------------------------------------------------------------------
15iTBGb-1(4/11,2)   CCA.C.GT....G...------------------------------------------------------------------------------------
15iTBGc-1(3/4,1)    C...........T...------------------------------------------------------------------------------------
6TBGa-1(10/46,1)    C...........T...------------------------------------------------------------------------------------
6TBGc-1(6/11,B)     ................------------------------------------------------------------------------------------
6TBGb-1(7/15,B)     CGT.C.C.T..T...A------------------------------------------------------------------------------------
6TBGd(10/10,1)      ----------------------------------------------------------------------------------------------------
6TBGe(2/2,1)        CGT.C.C.T..T...A------------------------------------------------------------------------------------
```

```
                          4410      4420      4430      4440      4450      4460      4470      4480      4490      4500
                   ....|....|....|....|....|....|....|....|....|....|....|....|....|....|....|....|....|....|....|....|
KC955130_BG8       TTCCTTTTACTTTTCCAGAGGAACACTGTGAATGGATGGGTGAGTCTCCCCTCCCAAATTAAAAATGTTGGGGTCTTCCTGTGAGAGCTGTGGGATGAGC
NTBGa-1(35/68,2)   ------------------.....................-------------------------------------------------------------
NTBGb(4/4,1)       ------------------.....................-------------------------------------------------------------
NTBGc(2/2,1)       ------------------.....................-------------------------------------------------------------
NTBGd(1/1,1)       ------------------.....................-------------------------------------------------------------
NTBGe(1/1,1)       ------------------.........T...........-------------------------------------------------------------
P2aTBGa-1(10/14,2) ------------------.....................-------------------------------------------------------------
P2aTBGb-1(2/6,2)   ------------------...C..........CA.....-------------------------------------------------------------
P2aTBGc-1(1/6,B)   ------------------..C.....ATG.C.GAAC...-------------------------------------------------------------
15iTBGa-1(6/24,2)  ------------------.....................-------------------------------------------------------------
15iTBGb-1(4/11,2)  ----------------------------------------------------------------------------------------------------
15iTBGc-1(3/4,1)   ------------------....C.........C......-------------------------------------------------------------
6TBGa-1(10/46,1)   ------------------..............CA.....-------------------------------------------------------------
6TBGc-1(6/11,B)    ------------------.....................-------------------------------------------------------------
6TBGb-1(7/15,B)    ------------------G.CT....ATG.C.GAAC...-------------------------------------------------------------
6TBGd(10/10,1)     ----------------------------------------------------------------------------------------------------
6TBGe(2/2,1)       ------------------G.CT....ATG.C.GAAC...-------------------------------------------------------------


                          4510      4520      4530      4540      4550      4560      4570      4580      4590      4600
                   ....|....|....|....|....|....|....|....|....|....|....|....|....|....|....|....|....|....|....|....|
KC955130_BG8       TGTTCCTCTCATCGTGCACTGTTTCTGCTTTTCCTTTGCAGTGAGAAGGAATGTAAAGTTGGGTGAGTCTTCTTCCCCAACCAAAGAGATTCGGAGTCTT
NTBGa-1(35/68,2)   -----------------------------------------.....................--------------------------------------
NTBGb(4/4,1)       -----------------------------------------.....................--------------------------------------
NTBGc(2/2,1)       -----------------------------------------.....................--------------------------------------
NTBGd(1/1,1)       -----------------------------------------.....................--------------------------------------
NTBGe(1/1,1)       -----------------------------------------A....................--------------------------------------
P2aTBGa-1(10/14,2) -----------------------------------------.....................--------------------------------------
P2aTBGb-1(2/6,2)   -----------------------------------------............C..G.....--------------------------------------
P2aTBGc-1(1/6,B)   -----------------------------------------..GATCAAGC..A.GCAG...--------------------------------------
15iTBGa-1(6/24,2)  -----------------------------------------................T....--------------------------------------
15iTBGb-1(4/11,2)  ----------------------------------------------------------------------------------------------------
15iTBGc-1(3/4,1)   ----------------------------------------------------------------------------------------------------
6TBGa-1(10/46,1)   -----------------------------------------A...........C........--------------------------------------
6TBGc-1(6/11,B)    -----------------------------------------.....................--------------------------------------
6TBGb-1(7/15,B)    -----------------------------------------..GATCAAGT..A.GCAG...--------------------------------------
6TBGd(10/10,1)     ----------------------------------------------------------------------------------------------------
6TBGe(2/2,1)       -----------------------------------------..GATCAAGT..A.GCAG...--------------------------------------


                          4610      4620      4630      4640      4650      4660      4670      4680      4690      4700
                   ....|....|....|....|....|....|....|....|....|....|....|....|....|....|....|....|....|....|....|....|
KC955130_BG8       CCATGGGATCAGCCATGGGATGATAACATGAACCTCATCACGTGTTTCTTATTTGTTCCTTTTGCAGAGGCAGCAGCTGTAAAAGTGGGTGAGTCTCCCC
NTBGa-1(35/68,2)   -------------------------------------------------------------------..AT.C...............------------
NTBGb(4/4,1)       -------------------------------------------------------------------..AT.C...............------------
NTBGc(2/2,1)       -------------------------------------------------------------------..AT.C...............------------
NTBGd(1/1,1)       -------------------------------------------------------------------..AT.C...............------------
NTBGe(1/1,1)       -------------------------------------------------------------------..AT.C.........T.C...------------
P2aTBGa-1(10/14,2) -------------------------------------------------------------------..AT.C...............------------
P2aTBGb-1(2/6,2)   -------------------------------------------------------------------.....................------------
P2aTBGc-1(1/6,B)   -------------------------------------------------------------------..AATCGGAAATC.G.GC..A------------
15iTBGa-1(6/24,2)  -------------------------------------------------------------------.....................------------
15iTBGb-1(4/11,2)  ----------------------------------------------------------------------------------------------------
15iTBGc-1(3/4,1)   ----------------------------------------------------------------------------------------------------
6TBGa-1(10/46,1)   -------------------------------------------------------------------.................C...------------
6TBGc-1(6/11,B)    -------------------------------------------------------------------.....................------------
6TBGb-1(7/15,B)    -------------------------------------------------------------------..AATCGGAAATC.G.GTG.A------------
6TBGd(10/10,1)     ----------------------------------------------------------------------------------------------------
6TBGe(2/2,1)       -------------------------------------------------------------------..AATCGGAAATC.G.GTG.A------------


                          4710      4720      4730      4740      4750      4760      4770      4780      4790      4800
                   ....|....|....|....|....|....|....|....|....|....|....|....|....|....|....|....|....|....|....|....|
KC955130_BG8       TCCCAAATTAAAAATGTTGGCGTCATCCTGTGAGAGCTGTGGGATGAGCTGTTCCTCTCATCGTGCACTGTTTCTGCTTTTCCTTTGCAGTGAGAAGGAA
NTBGa-1(35/68,2)   ----------------------------------------------------------------------------------------------------
NTBGb(4/4,1)       ----------------------------------------------------------------------------------------------------
NTBGc(2/2,1)       ----------------------------------------------------------------------------------------------------
NTBGd(1/1,1)       ----------------------------------------------------------------------------------------------------
NTBGe(1/1,1)       ----------------------------------------------------------------------------------------------------
P2aTBGa-1(10/14,2) ------------------------------------------------------------------------------------------..........
P2aTBGb-1(2/6,2)   ----------------------------------------------------------------------------------------------------
P2aTBGc-1(1/6,B)   ----------------------------------------------------------------------------------------------------
15iTBGa-1(6/24,2)  ------------------------------------------------------------------------------------------..........
15iTBGb-1(4/11,2)  ----------------------------------------------------------------------------------------------------
15iTBGc-1(3/4,1)   ----------------------------------------------------------------------------------------------------
6TBGa-1(10/46,1)   ----------------------------------------------------------------------------------------------------
6TBGc-1(6/11,B)    ------------------------------------------------------------------------------------------..........
6TBGb-1(7/15,B)    ----------------------------------------------------------------------------------------------------
6TBGd(10/10,1)     ----------------------------------------------------------------------------------------------------
6TBGe(2/2,1)       ----------------------------------------------------------------------------------------------------
```

```
                            4810      4820      4830      4840      4850      4860      4870      4880      4890      4900
                   ....|....|....|....|....|....|....|....|....|....|....|....|....|....|....|....|....|....|....|....|
KC955130_BG8       TGTAAAGTTGGGTGAGTCTTCTTCCCCAACCAAAGAGATGTGGGGTCTTCCATGGGATCAGCCATGGGATGATAAGCTGAACCTTATCACGTGTTTCTTA
NTBGa-1(35/68,2)   ----------------------------------------------------------------------------------------------------
NTBGb(4/4,1)       ----------------------------------------------------------------------------------------------------
NTBGc(2/2,1)       ----------------------------------------------------------------------------------------------------
NTBGd(1/1,1)       ----------------------------------------------------------------------------------------------------
NTBGe(1/1,1)       ----------------------------------------------------------------------------------------------------
P2aTBGa-1(10/14,2) ...........-----------------------------------------------------------------------------------------
P2aTBGb-1(2/6,2)   ----------------------------------------------------------------------------------------------------
P2aTBGc-1(1/6,B)   ----------------------------------------------------------------------------------------------------
15iTBGa-1(6/24,2)  ...........-----------------------------------------------------------------------------------------
15iTBGb-1(4/11,2)  ----------------------------------------------------------------------------------------------------
15iTBGc-1(3/4,1)   ----------------------------------------------------------------------------------------------------
6TBGa-1(10/46,1)   ----------------------------------------------------------------------------------------------------
6TBGc-1(6/11,B)    ...........-----------------------------------------------------------------------------------------
6TBGb-1(7/15,B)    ----------------------------------------------------------------------------------------------------
6TBGd(10/10,1)     ----------------------------------------------------------------------------------------------------
6TBGe(2/2,1)       ----------------------------------------------------------------------------------------------------

                            4910      4920      4930      4940      4950      4960      4970      4980      4990      5000
                   ....|....|....|....|....|....|....|....|....|....|....|....|....|....|....|....|....|....|....|....|
KC955130_BG8       TTTGTTCCTTTTGCAGAGGCAGCAGCTGTAAAAGTGGGTGAGTCCTCCCTCCCAAATCAAATACAAAAGGGGATCTGCCTGTGTGAGCTGTGGGATGAGA
NTBGa-1(35/68,2)   ----------------------------------------------------------------------------------------------------
NTBGb(4/4,1)       ----------------------------------------------------------------------------------------------------
NTBGc(2/2,1)       ----------------------------------------------------------------------------------------------------
NTBGd(1/1,1)       ----------------------------------------------------------------------------------------------------
NTBGe(1/1,1)       ----------------------------------------------------------------------------------------------------
P2aTBGa-1(10/14,2) ----------------..AT.C...............---------------------------------------------------------------
P2aTBGb-1(2/6,2)   ----------------------------------------------------------------------------------------------------
P2aTBGc-1(1/6,B)   ----------------------------------------------------------------------------------------------------
15iTBGa-1(6/24,2)  ----------------.....................---------------------------------------------------------------
15iTBGb-1(4/11,2)  ----------------------------------------------------------------------------------------------------
15iTBGc-1(3/4,1)   ----------------------------------------------------------------------------------------------------
6TBGa-1(10/46,1)   ----------------------------------------------------------------------------------------------------
6TBGc-1(6/11,B)    ----------------.....................---------------------------------------------------------------
6TBGb-1(7/15,B)    ----------------------------------------------------------------------------------------------------
6TBGd(10/10,1)     ----------------------------------------------------------------------------------------------------
6TBGe(2/2,1)       ----------------------------------------------------------------------------------------------------

                            5010      5020      5030      5040      5050      5060      5070      5080      5090      5100
                   ....|....|....|....|....|....|....|....|....|....|....|....|....|....|....|....|....|....|....|....|
KC955130_BG8       TGTTCCTCTCATCACGCATTGTTTTTCTCATTCATTTCCAGGACACAAAGCTAAAGAATCAGGTGAGTCTTCTTCCCTGTCCCAAAGGACTATGGGTTTC
NTBGa-1(35/68,2)   -----------------------------------------.G..AC...............--------------------------------------
NTBGb(4/4,1)       -----------------------------------------.G..AC...............--------------------------------------
NTBGc(2/2,1)       -----------------------------------------.G..AC...............--------------------------------------
NTBGd(1/1,1)       -----------------------------------------.G..AC...............--------------------------------------
NTBGe(1/1,1)       -----------------------------------------.G..AC...............--------------------------------------
P2aTBGa-1(10/14,2) -----------------------------------------.G..AC...............--------------------------------------
P2aTBGb-1(2/6,2)   -----------------------------------------.....................--------------------------------------
P2aTBGc-1(1/6,B)   ----------------------------------------------------------------------------------------------------
15iTBGa-1(6/24,2)  -----------------------------------------.....................--------------------------------------
15iTBGb-1(4/11,2)  -----------------------------------------.G..AC...............--------------------------------------
15iTBGc-1(3/4,1)   ----------------------------------------------------------------------------------------------------
6TBGa-1(10/46,1)   -----------------------------------------.....................--------------------------------------
6TBGc-1(6/11,B)    -----------------------------------------.....................--------------------------------------
6TBGb-1(7/15,B)    ----------------------------------------------------------------------------------------------------
6TBGd(10/10,1)     ----------------------------------------------------------------------------------------------------
6TBGe(2/2,1)       ----------------------------------------------------------------------------------------------------

                            5110      5120      5130      5140      5150      5160      5170      5180      5190      5200
                   ....|....|....|....|....|....|....|....|....|....|....|....|....|....|....|....|....|....|....|....|
KC955130_BG8       CCATGGGATGACAAGCTGTGCCACCTCCTCATGAGGTGCTTCTTCTTTCTTTGTGCAGAGAAACAGAAATCGGAGCTGAGTAAGTTGCAGTCACTGAACT
NTBGa-1(35/68,2)   ----------------------------------------------------------..G..................---------------------
NTBGb(4/4,1)       ----------------------------------------------------------..G..................---------------------
NTBGc(2/2,1)       ----------------------------------------------------------..G..................---------------------
NTBGd(1/1,1)       ----------------------------------------------------------..G....................................A.
NTBGe(1/1,1)       ----------------------------------------------------------.....................---------------------
P2aTBGa-1(10/14,2) ----------------------------------------------------------.....................---------------------
P2aTBGb-1(2/6,2)   ----------------------------------------------------------.....................---------------------
P2aTBGc-1(1/6,B)   ----------------------------------------------------------------------------------------------------
15iTBGa-1(6/24,2)  ----------------------------------------------------------.....................---------------------
15iTBGb-1(4/11,2)  ----------------------------------------------------------..................C..---------------------
15iTBGc-1(3/4,1)   ----------------------------------------------------------T..G.AG...TGTAA..T..G---------------------
6TBGa-1(10/46,1)   ----------------------------------------------------------------------------------------------------
6TBGc-1(6/11,B)    ----------------------------------------------------------.....................---------------------
6TBGb-1(7/15,B)    ----------------------------------------------------------------------------------------------------
6TBGd(10/10,1)     ----------------------------------------------------------------------------------------------------
6TBGe(2/2,1)       ----------------------------------------------------------------------------------------------------
```

```
                            5210      5220      5230      5240      5250      5260      5270      5280      5290      5300
                    ....|....|....|....|....|....|....|....|....|....|....|....|....|....|....|....|....|....|....|....|
KC955130_BG8        GAGGGAATGTGGGGTCTTCCCAAAGTCCTGCGTATGGGATGAAAAATCCCCTCTGACCATGCACTGCTTTTCTCCTCCTTTGCCAGAGGAGCGCCATGAG
NTBGa-1(35/68,2)    --------------------------------------------------------------------------------------......A.......
NTBGb(4/4,1)        --------------------------------------------------------------------------------------......A.......
NTBGc(2/2,1)        --------------------------------------------------------------------------------------......A.......
NTBGd(1/1,1)        ............................................................................................A.......
NTBGe(1/1,1)        --------------------------------------------------------------------------------------..............
P2aTBGa-1(10/14,2)  --------------------------------------------------------------------------------------..............
P2aTBGb-1(2/6,2)    --------------------------------------------------------------------------------------..............
P2aTBGc-1(1/6,B)    --------------------------------------------------------------------------------------..AGATATTTGACA
15iTBGa-1(6/24,2)   --------------------------------------------------------------------------------------..............
15iTBGb-1(4/11,2)   --------------------------------------------------------------------------------------..............
15iTBGc-1(3/4,1)    --------------------------------------------------------------------------------------..ATA.CAGC..TA
6TBGa-1(10/46,1)    ----------------------------------------------------------------------------------------------------
6TBGc-1(6/11,B)     --------------------------------------------------------------------------------------..............
6TBGb-1(7/15,B)     --------------------------------------------------------------------------------------..AGATATTTGACA
6TBGd(10/10,1)      ----------------------------------------------------------------------------------------------------
6TBGe(2/2,1)        --------------------------------------------------------------------------------------..AGATATTTGACA


                            5310      5320      5330      5340      5350      5360      5370      5380      5390      5400
                    ....|....|....|....|....|....|....|....|....|....|....|....|....|....|....|....|....|....|....|....|
KC955130_BG8        GAGATGGGTGAGTCTCCCCTCCCATATTAAAATCGTTGGGGTCTTCCTGTGTGAGCTGTGGGATGAGATGTTCCTCTCATCACACATTGTTTTTCTTTTC
NTBGa-1(35/68,2)    ....C..---------------------------------------------------------------------------------------------
NTBGb(4/4,1)        ....C..---------------------------------------------------------------------------------------------
NTBGc(2/2,1)        ....C..---------------------------------------------------------------------------------------------
NTBGd(1/1,1)        ....C..---------------------------------------------------------------------------------------------
NTBGe(1/1,1)        .......---------------------------------------------------------------------------------------------
P2aTBGa-1(10/14,2)  .......---------------------------------------------------------------------------------------------
P2aTBGb-1(2/6,2)    .......---------------------------------------------------------------------------------------------
P2aTBGc-1(1/6,B)    A.T..A.---------------------------------------------------------------------------------------------
15iTBGa-1(6/24,2)   .......---------------------------------------------------------------------------------------------
15iTBGb-1(4/11,2)   .......---------------------------------------------------------------------------------------------
15iTBGc-1(3/4,1)    A.AG...---------------------------------------------------------------------------------------------
6TBGa-1(10/46,1)    ----------------------------------------------------------------------------------------------------
6TBGc-1(6/11,B)     .......---------------------------------------------------------------------------------------------
6TBGb-1(7/15,B)     A.T..A.---------------------------------------------------------------------------------------------
6TBGd(10/10,1)      ----------------------------------------------------------------------------------------------------
6TBGe(2/2,1)        A.T..A.---------------------------------------------------------------------------------------------


                            5410      5420      5430      5440      5450      5460      5470      5480      5490      5500
                    ....|....|....|....|....|....|....|....|....|....|....|....|....|....|....|....|....|....|....|....|
KC955130_BG8        CAGGGCAACAAGCTAAAGAATCAGGTGAGTCTTCTTCCCCGTCCCAAAGGACTATGGGTTTCCCATGGGATGACAAGCTGTGCCACCTCCTCATGAGGTG
NTBGa-1(35/68,2)    ---.....................----------------------------------------------------------------------------
NTBGb(4/4,1)        ---.....................----------------------------------------------------------------------------
NTBGc(2/2,1)        ---.....................----------------------------------------------------------------------------
NTBGd(1/1,1)        ---.....................----------------------------------------------------------------------------
NTBGe(1/1,1)        ----------------------------------------------------------------------------------------------------
P2aTBGa-1(10/14,2)  ---.....................----------------------------------------------------------------------------
P2aTBGb-1(2/6,2)    ----------------------------------------------------------------------------------------------------
P2aTBGc-1(1/6,B)    ---.TTT..GT...GC...GCTGA----------------------------------------------------------------------------
15iTBGa-1(6/24,2)   ---.....................----------------------------------------------------------------------------
15iTBGb-1(4/11,2)   ----------------------------------------------------------------------------------------------------
15iTBGc-1(3/4,1)    ---...................G.----------------------------------------------------------------------------
6TBGa-1(10/46,1)    ----------------------------------------------------------------------------------------------------
6TBGc-1(6/11,B)     ---.....................----------------------------------------------------------------------------
6TBGb-1(7/15,B)     ---.TTT..GT...GC...GCTGA----------------------------------------------------------------------------
6TBGd(10/10,1)      ----------------------------------------------------------------------------------------------------
6TBGe(2/2,1)        ---.TTT..GT...GC...GCTGA----------------------------------------------------------------------------


                            5510      5520      5530      5540      5550      5560      5570      5580      5590      5600
                    ....|....|....|....|....|....|....|....|....|....|....|....|....|....|....|....|....|....|....|....|
KC955130_BG8        CTTCTTCTTTCTTTTTTGCAGAGAAACAGAAATCGGAGCTGAGTAAGTTGCAGTCACTGAACTGAGGGTATTTGGGGTCCTTTCAAGGGACTGTGTATGG
NTBGa-1(35/68,2)    ---------------------.....................----------------------------------------------------------
NTBGb(4/4,1)        ---------------------.....................----------------------------------------------------------
NTBGc(2/2,1)        ---------------------.....................----------------------------------------------------------
NTBGd(1/1,1)        ---------------------.....................----------------------------------------------------------
NTBGe(1/1,1)        ----------------------------------------------------------------------------------------------------
P2aTBGa-1(10/14,2)  ---------------------.....................----------------------------------------------------------
P2aTBGb-1(2/6,2)    ----------------------------------------------------------------------------------------------------
P2aTBGc-1(1/6,B)    ---------------------.A...T.CGTTG.A..A....----------------------------------------------------------
15iTBGa-1(6/24,2)   ---------------------.....................----------------------------------------------------------
15iTBGb-1(4/11,2)   ----------------------------------------------------------------------------------------------------
15iTBGc-1(3/4,1)    ----------------------------------------------------------------------------------------------------
6TBGa-1(10/46,1)    ---------------------.....................----------------------------------------------------------
6TBGc-1(6/11,B)     ---------------------.....................----------------------------------------------------------
6TBGb-1(7/15,B)     ---------------------.A...A.CGTTG.A..A....----------------------------------------------------------
6TBGd(10/10,1)      ----------------------------------------------------------------------------------------------------
6TBGe(2/2,1)        ---------------------.A...A.CGTTG.A..A....----------------------------------------------------------
```

```
                          5610      5620      5630      5640      5650      5660      5670      5680      5690      5700
                    ....|....|....|....|....|....|....|....|....|....|....|....|....|....|....|....|....|....|....|....|
KC955130_BG8        GATGAAAAATCCCCTCTGACCATGCACTGCTTTTCTCCTTCTTTGCCAGAGGAGCGCCATGAGGAGATGGGTGAGTCTCCCCTCCCATATTAAAATCGTT
NTBGa-1(35/68,2)    -------------------------------------------------.....................------------------------------
NTBGb(4/4,1)        -------------------------------------------------.....................------------------------------
NTBGc(2/2,1)        -------------------------------------------------.....................------------------------------
NTBGd(1/1,1)        -------------------------------------------------.....................------------------------------
NTBGe(1/1,1)        ----------------------------------------------------------------------------------------------------
P2aTBGa-1(10/14,2)  -------------------------------------------------.....................------------------------------
P2aTBGb-1(2/6,2)    ----------------------------------------------------------------------------------------------------
P2aTBGc-1(1/6,B)    -------------------------------------------------..A.A..AAC...A.CAT...------------------------------
15iTBGa-1(6/24,2)   -------------------------------------------------......A..............------------------------------
15iTBGb-1(4/11,2)   ----------------------------------------------------------------------------------------------------
15iTBGc-1(3/4,1)    -------------------------------------------------...G.................------------------------------
6TBGa-1(10/46,1)    -------------------------------------------------.....................------------------------------
6TBGc-1(6/11,B)     -------------------------------------------------.....................------------------------------
6TBGb-1(7/15,B)     -------------------------------------------------..A.A..AAT..GA.CAT...------------------------------
6TBGd(10/10,1)      ----------------------------------------------------------------------------------------------------
6TBGe(2/2,1)        -------------------------------------------------..A.A..AAT..GA.CAT...------------------------------

                          5710      5720      5730      5740      5750      5760      5770      5780      5790      5800
                    ....|....|....|....|....|....|....|....|....|....|....|....|....|....|....|....|....|....|....|....|
KC955130_BG8        GGGGTCTTCCTGTGTGAGCTGTGGGATGAGATGTTCCTCTCATCGTGTGGTGCTTTTCTCTCTTTTCCAGCAGAACAAACTGAAGCAGTGGGTGAGTCTT
NTBGa-1(35/68,2)    ----------------------------------------------------------------------.....................---------
NTBGb(4/4,1)        -------------------------------------------------------------------------..................---------
NTBGc(2/2,1)        ----------------------------------------------------------------------.....................---------
NTBGd(1/1,1)        -------------------------------------------------------------------------..................---------
NTBGe(1/1,1)        -------------------------------------------------------------------------..................---------
P2aTBGa-1(10/14,2)  ----------------------------------------------------------------------.....................---------
P2aTBGb-1(2/6,2)    ----------------------------------------------------------------------.....................---------
P2aTBGc-1(1/6,B)    ----------------------------------------------------------------------------------------------------
15iTBGa-1(6/24,2)   ----------------------------------------------------------------------.....................---------
15iTBGb-1(4/11,2)   ----------------------------------------------------------------------........T............---------
15iTBGc-1(3/4,1)    ----------------------------------------------------------------------.....................---------
6TBGa-1(10/46,1)    ----------------------------------------------------------------------.....................---------
6TBGc-1(6/11,B)     ----------------------------------------------------------------------.....................---------
6TBGb-1(7/15,B)     ----------------------------------------------------------------------------.....A...A.T...---------
6TBGd(10/10,1)      ----------------------------------------------------------------------------------------------------
6TBGe(2/2,1)        ----------------------------------------------------------------------------------------------------

                          5810      5820      5830      5840      5850      5860      5870      5880      5890      5900
                    ....|....|....|....|....|....|....|....|....|....|....|....|....|....|....|....|....|....|....|....|
KC955130_BG8        TGTCCCCAAACCAAAGGAATATGGGGCAATCCATGGGATGACAAGCTGTCCCATCTCATCATGTGTTGCTTTTCTATTCCTTTTTTCCAGTGGTAGAAAC
NTBGa-1(35/68,2)    ------------------------------------------------------------------------------------------..........
NTBGb(4/4,1)        ------------------------------------------------------------------------------------------..........
NTBGc(2/2,1)        ------------------------------------------------------------------------------------------..........
NTBGd(1/1,1)        ------------------------------------------------------------------------------------------..........
NTBGe(1/1,1)        ------------------------------------------------------------------------------------------..........
P2aTBGa-1(10/14,2)  ------------------------------------------------------------------------------------------..........
P2aTBGb-1(2/6,2)    ------------------------------------------------------------------------------------------..........
P2aTBGc-1(1/6,B)    ------------------------------------------------------------------------------------------------....
15iTBGa-1(6/24,2)   ------------------------------------------------------------------------------------------..........
15iTBGb-1(4/11,2)   ------------------------------------------------------------------------------------------.......T.A
15iTBGc-1(3/4,1)    ------------------------------------------------------------------------------------------...G....G.
6TBGa-1(10/46,1)    ------------------------------------------------------------------------------------------..........
6TBGc-1(6/11,B)     ------------------------------------------------------------------------------------------..........
6TBGb-1(7/15,B)     ----------------------------------------------------------------------------------------------------
6TBGd(10/10,1)      ----------------------------------------------------------------------------------------------------
6TBGe(2/2,1)        ------------------------------------------------------------------------------------------------....

                          5910      5920      5930      5940      5950      5960      5970      5980      5990      6000
                    ....|....|....|....|....|....|....|....|....|....|....|....|....|....|....|....|....|....|....|....|
KC955130_BG8        TGAAGAATAGGGTGAGTCTTTCCCAAACCAAAGCAATACAGGGTTTCCCATGGGATGACAAGCTGTCCCACCTCAGCATCCGTTCCTTTTTATTTCTTTT
NTBGa-1(35/68,2)    ...........-----------------------------------------------------------------------------------------
NTBGb(4/4,1)        ...........-----------------------------------------------------------------------------------------
NTBGc(2/2,1)        ...........-----------------------------------------------------------------------------------------
NTBGd(1/1,1)        ...........-----------------------------------------------------------------------------------------
NTBGe(1/1,1)        ...........-----------------------------------------------------------------------------------------
P2aTBGa-1(10/14,2)  ......T....-----------------------------------------------------------------------------------------
P2aTBGb-1(2/6,2)    .......GC..-----------------------------------------------------------------------------------------
P2aTBGc-1(1/6,B)    .A......C..-----------------------------------------------------------------------------------------
15iTBGa-1(6/24,2)   ........C..-----------------------------------------------------------------------------------------
15iTBGb-1(4/11,2)   ........C..-------------------------- ---------------------------------------------------------------
15iTBGc-1(3/4,1)    ........T..non..........................A..C.......................G......G.....C-........
6TBGa-1(10/46,1)    ...........-----------------------------------------------------------------------------------------
6TBGc-1(6/11,B)     ....................................................................................................
6TBGb-1(7/15,B)     ----------------------------------------------------------------------------------------------------
6TBGd(10/10,1)      ----------------------------------------------------------------------------------------------------
6TBGe(2/2,1)        .A......T..-----------------------------------------------------------------------------------------
```

```
                            6010      6020      6030      6040      6050      6060      6070      6080      6090      6100
                    ....|....|....|....|....|....|....|....|....|....|....|....|....|....|....|....|....|....|....|....|
KC955130_BG8        CCAGAAAAACCATCTGAAGAATCAGATTGAGAGATGAACTGCGCCTCGCAATAAGCACAGGAGTTAAGCTTCATAGATCAATAACTGCACAGCATACAAA
NTBGa-1(35/68,2)    ----..................TG..........................G...C.............................................
NTBGb(4/4,1)        ----..................TG..........................G...C.............................................
NTBGc(2/2,1)        ----..................TG..........................G...C.............................................
NTBGd(1/1,1)        ----..................TG..........................G...C.............................................
NTBGe(1/1,1)        ----..................TG..........................G...C.............................................
P2aTBGa-1(10/14,2)  ----.........G......................................................................................
P2aTBGb-1(2/6,2)    ----...........................................A..G...C....................C..........A.........A...
P2aTBGc-1(1/6,B)    ----..C........A.....CAG.......................A..G...C......................CTGC.G.T.......G...GC..
15iTBGa-1(6/24,2)   ----...........................................A....................................................
15iTBGb-1(4/11,2)   ----.GG........T......GG.......................A..G...C....................C..........A.........-...
15iTBGc-1(3/4,1)    ............G.........GG.......................A..G...C........A.............CTGC....A......G...GT..
6TBGa-1(10/46,1)    ----...........................................A........................T...........................
6TBGc-1(6/11,B)     ....................................................................................................
6TBGb-1(7/15,B)     ----...........A.....CAG.......................A..G...C........A.............CTGC.G.T.......G...GC..
6TBGd(10/10,1)      ----------------------------------------------------------------------------------------------------
6TBGe(2/2,1)        ----...........A.....CAG.......................A..G...C........A.............CTGC.G.T.......G...GC..


                            6110      6120      6130      6140      6150      6160      6170      6180      6190      6200
                    ....|....|....|....|....|....|....|....|....|....|....|....|....|....|....|....|....|....|....|....|
KC955130_BG8        A-CCACAATAACTCAAACAGAGTAAGGA--------------------GGAGCCAGTGTTTGTGTTGAGTGAGAACACTGCAGTTCTGTCAGCCAAAGCT
NTBGa-1(35/68,2)    .-..................G.......--------------------....................................................
NTBGb(4/4,1)        .-..................G.......--------------------....................................................
NTBGc(2/2,1)        .-..................G.......--------------------....................................................
NTBGd(1/1,1)        .-..................G.......--------------------....................................................
NTBGe(1/1,1)        .-..................G.......--------------------....................................................
P2aTBGa-1(10/14,2)  .-..................G.......--------------------......................................C..G..........
P2aTBGb-1(2/6,2)    .-....G..TC.A.........C.....AATCCAGAGCGAGAAAAAGA....................................................
P2aTBGc-1(1/6,B)    CAT.G.C.....G....GCA..C.....AATCCACACGGGGAACAAGA..........CC...A........................CA.....C....
15iTBGa-1(6/24,2)   .-....................C.....--------------------...............A....................................
15iTBGb-1(4/11,2)   .A....G..TC.A.........C.....AATCCACAGTGAGAACAAGA.............................G......................
15iTBGc-1(3/4,1)    CA..G.TG....G....AT...C.....AACCCAAAGCGAGAACAAGA.........................................G..........
6TBGa-1(10/46,1)    .-..........................--------------------....................................................
6TBGc-1(6/11,B)     .-..........................--------------------....................................................
6TBGb-1(7/15,B)     CAT.G.C.....G....GCA..A.....AATCCACACGGGGAACAAGA...............A........................CA.....C.A..
6TBGd(10/10,1)      ----------------------------------------------------------------------------------------------------
6TBGe(2/2,1)        CAT.G.C.....G....GCA..A.....AATCCACACGGGGAACAAGA...............A........................CA.....C.A..


                            6210      6220      6230      6240      6250      6260      6270      6280      6290      6300
                    ....|....|....|....|....|....|....|....|....|....|....|....|....|....|....|....|....|....|....|....|
KC955130_BG8        GCCTGAGGGACCGCCGAATTGAGGGTGTGCGACCTCCAACTCAAAGCCAATTGGAAGAAAGAAACCATAGAAA-GGAAGGAAAGGGGAGGGAGACAGAGA
NTBGa-1(35/68,2)    ...............C.........................................................-.....A..........A.........
NTBGb(4/4,1)        ...............C.........................................................-.....A..........A.........
NTBGc(2/2,1)        ...............C.........................................................-.....A..........A.........
NTBGd(1/1,1)        ...............C.........................................................-.....A..........A.........
NTBGe(1/1,1)        ...............C.........................................................-.....A..........A.........
P2aTBGa-1(10/14,2)  ...............C.........................................................-......C.........A.........
P2aTBGb-1(2/6,2)    ............AGA....................................................C.A...-..........................
P2aTBGc-1(1/6,B)    ............A..A..C..........T........T......T...G.......................-A....ACT.CAA....A.........
15iTBGa-1(6/24,2)   ...............C.............................T...G.......................-................A.........
15iTBGb-1(4/11,2)   ...............C.............................T...G.......................-................A.........
15iTBGc-1(3/4,1)    ......CA.......C.........................................................G.....ACT.CAA....A.........
6TBGa-1(10/46,1)    ...............C.............T...............T...G.......................-................A.........
6TBGc-1(6/11,B)     .........................................................................-..........................
6TBGb-1(7/15,B)     ............A..A..C..........T........T......T...G.......................-A....ACT.CAA....A.........
6TBGd(10/10,1)      ----------------------------------------------------------------------------------------------------
6TBGe(2/2,1)        ............A..A..C..........T........T......T...G.......................-A....ACT.CAA....A.........


                            6310      6320      6330      6340      6350      6360      6370      6380      6390      6400
                    ....|....|....|....|....|....|....|....|....|....|....|....|....|....|....|....|....|....|....|....|
KC955130_BG8        TCCTGGAAGAGATATGGGCATTTGGGGAAATAGTGTGACCGTGTATCAGGCTTTGTGGACATCTAACGAATATGTCATGTTTTTGTAAATACAAGCATGC
NTBGa-1(35/68,2)    ........................................A...........................................................
NTBGb(4/4,1)        .................................C......A...........................................................
NTBGc(2/2,1)        ........................................A...........................................................
NTBGd(1/1,1)        ........................................A...........................................................
NTBGe(1/1,1)        ........................................A.............................................G.............
P2aTBGa-1(10/14,2)  ....................................................................................................
P2aTBGb-1(2/6,2)    ........................................A........................................................-..
P2aTBGc-1(1/6,B)    .....A..A..GG.CA.A.....T.....T..ACA..G..A.........G...TA...AT.....T........A.G.C..C..G.....T..A.....
15iTBGa-1(6/24,2)   ........................................................................................C...........
15iTBGb-1(4/11,2)   ........................................A...........................................................
15iTBGc-1(3/4,1)    ...................................................................A....................C...........
6TBGa-1(10/46,1)    ........................................A.........................T............C........C...........
6TBGc-1(6/11,B)     ....................................................................................................
6TBGb-1(7/15,B)     ......G.A..GG.CA.A.....T.....T..ACA..G..A.........GG-..A...AT.....T........A.G.C..C..G.....T..A.....
6TBGd(10/10,1)      ----------------------------------------------------------------------------------------------------
6TBGe(2/2,1)        ......G.A..GG.CA.A.....T.....T..ACA..G..A.........GG-..A...AT.....T........A.G.C..C..G.....T..A.....
```

```
                   'U' primer  5' CNTCTTTTGACGTTTCCACAAT 3'
                    (UC650)

                                 <-------------------
                                 GNAGAAAACTGCTTTGGTGTTA
                            6410      6420      6430      6440      6450      6460
                   ....|....|....|....|....|....|....|....|....|....|....|....|...
KC955130_BG8       ACGCAGAAACAAAGGTAGAAAACTGCTTTGGGTGTTAGCACTGTTCTCTGTCCCTATATAATA
NTBGa-1(35/68,2)   ...............G.....................--------------------------
NTBGb(4/4,1)       ...............G.....................--------------------------
NTBGc(2/2,1)       ...............G.....................--------------------------
NTBGd(1/1,1)       ...............G.....................--------------------------
NTBGe(1/1,1)       ...............G.....................--------------------------
P2aTBGa-1(10/14,2) ...............C.....................--------------------------
P2aTBGb-1(2/6,2)   ...............C.....................--------------------------
P2aTBGc-1(1/6,B)   ..A.....GT.....C.....................--------------------------
15iTBGa-1(6/24,2)  ...............C.....................--------------------------
15iTBGb-1(4/11,2)  .T.............C.....................--------------------------
15iTBGc-1(3/4,1)   .T.............G.....................--------------------------
6TBGa-1(10/46,1)   ...............C.....................--------------------------
6TBGc-1(6/11,B)    ...............C.....................--------------------------
6TBGb-1(7/15,B)    ..A.....GT...........................--------------------------
6TBGd(10/10,1)     ---------------------------------------------------------------
6TBGe(2/2,1)       ..A.....GT.....C.....................--------------------------
```

Supplementary Fig. S2. Alignment of nucleotide cDNA sequences from the 16 genes (based on exon 2 sequences) from four chicken lines identified in this paper against the BG8 gene (including introns) of the B12 haplotype from Salomonsen et al 2014. Names of the transcripts follow the convention: abbreviated line name, "T" for T cells, "BG" and the letter "a" representing the most frequently detected clone from the most frequently detected exon 2 sequence (and "b" representing the most frequently detected clone from the second most frequently detected exon 2 sequence, and so forth). Numbers in parenthesis indicate the number of times the depicted sequence was found out of the number of times the gene (based on the exon 2 sequence) was found, followed by the number of independent PCRs in which the sequence was identified (1, found in one PCR; 2 found in 2 PCRs; B, found in one PCR described in this paper and one using B cell cDNA, data not shown). Names of the genes follow the convention "BG" and the number of the gene locus for the B12 haplotype. Colors indicate different coding regions (grey, 5'UTR and 3'UTR; dark green, signal sequence; light green, Ig-V domain; brown, transmembrane region; alternating yellow and red, cytoplasmic tail regions (codons from each 18, 21 or 24 nucleotide repeat, with nucleotides in the split codon indicated by the color of the exon in which the majority of the codon is located); purple, in-frame stop codon; light blue, introns (including two positions that are probably nucleotide mis-incorporations during PCR reaction) which are all deleted in the analyses for "(nearly) full-length conceptual transcripts" (that is, exons without introns). Letters indicate nucleotides, dot indicates identity with BG8 sequence; dash indicates not present in all sequences. Arrows indicate primers with sequences (so that sequence differences in these positions are not necessarily real).

1.3 Supplementary Figure S3

```
                        10        20        30        40        50        60        70        80        90       100
                ....|....|....|....|....|....|....|....|....|....|....|....|....|....|....|....|....|....|....|....|
BG8             TCCGCTCGAGCTCTCTC--CTCCTACAGCTTCTGCCCTCATATTCTCCCCACACTTCTTCCCCATATTCTTTCCAAATCCTCTT----------------
P2aTBGa         ................T--.............................A..G..C.............................----------------
NTBGa           .................--.........T.......................................................----------------
15iTBGa         ................T--...........G.....................................................----------------
BG9             .................--.................................................................----------------
BG12            .................--.................................................................----------------
BG13            ............T...T--.........T..T...........A.............---........................----------------
6TBGa           ................T--.........T..T...........A.............---........................----------------
BG6             C..T..G.GC.C.....--...........C..T..TG........T..TCA....T...TAA..C.............T....CCCCATCTGCTCCGGC
BG3             C..T..GAGC.C.....--...........C..T..TG........T..TGA....T...TAA..C.............T....CCCCATCTGCTCCAGC
BG4             C..T..G.GC.C.....TT..............TT...........T.A..A.A..T....A...CACA..........T....CCCCATCTTCTCCAGC
BG5             ................T--.........T.......................................................----------------
BG7             .................--..........C.....................T................................----------------
BG11            .................--..........C.....................T................................----------------
BG10            C..T..G.GC.C.....--....C......C..T..TG........T..TGA....T...TAA..C.............T....CCCCGTCTTCTCCAGC
BG2             C..T..G.GC.C.....--...........C..T..TG......A.T..TGA....T...TAA..C.............T....CCCCATCTGCTCCAGC
BG1             A..CTCT.GC.A.....TT.........T....T.....C....T....TG-.TC.T........C............AT..ACCCCCATCTTCTCCATC
BG0             -----------------------------------------------------------------------------GGGCACG----------------

                       110       120       130       140       150       160       170       180       190       200
                ....|....|....|....|....|....|....|....|....|....|....|....|....|....|....|....|....|....|....|....|
BG8             ----------------------------------------------------------------------------------------------------
P2aTBGa         ----------------------------------------------------------------------------------------------------
NTBGa           ----------------------------------------------------------------------------------------------------
15iTBGa         ----------------------------------------------------------------------------------------------------
BG9             ----------------------------------------------------------------------------------------------------
BG12            ----------------------------------------------------------------------------------------------------
BG13            ----------------------------------------------------------------------------------------------------
6TBGa           ----------------------------------------------------------------------------------------------------
BG6             ACCTCCTTCTCCATCTCCTTCCCCAAACTCCTCCTTGTATCCCCTTCCCCAATCTCCTTCCCCCACCACCTTCTCCTATCATCTTCTCTCATCTTTTACC
BG3             ACCTCCTTCTCCATCTCCTTCCCCAAACTCCTCCTTGTATCCCCTTCCCCAATCTCCTTCTCCCACCTCCTTTTCCTATCATCT---CTCATTTTTAACC
BG4             ACCTCCTTCTCCATCATCTTCCTCAATCCCCTTC------------------------------CCCACCTTCTTCCCTTGTCTTCTCTCATCTTTTACC
BG5             ----------------------------------------------------------------------------------------------------
BG7             ----------------------------------------------------------------------------------------------------
BG11            ----------------------------------------------------------------------------------------------------
BG10            ACCTCCTTCTCCATCTCCTTCCCCAAACTCCTCCTTGTATCCCCTTCCCCAATCTCCTTCTCCCACCTCCTTCTCCTATCATCTTCTCTCATCCTTTACC
BG2             ACCTCCTTCTCAGTCTCCTTCCCCAAACTCCGCCTGTTATCCCCTTCCCCAATCTCCTTCTCCCACCTCCTTCTCCTATCATTTTATCTCATCTTTTACC
BG1             ATCTCCTTCTCCATCTCCTTCCA---------------------------------------CCACTTCCTTCCCCTATCTTCGTCTCTCATCTTTTACC
BG0             ----------------------------------------------------------------------------------------------------

                       210       220       230       240       250       260       270       280       290       300
                ....|....|....|....|....|....|....|....|....|....|....|....|....|....|....|....|....|....|....|....|
BG8             --------------------CCCCATCTCCT--CCACCGTCTCCTTCTCAGAGTCCTTCCTCTCTCTCCCT-----AAATTCT---TCCCCCCTCCTCTT
P2aTBGa         --------------------...........--......................................-----.......---..............
NTBGa           --------------------...........--...T..................................-----.......---..............
15iTBGa         --------------------...........--...T..................................-----.......---...T..........
BG9             --------------------...........--......................................-----.......---...T..........
BG12            --------------------...........--..........T......C....................-----.......---..............
BG13            --------------------........T..--.....A............TA..................-----.......---..............
6TBGa           --------------------........T..--.....A............TA..................-----.......---..............
BG6             CATTTTCTACCCACATTCTG...........--...T.A............TC................T.TCCCC..C.C..---..............
BG3             CAATTTCTACCCACCTTCTG........T..--...T.A................................-----....C..---..............
BG4             TATTTTCTACCCACATTCTG...........--...T.A............TC................T.TCTCC..C.C..---..............
BG5             --------------------.AG.....T..--.....A............TA..................-----.......----.T.....T.....
BG7             --------------------...........--.....A..........................A.....-----.......---..............
BG11            --------------------...........--.....A................................-----.......---..............
BG10            TATTTTCTA------------....C..T..GC...T.A............TC................T.TCTCC..C.C..---..............
BG2             CATTTTCTACCCACCTTCTG...........--...T.A............TC................T.TCCCC..C.C..---..............
BG1             CATTTT---------TTTTTA.........C--...T.A.............C.T............T.T.TCCCC....C..CCTCT.....C..CT..
BG0             --------------------AGGACAGC.AGAAGGT.T.CACTGCT.T.CTTCAG.TT.TTAGAG.T..TTTTTG---CTT.T---T....T.T..T.TT

                       310       320       330       340       350       360       370       380       390       400
                ....|....|....|....|....|....|....|....|....|....|....|....|....|....|....|....|....|....|....|....|
BG8             CTCCAGCACAGATGGCCTTCACATCGGGCTGCAACCACCCCAGTTTCGCCCTCCCCTGGAGGACCCTCCTGCCTTATCTCGTGGCTCTGCACTTCCTCCA
P2aTBGa         ............................................................................................C.......
NTBGa           ...............................................A............................................C.......
15iTBGa         ...............................................A............................................C.......
BG9             ...............................................A............................................C.......
BG12            ...............................................A............................................C.......
BG13            ..............CA...............................A............................................C.......
6TBGa           ..............CA...............................A............................................C.......
BG6             .......................A.......................A............................................C.......
BG3             ...............................................A..................C.A...T...................C.......
BG4             ............................A......A.....T.......................G..A...T.......C...........C.......
BG5             ......T....................................A...A................A...............C...........C.......
BG7             ..............CG...............................A............................................C.....G.
BG11            ..............CG............A..............A...A............................................C.....G.
BG10            ........................T..........A...........A................................A...........C.......
BG2             ..............CG........T...A..................A....T.......................................CA......
BG1             ...T..........CA...TCT..T......................A....T.......................................C.......
BG0             .T............TGG...GT...A........A..GT................................G....C...........CA..C.....TG
```

```
                      410       420       430       440       450       460       470       480       490       500
            ....|....|....|....|....|....|....|....|....|....|....|....|....|....|....|....|....|....|....|....|
BG8         GCCGGGATCAGCCCAGCTCACGGTGGTGGCACCGAGCCTCCGTGTCACTGCCAATGTGGGACAGGACGTTGTGCTGCGCTGCCACTTGTCCCCATGCAAG
P2aTBGa     ................AG............ ........................C.............................................
NTBGa       ................A....................................TC...........T.................................
15iTBGa     .................AA.G.................................C...........T...............................T.
BG9         ................AG....................................C..............C......T.......................
BG12        ..T...................................................C...........T......T..........................
BG13        ..T...C...............................................C...........T......................G...T......
6TBGa       ..T...C...............................................C...........T......................G...T......
BG6         ....................G................................TC...........T..C...................G...T......
BG3         ............T.....T..A.....A..........................C...........T..C..............G........T......
BG4         ............T.....T..A.....A..........................C...........T..C..................A....T......
BG5         ............T.....T..A.....A.........................TC...........T..C.......................T......
BG7         ..T.............AG...................................TC...........T..C.A.....................T......
BG11        ..T.............AG....................................C...........T..........................T......
BG10        .....C..........A.....................................C...........T......................G...T......
BG2         ................AA..G................................TG..............C..............G........T......
BG1         ....................G................................TC...........T.................G........T......
BG0         T..A.........A......G..........TT..A.T...C...........C............T..C.......A...............T......

                      510       520       530       540       550       560       570       580       590       600
            ....|....|....|....|....|....|....|....|....|....|....|....|....|....|....|....|....|....|....|....|
BG8         GATGTTCGGAATTCAGACATCAGATGGATCCAGCAGCGGTCCTCTCGGCTTGTGCACCACTACCGAAATGGAGTGGACCTGGGGCAGATGGAGGAATATA
P2aTBGa     ....................................................................................................
NTBGa       ....................................................................................................
15iTBGa     ....................................................................................................
BG9         ....................................................................................................
BG12        ....C.....GC........................................................................................
BG13        ....C.T...GA.TG..............................G.TT...............A.G......A........A.................
6TBGa       ....C.T...GA.TG..............................G.TT...............A.G......A........A.................
BG6         ....C.T...GA.TG................T.............G.TT...............A........A........A...A...C.........
BG3         ....CCT...GC.................A....T..........G..A..............GA.G.................................
BG4         ....CCT...GC.................A....T..........G.CA..............AA.G.................................
BG5         ....C.T...GC.................A....T..........G..A...............A........A........A...A...C.........
BG7         ..................................T..........A..A...............A.......T.........AT................
BG11        ..................................T..........G..A...............A.......T.........AT...............G
BG10        A...CCT...GC.....T................T..........G.TT........................A........A.......ACA.......
BG2         ....C........................................G..A...............A.G...............A.......ACA.......
BG1         ....CCT...GC.......................C...A.....G.TT...............A........A........A...A.............
BG0         ....C.....GC.TG....................C.........G.T................A........A........AA................

                      610       620       630       640       650       660       670       680       690       700
            ....|....|....|....|....|....|....|....|....|....|....|....|....|....|....|....|....|....|....|....|
BG8         AAGGGAGAACAGAACTGCTCAGGGATGGTCTCTCTGATGGAAACCTGGATTTGCGCATCACTGCCGTGACCTCCTCTGATAGTGGCTCCTACAGCTGTGC
P2aTBGa     ...............................................................................C....................
NTBGa       ................................................................T...................................
15iTBGa     ................................................................T...................................
BG9         ....................................................................................................
BG12        ........................................................................................A...........
BG13        ...........................................T....................T....G..................A...........
6TBGa       ...........................................T....................T....G..................A...........
BG6         .......G.........................A..............................T....G.A....C...........A...........
BG3         ....................................................A.........T.T....G.........C........G...........
BG4         .......G.........................A..................A...........T....G.A....C.....C.....A...........
BG5         .................................A..............................T....G......C...........A...........
BG7         .......G....................................................T...T....G.........C.......TG..........T
BG11        .......G....................................................T...T....G.........C.......TG..........T
BG10        .......G...............A.G......................................T....G......C..........TG...........
BG2         .......G......T........A.G............................A.........T....G............A..........T...CAT
BG1         .......................A.G......................................T....G.A............................
BG0         .......G.................C.................T...T......A...T.....A....G......C.....C.....A..T.TG..CA.

                      710       720       730       740       750       760       770       780       790       800
            ....|....|....|....|....|....|....|....|....|....|....|....|....|....|....|....|....|....|....|....|
BG8         TGTGCAAGATGGTGATGCCTATGCAGAAGCTGTGGTGAACCTGGAGGTGTCAGACCCCTTTTCTATGATCATCCTTTACTGGACAGTGGCTCTGGCTGTG
P2aTBGa     ....................................................................................................
NTBGa       ....................................................................................................
15iTBGa     .......A.........G..................................................................................
BG9         ......................................................T.............................................
BG12        ......................................................T........CCA..................................
BG13        .........C............................................T........CCAA...G...A.CC.....AG...............
6TBGa       .........C............................................T........CCAA...G...A.CC.....AG...............
BG6         ......G..........G.........C.........G................T........CCA....G...A.CC.....AG...............
BG3         ...T.............G.........C.....T...G.G..............T.T......CCA....G...A.CC.....AG...............
BG4         ...........A.....G.........C.........G................T........CCAC.......A.CC.....AG...............
BG5         .................G.........C.........G................T........CCA....C.T.A.CC.....AG...............
BG7         ...........AC....G....................................T........CCA....G...A.CC.....AG...............
BG11        ...........AC....G....................................T.T......CCA....G...A.CC.....AG...............
BG10        .................G.............T.....G.G.......................CCA....G...A.CC.....AG...............
BG2         ...........A.....G....T...............................T........CCA........A.CCT....AG...............
BG1         .....T...........G.........C.........G.........................CCA.....C..A.CC.....AG...............
BG0         ...........A..GC.G.....T.......A...........C....A.....T........CCAT..TG...A.CC.....AG...............
```

```
                 810       820       830       840       850       860       870       880       890       900
          ....|....|....|....|....|....|....|....|....|....|....|....|....|....|....|....|....|....|....|....|
BG8       ATCATCACACTTCTGGTTGGGTCATTTGTCGTCAATGTTTTTCTCCATAGAAAGAAAGTGGCACAGAGCAGAGAGCTGAAGAGAAAAGATGCAGAGTTG-
P2aTBGa   ...................................................................................................-
NTBGa     ...................................................................................................-
15iTBGa   ...................................................................................................G
BG9       ...................................................................................................-
BG12      ...................................................................................................-
BG13      ..AG..........................A...T..C.......T............C...................----------------------
6TBGa     ..AG..........................A...T..C.......T............C...................----------------------
BG6       G..G.....A....C................A...T.........TG...G.......C...................----------------------
BG3       G.TG..........................A..............T....G.......C.............CAT..----------------------
BG4       ..TG..........................A..............T....G.......CCT.................----------------------
BG5       ...G..........................A...T..C.......T....G.......C...................----------------------
BG7       G..G.....A...............C....A...T..........TG...G..........T................----------------------
BG11      G..G.....A....................A...T..C.......T....G...........................----------------------
BG10      ...G.....A....................A...T..C.......T....G.......C.A..............T....A.A..........AT....-
BG2       G....A.TT.C.....C.............A.............TT.........G..CC..................----------------------
BG1       ...G.....A....................A...C..........T....G.......CA..TG...C..C.A...A.----------------------
BG0       G..C........T..T...C......C...A...T.....................C...C.......C..A.A.T...----------------------

                 910       920       930       940       950       960       970       980       990      1000
          ....|....|....|....|....|....|....|....|....|....|....|....|....|....|....|....|....|....|....|....|
BG8       -----------------------------------------------------------------------------------GTGGAGAAAGCTGCAGC
P2aTBGa   -----------------------------------------------------------------------------------.................
NTBGa     -----------------------------------------------------------------------------------.................
15iTBGa   TGGAGAAAGCTGCAGCATTGGTGAGAAAAGATGCAGCACTGGCGGAGAAAGTTGCAGCATTGGAGAGAAAAGATGCAATGTTG.................
BG9       -----------------------------------------------------------------------------------.................
BG12      -----------------------------------------------------------------------------------.................
BG13      -----------------------------------------------------------------------------------A....CC..........
6TBGa     -----------------------------------------------------------------------------------A....CC..........
BG6       ----------------------------------------------------------------------------------------------------
BG3       ----------------------------------------------------------------------------------------------------
BG4       ----------------------------------------------------------------------------------------------------
BG5       ----------------------------------------------------------------------------------------------------
BG7       ----------------------------------------------------------------------------------------------------
BG11      ----------------------------------------------------------------------------------------------------
BG10      --------------------------------------------------------------------------------------..TC...T..TGA.
BG2       ----------------------------------------------------------------------------------------------------
BG1       ----------------------------------------------------------------------------------------------------
BG0       ----------------------------------------------------------------------------------------------------

                1010      1020      1030      1040      1050      1060      1070      1080      1090      1100
          ....|....|....|....|....|....|....|....|....|....|....|....|....|....|....|....|....|....|....|....|
BG8       ATTGGAGAGAAAAGATGCAGAGTTGGCGGAACAAGCAGCGCTATCGAAGCAAAGAGATGCAATGTTGGAGAAACACGTTCTAAAACTGGAGGAAAAGACA
P2aTBGa   .....................................................................C..............................
NTBGa     .........................................A...........................C..............................
15iTBGa   .................A..................................................................................
BG9       ....................................................................................................
BG12      ....................................................................................................
BG13      ...........................T.....T..AT.A.A.....GGTTT...T.C..A..GTC..A..C..TTA.C.TC.......A.C.....TG.T
6TBGa     ...........................T.....T..AT.A.A.....GGTTT...T.C..A..GTC..A..C..TTA.C.TC.......A.C.....TG.T
BG6       ----A...............C...........T..AT.A.A.....GGTTT...T.C..A..ATC..A.....TTA.C.TC.......A.C.....TG.T
BG3       ----A..............AT....---------------------GGTTT...T.C..A..ATC..A..G..TTA.CCTC.......A.CA....TG.T
BG4       ----A..............AT.....G.TCT.CCCAT.GCA.CAGCTGTTT...T.C..A..ATC..A...CC.TA.CCTC.......A.C.....TG.T
BG5       ----A..G.G..........CAC.........T.C.T...A...T.GGTGT.T.T.C.....ATC..A...TC.TA.C.TC.......AT.A..C.A.TG
BG7       ----A..G............CAC.........T.C.T...A...T.GGTGT.T.TAC.....ATC..A...TC.TA.C.TC.......AT.A..C.A.TG
BG11      ----A..G............CAC.........T.C.T...A...T.GGTGT.T.TAC.....ATC..A...TC.TA.C.TC.......AT.A..C.A.TG
BG10      .A..AGA.............T.C...A.....T.C.T...A...TAG.TTC...T.C.....ATC..A...T..TA.C.TC........T.A..C.A..T
BG2       ----A...............CA....A...GA....T..AA.G.T.GGAAG..A.............A.A....TGAC.GC..TGTG.------------
BG1       ----A..G..................A..G.ATG.AT..AAAG.T.GG.AC.CT..C....GAAC......G.AGG.A.GC...GT.....AC.CTAGTT
BG0       ----A...T...........T.....A.....T.C.T.T.A...T.------------------------------------------.T.A....A.TT

                1110      1120      1130      1140      1150      1160      1170      1180      1190      1200
          ....|....|....|....|....|....|....|....|....|....|....|....|....|....|....|....|....|....|....|....|
BG8       GACGAAGTGGAGAATTGGAATTCAGTGCTGAAGAAAGACAGTGAAGAGATG------------------------------------------GGTTATG
P2aTBGa   ................................A..................------------------------------------------.......
NTBGa     ................................A..................------------------------------------------.......
15iTBGa   ...................................................------------------------------------------....C..
BG9       ...................................................------------------------------------------.......
BG12      ...................................................------------------------------------------.......
BG13      ............C....C....T..AT............T...........------------------------------------------C...C..
6TBGa     ............C....C....T..AT............T...........------------------------------------------C...C..
BG6       ............G....C.......A.............T...........------------------------------------------....C..
BG3       ..T.........G....C.......A.............T...........------------------------------------------....C..
BG4       ............G....C.......A..........C..T...........------------------------------------------....T..
BG5       ..AA..T......T.CA.........CT..G.....TGGTA...GA.T.C.-------------------------------------------------
BG7       ..AA..T......T.CA.........CT..G.....CGGTA...GA.T.C.-------------------------------------------------
BG11      ..AA..T......T.CA.........CT..G.....CGGTA...GA.T.C.-------------------------------------------------
BG10      ..AA..T....C.TAC........C.AA........C.GTA....AT..CAGAGAAACAAGCTGCAGAACTGGAGAAACACTTAATAAATACC.A..TAA
BG2       ----------------------------------------------------------------------------------------------------
BG1       ..AA.TC......GAA.A..CA...A.T.C----------------------------------------------------------------------
BG0       ..A...T.T.....AGA.......CAAT..----------------------------------------------------------------------
```

```
                1210      1220      1230      1240      1250      1260      1270      1280      1290      1300
          ....|....|....|....|....|....|....|....|....|....|....|....|....|....|....|....|....|....|....|....|
BG8       GCTTTGCAGAACTGAAGAAACTGGCTGCAGAACTGGAGAAACACTCTGAAGAGATG---------------------GGGACAAGGGATTTAAAGTTGGA
P2aTBGa   ......G...T.............................................---------------------.......................
NTBGa     ......G...T.............................................---------------------.......................
15iTBGa   .......................A...A............................---------------------............G..........
BG9       ........................................................---------------------.......................
BG12      ........................................................---------------------.......................
BG13      ..G.......T...............AG..........G..A.T...AC..T...T---------------------T.....T.....G...T....A.
6TBGa     ..G.......T...............AG..........G..A.T...AC..T...T---------------------T.....T.....G...T....A.
BG6       ..G.......T......G...........A..T.....G..T.TAT..C..T..AT---------------------C...G....A..G........A.
BG3       ..G.......T......G....................G.................---------------------...CT.T..AG.G..CC....A.
BG4       ..G.......T......G....................G.....C...TC.T...T---------------------T......................
BG5       --------------G..G.............T......G....TCT..CT....A.---------------------.ATTT...CAC.GC.G.TC..A.
BG7       --------------G..G.............T......G....TCT..CT....A.---------------------.ATTT...CAC.GC.G.TC..A.
BG11      --------------G..G.............T......G....TCT..CT....A.---------------------.ATTT...CAC.GC.G.TC..A.
BG10      .TGC......T.....C.T.GCA......A.......C.....AA.......AC..GACAAATGGAAATCAGCACTGAA..T.CAAT..GA........G
BG2       --------------G.TTT.AGT......A.T...---------------------------------------------------------------A.
BG1       -----------GCA........A...T........------------------------------------------.A..G....A..GC.C.......
BG0       --------------.......AA.T...........T.G....AAT......AT.T---------------------.A..A.GA.A...C.C.A...A.

                1310      1320      1330      1340      1350      1360      1370      1380      1390      1400
          ....|....|....|....|....|....|....|....|....|....|....|....|....|....|....|....|....|....|....|....|
BG8       GCGACTAGCTGCCAAACTGGAACATCAAACTAAAGAATTGGAGAAACAGCATTCACAGTTCCAGAGACACTTTCAGAATATGTATTTAAGTGCTGGAAAA
P2aTBGa   ....................................................................................................
NTBGa     ....................................................................................................
15iTBGa   ........................A.G...............C....................C....................................
BG9       ....................................................................................................
BG12      ....................................................................................................
BG13      .GT.................G...CA..G........................T.......T.C............C.........C.............
6TBGa     .GT.................G...CA..G........................T.......T.C............C.........C.............
BG6       TAATA...............C...A......................................C............CG....G............T...C
BG3       ..T.................G..GA....................................T.C........C...C.....G...C...A....T....
BG4       ....A...............C...A....................................T.C........C...C.....G............T....
BG5       ..T.........A.......TGG.A....GAG...C.G.....G..TG.G........C.GAG..A...G.A.G.A..GT..GG..CGT......C...T
BG7       ..T.........A.......TGG.A....GAG...C.G.....G..TG.G........C.GAG..A...G.ACG.A..GT..GG..CGC......C...T
BG11      ..T.........A.......TGG.A....GAG...C.G.....G..TG.G........C.GAG..A...G.ACG.A..GT..GG..CGC......C...T
BG10      TTT..GT.....A..T...A.GA.A...GT..C....C...C......AAC.GA.G.AG.GG.A.AT....A.G.AG.G...GG....C.....CCT..T
BG2       AGC..AG...A...T.....TGG.AAG....GG...........G...TTTAAG.A..A.GG..CA....A..GTAG.AGG.G..A.........A...T
BG1       TAA.......T.AG.C....TG..A.....C....C.G........TT.A.........GG---..TA.GC.G...TCAT.A------------------
BG0       .AA..A..T...AG......TGG.A....T.G.......T......G..A.......A..GA..GA...T.A.AT...G...GT...........C.G.T

                1410      1420      1430      1440      1450      1460      1470      1480      1490      1500
          ....|....|....|....|....|....|....|....|....|....|....|....|....|....|....|....|....|....|....|....|
BG8       CAGAAGAAAATGGTTACAAAACTGGAGGAACACTGTGAATGGATGGTGAGAAGGAATGTAAAGTTGGAGGCAGCAGCTGTAAAAGTGGTGAGAAGGAATG
P2aTBGa   .....................................................................AT.C...........................
NTBGa     .....................................................................AT.C..............-------------
15iTBGa   ..............................................................T.....................................
BG9       .......................................................................................-------------
BG12      .......................................................................................-------------
BG13      ......G..C...........T.................CA.....A...........C.........................C..-------------
6TBGa     ......G..C...........T.................CA.....A...........C.........................C..-------------
BG6       .........C........................T...........A...............T......AT.C.........T.C..-------------
BG3       .........C........................C....CA.....A........................................-------------
BG4       .........C........................C....CA.....A........................................-------------
BG5       .T.....C.CAAC...A...GT.....A.CG.AAT....GAAG...A..A.CACCT.AA....A.T.GTAT.CGT...CCT..TC..AG.CT.CAC.TG.
BG7       .T.....C.CAAC...A...GT.....A.CG.AAT....GAAG...A..A.CACCT.AA....A.T.GTAT.CGT...CCT..TC..AG.CT.CAC.TG.
BG11      .T.....C.CAAC...A...GT.....A.CG.AAT....GAAG...A..A.CACCT.AA....A.T.GTAT.CGT...CCT..TC..AG.CT.CAC.TG.
BG10      .T........ATA.AGT.G........A....AAC...GCACG...A..ATC....ATC.G..C..A..AA.CAGTA..A...TT...CTTC.CATGC.T
BG2       .T....G..CAA.C.......T...T......AAC.A..GAATCA-------------------------------------------------------
BG1       ---..ACTG.CCAAGT.TG.CACCATCC..A.TAACTTCATAGGCTATGA..AATCCCCCC..GCC.T.AACTACT..CCTCTTTCTAACCC.GA...AC
BG0       .T.......CAA...G..G.........ATTGGGTGAGTCT.CCCCA------------------------------------------AACCA.......A

                1510      1520      1530      1540      1550      1560      1570      1580      1590      1600
          ....|....|....|....|....|....|....|....|....|....|....|....|....|....|....|....|....|....|....|....|
BG8       TAAAGTTGGAGGCAGCAGCTGTAAAAGTGGGACACAAAGCTAAAGAATCAGAGAAACAGAAATCGGAGCTGAAGGAGCGCCATGAGGAGATGGGGCAACA
P2aTBGa   ...........AT.C................G..AC................................................................
NTBGa     -----------------------------..G..AC.................G........................A...........C.........
15iTBGa   ....................................................................................................
BG9       -----------------------------.....................--------------------------------------------------
BG12      -----------------------------.......................................................................
BG13      -----------------------------.....................--------------------------------------------------
6TBGa     -----------------------------.....................--------------------------------------------------
BG6       -----------------------------..G..AC........................................................--------
BG3       -----------------------------..G..AC........................................................--------
BG4       -----------------------------..G..AC...................G....................................--------
BG5       C.G.AC..---------------------.TGG.TC...T.G...C.GTG.....T.G......A...TG....AGATATTTGACAA.T..A..TTT..G
BG7       C.G.AC..---------------------.TGG.TC...T.G...C.GTG.....T.G......A...TG....AGATATTTGACAA.T..A..TTT..G
BG11      C.G.AC..---------------------.TGG.TC...T.G...C.GTG.....T.G......A...TG....AGATATTTGACAA.T..A..TTT..G
BG10      C.G..C..---------------------AAGA.AC.....G...T.CTG...G....AGCTGAAC.A...G..ATT.AGA..TCACT.T..AA.AT..G
BG2       -----------------------------.T.GTAG..T..G...........G....CTGCGAACG.A..G.ATTAA.T.T..CA..TC..--------
BG1       ACC.CGAA------------------------------------------.CA..GAG.CG..G.T.TA.A...TCTGATT..CCTC..TAC--------
BG0       .GGGA..T--------------------------------------------------------------------------------------------
```

```
          1610      1620      1630      1640      1650      1660      1670      1680      1690      1700
      ....|....|....|....|....|....|....|....|....|....|....|....|....|....|....|....|....|....|....|....|
BG8     AGCTAAAGAATCAGAGAAACAGAAATCGGAGCTGAAGGAGCGCCATGAGGAGATGGCAGAACAAACTGAAGCAGTGGTGGTAGAAACTGAAGAATAG---
P2aTBGa .....................................................................................T..---
NTBGa   ..........................................................................................---
15iTBGa .........................................A............................................C.---
BG9     -------------.............................................................................C.---
BG12    ..........................................................................................---
BG13    -------------.............................................................................---
6TBGa   -------------.............................................................................---
BG6     -------------------------------------------------------.........................T.........GC.---
BG3     -------------------------------------------------------.......................C....CT...A......C.---
BG4     -------------------------------------------------------.........................T.........GC.---
BG5     T...GC...GCTGA.A...A.CGTTG.A..A......A.A..AAT..GA.CAT..---------------------------.....A......T.---
BG7     T...GC...GCTGA.A...A.CGTTG.A..A......A.A..AAT..GA.CAT..---------------------------.....A......T.---
BG11    T...GC...GCTGA.A...A.CGTTG.A..A......A.A..AAT..GA.CAT..---------------------------.....A......T.---
BG10    CAA....C.TAGG....G.A....TGAAAT.T..G..A.A.AAAC..TA..ACA.---.............A.TG---.C....T..A..A...C.---
BG2     ----------------------------------------------------------------------------------------------------
BG1     -------------------------------------------------------C..A.C....GA.GT.TG.GTCCT.C.C.CGTG.GC---------
BG0     ---------------------------------------------------------------------------CCC..G.G.TG.C.AGCT

          1710      1720      1730      1740      1750      1760      1770      1780      1790      1800
      ....|....|....|....|....|....|....|....|....|....|....|....|....|....|....|....|....|....|....|....|
BG8     ---------------------------------------GAAAAACCATCTGAAGAATCAGATTGAGAGATGAACTGCGCCTCGCAATAAGCACAGGAGT
P2aTBGa ---------------------------------------..........G..................................................
NTBGa   ---------------------------------------.................TG................................G...C........
15iTBGa ---------------------------------------.............................................A...............
BG9     ---------------------------------------.....................GG..............................A..G...C........
BG12    ---------------------------------------.............................................................
BG13    ---------------------------------------.............................................A...............
6TBGa   ---------------------------------------.............................................A...............
BG6     ---------------------------------------.................C.TG.......A.............A..G...C........
BG3     ---------------------------------------.................C.TG.......................A..G...C........
BG4     ---------------------------------------.................C.TG.......................A..G...C........
BG5     ---------------------------------------............A.....CAG.......................A..G...C........A
BG7     ---------------------------------------............A.....CAG.......................A..G...C........A
BG11    ---------------------------------------............A.....CAG.......................A..G...C........A
BG10    ------------------GTGGTTGAAACTAAAGAATTG...C........A.....CAG.......................A..G...C........
BG2     ------------------AAGGAAGAAGCTGAATAAGTG.................C.TG.......................A..G...C........
BG1     ---------------------------------------------A..CA..GGACTA..ATC...CCC.GA..AACAAT..GAAG.ACGAG..T.AGA.
BG0     GTCCCTCCTCACTTCCGTTGCTTTTCTCTTTCTTTTCCT.G.....A..T..................A.....T..T........G...C......TT.

          1810      1820      1830      1840      1850      1860      1870      1880      1890      1900
      ....|....|....|....|....|....|....|....|....|....|....|....|....|....|....|....|....|....|....|....|
BG8     TAAGCTTCATAGATCAATAACTGCACAGCATACA-AAACCACAATAACTCAAACAGAGTAAGGA--------------------GGAGCCAGTGTTTGTG
P2aTBGa ..................................-......................G......--------------------................
NTBGa   ..................................-......................G......--------------------................
15iTBGa ..................................-........................C....--------------------...............A
BG9     ..................................G.......G...C......A....C.....AATCCACAGCGAGAACAAGA................
BG12    ..................................-.............................--------------------................
BG13    ........T.........................-.............................--------------------................
6TBGa   ........T.........................-.............................--------------------................
BG6     ..................T...A.........A.--.T....G..TC.A.........C.....AATCCA---CGAGAACAAGA................
BG3     ..................T...A.........A.--.T....G..TC.A.........C.....AATCCACAGCGAGAACAAGA.......C........
BG4     ..................T...A.........A.--.T...TG..TC.A.........C.....AATCCACAGCGAGAACAAGA................
BG5     .............CTGC.G.T.......G...GCA.C.T.G.C.....G....GCA..A.....AATCCACACGGGGAACAAGA...............A
BG7     .............CTGC.G.T.......G...GCA.C.T.G.C.....G....GCA..A.....AATCCACACGGGGAACAAGA...............A
BG11    .............CTGC.G.T.......G...GCA.C.T.G.C.....G....GCA..C.....AATCCACATGGGGAACAAGA..........CC...A
BG10    ..........G..CTGC.G.........G...GCA.C...G.C....TG....GC...C.....AATCCACAGCGAAAACAAGA................
BG2     ..................G...............G.......G...C...........C.....AATCCACAGCGAGAACAAGA................
BG1     ATTAAA.GCAC------------.T...TTAC..CTGGTGTT..A..TC..T..T.GAAG.A..--------------------....A..........A
BG0     C......AC....CT.C.G.T....T..G.C.TCA.....CTG......TG..GCAG.C..T..AAACCACAAGGGGAACAAGAC....T.......ACA

          1910      1920      1930      1940      1950      1960      1970      1980      1990      2000
      ....|....|....|....|....|....|....|....|....|....|....|....|....|....|....|....|....|....|....|....|
BG8     TTGAGTGAGAACAC-TGCAGTTCTGTCAGCCAAAGCTGCCTGAGGGACCGCCGAATTGAGGGTGTGCGACCTCCAACTCAAAGCCAATTGGAAGAAAGAA
P2aTBGa ..............-.........C..G........................C...............................................
NTBGa   ..............-.....................................C...............................................
15iTBGa ..............-.....................................C.......................................T...G............
BG9     ..............-.....................................................................................
BG12    ..............-.....................................C...............................................
BG13    ..............-.....................................C.............T..............T...G............
6TBGa   ..............-.....................................C.............T..............T...G............
BG6     ..............-................................T...C.............T..............T...G............
BG3     ..............-................................T...C...............................................
BG4     ..............-.....................................................................................
BG5     ..............-..........CA.....C.A..............A..A..C..........T........T......T...G............
BG7     ..............-..........CA.....C.A..............A..A..C..........T........T......T...G............
BG11    ..............-..........CA.....C................AG.A..C..........T........T......T...G............
BG10    ..............-........CA.G......C.................C..........T.....................................
BG2     ..............-..............AT..G...............A..A..C..........T..............T..............---
BG1     ..............-.........G................AA...A....C..........T..............T......................
BG0     ..............C....T..T...G.C-....AT...A...A..G.TA.....CA....A..CAT..A..........T.TT..GC.....T......
```

```
                  2010      2020      2030      2040      2050      2060      2070      2080      2090      2100
           ....|....|....|....|....|....|....|....|....|....|....|....|....|....|....|....|....|....|....|....|
BG8        ACCATAGAAAGGAAGGAAAGGGGAGGGAGACAGAGATCCTGGAAGAGATATGGGCATTTGGGGAAATAGTGTGACCGTGTATCAGGCTTTGTGGACATCT
P2aTBGa    ................C.........A..........................................................................
NTBGa      ...............A..........A.................................................A.......................
15iTBGa    ..........................A.........................................................................
BG9        ....................................................................................................
BG12       ..........................A.................................................A.......................
BG13       ..........................A.................................................A.......................
6TBGa      ..........................A.................................................A.......................
BG6        ............................................................................A.......................
BG3        ....................A.....A......T..........................................A.......................
BG4        ..........................A.................................................A.......................
BG5        ..........A....ACT.CAA....A...............G.A..GG.CA.A.....T.....T..ACA..G..A.........GG.-.A...AT...
BG7        ..........A....ACT.CAA....A...............G.A..GG.CA.A.....T.....T..ACA..G..A.........GG.-.A...AT...
BG11       ..........AA...ACT.CAA....A.......T.........A..GG..A.AG.G..T.....T..ACA..G..A.........GA...A...AT..C
BG10       ...............ACT.CAA....A..................A..GG..A.A.....T....TT..ACA..G..A.........G....A...AT...
BG2        -.T............ACT.CAA....A..................A..GG..A.A.G...T.....T..ACA..G..A.........G....A...AT...
BG1        ....C..G.......A...T...T..A..................A..............T......C.........AC........A..CA....A...C
BG0        ....C.T-.......A...T...TT.A...TG...T........N....--.CA.AA....T..........A...T.A.C.....A....CA.A..A....

                  2110      2120      2130      2140      2150      2160      2170
           ....|....|....|....|....|....|....|....|....|....|....|....|....|....|...
BG8        AACGAATATGTCATGTTTTTGTAAATACAAGCATGCACGCAGAAACAAAGGTAGAAAACTGCTTTGGGTGTTA
P2aTBGa    ...................................................C.....................
NTBGa      ...................................................G.....................
15iTBGa    ........................C..........................C.....................
BG9        .........................................................................
BG12       .........................................................................
BG13       ..T............C........C................................................
6TBGa      ..T............C........C..........................C.....................
BG6        .........................................................................
BG3        ..T..G.....A.CAC...G.G.....TG.A.........T...G............................
BG4        .....GC....A...C..A..G..G..G......T...A.....G......C.....................
BG5        ..T........A.G.C..C..G.....T..A.......A.....GT...........................
BG7        ..T........A.G.C..C..G.....T..A.......A.....GT...........................
BG11       ..T..G.....A.G.C.....G........A.......A.....GT.....A.....................
BG10       ....T....A.A.G.C.....G.....T..A.........T.........................A.....
BG2        ..T.T....A.A.G.C.....G.....T..A.......A.....G......A.A...................
BG1        ..T........A.G.C.....G..----..........A.....G..G.....................A.C.
BG0        ..T........A...C.....G...C...........TATC...G.....AC.....................
```

Supplementary Figure S3. Alignment of nucleotide sequences from the "(nearly) full-length conceptual transcripts" (that is, exons without introns) for the dominantly-expressed genes from four chicken lines identified in this paper, and for the 14 BG genes of the B12 haplotype from Salomonsen et al 2014. Names of the transcripts follow the convention: abbreviated line name, "T" for T cells, "BG" and the letter "a" representing the most frequently detected clone from the most frequently detected exon 2 sequence. Names of the genes follow the convention "BG" and the number of the gene locus for the B12 haplotype. Colors indicate different coding regions (grey, 5'UTR and 3'UTR; dark green, signal sequence; light green, Ig-V domain; brown, transmembrane region; alternating yellow and red, cytoplasmic tail regions (codons from each 18, 21 or 24 nucleotide repeat); purple, in-frame stop codon; light blue, introns (including two positions that are probably nucleotide misincorporations during PCR reaction) which are all deleted in the analyses for "(nearly) full-length conceptual transcripts" (that is, exons without introns). In this figure, the colors of the nucleotides of split codons reflect the protein region rather than the exon (for instance, last amino acid of the signal sequence is assigned to the signal sequence even though the last two nucleotides of the codon are part of exon 2 that encodes the Ig-V domain). Letters indicate nucleotides, dot indicates identity with BG8 sequence; dash indicates not present in all sequences.

1.4 Supplementary Figure S4

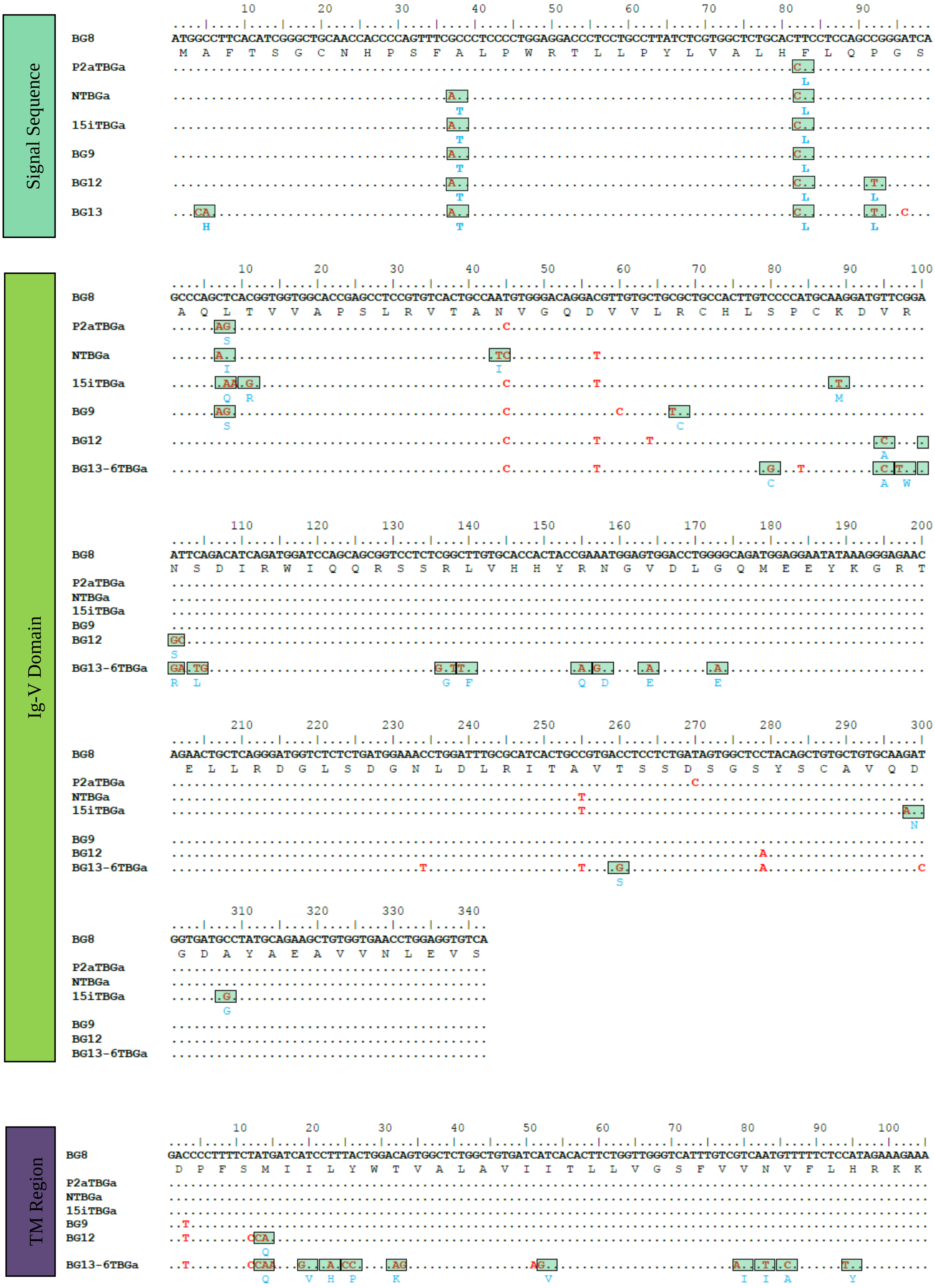

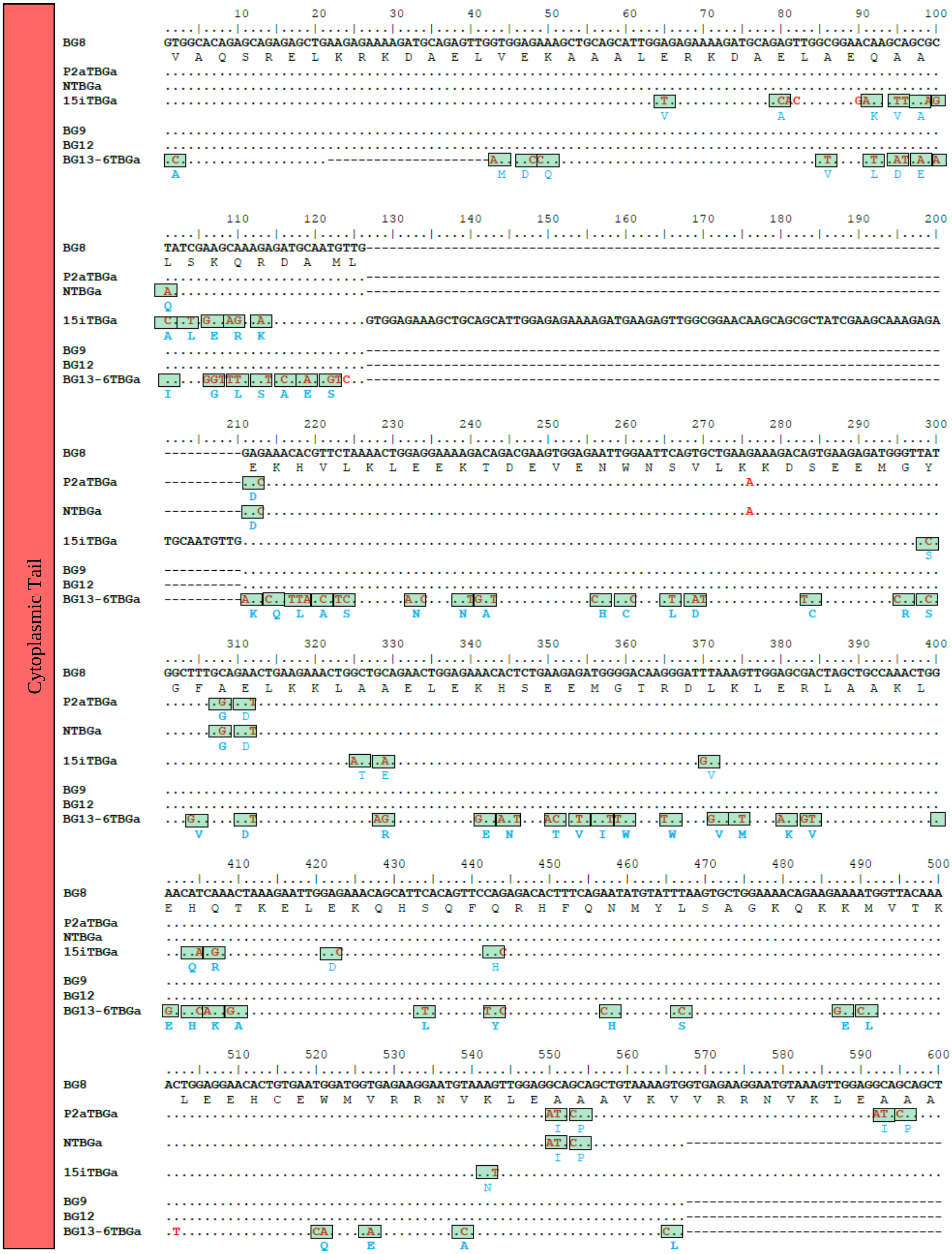
Cytoplasmic Tail
BG8
P2aTBGa
NTBGa
15iTBGa
BG9
BG12
BG13-6TBGa

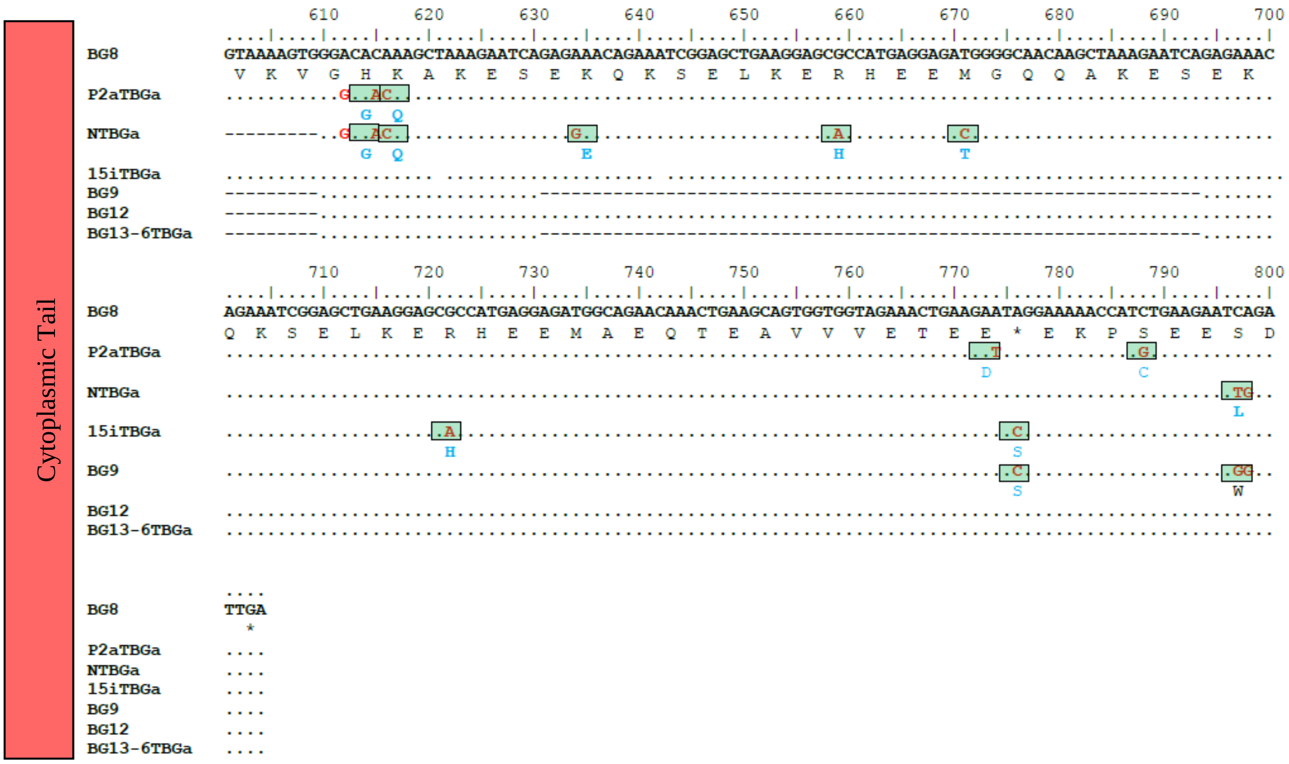

Supplementary Figure S4. Alignments of nucleotide sequences (along with amino acid positions that differ from the BG8 gene from the B12 haplotype) for portions of the "(nearly) full-length conceptual transcripts" (that is, exons without introns) of the dominantly-expressed genes from four chicken lines identified in this paper, and for the appropriate BG genes of the B12 haplotype from Salomonsen et al 2014. Names of the transcripts follow the convention: abbreviated line name, "T" for T cells, "BG" and the letter "a" representing the most frequently detected clone from the most frequently detected exon 2 sequence. Portions of the sequence analyzed are: signal sequence from exon 1, exon 2 (mostly Ig-V domain), exon 3 (transmembrane region), and exons corresponding to the cytoplasmic tail (including translated sequences in the last exon). In this figure, the amino acids from split codons at the edges of the exons are assigned to the exon with two of the three nucleotides of the codon (for instance, last amino acid of the signal sequence is assigned to the Ig-V domain, which in fact starts with glutamine in the mature protein); these split codons are not important for tallying up the amino acid differences between the sequences. The four exons apparently inserted into the cytoplasmic tail of P2aBGTa were not considered in this analysis. Codons corresponding to nucleotide changes that lead to amino acid changes are boxed.

1.5 Supplementary Figure S5

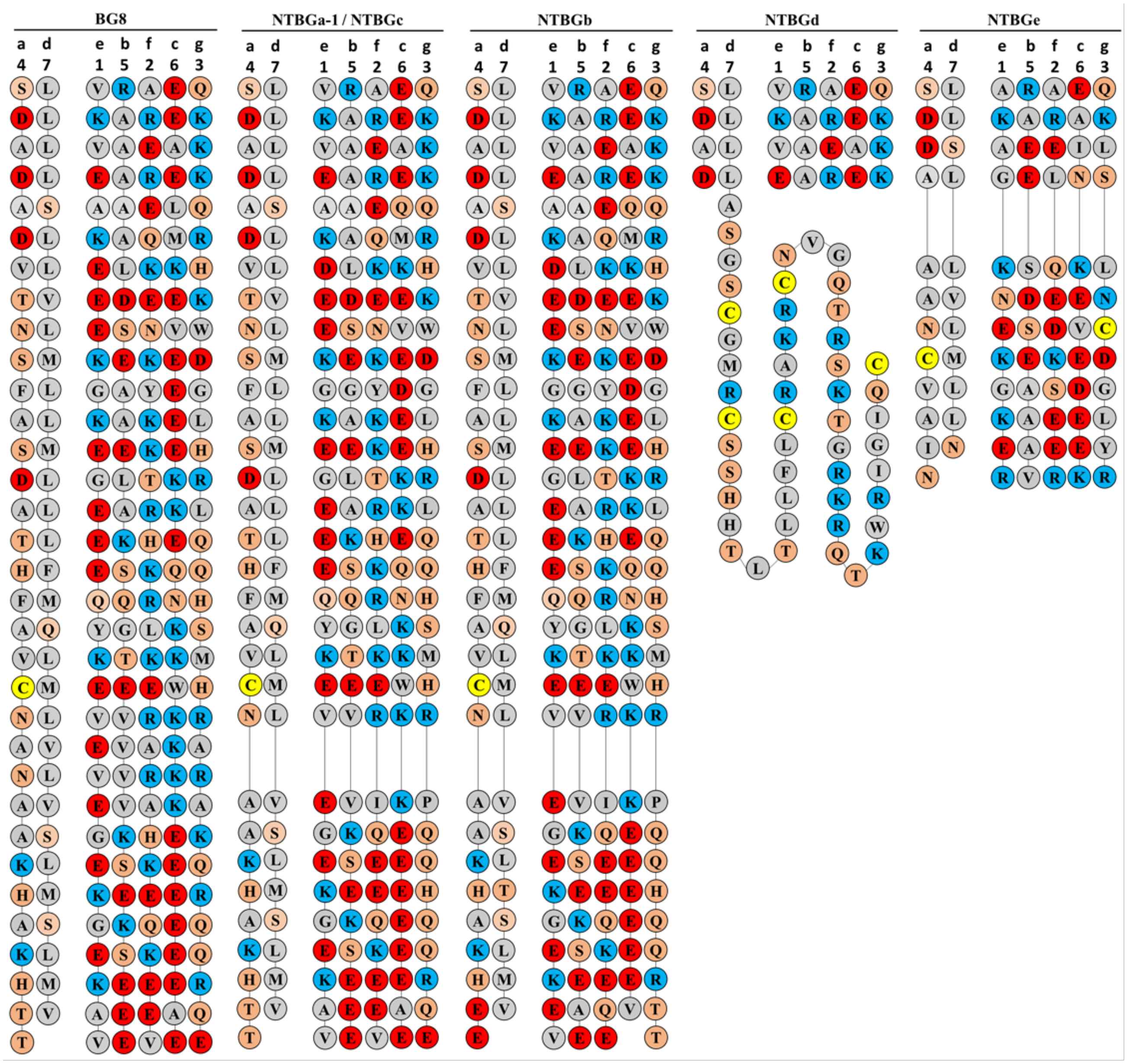

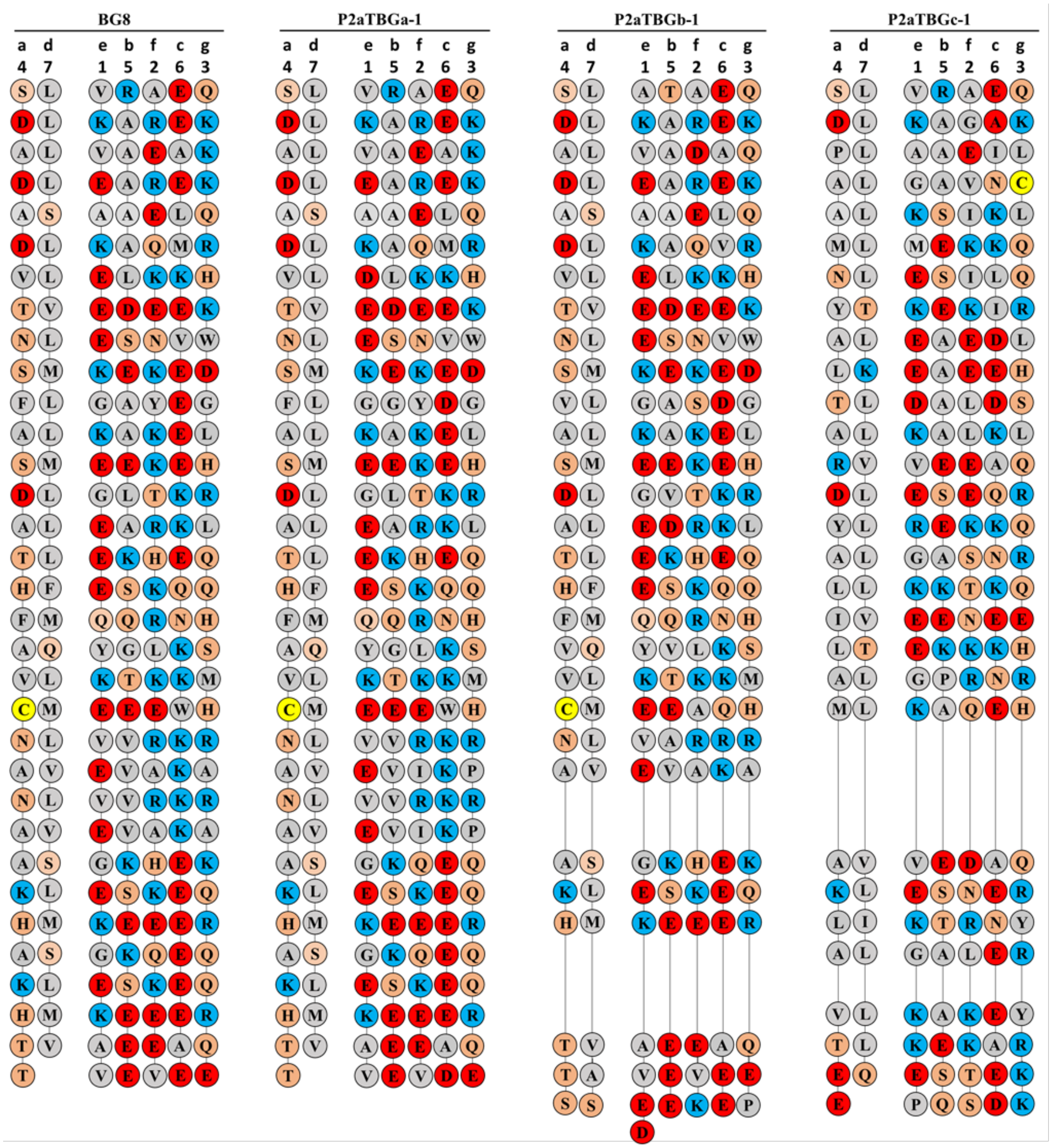
BG8
P2aTBGa-1
P2aTBGb-1
P2aTBGc-1

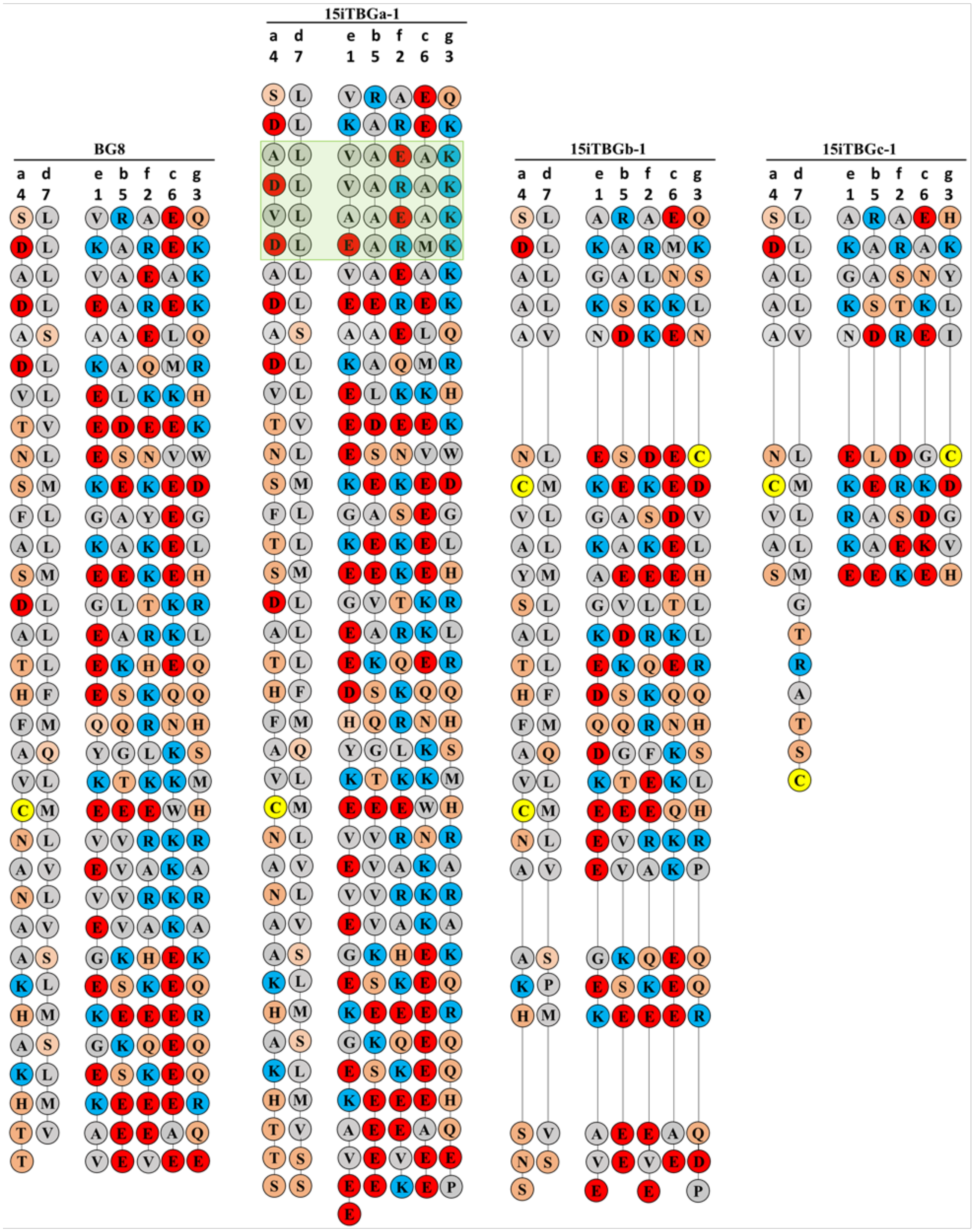
15iTBGa-1
BG8
15iTBGb-1
15iTBGc-1

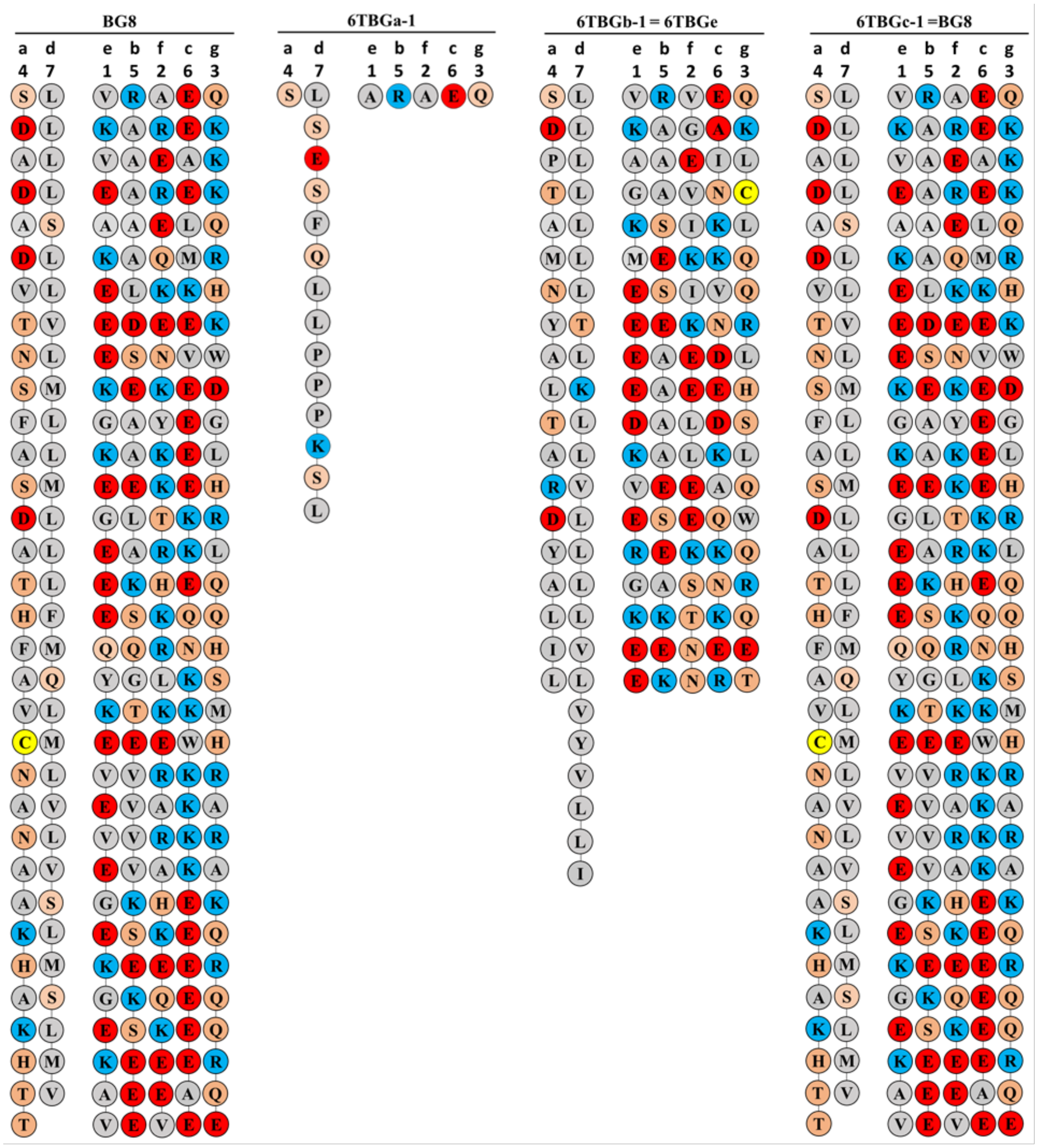

Supplementary Fig. S5. Coiled-coil representations of the cytoplasmic tails inferred from the most frequent clones for each of the 16 genes from line N (B21), line P2a (B19), line 15I (B15) and line 6_1 (B2) based on exon 2 sequences, using the real transcript (that is, exons with the intron read-throughs leading to an early stop codon. Names of the transcripts follow the convention: abbreviated line name, "T" for T cells, "BG" and the letter "a" representing the most frequently detected clone from the most frequently detected exon 2 sequence (and "b" representing the most frequently detected clone from the second most frequently detected exon 2 sequence, and so forth), followed in some cases by a dash and then a number representing the alternative splicing variant with "1" being the most frequently detected clone. The transmembrane region would be at the top of the page, so the C-terminus of the BG protein is at the bottom of the page. The positions of the seven codons in the 21 nucleotide repeat are indicated with numbers at the top, and the position of the seven amino acid positions of the "true heptad repeat" are indicated with letters. Colors of circles surrounding the amino acids (single letter code) indicate features of the amino acids (red, acidic; blue, basic; orange, polar; grey, hydrophobic except for yellow, cysteine), with the full understanding that these features do not correspond to full descriptions of the properties of the amino acids. The amino acids encoded by the four extra exons inserted in 15iBGTa are indicated by a light green box, and elsewhere the positions of the heptads are shifted to illustrate sequence similarities or identities between cytoplasmic tails.
